# Recent Advances in the Use of Ionic Liquids and Deep Eutectic Solvents for Lignocellulosic Biorefineries and Biobased Chemical and Material Production

**DOI:** 10.1021/acs.chemrev.4c00754

**Published:** 2025-06-06

**Authors:** Pedro Verdía Barbará, Hemant Choudhary, Pedro S. Nakasu, Amir Al-Ghatta, Yinglei Han, Cynthia Hopson, Raul I. Aravena, Dhirendra Kumar Mishra, Antonio Ovejero-Pérez, Blake A. Simmons, Jason P. Hallett

**Affiliations:** † Department of Chemical Engineering and Materials, Universidad Complutense de Madrid, 28040 Madrid, Spain; ‡ Deconstruction Division, 124489 Joint BioEnergy Institute, Emeryville, California 94608, United States; § Department of Bioresource and Environmental Security, 111651 Sandia National Laboratories, Livermore, California 94551, United States; ∥ Department of Chemical Engineering, 4615 Imperial College London, South Kensington Campus, Exhibition Road, London SW7 2AZ, United Kingdom; ⊥ Biological Systems and Engineering Division, 1666 Lawrence Berkeley National Laboratory, Berkeley, California 94720, United States

## Abstract

Biorefineries, which process biomass feedstocks into valuable (bio)­products, aim to replace fossil fuel-based refineries to produce energy and chemicals, reducing environmental and health hazards, including climate change, and supporting a sustainable economy. In particular, lignocellulose-based biorefineries, utilizing the most abundant renewable feedstock on Earth, have significant potential to supply sustainable energy, chemicals and materials. Ionic liquids (ILs, organic salts with low melting temperatures) and deep eutectic solvents (DESs, mixtures with eutectic points lower than the ideal mixture) are capable of dissolving some of the key lignocellulose polymers, and even the whole biomass. Furthermore, they have intrinsic advantages over molecular solvents, including safer usage profiles and high tunability, which allow tailored physicochemical properties. Such properties provide unique opportunities for the development of new processes that could unlock the full potential of future biorefineries. Here, we review the current state of lignocellulosic biomass processing with ILs and DESs, with a specific focus on the pretreatment chemistry, process flow and products from each component; followed by discussions on sustainability assessments and technological challenges. We aim to inform the research community about the opportunities, challenges and perspectives in developing truly sustainable lignocellulose-based biorefineries.

## Graphical abstract

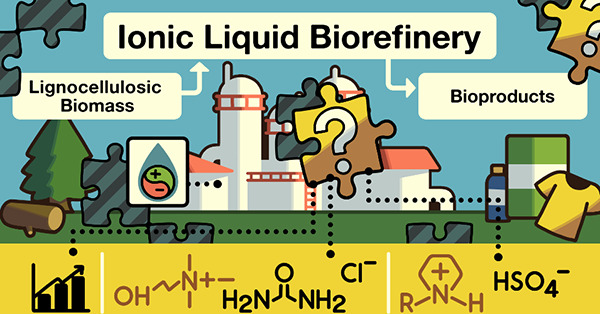

## 1 Introduction

### 1.1 Importance of Biorefining in the Global Context

The reliance of current industries on fossil feedstocks to produce energy, chemicals, and materials is a major driving force behind increasing pollution and environmental hazards, including those affecting human and environmental health and ecosystem stability.^1,2^

Furthermore, fossil feedstocks face constant issues related to price instability and are deemed to be long-term depleted. Public awareness of these issues is driving consumer pressures and policy changes to set new standards for industrial production, demanding the development of more sustainable and less hazardous processes based on the use of renewable feedstocks. Therefore, the foundations of an economy with long-term sustainability must transition to affordable and renewable supplies of raw materials. This will lead to additional benefits in different areas, including more widespread and equal access to energy that is more resilient and less dependent of geopolitics; the generation of new and safer employment opportunities; the development of new technology innovations; etc.^1–3^

The achievement of such a sustainable economy will require biorefineries to replace the current production of energy and chemicals from fossil carbon sources. Biorefineries use plant biomass as a feedstock to produce different biobased products, including biofuels and platform chemicals.^4^

### 1.2 Biorefinery Concept

Plant biomass (aka lignocellulosic biomass) is a sustainable and renewable carbon resource for bioenergy generation and the manufacturing of bio-based products. It has a range of advantages, including very low carbon footprint, since plants use atmospheric CO_2_ for their growth, abundant availability, and price stability. It has been estimated that the substitution of fossil feedstock for biomass derived feedstocks to produce chemicals can lead to a decrease of up to 86% in the emissions of greenhouse gas emissions and carbon footprint.^5^

The potential for utilizing biomass as an energy source is still limited by the efficiency of conversion processes, and a comparatively lower calorific value. Nevertheless, the integrated biorefinery is a viable approach for optimizing the utilization of biomass, including the byproducts generated from several conversion processes, and transforming them into lucrative bio-based product streams.^8^ The concept was born as an effort to highlight the necessity to achieve not only economic viability but also technical and product flexibility, with production sites capable of producing fuels, platform chemicals, materials, and polymers.^9^

A biorefinery refers to a facility that combines different deconstruction and conversion technologies, including thermochemical, biochemical, combustion, and microorganism growth platforms, to effectively generate a range of sustainable bio-based product streams.^10^ These product streams encompass biofuels, biochemicals, bioenergy, and other bio-products of significant value. The biorefinery concept has been developed over decades and employed for the purpose of processing a range of biomass feedstocks, including lignocelluloses, algae, and different forms of waste materials.^11^ The US Department of Energy (DOE) provided the first precise definition of a biorefinery in 2004: “*A biorefinery is an all-encompassing term for a processing facility where biomass feedstocks are converted and extracted into a variety of valuable products.*”^12^

Biorefineries are typically classified into four distinct categories based on the type of substrate utilized: first-generation (1G) biorefineries employ starch and sugar feedstocks; second-generation (2G) biorefineries utilize lignocellulosic biomass feedstocks; third-generation (3G) biorefineries rely on algal feedstocks; and fourth generation (4G) biorefineries use genetically modified microorganisms to create a carbon-sink.^13^ Since lignocellulosic biomass is the most abundant renewable source of materials on earth, with 180 billion tons produced per year by plants, second-generation biorefineries have the potential to be a major supplier for sustainable energy, chemical products and materials.^14^ Nevertheless, lignocellulosic biomass has a very recalcitrant structure that needs to be deconstructed to access the valuable biopolymers before they can be further processed. Traditional lignocellulose deconstruction technologies have important environmental and safety concerns. Nowadays, the Kraft process, a process developed in the XIX century, based on the use of highly alkaline solutions containing sodium sulfite, represents 90% of the pulping industry.^15^ This process presents serious environmental concerns. It has been estimated that, per ton of processed dry pulp, it consumes 45 tons of water and produces 3 kg of sulfur containing gases, 15 kg of other volatile organic compounds and 150 kg of fine particulate matter.^16^ The aqueous effluents from this process contain more than 250 different compounds, including halogenates and heavy metals, and current treatment methods can not completely decontaminate them.^16^ Furthermore, it is the fourth largest industrial energy user, accounting for 6% of global industrial energy consumption.^15^

Therefore, the development of new, cleaner and more sustainable processes is highly needed. The discovery in the early 2000s that a novel class of nonmolecular solvents called ionic liquids (ILs) could dissolve some of the key lignocellulose polymers, and even the whole biomass, opened a big opportunity for the development of new processes that could satisfy the needs of future biorefineries.^17^

### 1.3 ILs and DESs: Introduction and History

ILs are salts that can be found in the liquid state before their decomposition temperature.^18^ They present relatively low melting points due to having molecular structures that present some degree of asymmetry, charge delocalization, and weak intramolecular interactions. As salts, ILs have negligible vapor pressure at normal conditions, high thermal stability, and high resistance to flammability, reducing drastically safety and environmental concerns associated with solvent volatility (toxicity by inhalation, solvent release to the atmosphere, explosion hazards, etc.). Furthermore, the high number of possible combinations of different cations and anions makes possible to adjust their chemical-physical properties designing ILs best suited for a given application (stability, melting point, viscosity, hydrophilicity).^19–22^

Due to the nonsystematic approach that characterizes the literature regarding abbreviation conventions for naming ILs, which leads to a variety of ambiguous forms, here we will follow the guidelines proposed by Hallett and Welton in 2011, where possible.^19^ It establishes an alphanumeric system for the alkyl chains, where the alkyl chains are indicated by a capital C with the number of carbon atoms indicated as a subscript number. Branching of the alkyl chains is indicated by the corresponding superscript before the “C” (*e.g.,* with this system a *tert*-butyl chain will be indicated as *^t^* C_4_). Similarly, the presence of functional groups will be indicated by the type of functional group, followed a subscript number before the “C” indicating its position in the chain (*e.g.*, a butyl chain functionalized with an alcohol group at the terminal carbon will be indicated as (HO)^4^C_4_). The charged centers are indicated with the most common alphabetical abbreviations (“im” for imidazolium, “py” for pyridinium, “pyrr” for pyrrolidinium, “N” for ammonium, “P” for phosphonium, etc). The most common anions and cations studied today are reported in Figure 1.

**Figure 1:**
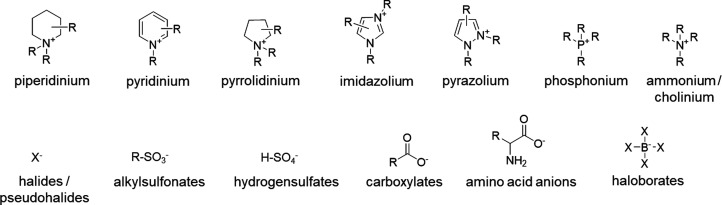
Common cations and anions used for synthesis of ILs.

Eutectic solvents (ESs) have been traditionally defined as mixtures of a hydrogen bond accepting (HBA) salt and a hydrogen bond donor (HBD) molecule with lower melting points than their precursors. In 2003, Abbott *et al*. coined the term “*deep eutectic solvents*” to describe the decrease in melting point of the liquid obtained by mixing two solid components, choline chloride ([Ch]­Cl, 302 °C) and urea (133 °C), in stoichiometric proportions, which resulted in the formation of a eutectic solution with a remarkably low melting point (12 °C).^23^ Since then, the definition of Deep Eutectic Solvents (DESs) has been revised as mixtures of pure compounds that have an eutectic point temperature that is significantly lower than that of the ideal mixture, including mixtures of both Brønsted and Lewis acids and bases.^24^ As with ILs, DESs offer high design flexibility with tunable properties and are also considered as designer solvents.

The close relationship between ILs and DESs has led the scientific community to often treat DESs as an extension of ILs research; with both types of compounds being investigated for the same applications, most notably for the dissolution, fractionation and purification of biopolymers, and their performances compared.^25^ In fact, in many cases, a wide range of DESs employed in the literature are prepared using ILs as one of the starting materials, normally as the HBA. Moreover, there is some overlap between DESs with protic ionic liquids (PILs) and even certain mixtures of ILs and solvents as water (Figure 2).^26^

**Figure 2:**
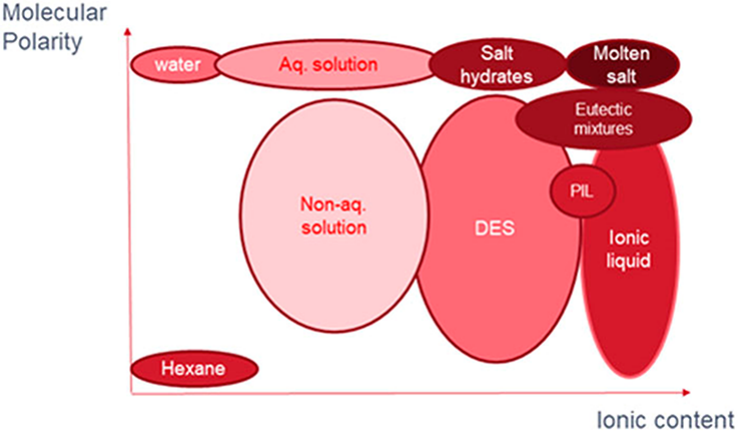
Representation of the chemical space, in terms of ionic content and polarity of their molecular component, occupied by the types of solvents under review in that document, DESs and ILs, compared to other related solvent systems as depicted by Abbot *et al*.^26^ Adapted with permission from ref 26. Copyright 2021 American Institute of Physics.

### 1.4 Scope of This Review

This review provides an overview of IL and DES-based biorefinery scenarios (*e.g.*, lignin-first, lignin-last, simultaneous conversion) with a specific focus on the pretreatment chemistry, process flow and products from each component (including cellulose, hemicellulose, lignin, lipids and extractive products); followed by discussions on sustainability assessment and technological challenges (scaling, IL/DES recycle and recovery, potential product recovery and integration of upstream and downstream processes) around each specific scenario. Since the technological challenges around solvent cost and recovery in biorefineries are an active research frontier, key to ensure their success, it is our aim to provide a critical report about the opportunities, challenges and perspectives related to the use of ILs and DESs in biorefining. The intent is to inform the research community working in this field, to keep making progress towards a truly sustainable lignocellulose-based biorefinery.

## 2 Discussion

### 2.1 Biorefinery Types and Generations

The next section provides a more comprehensive discussion of different types of biorefineries, along with a corresponding list of examples and their associated products, as seen in Figure 3. The four generations of biorefineries will certainly play a considerable role in the global society to achieve the goals of sustainable development and implementing a circular economy. Nevertheless, generations 1, 3, and 4 still present issues related to land use, feedstock availability, and technology development. Due to its feasibility and inherent potential to help mitigate GHG emissions, we will focus on the application of ILs for the development of 2G biorefineries, based on the conversion of lignocellulose.

**Figure 3:**
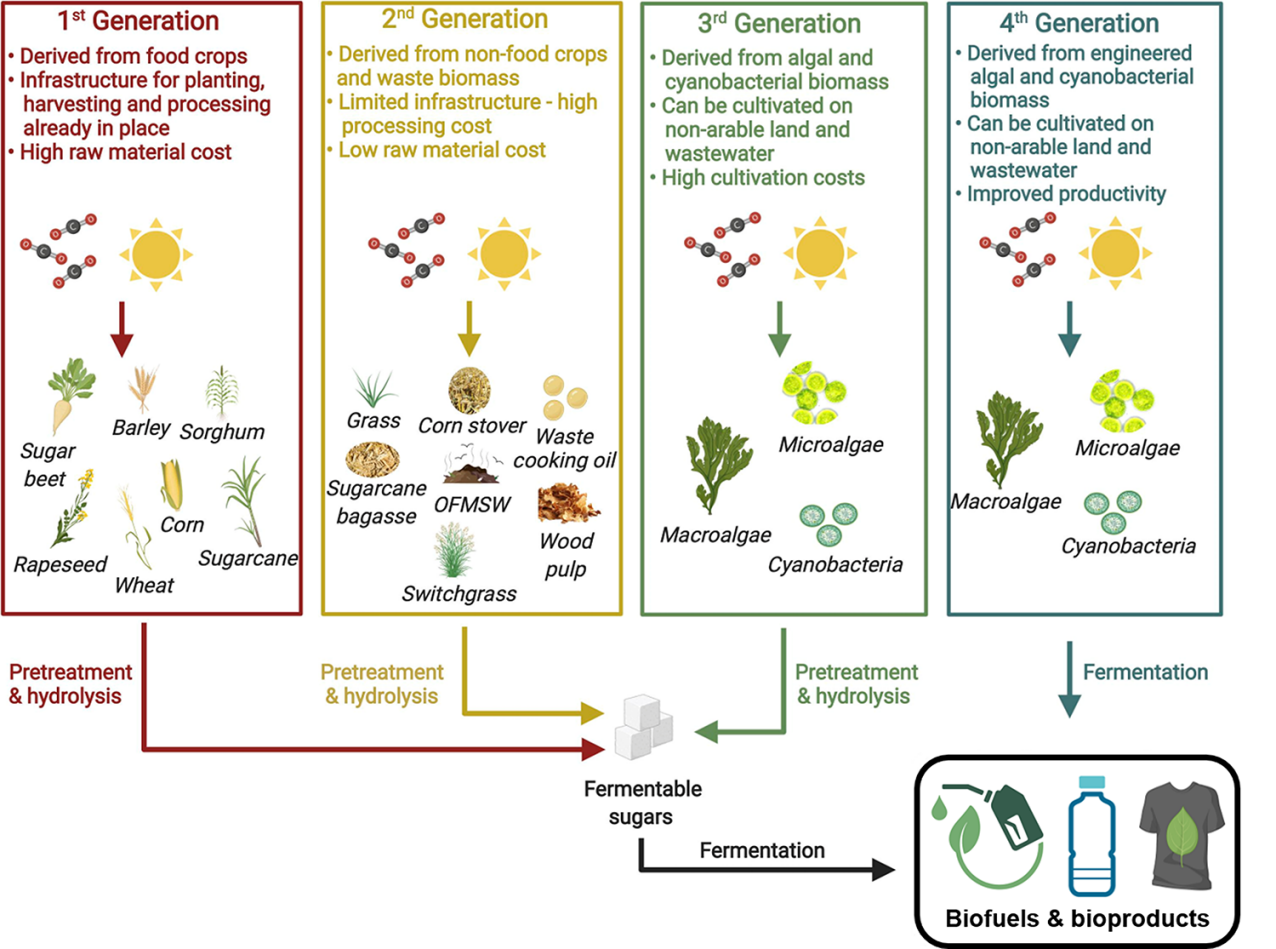
Illustrations of possible feedstocks are depicted alongside the advantages and disadvantages associated with each generation of biofuel. Adapted with permission from ref 13. We also want to highlight that the 2G feedstock is an abundant feedstock in contrast to the note by authors on limited availability.^27,28^ Adapted with permission from ref 13. Copyright 2023 the Public Library of Science under CC BY 4.0 (https://creativecommons.org/licenses/by/4.0/).

#### 2.1.1 First-Generation Biorefineries

1G biorefineries, also known as conventional biorefineries, utilize feedstocks derived from glucose, sugar and vegetable oil-containing crops. Corn, wheat and sugarcane are used to produce bioethanol, while soybeans and rapeseed are used to produce biodiesel.^29,30^ In 2012, approximately 40% of the maize crop in the United States was used to produce ethanol, making corn the primary source of first-generation bioethanol.^31^ Sugarcane is the second most important feedstock for first-generation biofuel production, such as ethanol. Unlike corn, sugarcane provides sugar that can be readily converted to ethanol through fermentation. Corn, on the other hand, provides starch that must be heated prior to fermentation.^32^ The feedstock is first subjected to enzymatic hydrolysis (for starch) and fermentation, followed by distillation to separate the biofuel from the byproducts.^33^ Other resources, such as wheat, rapeseed, sugar beets and peanuts, could be used as well.^34^

Although first-generation biofuels have been extensively commercialized, they have a number of drawbacks.^35^ The crops used to produce first-generation biofuels are the same used to feed humans and animals, a sensitive topic with potential ethical implications.^36^ Moreover, large-scale monocultured feedstocks further endanger food chains, while cultivation of these resources outside of conventional agricultural regions imperil biodiversity by occupying more land and invading natural ecosystems. However, the effect of the production of first-generation biofuels on food prices is far from being clear, with many factors playing a role. There is an intense debate around this issue, with no consensus so far among researchers.^37^

#### 2.1.2 Second-Generation Biorefineries

The contentious relationship between 1G feedstocks and food security and the potential of the conversion of agricultural and forestry residues into valuable products has led to the transition to 2G lignocellulosic feedstocks as an alternative source for biofuels and chemicals production.^7^ 2G biorefineries use non-edible lignocellulosic biomass, including non-food crops, agricultural and forestry byproducts and waste materials from different manufacturing processes.^38^ Hence, these largely avoid the competition for land of agricultural value. Reduced needs of fertilizer also help mitigate the carbon footprint. Another key advantage is its availability: lignocellulosic biomass is the most abundant renewable material on the planet. Globally, approximately 180 billion tons of lignocellulosic biomass are produced annually, primarily by perennial herbaceous and woody plant species, of which only about 4.4% are utilized for producing biochemicals, bioenergy and non-food bioproducts.^14^ Thanks to the diversity of plant options and high availability in tropical and temperate regions, lignocellulosic materials are a practical source of biomass feedstock for biorefineries.^39^

2G biomass feedstocks can be divided into three categories according to their origin. Plants harvested for cellulose production are considered primary cellulose sources. For example, cotton is grown as a feedstock for the textile industry, while tree species like pine, spruce, and *Eucalyptus* are used to feed pulp and paper mills. Similarly, some fast-growing crops such as *Miscanthus*, willow, and poplar can be harvested for energy production. Even though these materials are non-edible, and some of them can be grown on land not suitable for food production and of low ecological value, in some cases their production might compete for arable land with food crops or require the clearing of forest for new land, prompting a new “forest versus fuel” debate. By-products of forestry and agricultural production are considered secondary cellulose sources. Maize stover, rice or wheat straw and oil palm empty fruit bunches fit in this category. Finally, tertiary cellulose sources include cellulose-containing by-products and waste materials from different manufacturing processes, such as construction and demolition (C&D), breweries, textile industry, etc.^38^ These waste streams have low or even negative value, due to the costs associated with their disposal. While some of them are incinerated to produce electricity, landfilling is the main means of disposal for these streams.^40^ As a result, waste is released to the environment, producers are economically penalized and a stream of potentially valuable feedstocks is lost.^41^ Their potential valorization would open the door for a cheap raw material that is available locally even in areas, where access to primary and secondary cellulose is limited while reducing the costs associated with waste disposal.^38^ On the other hand, tertiary cellulose is usually found in complex mixtures with other components, such as other biopolymers and biomolecules and different types of contaminants including preservatives, resins and paints, plastic, sand and glass, etc., which can make their valorization challenging.^38,42^ There have been several studies that have presented techno-economic analyses of second-generation biorefineries that use ILs and DESs.^43–45^

#### 2.1.3 Third-Generation Biorefineries

Typically, 3G biorefineries use algae and seaweed as feedstocks for bio-renewables production.^46,47^ Microalgae are aquatic unicellular biomass mainly formed by proteins, carbohydrates and lipids.^48^ The primary advantages of microalgal biomass for biodiesel production are their rapid growth rate, high photosynthetic efficiency and low cultivation costs since they can be grown in sewage and wastewater. Nevertheless, their growth and lipid content are highly dependent on cultivation conditions: temperature, light intensity, CO_2_ concentration, pH value and nutrient composition of the culture medium.^49^ Algal crops are well-known commercial producers of nutraceuticals, animal feed and feed supplements and numerous other goods. The objective of third-generation biorefineries is to use microbial cell factories to utilize atmospheric CO_2_, sunlight, inorganic compounds from waste streams, and electricity generated by sustainable sources (*e.g.*, photovoltaic cells and wind power) for bioproduction.^50,51^ Third-generation biorefineries reduce the cost of processing feedstock and pose fewer security risks to food and water supplies. On the other hand, the availability of sunlight and the maintenance of inorganic ions’ pH pose the greatest difficulty in microalgal cultivation. Sunlight availability can be increased by constructing the system from low-cost acrylic-type material.^52^ The lack of sunlight during the night can be compensated for by providing artificial illumination, which will ultimately increase the cultivation system's overall yield.^53^ However, the efficient capture of renewable energy for bioproduction is a crucial challenge.^54^ Another key challenge is the efficient fixation of atmospheric CO_2_.^54^ Furthermore, the optimal development of industrially viable products requires the selection of robust algal strains, reasonable capital and operating costs for cultivation, pretreatment and extraction and the desired process sustainability.^55^ In this regard, achieving an initial fractionation of algal biomass with minimally tunable parameters is the most important step toward the production of sustainable products.^56^

Significant progress has been made to date, including the validation of eight natural and synthetic CO_2_ fixation pathways, the development of synthetic energy capture techniques and the commercialization of several CO_2_-based plants.^50^ Despite recent progress, the fractionation of algal biomass into industrial products is still in its infancy. Issues around cell concentration, dewatering, affordability and efficient deconstruction of algae at scale remain extremely challenging. Considering future food/feed demand, shifting environmental conditions, and political instability in major oil-producing countries, more research into third-generation algae-based biorefineries is inevitable.

#### 2.1.4 Fourth-Generation Biorefineries

Fourth-generation biorefineries are derived from genetically modified microorganisms, such as microalgae and cyanobacteria, to enhance biofuel-producing organisms as feedstocks.^57,13^ The capacity of microorganisms to convert CO_2_ into fuel via photosynthesis is exploited and maximized by genetic modifications, thereby creating an artificial “carbon sink”.^58^ Several types of microalgae have been successfully produced by introducing genes into the nucleus, chloroplasts and mitochondria of cells.^59^

4G biorefineries require a minimum number of stages to convert energy, reducing processing needs. The main environmental benefits are CO_2_ assimilation, wastewater purification, and reduced greenhouse gas emissions. On the other hand, the environmental impact of the gene modification process requires further investigation.^60^

### 2.2 Lignocellulose Structure

Lignocellulosic biomass is a composite material synthesized by plant cells. It consists primarily of a mix of polymeric carbohydrates, cellulose and hemicellulose, and aromatic polymer–lignin intertwined together in a complex structure (Figure 4). It also contains smaller amounts of structural proteins, lipids and ashes. The arrangement of polymers in lignocellulosic biomass results in a highly recalcitrant structure, which must be decomposed to use it as feedstock for chemicals or energy.

**Figure 4:**
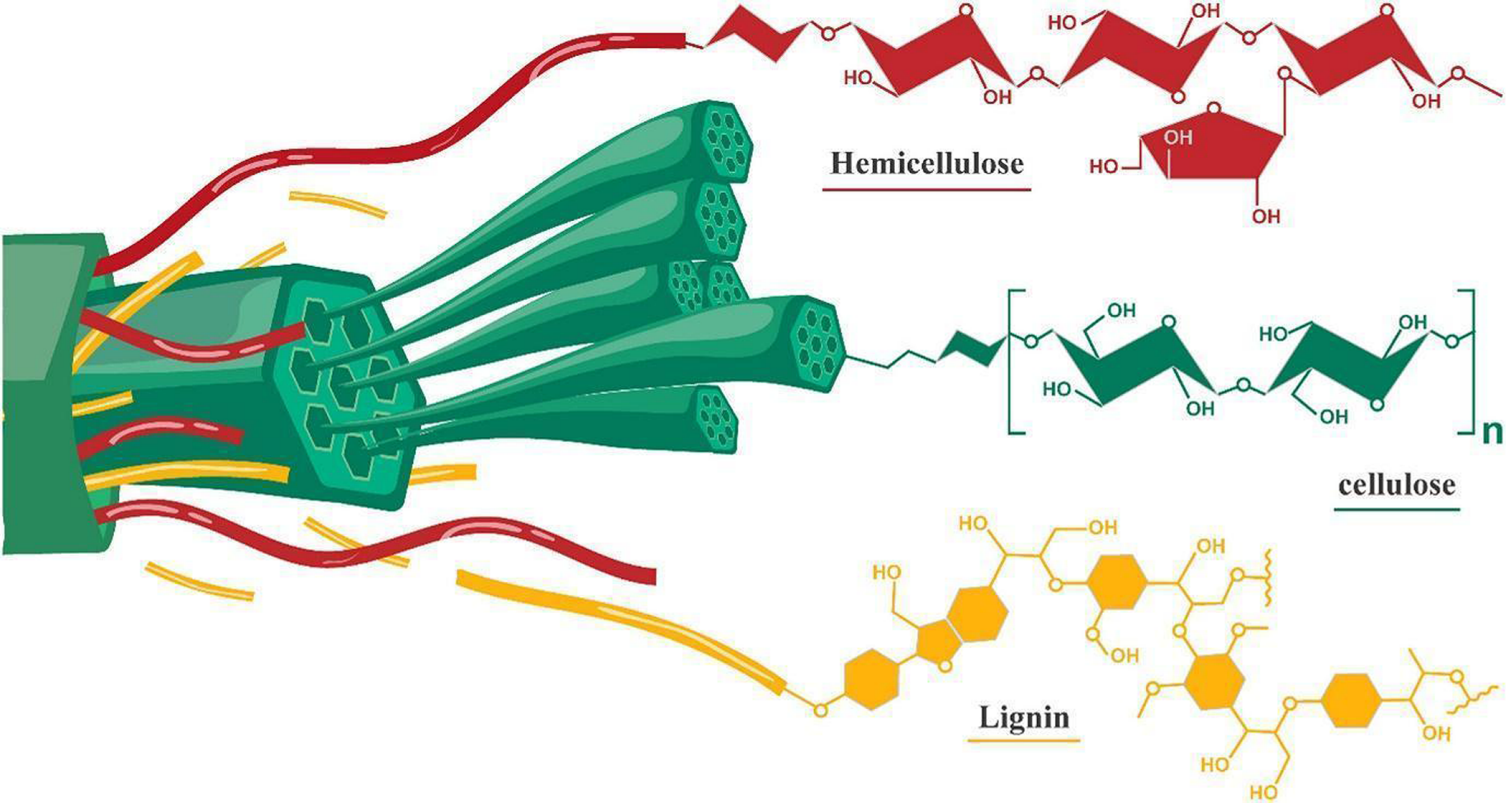
Schematic representation of the plant cell wall of a lignocellulosic biomass consisting of cellulose, lignin, and hemicellulose. Adapted with permission from ref 29. Copyright 2023 Wiley-VCH.

Its appearance and properties vary greatly depending on the type of plant (Figure 4).^61^ Cellulose (C6 sugar) accounts for 30–50% of total lignocellulosic dry matter, whereas hemicellulose (a mixture of C5 and C6 sugars) and lignin (provides structure rigidity) account for 20–40% and 15–25% of total feedstock dry matter, respectively.^29^ Cellulose is composed of glucopyranosyl monomeric units connected by 1,4-glycosidic bonds, resulting in a sheet-like structure that enables the packaging of multiple cellulose strands into crystalline fibrils. The main forces between the flat sheets of cellulose are Van der Waals (VdW) interactions. Hemicelluloses are bound noncovalently to the surface of cellulose fibrils to function as an amorphous matrix material. Hemicellulose is a branched polymer with greater functional and compositional diversity than cellulose. Functional compounds such as acetyl, methyl, cinnamic, and glucuronic acids are also in its structure.^62,63,8^ Lignin, an aromatic-containing polymer, produced when plant growth terminates, provides structural reinforcement and resilience to plant tissue.^39^ It is formed by the combination of three main monomeric units guaiacyl (G units), syringyl (S units) and *p*-hydroxyphenyl (H units). Lignin of different types of species (softwoods, hardwoods, and grasses) differ in the relative amounts of these 3 monomeric units. These disparities in composition have a substantial effect on delignification and biomass destruction.^64,65^

#### 2.2.1 Cellulose

Cellulose is the largest single component of lignocellulosic biomass whose composition on a dry weight basis is typically in the range of 35–50 wt%.^39^ It is a homopolysaccharide composed of glucopyranosyl monomers linked by β-1,4-glycosidic bonds. The configuration at the anomeric carbons results in a stretched chain shape, with hydrogen bonds connecting these chains to form flat sheets.^39^ Cellobiose is defined as the minimum conformational unit of cellulose, whereas glucose represents the fundamental unit of the homopolymer chains.^66^

In cellulose, glucose chains are bound by dispersion forces and hydrogen bonds in the crystalline structure, in approximately 40 glucan chains known as elementary fibrils.^67^ Such elementary fibrils, which essentially have a very long length and a width of approximately 250 Å, bundle into microfibrils.^68^ Microfibrils contain highly ordered, crystalline regions and less organized, amorphous regions. Both regions occur in characteristic proportions in different celluloses.^69^ The number of glucopyranose units per cellulose chain is defined as the degree of polymerization (DP), and it can vary depending on the source and extraction method.^70^ In nature, cellulose chains have a degree of polymerization of 10,000 glucopyranose units in wood cellulose and 15,000 in native cotton cellulose.^71^

The supramolecular organization of cellulose chains with numerous recognized polymorphs and amorphous domains in the solid state is determined by the complex hydrogen bonding network formed by the hydroxyl groups.^70^ Cellulose polymorphs are divided into four categories: cellulose I, which is found in native cellulose, and celluloses II, III and IV, which can be obtained irreversibly under specific circumstances and are thermodynamically more favorable. The most significant cellulose allomorph for materials science is cellulose II. It develops from cellulose I following mercerization (treatment with aqueous NaOH) or after native cellulose has been dissolved and then precipitated in an antisolvent, a procedure that is frequently referred to as regeneration.^70^ Cellulose dissolving ILs such as dialkyl imidazolium acetates can form the cellulose II structure by dissolution followed by regeneration, typically achieved by “crashing out” the cellulose through the addition of an antisolvent.

#### 2.2.2 Hemicellulose

Hemicelluloses are branched heteropolysaccharides with shorter chains than cellulose. Sugar moieties in hemicelluloses may be subdivided into pentoses, hexoses, hexuronic acids, and deoxyhexoses. Hydroxyl groups from β-d-xylopyranosyl units may be partially substituted by acetyl groups at O-2 or O-3.^72^ Small quantities of other sugars, such α-l-rhamnose and α-l-fucose, might also make up hemicelluloses, and acetyl groups can partially replace the hydroxyl groups in the sugar moieties.^72^ Only seaweeds, red and green algae, contain homopolymers of xylose, also known as homoxylans. The degree of acetylation varies according to the type of biomass and the amount of acetyl groups is between 1–6 wt% of total biomass on a dry basis.^73^ The main composition of the various hemicelluloses was depicted in Table 1.

**Table 1:** Main Types of Hemicelluloses Found in Diverse Feedstocks (Adapted from Girio *et al.* 2010)^74^

				units	
type of hemicellulose sugar	feedstock type	content	degree of polymerization	backbone	side chain
arabinogalactan (AG)	softwoods	up to 35 wt%	100–600	β-d-Gal*p*	β-d-Gal*p*
					α-l-Ara*f*
					β-l-Ara*p*
xyloglucan (XG)	hardwoods, grasses	2–25 wt%		β-d-Glc*p*	β-d-Xyl*p*
				β-d-Xyl*o*	β-d-Gal*p*
					α-l-Ara*f*
					α-l-Fuc*p*
					acetyl
galactoglucomannan (GGM)	softwoods	10–25 wt%	40–100	β-d-Man*p*	β-d-Gal*p*
				β-d-Glc*p*	acetyl
glucomannan (GM)	softwoods and hardwoods	2–5 wt%	40–70	β-d-Man*p*	
				β-d-Glc*p*	
glucuronoxylan (GX)	hardwoods	15–30 wt%	100–200	β-d-Xyl*p*	4-*O*-Me-α-d-Glc*p*A
					acetyl
arabinoglucuronoxylan (AGX)	grasses, cereals and softwoods	5–10 wt%	50–185	β-d-Xyl*p*	4-*O*-Me-α-d-Glc*p*Aβ-l-Ara*f*
arabinoxylans (AX)	cereals	0.15–30.0 wt%		α-l-Ara*f* Feruloy	α-l-Ara*f*
					4-*O*-Me-α-d-Glc*p*A
					acetyl
glucuronoarabinoxylans (GAX)	grasses and cereals	15–30 wt%		α-l-Ara*f*	
				4-*O*-Me-α-d-Glc*p*A	
				acetyl	
homoxylans (X)	algae			β-d-Xyl*p*	

Xylans and glucomannans are the two hemicelluloses that are most significant regarding abundance. The primary hemicellulose found in secondary cell walls, xylans make up 20–30% of the biomass found in herbaceous and woody plants. Additionally, xylans can make up as much as 50% of the tissues found in certain grasses and grains. The primary hemicellulosic elements in the secondary wall of softwoods are manan-type hemicelluloses, such as glucomannans and galactoglucomannans, while they are found in smaller quantities in hardwoods.

#### 2.2.3 Lignin

Lignin is a complex amorphous macromolecule made up of aromatic monomers. It is the most abundant natural source of aromatic structures. It evolved to give vascular plants rigidity and stiffness, allowing their tissue to resist the negative pressure created by the transport of water within the plants.^75^ Furthermore, it provides water impermeability and a physical and chemical barrier that protects plants from microbial and animal attacks.

The fact that native lignin cannot be recovered unaltered, together with the natural variability depending on species, genetic variability and growing conditions, means that its native structure has not been completely elucidated. However, a lot about its structure and biosynthesis is known. Lignin is a complex and amorphous biopolymer formed of phenylpropane based sub-units linked by ether and C–C bonds, and has a molecular weight of between 2,500 to 10,000 g/mol.^25^

Lignin is biosynthesized mainly from the radical polymerization of three hydroxycinnamyl alcohols (monolignols), namely guaiacyl, *p*-coumaryl and sinapyl alcohols, derived from the enzymatic conversion of l-phenylalanine.^76^ Their polymerization yields guaiacyl (G), *p*-hydroxyphenyl (H) and syringyl (S) units, respectively, which vary in the degree of methoxylation presented on the aromatic ring (Figure 5).^77,78^ Other less common subunits found in lignin are ferulates (which connect lignin with hemicellulose), coniferaldehyde, sinapaldehyde and 5-hydroxyconiferyl.^25^

**Figure 5:**
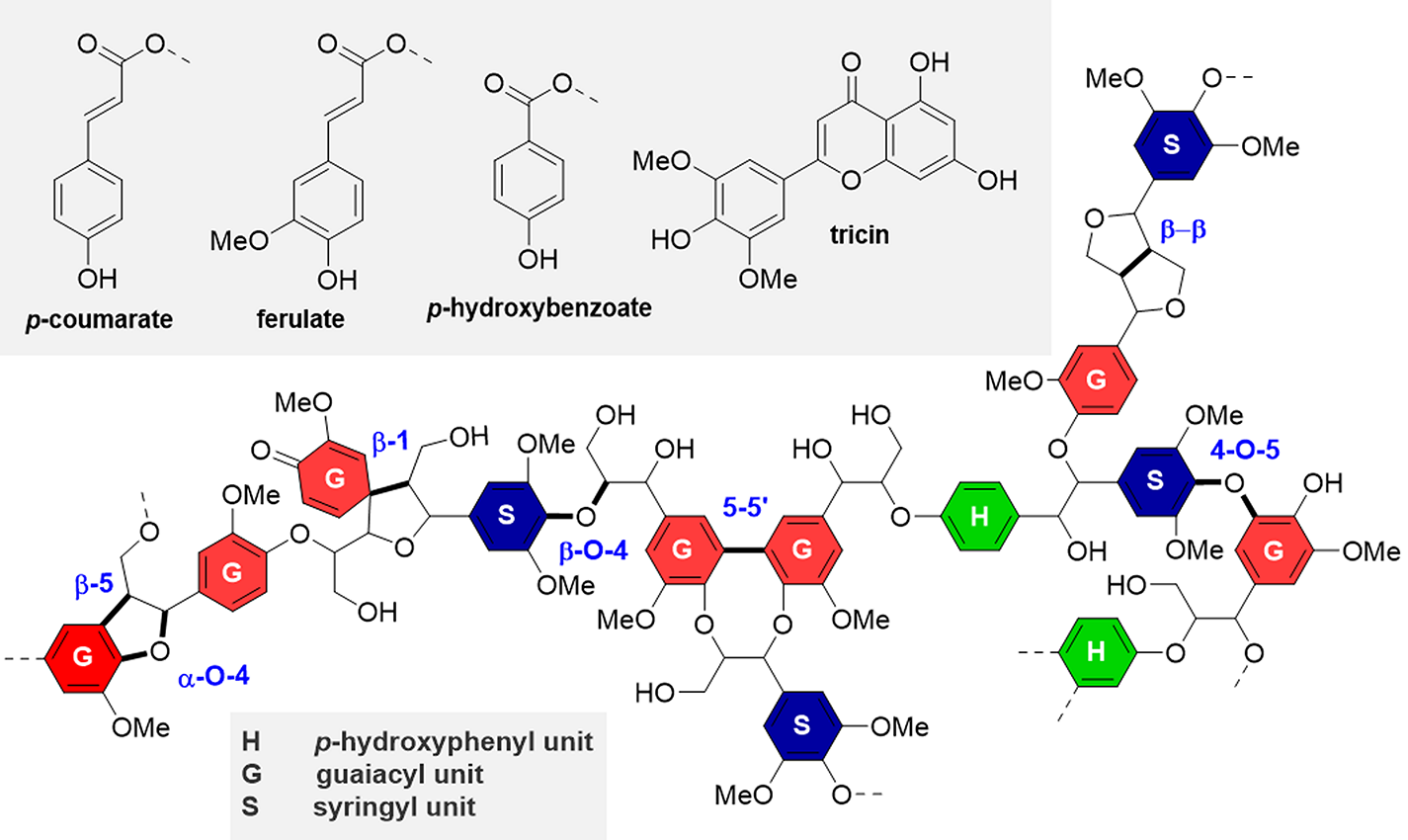
Representative structure of lignin to include various known units and interunit linkages in a range of grassy and woody lignocellulosic biomass. Adapted with permission from ref 78. Copyright 2024 Wiley-VCH.

The proportion of each of these units varies in different types of biomass. Lignocellulosic biomass employed for biorefining can be divided into 3 main groups according to species: softwoods, hardwood and grasses. Softwood is wood from gymnosperm trees (*e.g.*, pine or spruce) and has the highest lignin content (25–35%). Lignin from gymnosperms is very homogeneous, mostly made of coniferyl alcohol (G units, up to 95%) and lacking S units (syringyl alcohol) and is generally more branched than angiosperm lignin.^75,76^ Hardwoods are angiosperm trees and have lower lignin content than softwoods (15–30%). Hardwood lignin contains a mixture of about 60% of S-units and 40% of G-units.^75^ It contains abundance of β-*O*-aryl ether linkages, and it is crosslinked to polysaccharides by lignin carbohydrate linkages.^76^ Grasses contain less lignin than both soft- and hardwoods (9–20%), being a mixture of S, G, and H units (*p*-coumaryl alcohol, ranging from 20% to 50%).^69^ Grasses also contain high proportions of coumarates and ferulates.^76^ Furthermore, a different type of lignin, formed only by catechyl alcohol units (C-units), can be found in the seed coating of many plant species, including members of the *Cactaceae*, *Orchidaceae*, *Euphorbiaceae*, and *Cleomaceae* families.^79,80^ C units are nonmethylated; hence, C-lignin is a linear homopolymer. C-Lignin can coexist together with the more common G/S lignin, but it is not attached to it.^76^

Lignin monomers are linked by different types of ether bonds, such as β-O-4′ (β-aryl ether), 4-O-5′, α-O-4′, 4-O-5, and C–C bonds as β-5′ (phenylcoumaran), β–β′ (resinol), 5–5′, and β-1′ linkages. β-O-4′ is the most abundant linkage found in lignin, typically representing around half of the total amount of lignin interunit linkages, but ranges between 20% for some softwoods up to 80% for some hardwood species have been reported. Next in abundance are β–β′ and β-5′ linkages, which allow lignin chains to grow linearly forming long strains.^25,39,81^

Furthermore, lignin can be crosslinked to carbohydrates (mainly hemicellulose, but also to some extent to cellulose) via covalent bonds, forming what is known as lignin–carbohydrate complexes (LCCs). It has been suggested that the level of cross linking via LCCs is directly related to the cell wall rigidity and resistance to enzymatic attack of biomass. In softwoods, all lignin fragments are linked to carbohydrates, up to 50% being linked to cellulose. In hardwoods between 47% to 66% of lignin fragments are linked to carbohydrates, with up to 17% linked to cellulose.^82^ Eight different types of lignin–carbohydrate bonds have been found: benzyl ether, benzyl ester, glycosidic or phenyl glycosidic, hemiacetal or acetal linkages, and ferulate or di-ferulate esters (Figure 6).^82^ Efficient biomass pretreatment processes must be able to cleave and/or hydrolyze these linkages.^39^ LCCs of grasses contain ferulic acid (FA) bonded to hemicellulose (feruloylated arabinoxylan) by ester bonds, LCCs involving glucan and xylan have been reported as well. LCCs of grasses show prevalence of phenyl glycosidic bonds.^82,83^ LCCs of hardwood involve xylan and glucan moieties, and phenyl-glycosidic linkages are predominant. For softwoods, benzyl ether linkages are prevalent and different LCC structures have been proposed, a branched structure involving glucomannan and a linear structure involving xylan.^82^

**Figure 6:**
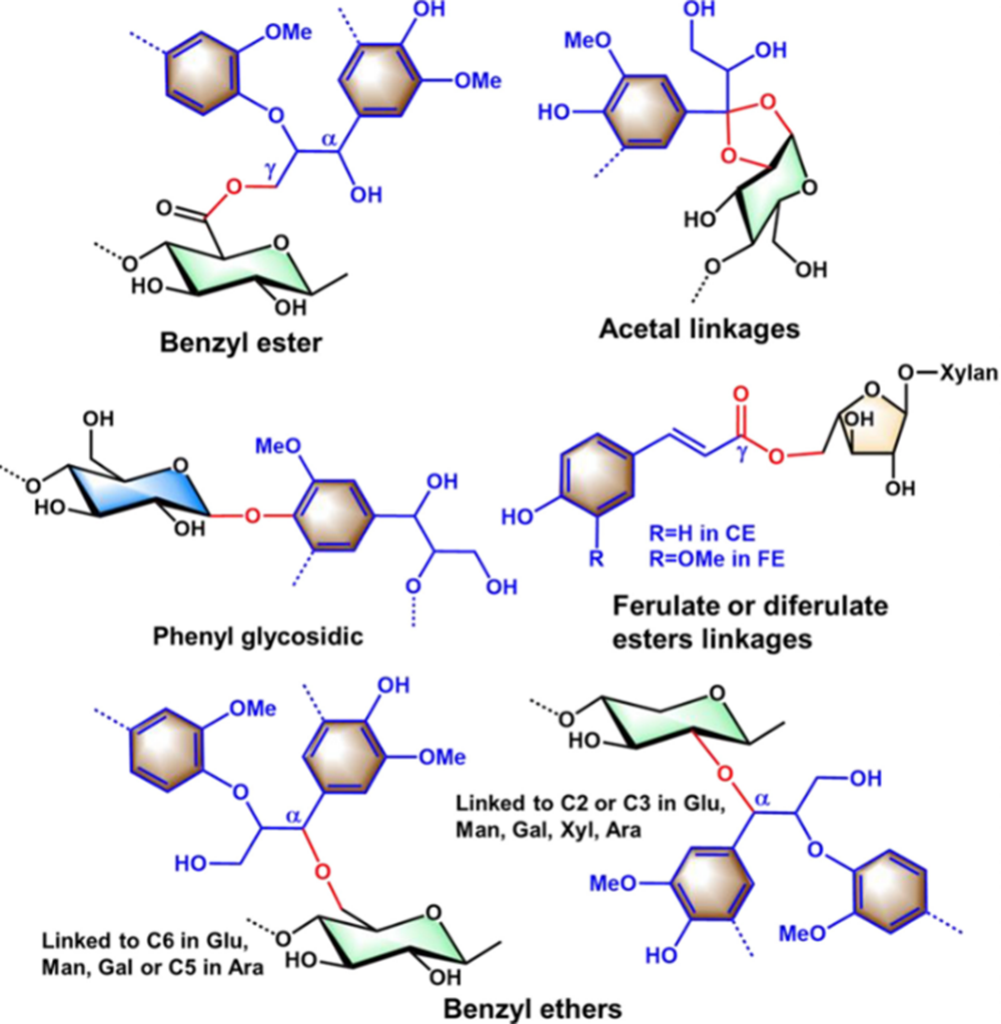
Some of the main types of LCC bonds found in lignocellulosic biomass. Adapted with permission from ref 84. Copyright 2023 Elsevier Ltd.

It should be noted that lignin biosynthesis, and consequently its structure, is very adaptable and admits different variations on the aromatic ring and the side chain. Gene editing has been used to produce genetically modified strains with tailored lignin content and characteristics (*e.g.,* reduced recalcitrance). This allows for milder pretreatment conditions for cellulose fractionation and further conversion or the development of specific lignin products.^85^ In this regard, it has been proposed that engineering of the lignin in the pith tissue of certain species, where is more easily accessible, is a potential avenue to produce lignins for targeted applications.^86^

#### 2.2.4 Lipids and Extractives

Although cellulose, hemicellulose, and lignin are the main structural components of lignocellulosic biomass, different amounts of other compounds such as proteins, lipids and inorganic material can be also found. These families of compounds are referred to as extractives. Extractives are usually secondary metabolites produced by plants not for structural purposes, but to perform different biological activities. These include plant defense against pathogens and herbivores^87^ and adaptations to environmental conditions.^88^ The term extractive covers thousands of different molecules, which are classified in families depending on their molecule structure and biosynthetic pathway. The main families of compounds are alkaloids, phenolic compounds, or polyphenols and terpenoids. Other classes that have been reported include saponins, lactones, ginsenosides, tocopherols, sterols and carotenoids. Extractives can be recovered from different plant parts, most commonly from leaves, roots and barks, for their use in a wide range of applications. For example, extracts rich in the terpenoids carnosic acid and carnosol produced from rosemary leaves are currently authorized as antioxidants in foods and cosmetics;^89^ extracts rich in the polyphenols hydroxytyrosol produced from olive tree leaves are used in foods^90^ and as bleaching in cosmetic products;^91^ extracts rich on triterpenic saponins from the wood and/or bark of *Quillaja saponaria* tree are currently approved as foaming agents and emulsifiers in foods and dietary supplements;^92^ phytosterol derived from tall oil, a by-product from the Kraft process, are used as nutraceuticals to reduce cholesterol;^93^ and tropane alkaloids are recovered from roots of several plants to be used in pharmaceutical products because of their anticholinergic activity.^94,95^

### 2.3 Lignocellulosic Biomass Utilization Strategies

The words pretreatment and fractionation have been inadvertently used as interchangeable terms in the literature. However, they present different meanings in the field of biomass utilization. Pretreatment methods change the structure and composition of biomass, making it more suitable for subsequent processes. Fractionation methods, on the other hand, aim to separate the structural components of biomass for separate valorization. A pretreatment method that can be considered good may not efficiently fractionate lignocellulosic biomass and vice versa. There is not a single pretreatment or fractionation method that gives the best results for all cases due to differences in the structure of different biomasses and different end-use requirements. The selection of the most suitable method for a given process depends on several factors, including productive factors such as yields and recovery and economic and technological aspects that must be considered for implementation at an industrial scale. Further valorization of the streams must be also considered, *i.e.*, processes that break down lignin could be useful to produce ethanol but may not be suitable for lignin recovery. Therefore, several pretreatment and fractionation methods have been developed and studied using different biomass sources. Biomass pretreatment and fractionation processes can be classified into physical, chemical, physicochemical, thermochemical and biochemical methods, depending on how the changes in the biomass are achieved. Although, many times they are combined to improve their overall efficiency.

Physical processes, including milling/crushing,^96–100^ extrusion,^101–104^ microwave,^105–110^ and ultrasound,^110–113^ modify the lignocellulosic biomass structure without the need of chemical or biochemical reactions. They are, in general, more environmentally friendly and prevent chemical degradation of biomass, avoiding loss of sugars and other compounds. On the other hand, they suffer from high energy consumption and insufficient biomass deconstruction. To overcome these disadvantages, they are usually performed followed by chemical, physicochemical, or biological methods.

Chemical processes are among the most employed. These methods use acids,^114–120^ alkalis,^116,120–125^ oxidants^126–130^ and organic solvents^120,131–136^ to perform biomass degradation, usually breaking the linkages between the different biopolymers. They lead to extensive chemical changes in the biomass and are usually more efficient than physical and biological methods, but these chemical changes can also produce undesirable by-products. Other disadvantages of chemical methods are the large amount of chemicals consumed and the need to remove them from the biomass.^114,137^ Most of the chemical methods employ reagents and pH-dependent conditions that are noncompatible with downstream bioconversion processes involving enzymes and microbial strains. To enable downstream processes, separation of these reagents is mostly achieved by (water)-washing. However, this adds on to the process complexity, operational cost, and carbon loss.^45,138,139^ This has led to the foundation of biocompatible deconstruction technologies to overcome the above-mentioned shortcomings and explore the benefits of process integration.^140–142^ Combinations between chemical and physical methods have been largely studied to achieve better results, indeed physicochemical have been evaluated for biomass deconstruction to take advantage of physical and chemical changes at the same time. Physicochemical methods used to pretreat biomass include liquid hot water,^143–145^ steam explosion (SE),^143,146–150^ ammonia fiber explosion (AFEX),^150–152^ and CO_2_ explosion.^153–155^ These methods exert physical and chemical changes over the biomass to reach the biomass deconstruction, particularly in SE and AFEX, in which the swift release of pressure promotes the biomass deconstruction while organic acids derived from the biomass in SE or the ammonia in AFEX promote chemical changes in the biomass. Sometimes chemicals such as acid in SE or hydrogen peroxide in AFEX are added to further improve the chemical deconstruction.^156^

Biochemical methods are considered eco-friendly and safe as they do not require chemicals or high temperature/pressure conditions. However, they are usually expensive and slow in comparison to other methods due to the high cost of enzymes and the slow hydrolysis rates.^114^ Microbial pretreatments can be divided into two different processes: biodelignification, which aims to remove lignin from the biomass, and saccharification which aims to hydrolyze cellulose and hemicellulose in sugars.^114,157^ Enzymes and microorganisms have been evaluated to perform biomass delignification, while saccharification is normally performed using enzymes.^158^

When not physical, the pretreatment and fractionation strategies can be summarized in Figure 7. Basically, they aim to remove the lignin from the lignocellulosic biomass, therefore they are delignifying. Hemicelluloses are more sensitive to changes in process parameters such as temperature, time and pH and they can be greatly removed (strategy 1) or they can be preserved (or partially preserved as it will be seen later on the IL categories) in the treated material (strategy 2). If fractionation is efficient, lignin and hemicellulose products can be valorized into different chemicals and/or materials. Otherwise, the pretreated material can be biochemically converted into biofuels such as bio-ethanol, bio-butanol, or bio-succinic acid.

**Figure 7:**
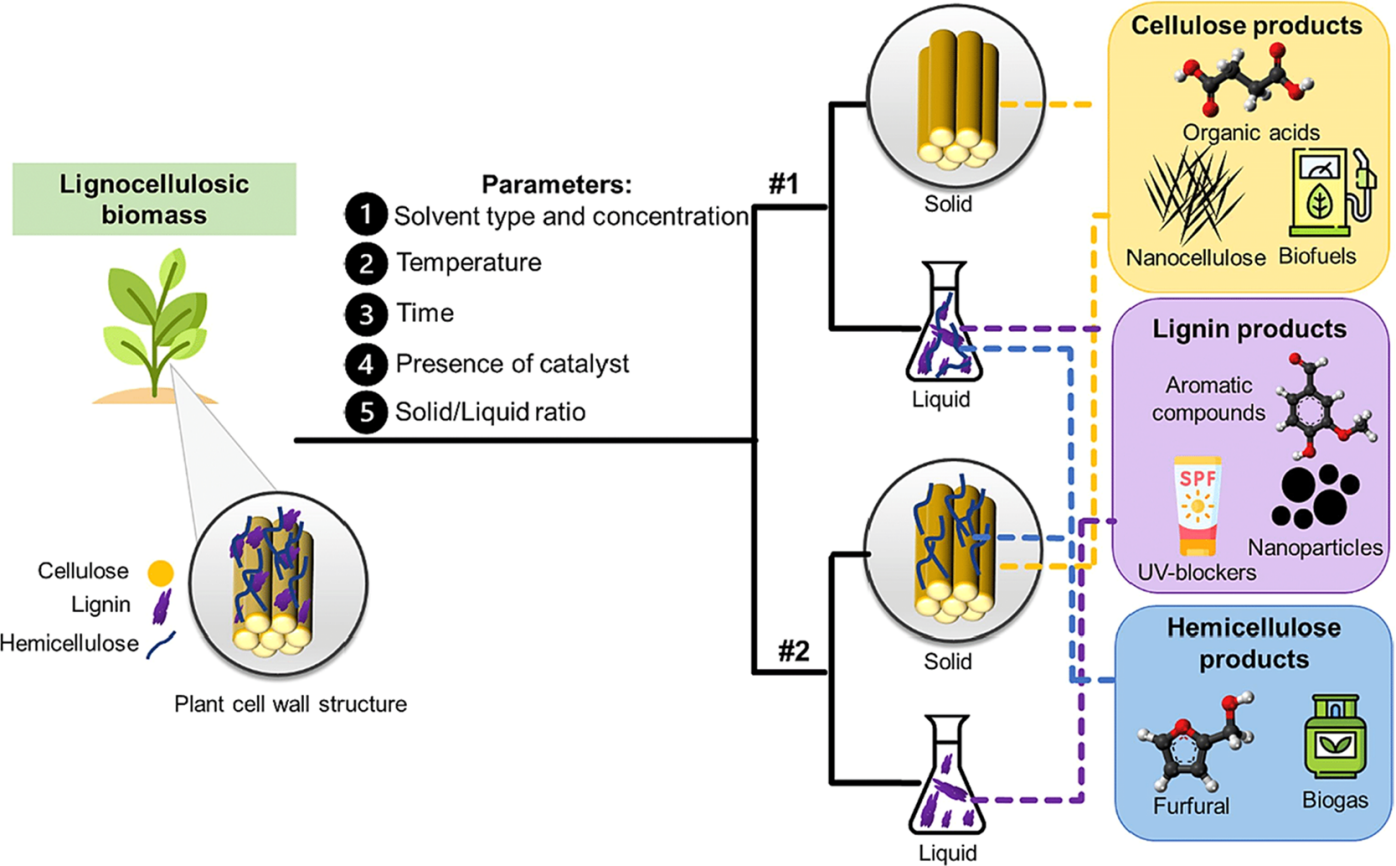
Pretreatment and fractionation strategies for the utilization of lignocellulosic biomass. Adapted with permission from ref 159. Copyright 2023 Elsevier Ltd.

### 2.4 ILs and DESs as a Solution for Biorefining

Among the chemical biomass processing methods, the use of ILs and DESs for the pretreatment and fractionation of lignocellulosic biomass has shown to be very effective, being one of the more promising areas for their application at the industrial level. The finding of the dissolution of cellulose in ILs in the early 2000s became the stepping stone of the use of ILs (and DESs) in biorefinery.^17,160^ From the decade of the 2010s onwards, the biomass pretreatment field has been focusing on: (1) expanding the feedstock portfolio to different types of biomasses,^161–163^ (2) optimization of pretreatment parameters for each feedstock,^141,164^ (3) understanding the relationship between IL/DES structure and pretreatment performance,^165–172^ (4) looking into the fate of the hemicellulose and lignin fraction upon pretreatment,^173–177^ (5) evaluating the reuse and recycling of the IL/DES,^174,178–181^ (6) studying the scale-up of the pretreatment^182–186^ and (7) evaluating the feasibility of IL/DES pretreatment by techno-economic analysis.^43,45,187–192,185,193,194^ From all of the literature on the topic, it can be concluded that ILs/DESs are efficient pretreatment agents and, depending on the cation and anion, they can be used to create an economically viable process.

The main types of anions and cations employed on the pretreatment of lignocellulosic biomass are shown in a timeline in Figure 8. The main cations range from nitrogen-containing heteroaromatic compounds such as imidazolium and pyridinium to aliphatic and cyclic ammonium (Figure 8). More exotic phosphonium cations have been also employed, but to a lesser extent.^195^ Compared to the variation of anions, cations are less diverse because anions play a bigger role in the interaction between ILs/DESs and lignocellulosic biomass.^40^ The most employed anions are halides, carboxylates, hydrogen sulphate, and amino acid (AA) derived anions. It is important to note that the work mentioned in Figure 8 is related to biomass pretreatment and not to the solubilization of lignocellulosic fractions. Therefore, they employ enzymatic hydrolysis as a means of evaluating the pretreatment.

**Figure 8:**
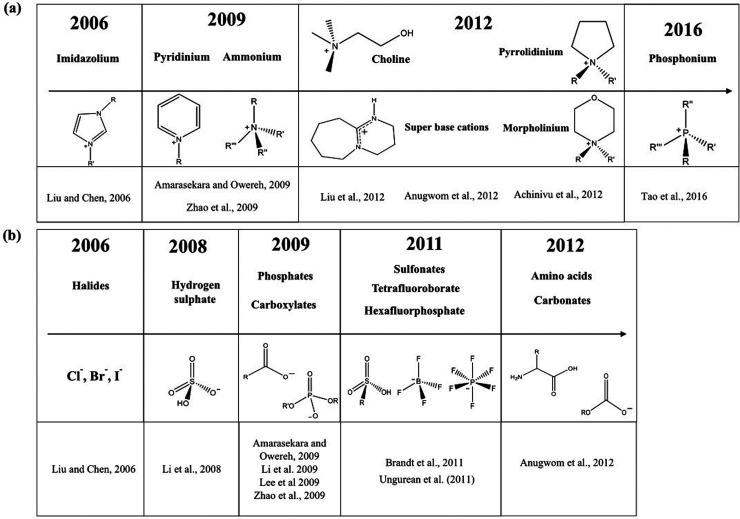
Timeline of the main types of (a) cations and (b) anions introduced as pretreatment agents of lignocellulosic biomass. These studies performed pretreatment and enzymatic saccharification as a means of evaluation pretreatment performance. R and R′ denote either −H or −alkyl substituents. References from this figure are: Liu and Chen, 2006;^196^ Amarasekara and Owereh, 2009;^197^ Zhao *et al*., 2009;^198^ Liu *et al*., 2012;^199^ Anugwom *et al*., 2012;^200^ Achinivu *et al*., 2012;^201^ Tao *et al*., 2016;^195^ Li *et al*., 2009;^202^ Lee *et al*., 2009;^203^ Brandt *et al*., 2011;^204^ Ungurean *et al*., 2011.^205^

In the following sections we will be reviewing and assessing all the relevant aspects of lignocellulosic biomass pretreatment and deconstruction with ILs and DESs, from the properties of these novel types of solvents and their mechanisms of interaction with lignocellulose, to the implications and challenges of their integration in commercial scale biorefineries (>25 MMtons/year production capacity).

#### 2.4.1 Properties, Toxicity, Degradability, and Biocompatibility of ILs and DESs

The uses and applications of ILs and DESs have evolved in the last decade linked to the development in the understanding of their properties. In general, ILs and DESs as a class of materials have some widely accepted generic physicochemical characteristics (*e.g.*, high viscosities and densities, ultra-low vapor pressure at ambient conditions, etc.). Nevertheless, considering the ability to tune the structural and functional properties of ILs and DESs as a function of their constituents, a wide range of ILs/DESs are available (the total number of possible ILs/DESs has been estimated to be >10^20^). Hence, it is very difficult to generalize and summarize all of their properties.^18,19,206–212^ The evolution of the understanding of the physicochemical properties of ILs and DESs, thanks to the improvement of characterization and quantification methods, is putting under question some of the previously generalized properties, including electrochemical window, long term thermal stability, polarity, and volatility.^213,214,211^

When designing ILs for targeted applications, traditionally, the choice of anion was used as the constituent with the largest impact on the values of the key physicochemical properties of the final ILs, while the choice of cation charged group and side chains was used to fine tune such properties.^20^ Another factor that has a significant effect on the design of ILs and DESs are their hydrophobic/hydrophilic properties, which are responsible for their solvation performance. In this review article, a detailed description will not be provided. Rather, only essential observations are given below:

- Melting points of ILs and/or DESs can be unpredictable in nature as they can undergo supercooling and may contain different amounts of impurities.
- The ultra-low vapor pressure and resistance to flammability of ILs at ambient conditions, make the handling of ILs/DESs safer than that of common molecular solvents.
- ILs and DESs are denser than molecular organic solvents, with typical density values ranging from 1 to 1.6 g·cm^‑3^.^215,216^

- The viscosity of a solvent plays a big role in catalytic, mixing, and pumping applications. The viscosity of the majority of ILs/DESs is one to three orders of magnitude higher than conventional solvents.^216,217^

The unique physical and chemical properties of ILs and DESs can be exploited for addressing many of the drawbacks associated with the use of organic solvents in industrial processes, such as flammability and volatility. In a common petrochemical plant, the use of an organic solvent requires the addition of flares and catalytic burners to ensure that emissions are below the threshold established by legislation. On the other hand, the majority of ILs and DESs are generally highly resistant to flammability and thanks to their negligible vapor pressure that do not require the addition of any extra unit operations to manage vapors safely in terms of personnel exposure. The negligible vapor pressure also implies that the chance of dispersing ILs and DESs in the environment is much lower compared to organic solvents and facilitates their recovery and recycling. An efficient separation of the products is needed to guarantee high recovery and minimize the washing steps.

The idea that ILs (or/and DESs) could replace conventional solvents has created a lot of interest in the academic and industrial communities. This substitution is enforced by the EU REACH (Registration, Evaluation, Authorization and Restriction of Chemicals) regulations that are designed to improve safety and protect the environment and their restricted substances list, which phases out hazardous chemicals and provides further opportunities for safer, ILs and DESs-based processes.^218–220^ However, due to the relative higher cost and concerns about the potential toxicity of some ILs/DESs, their use in large scale applications needs to be justified.^43,221^ The implementation of ILs/DESs at industrial scale is feasible in processes where the benefits of employing an IL/DES overcome the associated costs of its use (as in some electrochemical applications) or where the cost is not a key factor (pharmaceutical and medical applications); those where they are used in a scale that is relatively small in the overall process (as catalysts, in coatings and thin films, as lubricants, surfactants, or additives, etc.); and those that can be performed with low-cost ILs (such as certain protic ILs). Currently, several examples of ILs/DESs applications established at the industrial scale, either already commercialized or at pilot plant stage, can be found and have been summarized in literature.^222,223^

##### 2.4.1.1 Toxicity

Solvent toxicity represents a danger for humans and the environment since potential accidents due to release can arise. Legislation regulating the handling of chemical compounds usually sets limits on the volume of flammable and toxic chemicals in a chemical plant including storage and process flow streams. This, together with experience gained from past incidents, has been pushing the chemical industry to keep raising the safety standards, aiming to create effective health and safety measures. In this regard, toxicity and biodegradability have been major challenges for ILs/DESs as the first generation of ILs were not biodegradable and the assessments of their toxicities were not well established.^224^ However, taking these considerations into account, in recent years a lot of effort has been made in exploring alternative, less toxic and more environmentally friendly ILs. Thanks to this, the perceptions of high toxicity and low biodegradability are being diminished. In fact, a significant range of bio-derived and biocompatible ILs have been established in the literature.^225–228^ It is important to note that each IL or DES needs to meet certain minimum requirements to qualify as a biocompatible ILs as far as toxicity is concerned. Nevertheless, the large-scale production of sustainable, environmentally friendly, and cost competitive ILs remains challenging. Finally, it should be highlighted that very often the organic solvents that the ILs/DESs are replacing have higher toxicity and environmental impacts, so their substitution is still advantageous even when the replacement is not completely innocuous.

It is well known that the head group of the cation in an IL plays a significant role in toxicity,^229–231^ with longer side chains having a greater impact on living organisms.^232^ It has been demonstrated that the inclusion of an ester group in the alkyl chains of an IL increases the susceptibility of such side chain to be biodegraded.^233^ However, the assessment of the (bio)­degradability of the IL needs to take into account the bioavailability and the fate of the resulting fragments and, hence, all the other present functional groups.

The effect of the anions on the toxicity of ILs seem to be more difficult to predict but, as a broad generalization, seem to be related with their hydrophobicity/lipophilicity. Other effects, such as their stability towards hydrolysis also play a role in some cases (*e.g.,* anions that can release HF show higher cytotoxicity than that predicted according to their lipophilicity).^234,235^

#### 2.4.2 Integration of ILs and DESs with Industry

Although the substitution of the solvents employed in the chemical industry for safer and more environmentally friendly alternatives can lead to significant improvements in the safety levels of industrial processes, including health and environmental benefits, it can also lead to a drastic impact on the commercial viability of the process. A change in the solvent employed in a process can have a deep impact in the way such processes are performed: from operational parameters to the selection of materials for most plant components. Moreover, the choice of one specific solvent can favor one step by penalizing other steps. For example, in the Difasol process the application of ILs in a large-scale process is still hindered by the capital cost of the equipment.^236,237^ For new solvents such as ILs or DESs to be economically sustainable, they should be supplied at competitive prices and industry-scale volumes. Economic considerations can therefore help shape technical aspects of solvent design. Nevertheless, as mentioned in the previous section, several applications of ILs/DESs at scale have been already established, highlighting the potential of ILs/DESs to help shape the future of the chemical industry.^222,223,238^

##### 2.4.2.1 Methods for Solvent Selection

Jin *et al.* proposed a useful 10-step methodology to evaluate the choice of solvent for any given process (Figure 9).^239^ The selection of potential viable replacements for a solvent that needs to be abandoned is based on the comparison of the Kamlet–Taft (K−T) parameters as a tool to compare solvent properties. Solvents with similar values for these parameters are considered the most promising candidates to substitute the problematic solvent. Further studies to evaluate the viability of the potential candidates have to be performed. These include the evaluation of their physical properties, synthetic routes, toxicology and final implementation through Life Cycle Assessment (LCA).

**Figure 9:**
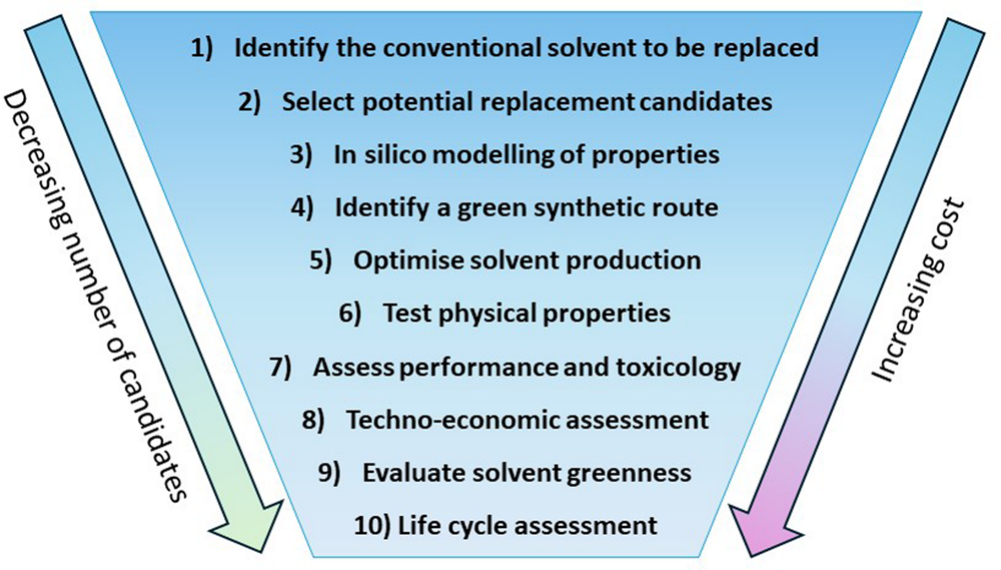
Method for the selection of a solvent for a defined application proposed by Jin *et al.* ^239^ Adapted with permission from ref 239. Copyright 2017 Royal Society of Chemistry.

A simple criterion to quickly assess the most promising IL/DES for a given process should focus on the role that the IL/DES will play in the process and its potential performance. After the identification of suitable candidates, production cost assessments should be made. The number of synthetic steps required for solvent synthesis can offer guidance for both potential cost and sustainability. As a rough estimation, the cost of a solvent could be assumed to double with each step away from a precursor.^240^ In the case of ILs, those employing aprotic (fully alkylated) cations normally require an extra step (ion metathesis) and will therefore be much more expensive than their protic counterparts.

##### 2.4.2.2 Industrial Processes Based on the Use of ILs/DESs

ILs have been already successfully established in at least 57 industrial processes at scale, starting in 1996 with their application in the isomerization of 3,4-epoxybut-1-one to 2,5-dihydrofuran, an intermediate for the production of tetrahydrofuran, by the Eastman Chemical Company.^241^ In 2002, BASF demonstrated the large-scale application and recycling of ILs in their BASIL (biphasic acid scavenging with ILs) process.^241^ By 2019, 57 processes based on ILs had been either already commercialized or were in the pilot development stage.^242^ A timeline of the introduction of industrial processes based on ILs is shown in Figure 10.

**Figure 10:**
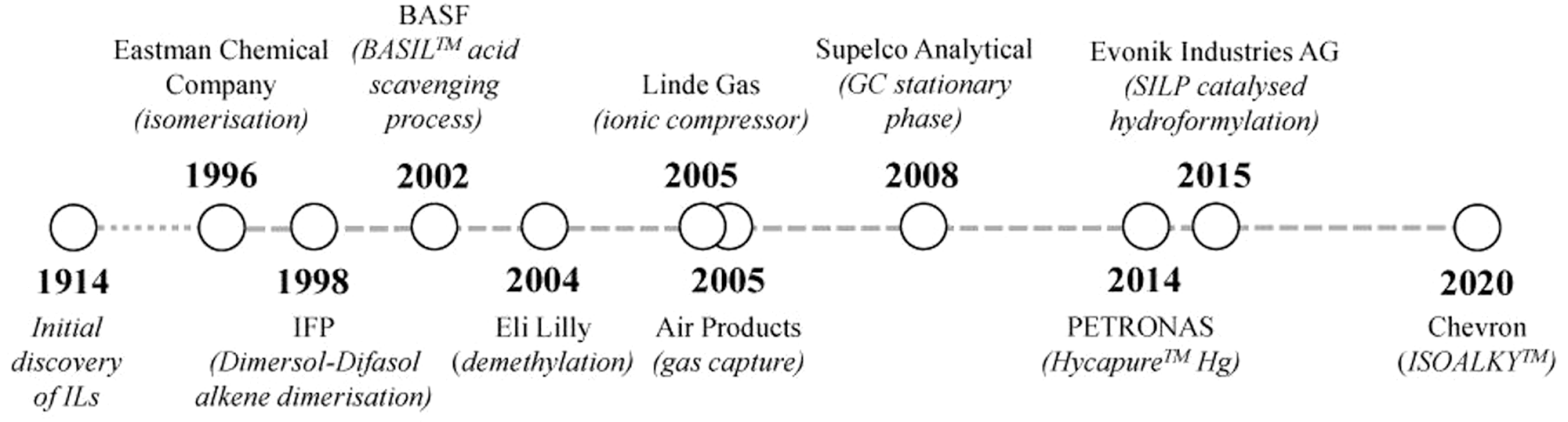
Timeline of industrial processes based on the use of ILs. From Greer *et al*. 2020.^241^ Adapted with permission from ref 241. Copyright 2020 MDPI under CC BY 4.0 (https://creativecommons.org/licenses/by/4.0/).

Of special interest for this review is the integration of ILs in industrial pulping processes. In this regard, there are a few applications that are already at the pilot stage of development. These include the Ioncell process, an alternative to Lyocell processes developed at the Aalto University in Finland.^243^ It uses superbase ILs, such as 1,5-diazabicyclo[4.3.0]­non-5-ene acetate ([DBNH]­[C_1_CO_2_]), to dissolve cellulose-pulp for dry-jet wet spinning production of high performance cellulose fibers with application in fabrication of textiles. Metsä Spring is also developing a fiber-production technology based on cellulose pulp dissolution with ILs.^244^ Lixea, a start-up company from Imperial College London, is commercializing biomass fractionation with ILs via their registered Dendronic process. Their pilot plant, located in Bäckhammar, Sweden, started operating in May 2022.^245^ Erg Bio Inc. is a startup located in Dublin, CA that is commercializing the ASPIRE IL technology for biomass conversion into biofuels and bioproducts. In contrary to the IL-based development, there are limited examples of DES-based startup. Bioeutectics, an Argentinian biotech, develops and provides natural and high-performance solvents for sustainable industrial products and processes.

##### 2.4.2.3 Industrial Production of ILs and DESs

The implementation of ILs/DESs in industry demands their production at the relevant scale. This can be a challenging task, considering the high costs of some ILs/DESs and some of their properties, such as high viscosities, which can make them hard to handle. The availability of certain starting materials can also limit the scaling up of the production of some ILs/DESs.

Many companies now commercialize ILs/DESs as part of their portfolio, including Merck, Acros Organics, BASF, Evonik, etc., and there are a few companies that are primarily IL producers (with either a few or no DES product line), *e.g.*, Iolitec, Proionic, Scionix and Solvionic. More than 500 ILs can be purchased from those companies. Moreover, at least 10 ILs are available in quantities over 1 tonne.^241^ Proionic has developed the carbonate-based IL synthesis process (CBILS), a low impact technology that allows to produce a variety of ILs in multi-tonne scale using a continuous flow process.^246^ Iolitec are also capable of producing ILs in the tonne scale.^247^ As a consequence of the increasing production capabilities, the prices of several ILs are being reduced.^241,248^ DESs, on the other hand, owing to their limited industrial applications and simple preparation (requiring only heating and stirring or two or more components) have not been a top rated product portfolio for most of the chemical companies.

### 2.5 Types and Classification of ILs and DESs for Biorefining

For ILs, in terms of their synthetic mechanism and proton availability, two main categories can be considered, protic (PILs) and aprotic ILs (AILs).^163^ This distinction is important since both classes of ILs work differently when applied for lignocellulosic biomass pretreatment. The main structural difference between PILs and AILs is that while AILs have fully alkylated cations, PILs have an acidic proton, a proton that can dissociate in aqueous medium and decrease the medium’s pH.^249^ Some AILs with halide and alkyl sulfate anions can dissolve cellulose and establish chemical pathways to valorize it to further chemical compounds. On the other hand, Brønsted acidic PILs can fractionate biomass by dissolving lignin, obtaining a high purity cellulose which can be used for further processing.^39^

Furthermore, other categories can be considered in terms of their origin −biobased ILs, BILs, which are ILs that can be obtained from renewable sources, chemical behavior and alkaline ILs, a category that includes ILs that can be either AILs of PILs, with anions with high β, such as acetates or derived from AAs, or DESs, a class of solvents that share many characteristics with ILs and even show some overlap between both classes of solvents, as already discussed.

Regarding DESs, a novel class of innovative solvents that have acquired significant scientific and technological significance, are typically considered as cost-effective alternatives to conventional organic solvents and ILs.^23^ Synthesized by combining Brønsted or Lewis acids and bases in precise molar ratios, DESs have significantly lower eutectic points than the optimal liquid mixture.^23,250^ The resulting liquid always has a lower freezing point than the components used to synthesize DESs.^251^ The majority of commonly reported DESs are HBAs such as quaternary ammonium, phosphonium salts with amides, carboxylic acids, or other HBDs, such as urea, thiourea, glycerol, and oxalic acids.^252^ Based on the chemical nature and type of these HBDs and HBAs, DESs are classified into several types (types I, II, III, IV, and V) as discussed below.

#### 2.5.1 Aprotic ILs

AILs are the most common type of ILs found in the literature. They have fully alkylated cations and their synthesis usually involves at least an amine or phosphine quaternization reaction followed by an ion metathesis stage. Other synthetic stages might be needed depending on the target structure. These preparations are lengthier and more complicated than those of PILs and they usually employ more expensive starting materials compared with PILs. The need of ion metathesis for the preparation of many AILs implies low atom economies and the production of salt-containing waste streams, necessitating costly waste disposal. Often, the preparation of AILs present problems associated with halide contamination in the final product, requiring appropriate analysis and purification or specialized synthetic procedures.^253^

When applied to biomass processing, alkaline AILs follow a different mechanism than neutral AILs, as will be explained with detail in section 2.6. It has been reported that, under certain conditions, some AILs with basic anions and imidazolium or phosphonium cations, such as 1-ethyl-3-methylimidazolium acetate ([C_2_C_1_im]­[C_1_CO_2_]) and tetradecyltrihexylphosphonium acetate ([C_14_C_6_C_6_C_6_P]­[C_1_CO_2_]), can act as PILs, displaying acid–base equilibrium behavior, via carbenes or ylide formation, respectively.^254^

#### 2.5.2 Protic ILs

The synthesis of protic ionic liquids (PILs) is a simple neutralization reaction via transfer of a proton (H^+^) between an acid and a Brønsted base. Often, PIL synthesis can be performed solvent-free.^254,255^ However, if strong Brønsted acids such as sulfuric or nitric are employed the reaction is exothermic. To minimize hazards, dropwise addition of aqueous diluted acid to the Brønsted base or appropriate cooling should be employed. PILs possess remarkably different physicochemical properties compared to conventional AILs, including in some cases an ability to distill due to high volatility, although this can be related to a low-ionicity IL and the fact that distillation takes place via the neutral acid and base.^256,257^ By having an exchangeable proton, PILs exhibit Brønsted acidity, which allows them to be used as solvents for a number of acid catalyzed reactions including Diels–Alder, Beckmann rearrangement and condensation reactions such as aldol or Baeyer.^257^ Since most PILs are synthesized from simple acids and bases, they usually present short life cycle trees and tend to be more environmentally friendly due to reduction in by-product generation, solvent losses, energy use and carbon dioxide generation. This also makes PILs manufacture cheaper (up to 40 times) when compared to the most common AILs studied in the literature for applications in biomass pretreatment.^258,168,174^

In either case, for both AILs and PILs, the choice of the precursor is reflected in the final cost of the IL. For example, longer alkyl chain ILs and functionalization or the presence of heteroatoms such as phosphorus or fluorine increase production costs. Techno-economic analysis of the bulk-scale synthesis of ILs showed that while aqueous mixtures of alkylimidazolium hydrogen sulfate ILs can be produced in the price range of $2.96–5.88 kg^–1^; replacing the cation precursor for simpler and cheaper trialkylamines can reduce the cost to bulk scale for as little as $0.78 kg^–1^,^259^ which is comparable to the cost of common organic solvents like acetone and toluene.^259,168^ For reference, estimates of bulk prices of frequently investigated AILs are in the range of $40–81 kg^–1^,^174^ or 5–20 times of the price of organic solvents. This finding addressed one of the main concerns raised about using ILs in large quantities: their alleged high cost. Furthermore, in the long term, it is expected that synthesis of biobased PILs from the lignocellulosic biomass itself,^141^ in a strategy similar to Socha and co-workers, will be possible further reducing the environmental impacts associated with the production of ILs.^228,260^

One potential issue of certain PILs is that, if the difference in p*K*_a_ between the two-precursor species is not large enough, the proton transfer might not be complete, leaving behind some proportion of the molecular species.^249,257^ Additionally, the drying step can push the reaction equilibrium towards the molecular precursors, favoring the removal of one of them from the mixture, creating a nonstoichiometric mixture that is concentrated in the less volatile precursor species. Comprehensive discussions on PILs proton transfer, ionicity, and their impact on the physicochemical properties of the ILs can be found elsewhere.^257,261–263^

#### 2.5.3 Biobased ILs and DESs

Biobased ionic liquids (BILs) are defined as the ILs obtained from natural products, their analogues, or bioactive molecules.^264^ Typically, this class of ILs is considered as green, renewable, biocompatible and/or biodegradable. Similarly, natural DESs (or NADESs) are also biobased DESs prepared from components that could be obtained from natural sources such as [Ch] and carbohydrates, among others.^265,260,266^ Such class of solvents are interesting due to their origin and have potential to sustain a closed-loop biorefinery for continuous production of biochemicals and bio-based materials.^228,260^

Although several components, including AAs, sugars, aromatic aldehydes, alkaloids, terpenes and fatty acids, have been employed as precursors to the components of BILs or NADES, [Ch]- and AA-based ILs/DESs have dominated the literature mostly owing to their simple synthetic protocol (Figure 11).^267,264^ It is worth remarking that, despite being labeled as bio-based, many of the DESs and ILs reported in literature are being obtained from [Ch] and glycine, which are produced from petrochemicals. These could, indeed, be obtained from biological sources, but at significantly higher prices (up to six-fold). This is reflected in the prices of AAs produced from fermentation (lysine) or protein hydrolysis, which are considerably higher. In a similar fashion, other amines usually produced from fossil sources, and therefore not labeled as “bio”, could be also obtained from renewable and low-carbon footprint feedstocks and processes, but again still at higher prices than their petro-based counterparts.^268,269^

**Figure 11:**
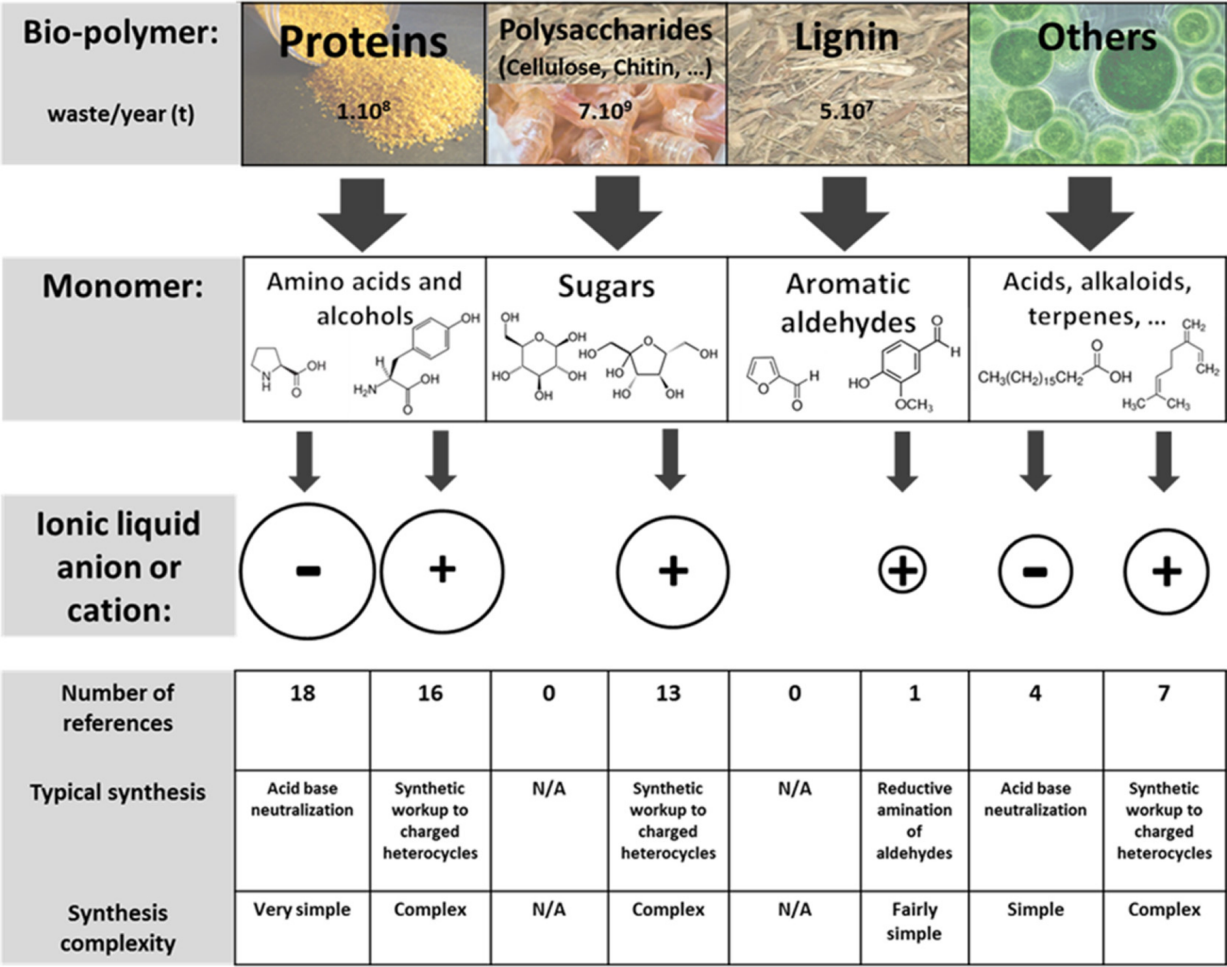
Precursors to bio-based ILs. Adapted with permission from ref 264. Copyright 2016 American Chemical Society.

Since the first synthetic report of AA-based [Ch] ILs by Ohno *et al.*,^270^ several newer synthetic protocols have been developed and studied for their physicochemical, toxicity and biodegradability properties.^271^ Liu *et al.* first explored the application of these [Ch]­[AA] ILs in the biomass processing demonstrating about 30 times higher lignin solubility compared to polysaccharides.^199^ Thermal stability and viscosity are important parameters for successful application of these ILs in biorefinery. Most of these ILs were found to be thermally stable in the temperature range of 150–200 °C, while the viscosity depends on the size and complexity of the anions. The toxicity studies on the [Ch]­[AA] ILs have classified these as practically non-toxic to most bacterial cultures paving the path to integrate the bioconversion processes without separation of ILs unlike traditional imidazolium-based ILs.^233,272^

#### 2.5.4 Deep Eutectic Solvents (DESs)

The majority of commonly reported DESs contain HBAs such as quaternary ammonium and phosphonium salts with HBDs such as amides, carboxylic and oxalic acids, urea, thiourea, glycerol, etc.^252^ By their own nature, DESs are non-stoichiometric and can be defined by the general formula: *Cat* ^+^ *X* ^–^ *zY* where Cat^+^ represents ammonium, sulfonium, or phosphonium cation, while *X* is a Lewis base, usually a halide anion, and *z* is the total number of *Y* molecules which interact with anion.^273^

##### 2.5.4.1 Type I DES

This category of eutectic mixtures is comprised of quaternary ammonium, phosphonium, sulfonium salts and non-hydrated metal halides, including FeCl_3_, ZnCl_2_, LaCl_3_, and SnCl_2_.^274,275^ However, the high cost and limited availability of anhydrous metal halides appropriate for DESs synthesis limit their application.

##### 2.5.4.2 Type II DES

This type of DESs are characterized by the utilization of hydrated metal halides and quaternary ammonium salts. Due to the low cost of hydrated metal halides and their insensitivity to moisture, type II DESs compounds are used in numerous industrial applications.^276^

##### 2.5.4.3 Type III DES

This class of DESs is the most researched and well-liked in the scientific community, as it is derived from inexpensive, non-toxic, and biodegradable starting compounds. In particular, quaternary ammonium salts are combined with HBDs such as alcohols, amides, and carboxylic acids to create these eutectic compositions. [Ch]Cl is a frequently employed quaternary ammonium salt (HBA) for type III DESs that is derived from biomass and classified as a vitamin source.^277,274,278^

##### 2.5.4.4 Type IV DES

Abbott *et al*. reported the development of type IV DESs by combining transition metal halides with appropriate HBDs such as ethylene glycol, acetamide, and 1,6-hexanediol. This category of DESs is still in its infancy in terms of research.^274,279^

##### 2.5.4.5 Type V DES

Non-ionic compounds are also utilized in the preparation of eutectic mixtures with low melting points for DES of type V. Recently, Coutinho *et al*. found that the thymol–menthol system exhibits type V non-ionic deep eutectic mixtures with extraordinarily strong interactions.^280^

Figure 12 summarizes the principal characteristics of the solid–liquid phase diagrams for these binary DESs. Note that the eutectic composition is a single value corresponding to the minimal melting temperature in the phase diagram, as shown in Figure 12.^281^ The formation of these low-melting eutectic composites is driven by hydrogen bonding interactions between the components. It is hypothesized that the interaction of the HBD with the quaternary salt reduces the anion–cation electrostatic force responsible for the formation of hydrogen bonds, thereby significantly lowering the freezing point of the mixture.^278^ DESs share the advantageous solvent properties of ILs, such as minimal volatility, a broad liquid range and biocompatibility.^282,283^ Nonetheless, several hypotheses, such as charge delocalization, cluster formation, and a decrease in lattice energies due to the use of asymmetrical cations in DESs, have been proposed to explain the lowering of the eutectic solutions' melting point.

**Figure 12:**
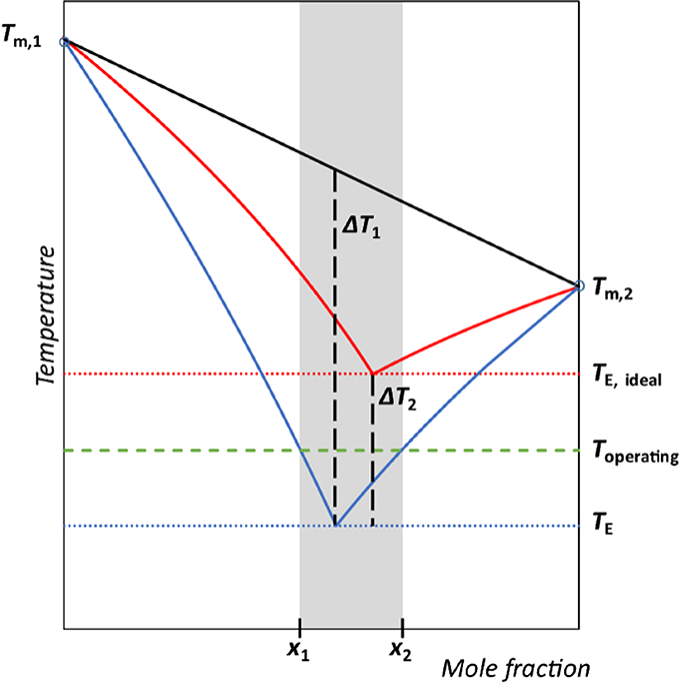
Schematic representation of the comparison of the SLE of a simple ideal eutectic mixture (red line) and a deep eutectic mixture (blue line).^24^ Adapted with permission from ref 24. Copyright 2018 Springer Nature.

Figure 13 depicts the HBAs and HBDs used to prepare a variety of popular DESs. As it shows, it is possible to obtain neoteric DESs with specific physical and chemical properties by modulating the HBAs and HBDs. Due to the wide availability of HBAs and HBDs, a variety of structural modifications are conceivable; therefore, as with ILs, DESs are also known as “designer solvents”.^284–286^ Choi *et al*. reported the production of highly viscous natural deep eutectic solvents (NADES) by combining [Ch]Cl with various HBDs such as AAs, organic acids, and carbohydrates.^287^ In a similar fashion, Silva *et al*. prepared and termed as “*therapeutic deep eutectic solvents (THEDES)*” by combining an active pharmaceutical ingredient (API) with a previously prepared eutectic mixture.^288^ While the majority of the innovated DESs till date are hydrophilic and water-miscible, Osch *et al.* reported the formation of water-immiscible hydrophobic DESs.^289–291^

**Figure 13:**
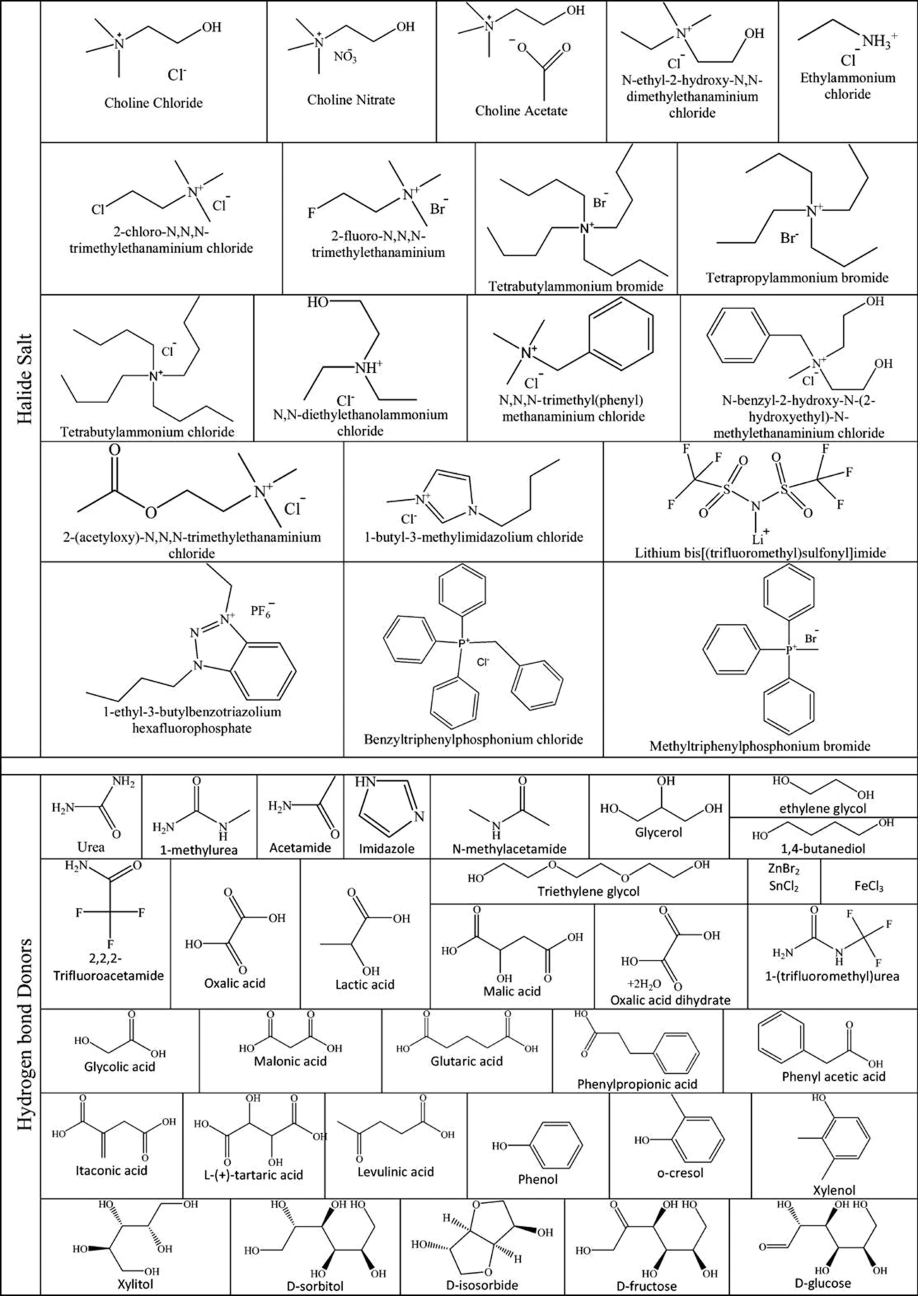
The most prevalent structures of hydrogen-bond donors and halide compounds employed in the synthesis of DESs. Adapted with permission from ref 281. 2015 American Chemical Society.

Due to the versatility and uniqueness of DESs, they have acquired significant scientific and technological significance as alternatives to conventional organic solvents and ILs and have been studied in applications including biomass processing and lignin chemistry.^292,25^ The potential for designing suitable DESs with high applicability for the dissolution of various biomass is significant, as the properties of DESs may be readily adjusted by modifying the HBDs and HBAs.^293,294^ As such, effective utilization of DESs for the treatment of biomass can be possible only with a thorough understanding of their physicochemical properties.^252^ DESs still have the challenges associated with ILs for their use at scale, most notably costs associated with efficient recovery and recycling and biocompatibility.^25^

### 2.6 Mechanisms of Biomass Pretreatment with the Different Types of ILs and DESs

Biomass delignification with four different types of IL-based solvent systems, that follow different mechanisms, has been very successful: (I) neutral AILs, usually based on chloride or alkylsufate anions, (II) alkaline ILs (which can be either aprotic or protic ILs and include most BILs),^295,296^ typically based on acetate or AA derived anions, and (III) Brønsted acidic PILs, in particular those with hydrogen sulfate and chloride anions; and (IV) DESs.^25,297,298^

The type of IL and the water content employed during the pretreatment determine which mechanism comes into play.^298^ Each of these types of systems offer different advantages, disadvantages, and working mechanisms and will be discussed in this review. Neutral AILs are capable of dissolving the lignocellulosic biomass, but usually they are not very selective towards specific macrocomponent like lignin or cellulose. Alkaline ILs (either protic or aprotic) are capable of solubilizing lignin but, depending on the severity, have limited ability to solubilize hemicelluloses. Brønsted acidic PILs that solubilize most of the hemicelluloses and lignin, producing a cellulose-rich pulp (Figure 14). These categories are mainly based on the type of anion that constitutes the IL, as it determines the main interaction with lignocellulosic biomass.^39^ DESs typically dissolve both lignin and hemicellulose, in a similar fashion to Brønsted acidic PILs.

**Figure 14:**
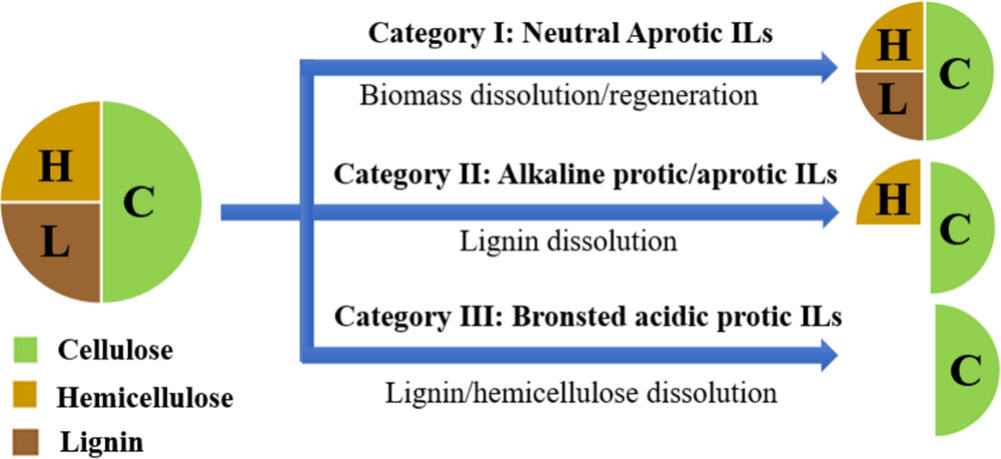
The three main categories of ILs used in the pretreatment of lignocellulosic biomass.

Since most IL-based pretreatment technologies typically have low water content during biomass pretreatment, acidity rules from the pH scale are not applicable. Therefore, other types of acidity scales have been developed, such as the Hammett acidity function which uses a range of closely related UV–vis probes to generate the Hammett acidity, *H*_0_.^299,300^

Abouelela *et al.* (2023) introduced the *H*_0_ values of the IL butyl-*N*,*N*-dimethylammonium hydrogensulfate ([C_4_C_1_C_1_N]­[HSO_4_]) to the pretreatment severity factor, *R*_0_, commonly used for hydrothermal and dilute acid pretreatments, on the pretreatment of pine and observed that the new *R* ^•^_0_ presented better correlation between severity and pretreatment parameters such as mass loss, glucan and lignin recovery.^301^ This will be discussed in further detail in section 2.8.3.

Other methods to measure the acidity of ILs include the one developed by Fărcaşiu to estimate the acidity of organic solvents by using the ^13^C NMR spectrum of mesityl oxide as a probe,^302^ that was later adapted by Grasvik *et al*. for [HSO_4_]-based ILs.^300^ They were able to correlate the Δδ of mesityl oxide with the *H*_0_ of the ILs within the range of −1 < *H*_0_ < −9, outside such range, large changes in H_0_ correspond to small changes in Δδ. In depth studies about the structure, proton dissociation, and acidity of protic ILs based on hydrogen sulfate anions and their interactions with water have been recently published.^303,304^

It should be noted that the delignification ability of a solvent is not necessarily reflected in the recovered lignin yields. Delignification quantifies the proportion of lignin in the biomass that gets dissolved into the pretreatment solvent, independently of how much of it is recovered from the liquor. Lignin yield quantifies the amount of lignin precipitated from the pretreatment and washing liquor upon the addition of an antisolvent.^305^ Antisolvents are added to the pretreatment liquor to reduce the solvation power of the IL, allowing precipitation of the lignin from the IL (and also of the cellulose if the process follows the dissolution mechanism). Appropriate selection of antisolvent is key to maximize lignin recovery and minimize further reactivity of lignin.^164^

#### 2.6.1 Pretreatment with Neutral AILs

Neutral or slightly acidic AILs containing anions with low Brønsted basicity (such as halides, alkylsulfates, etc) but relatively high β can dissolve lignocellulosic biomass, but they usually are not very selective towards specific macrocomponent like lignin or cellulose (Figure 14). Typical aprotic cations include dialkylimidazolium-derived structures like [C_2_C_1_im] or [C_4_C_1_im].^306–308^ The most used and classic example is [C_4_C_1_im]­[Cl], one of the first aprotic ILs employed in cellulose and biomass dissolution studies.^17^ Several studies were dedicated to understand the effect of this IL on lignocellulosic biomasses such as corn stover,^309^ palm oil fronds,^310^ rice straw and husk^311,312^ and sunn hemp fibre.^313^ Additionally, a few review papers have been dedicated to explore the use of such ILs such as the works by Cao *et al.* (2017), Halder *et al.* (2019) and Colussi *et al.* (2023).^314,315,258^

The main working mechanism for most pretreatments with AILs is based on cellulose swelling and solubilization (at least partially) in the IL, which takes place together with that of lignin and hemicellulose.^81^ Fort *et al.* investigated the solubilization of wood chips in 1-butyl-3-methylimidazolium chloride ([C_4_C_1_im]­[Cl]) by using nuclear magnetic resonance NMR analysis. They discovered that the weight ratio of dissolved cellulosic material to lignin was largely constant at 2:1 and consistent with the original composition of the biomass. This finding proves that cellulose and lignin dissolve simultaneously and without obvious selectivity.^316^ Dissolving the whole biomass opens the opportunity for catalytic depolymerization of the cellulose, or for the preparation of composites upon biomass recovery, thanks to the capability of ILs to induce thermoreversible crosslinking between biomass components. Also, since the ILs can act as plasticizers and aid in extrusion processes, to apply blending and wet spinning.^317^

Some molecular dynamics studies attempted to glimpse into the mechanistic role of imidazolium-based ILs in cellulose and lignin dissolution. Li *et al.* (2015) analyzed the entire dissolution process of cellulose bunches (a group of four and seven glucan chains) and found out about the synergistic influence of cations and anions.^318^ They showed that, in the beginning, anions insert into the cellulose strands to form H-bonds with hydroxyl groups whereas cations attach to the side face of the cellulose bunch. Then, because of their potent electrostatic interaction with the incoming anions, cations begin to intercalate into cellulose chains. 1-Ethyl-3-methylimidazolium chloride ([C_2_C_1_im]­[Cl]) and [C_4_C_1_im]­[Cl] dissolved cellulose slower than [C_2_C_1_im]­[C_1_CO_2_] because the H-bonds created by [Cl]^−^ could not interact with the cellulose chains as efficiently as [C_1_CO_2_]^−^. Regarding the interaction of the ILs with lignin, Zubeltzu *et al.* (2020) employed Ab Initio Molecular Dynamics (AIMD) simulations, found out that the cation is crucial to the lignin's solvation process because it stabilizes the aromatic ring with the alkyl chain and the hydroxyl oxygen with the cation ring.^319^ Finding out how the IL interacts with the hydroxyl group is particularly important for lignin's depolymerization process, which frequently starts with dehydration reactions.

It should be highlighted that with this pretreatment mechanism cellulose digestibility is achieved by the disruption of the cellulose crystallinity after recovery from the solvent.^25^ This means that cellulose regeneration is accompanied by shift of crystal structure from I to II which represents a lower degree of crystallinity.^312,320^ It is important to highlight that even with imidazolium-based ILs, a decrease in crystallinity can only take place under anhydrous conditions.

A drawback of these processes is the need of an antisolvent to recover the cellulose, which usually leads to the co-precipitation of lignin, hindering the separate valorization of both streams since the presence of lignin poses significant barriers to enzyme and microbial hydrolysis and fermentation of the pretreated cellulosic materials.^321^ Therefore, this type of ILs is not much employed in the biochemical route. Additionally, at room temperature (r.t.), the majority of chloride-based ILs are solid or a gooey paste; they present viscosity values that are tens or hundreds of times greater than those of water and organic solvents.^258^ The difficulty handling and high melting point of these ILs are often seen as technical drawbacks to the solvents’ potential to be recycled. Additionally, the presence of water often makes cellulose solubilization problematic; an extremely viscous mixture is produced when cellulose precipitates in the presence of trace amounts of water.

#### 2.6.2 Pretreatment with Alkaline (Protic or Aprotic) ILs

Alkaline ILs (both protic or aprotic) are capable of solubilizing lignin and, to a lower extent, partly solubilize hemicelluloses depending on the severity of the pretreatment (*i.e.*, higher temperature and longer times will favor hemicellulose dissolution).^322^ These ILs contain anions from weak acids such as acetic, phosphoric and AAs and usually present high β value. Acetic acid is the most common carboxylic acid employed, due to the high hydrogen basicity of the acetate anion (β > 0.80)^39,314^ and to its wide availability and green synthesis (it could be produced by oxidation of bio-ethanol, though at present the majority source is petrochemical).

The most common types of cations found in this category and used for biomass pretreatment are [C_2_C_1_im], [C_4_C_1_im], [Ch] and [C_2_C_1_N].^141,165,178,204^ ^,^ ^221,313,323–327^ Nevertheless, imidazolium-based ILs are being progressively replaced by ammonium-based acetate ILs (*e.g.*, AIL [Ch] acetate, [Ch]­[C_1_CO_2_] and PIL *N*-ethylmethylammonium acetate, [C_2_C_1_N]­[C_1_CO_2_]) due to the high cost and ecotoxicity associated with the imidazolium cation.^328^ The thermal stability of these ILs is another concerning issue. However, these types of pretreatments are still effective for the production of sugar-based chemicals, since they rely on the disruption of the hydrogen-bonding network and can achieve high cellulose saccharification yields with only partial lignin removal.^81,161^

As already mentioned, hemicellulose fractionation can be poor with alkaline ILs, as it could been seen from works by Gschwend *et al.* 2020 with [C_2_C_1_im]­[C_1_CO_2_] on spruce, Velmurugan *et al.* (2023) on corncob and Zhang *et al.* (2013).^322,308,329^ However, the poor solubility of hemicellulose in these ILs might be seen as an advantage for certain applications, since the recovery of hemicellulosic sugars from the IL liquor is difficult due to the high polarity of ILs, such as those that use the one-pot configuration.^330–335^ Hence, lignocellulosic biomass fractionation with alkaline ILs produce carbohydrate-rich pulps containing both cellulose and hemicellulose sugars (Figure 14). These pulps can be hydrolyzed by cellulases and hemicellulases into a hexose/pentose rich syrup that can then be metabolized by a microorganism able to consume both C5 and C6 sugars. Due to their high efficiency, this category of ILs is frequently employed to produce cellulose-rich pulps that can be bioconverted into platform chemicals.

##### 2.6.2.1 Pretreatments with Biobased ILs

Most BILs are based on AA anions. Therefore, pretreatment of biomass with them falls in this category of alkaline ILs. BILs offer a cheaper, renewable and biodegradable alternative for biomass processing compared with imidazolium based ILs (*e.g.,* ILs with a [Ch] cation and an AA-derived anion such as [Ch] lysinate, [Ch]­[Lys]). Their biggest advantage is their compatibility with enzymes and yeasts used for the hydrolysis of the biopolymers recovered after pretreatment.^81^ However, IL recovery procedures can be complicated as BILs often present thermal stability issues or the need of acidification and neutralization stages for its recycling.^81,336^

#### 2.6.3 Pretreatments with Acidic PILs (ionoSolv Fractionation)

Among the different types of ILs, acidic PILs are interesting solvents due to their low synthesis cost and higher environmental friendliness. Acidic PILs are synthesized using strong Brønsted acids such as sulfuric, nitric, hydrochloric, or methanesulfonic acid. These acids can either be in the form of anion such as in 1-ethyl-3-methylimidazolium hydrogensulfate ([C_2_C_1_im]­[HSO_4_]), triethylammonium hydrogensulfate ([C_2_C_2_C_2_N]­[HSO_4_]), or pyridinium nitrate ([Py]­[NO_3_]) or can be present as acid co-catalysts in the IL system such as [C_4_C_1_im]Cl + HCl or [C_4_C_1_im]Cl + HNO_3_, but the main characteristic of this category is the presence of free protons, H^+^, in the medium.

They were firstly employed for biomass treatment at the start of the 2010 decade, aiming to replace conventional AILs, which are much more expensive and require a higher number of synthesis steps.^337^ It was soon found that pretreatments with most PILs dissolve only the lignin and hemicellulose fractions, leaving a cellulose rich pulp behind with preserved crystallinity (Figure 14).^204^ This has the advantage of allowing for separate valorization of lignin and cellulose streams after isolation of these fractions from the IL liquor. Due to its similarities with organosolv pretreatments for the dissolution of lignin and hemicellulose with organic solvents, the biomass treatment process with PILs was then called the ionoSolv process, a term proposed and popularized by Brandt *et al*.^39^ The substitution of molecular solvents by PILs entails several key advantages in terms of safety, environmental friendliness and cost.^298^ It also has some important advantages over the dissolution process, which aims to solubilize the entire biomass structure. As mentioned before, PILs are much cheaper than the conventional aprotic ones, which greatly improves the economic competitiveness of the process.^338^ Hallett and co-workers have demonstrated that ammonium-based [HSO_4_] ILs are cheap, robust and can be reused for several pretreatment cycles to yield high-purity cellulose pulps and lignin as precipitate. Several types of feedstocks have been probed including grasses such as *Miscanthus* ^337^ and sugarcane bagasse,^339^ softwoods such as pine^340^ and hardwoods such as willow.^341^ In addition, the number of ILs that are capable of dissolving lignin is greater than that of those dissolving cellulose, giving rise to a higher number of possibilities for biomass fractionation. PILs are also generally more thermally stable than aprotic ones, which is crucial for recycling purposes. In this review, and in order to make things easier, this term would be applied to all the relevant works done with PILs in biomass treatment and not only those where the authors stated they were performing an ionoSolv treatment.

For ionoSolv pretreatments delignification and cellulose yields are closely correlated, and values of *R* ^2^ in the ranges of 0.92–0.94 have been reported.^342^ IonoSolv lignin yields are typically higher than those achieved with Organosolv fractionations even at milder conditions.^174,343^ However, ionoSolv lignins often show partial recondensation not observed for organosolv lignins, particularly at high pretreatment severity, which can influence their further processability. The delignification of the biomass is highly influenced by the nucleophilic acidic nature of the anion of the IL, acting both as catalyst and solvent during the ionoSolv process.^344^ The precipitation of the different fractions of the biomass, in particular lignin, can be carried out by a medium of different antisolvents, such as acetone, ethanol, dimethyl sulfoxide or water, which are capable of keeping certain fractions in dissolution so that they can be recovered later.^345^

A potential drawback of ionoSolv fractionation is the solubilization of hemicellulose in the IL, which makes the recovery of the hemicellulose sugars non-trivial. Previous studies have shown that significant amounts of xylose and arabinose extracted from *Miscanthus*, a grassy biomass, with [C_2_C_2_C_2_N]­[HSO_4_] can be found in the IL solution as monomers or furfural.^168^ Furthermore, by analyzing HSQC NMR spectra it has been shown that hemicellulose sugars do not precipitate with the ionoSolv lignin, other than during the very early stages of pretreatment.^174^ There are options to tackle this issue. The first one is to perform a two-stage fractionation to prior remove the hemicelluloses with pretreaments such as dilute acid or hydrothermal, strategies followed by Qureshi *et al.* and Ovejero-Perez *et al.* ^346,347^ However, a two-stage pretreatment process implies increasing operational costs and waste generation. The second option relies on continuously extracting furfural from the IL during pretreatment. This will ensure the hemicelluloses are being broken down into pentose monomers and then dehydrated into furfural, shifting the equilibrium towards the anhydrosugars. A schematic example of the ionoSolv process can be found in Figure 15.

**Figure 15:**
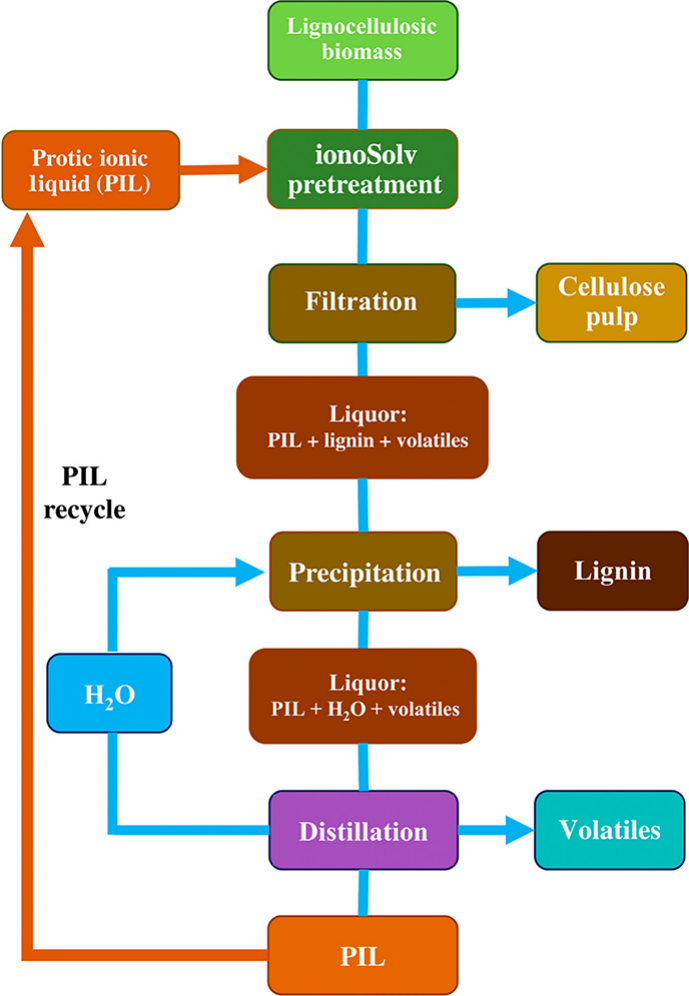
Scheme of the ionoSolv process for biomass treatment. Adapted with permission from ref 298. Copyright 2022 Elsevier Ltd.

This process has been successfully employed with different biomass types towards completing two main objectives: fractionating the lignocellulosic material into its main components and improving the yield of subsequent processing steps. Figure 16 summarizes some relevant works that achieved something unique in terms of biomass fractionation with PILs.

**Figure 16:**
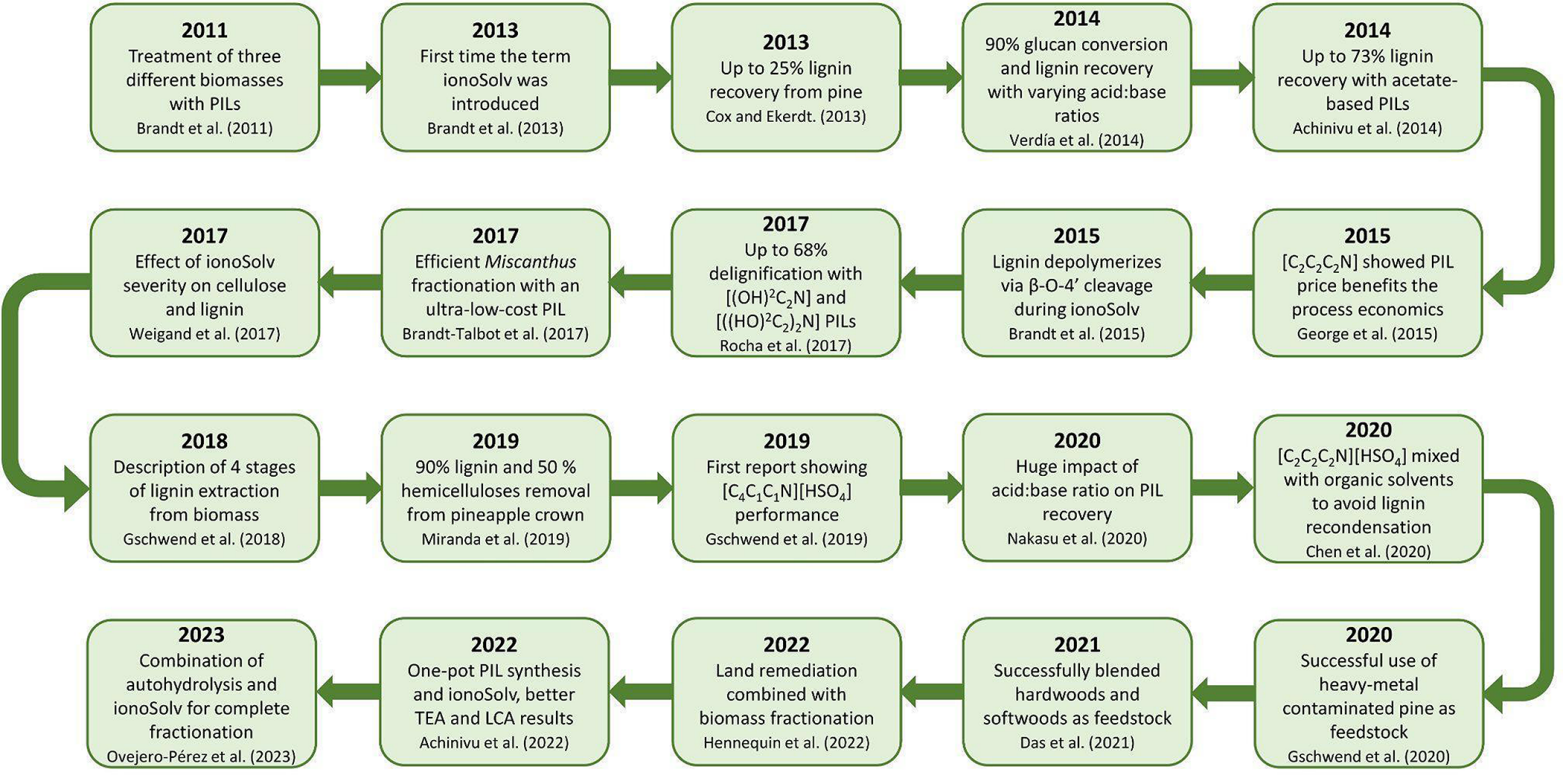
Some relevant works in the field of biomass fractionation with the ionoSolv process. References from this figure are: Brandt *et al*. (2011),^204^ Brandt *et al*. (2013),^39^ Cox and Ekerdt (2013),^348^ Verdía *et al*. (2014),^337^ Achinivu *et al*. (2014),^349^ George *et al*. (2015),^168^ Brandt *et al*. (2015),^83^ Rocha *et al*. (2017),^342^ Brandt-Talbot *et al*. (2017),^174^ Weigand *et al*. (2017),^341^ Gschwend *et al*. (2018),^350^ Miranda *et al*. (2019),^351^ Gschwend *et al*. (2019),^340^ Nakasu *et al*. (2020),^178^ Chen *et al*. (2020),^352^ Gschwend *et al*. (2020),^353^ Das *et al*. (2021),^163^ Hennequin *et al*. (2022),^354^ Achinivu *et al*. (2022),^265^ and Ovejero-Pérez *et al*. (2023).^347^

Both the cation and anion play an important role in the dissolution of lignin. ILs with imidazolium cations are particularly suitable for the solubilization of lignin, since the aromatic ring of the cation can interact favorably with lignin rings through aromatic interactions.^355^ However, these ILs are more expensive than ammonium-based ones and are less environmentally friendly. The ammonium-based ILs are a highly commonly used family of ILs, where the amine can be primary, secondary or tertiary.^349^ The length of the cation chain affects the structure of the recovered lignins. A greater length gives rise to lignins of higher molecular weight, which can be used in additives or resins, while those with a shorter chain length produce the depolymerization of the structure, giving rise to monomers with applicability in fine chemistry.^167^ Regarding anions, ILs based on acetate anions are capable of better solubilizing lignin and also help to reduce the crystallinity of cellulose, while those based on the hydrogen sulfate anion are capable of extracting lignin by acid cleavage of C–O bonds, as well as degrading carbohydrates into HMF and furfural.^356^

IL properties (acid/base ratio, cation and anion, acidity) and process conditions (temperature, time, biomass loading) have a huge impact on biomass fractionation efficiency. By tuning these, the delignification stages explained before can be controlled to reach a high degree of delignification while maintaining a low lignin condensation and pseudo-lignin formation, that is defined as lignin-like polymer that is formed by the polymerization of sugar degradation products (furfural, HMF) and condensed lignin fragments.^357^ Lignin condensation and pseudo-lignin formation are well-known phenomena that are promoted by acidic media.^358^ It is normally important to avoid pseudo-lignin formation and lignin condensation for economic reasons. Pseudo-lignin usually precipitates back with the cellulosic material, making it more difficult to valorize the cellulose pulps, either by fermentative routes or in the search for advanced and high value-added materials.^359^ Lignin condensation also makes lignin valorization a challenge, since the recovered lignin is highly heterogeneous, with polydispersity values between 10 and 70 and high molecular weights.^360^ Increasing treatment severity (temperature, time, acidity) normally increases lignin removal from the biomass, as the intermolecular bonds in the lignocellulosic material as destabilized and the IL viscosity is lowered, improving mass transfer rate between the wood and the IL.^360^ In addition, working at temperatures higher than the glass transition of lignin (around 130–150 °C, depending on the lignin source) improves the kinetics of lignin removal.^361^ However, an excessive increase in process severity normally leads to the formation of the aforementioned pseudo-lignin and to the condensation of the extracted lignin fragments.^340^

The recovered cellulose after the ionoSolv treatment normally maintains its crystallinity, the surface area increases and pore size decreases.^362^ However, fiber length in the recovered cellulose is shorter than that of the original cellulose, which limits its potential valorization to applications that require long fiber length. Due to the ability of the PILs of removing lignin and hemicelluloses from the lignocellulosic material, ionoSolv cellulose pulps are normally of high purity, giving rise to a high potential for material applications, such as gels or cellulose fibers, among others.^359,363^

To determine ionoSolv treatment effectiveness, researchers have looked into enzymatic digestibility of the recovered cellulosic pulps, as the elimination of lignin and hemicelluloses normally increases cellulose accessibility for the enzymes. In addition, they can act as inhibitory compounds due to nonproductive enzyme binding during the saccharification.^364^ Thus, ionoSolv process conditions have an impact on cellulose digestibility, closely related to a higher or lower lignin elimination and later redeposition. Enzymatic digestibility normally increases with increasing the severity of the treatment due to a major cellulose purity. This has been observed with different biomass types, such as grasses, hardwoods and softwoods,^164,350,365^ and positive correlations between delignification and enzymatic saccharification yields have been reported.^164,350,365^ When pretreating *Miscanthus* with [C_2_C_2_C_2_N]­[HSO_4_] at different times, it was observed that increasing treatment time led to higher glucose releases during saccharification due to a lower lignin content in the cellulosic pulp up to a maximum. From that point, longer times led to lower digestibilities. This was attributed to the redeposition of pseudo-lignin on the surface of the cellulosic pulp at longer times, as observed by the authors. Indeed, bands attributed to pseudo-lignin redeposition have been found on FTIR spectra of *Miscanthus* pulps pretreated with [C_2_C_2_C_2_N]­[HSO_4_] at long pretreatment times.^362^ IonoSolv temperature has a similar effect on enzymatic saccharification yields. Increasing the treatment temperature normally causes better lignin extraction, and thus improves enzymatic digestibility, until pseudo-lignin redeposition begins, worsening saccharification yields.^350^ Another important effect of increasing treatment temperature is that shorter times can be employed, achieving similar or even better results in terms of saccharification yields, which could favorably impact the economics of the process.^350^ IL structure also plays a role in enzymatic digestibility. While it is true that acidic ILs, like those based on hydrogensulfate anion, are normally better for lignin extraction thanks to a more extensive cleavage of lignin β-O-4′ linkages, thus improving cellulose accessibility, the crystallinity of the cellulosic pulp is normally unaltered, a fact that also plays a role in enzymatic digestibility. In this sense, ILs based on the acetate anion have been reported to decrease cellulose crystallinity apart from removing lignin, which improves saccharification yields.

Lignin is normally recovered by water addition. The high molecular weight fragments precipitate first, while the smaller parts remain in dissolution and are harder to precipitate due to stronger π–π interactions of the lignin monomers aromatic rings and the IL cation. The difference in lignin fragments solubility makes it possible to fractionate the recovered lignin by controlling the water addition during the precipitation step, which opens up opportunities for advanced lignin valorization depending on the obtained molecular weights and polydispersities.^366^ Finally, water needs to be removed from the IL after lignin precipitation in order to reuse the PIL in another treatment step (as shown in Figure 15). The PILs employed in the ionoSolv treatment are normally highly soluble in water, which makes the IL recovery step highly energy intensive, representing one of the main challenges in the implementation of the ionoSolv process at a higher scale.

When designing a process scale-up of the ionoSolv treatment, it is important to think about increasing both solid loading and particle size, as higher solid loadings allow lower reactor volumes and IL consumption, and the possibility of working with high particle sizes makes grinding and feedstock preparation cheaper. According to Chambon *et al.* (2021), solid loading could be increased up to 20% if the stirring and the pulp washing are optimized.^182^ They also found that particle sizes of ∼3 × 0.02 × 0.01 cm^3^ are the most effective for the ionoSolv treatment since lignin extraction is enhanced without promoting lignin redeposition on the surface due to a high surface area availability.

#### 2.6.4 Comparative of the Different Biomass Pretreatment with ILs Mechanisms

Wang *et al*. found that lignin extraction from poplar with two acidic PILs based on chloride anion ([C_1_im]Cl and pyridinium chloride, [Py]­Cl) achieved higher lignin extraction yields (60.4 and 61.0%, respectively) at milder conditions (30 min and 100 °C) than those previously reported using the alkaline aprotic IL [C_2_C_1_im]­[C_1_CO_2_] (31.9% at 130 °C for 12 h; 10.1% at 125 °C for 1 h).^367^ X-ray diffraction (XRD) analysis of the recovered pulps showed no significant changes in the crystalline structure of the cellulose, suggesting that these PILs did not dissolved the cellulose fraction in the process.^367^ A comparison study by Hossain *et al*. found that the acidic PIL 1-ethylimidazolium chloride, [C_2_im]­Cl, showed similar effectiveness as the alkaline aprotic [C_2_C_1_im]­[C_1_CO_2_] (75% of glucose release vs 80%) and higher than the acidic AIL [C_2_C_1_im]­Cl. Their findings confirm the different action mechanism by which the acidic PIL does not dissolve the crystalline cellulose fractions. However, they found that the PIL [C_2_im]Cl was capable of dissolving up to 3.5 wt% of whole pine wood flour at 115 °C, showing an unusual capability to dissolve whole biomass, that could be influenced by the surface area/volume ratio of the solids employed. This whole biomass solubilization capability was found to be higher than that of the AIL [C_2_C_1_im]Cl (2.5 wt%), but lower than that of the alkaline AIL [C_2_C_1_im]­[C_1_CO_2_] (5.5 wt%). The alkaline PIL [C_1_im]­[C_1_CO_2_] dissociated at the tested conditions and was unable to dissolve the biomass.^249^

A further study comparing 3 AILs, 3 PILs and 3 BILs found that although the highest cellulose digestibility was achieved with the alkaline AIL [C_2_C_1_im]­[C_1_CO_2_] (84%), the best ability extracting hemicellulose and lignin from hardwoods (*Eucalyptus*, 100% hemicellulose removal and 52% delignification) and softwoods (pine, 100% hemicellulose removal and 43% delignification) was obtained with the acidic PIL [C_1_im]­Cl.^81^ On the other hand, [Ch]-derived ILs were successful in the pretreatment of *Eucalyptus* (with glucose digestibility values up to 69%) but showed limited effect on the softwood feedstock (pine) due to its higher recalcitrance. In another example, the alkaline BIL [Ch]­[Lys] was found to be more effective than the PIL [C_4_C_1_C_1_N]­[HSO_4_] removing lignin from rice and wheat straws at low temperatures (51% vs 38%, 8 h at 80 °C and 87% vs 77% at 120 °C, respectively). However, lignin recovery for all the alkaline ILs (both acetate and lysinate based) needed acidification of the IL and was significantly lower than their delignification ability, due to their alkaline character, which highlights the recycling issues faced by these types of ILs.^81,336^

Recently, Yao *et al*. proposed the possibility of taking advantage of the different mechanisms of specific ions by performing biomass pretreatment using mixtures of different ILs, commonly known as double salt ILs.^368^ The studied a combination of imidazolium, cholinium, lysinate, and acetate ions for the pretreatment of pine achieving 80% glucose yields, and a combination of cholinium, lysinate and palmitate for the pretreatment of sorghum, obtaining 98% glucose yields. Furthermore, such combinations were found to be effective at lower IL concentrations, rendering them biocompatible.

##### 2.6.4.1 Effect of Pretreatment with ILs on the Surface Morphology of Pulps

In addition to delignification and disruption of the crystalline structure of cellulose, increases in pulp surface area and porosity are also linked to an increase in enzymatic hydrolysis yields due to the improved accessibility for enzymes. Raj *et al*. found a linear correlation between pulp specific surface area and glucose yields after saccharification for Mustard stalk pulps.^369^ They compared 5 aprotic ILs and found that the highest increase in specific surface area with respect the raw material, 4.7-fold, was obtained with the alkaline IL [C_2_C_1_im]­[C_1_CO_2_], whereas the best acidic AIL, [C_2_C_1_im]­Cl, only achieved a 1.8-fold increase. These results are consistent with those reported by Torr *et al*. on the pretreatment of pine with the same 2 ILs.^370^ They reported that the pretreatment with [C_2_C_1_im]­[C_1_CO_2_] resulted in bigger surface area, defibrillation, and pore volume than the pretreatments with [C_2_C_1_im]Cl under the same conditions. Their results showed a linear correlation between internal pore volume and enzymatic saccharification, obtained under conditions where no delignification was observed and the disruption of the cellulose crystallinity only occurred for the most severe pretreatment.

For PILs, it has been reported that pretreatment of *Miscanthus* with [C_2_C_2_C_2_N]­[HSO_4_] led to an increase of up to eight-fold in the surface area of the pulp compared to the raw material (4.34 m^2^/g after 24 h vs 0.49 m^2^/g). On the other hand, the pore size was slightly reduced (from 8.5 nm to 6.9 nm after 4 h and 7.5 nm after 24 h of pretreatment), but SEM imaging showed the appearance of micropores not present in the raw biomass. A reduction in particle size, in particular in the longer axis of the particles, was also found with respect to the untreated materials. Interestingly, after reaching a minimum particle size at optimal pretreatment conditions, authors found an increase in both length and width of the particles at prolonged pretreatments, which they linked to a coating effect produced by the redeposition of pseudo-lignin on the particle surface.^362^ Different studies analyzing the surface of pulps from different feedstocks such as poplar, wheat straw, rice husk pretreated with different PILs, such as [C_2_C_1_N]­[HSO_4_], [C_2_C_2_C_2_N]­[HSO_4_] and [Py]­[H_2_PO_4_], have found the formation of pores and defibrillation occurring in the pretreated pulps, which have been reported as evidence of IL penetration into the fibers during the pretreatment.^371,372^

SEM imaging of pulps recovered after pretreatments (at 120 °C for 6 h) of the hardwood *Eucalyptus* and the softwood pine with different types of ILs (PILs, AILs, and [Ch] ILs) demonstrated how the different modes of action of different ILs and the different feedstock recalcitrance affect the surface morphology, which also has a big impact on its further processing (Figure 17). SEM imaging and confocal fluorescence microscopy (CFM) were used to observe differences in pulp surface morphology, lignin distribution, and redeposition patterns. Pulps pretreated with the AILs [C_2_C_1_im]­[C_1_CO_2_], 1-allyl-3-methylimidazolim chloride ([(C_1_=C_2_)­C_1_im]­Cl) and 1-ethyl-3-methylimidazolium dimethylphosphate ([C_2_C_1_im]­[(C_1_O)_2_PO_2_]) had lots of visible holes and surface porosity as a consequence of the dissolution and regeneration of wood fibers, with this increase in porosity being more accused in the case of [C_2_C_1_im]­[C_1_CO_2_]. Increased porosity was also observed for the [Ch] based ILs ([Ch]­[C_1_CO_2_], [Ch]­[Lys] and [Ch] serinate, [Ch]­[Ser]), in this case the size of the recovered particles was bigger than with AILs. Also, the lignin surface ratio increased for most of the *Eucalyptus* samples, independently of the type of IL employed, with the highest value obtained for the sample pretreated with [C_2_C_1_im]­[C_1_CO_2_].^81^ Pine pulps recovered after pretreatment with the PILs 2-hydroxyethylammonium formate ([(OH)^2^C_2_N]­[HCO_2_]) and 2-hydroxyethylammonium acetate ([(OH)^2^C_2_N]­[C_1_CO_2_]) did not show significant change in morphology. Pine pulps recovered with [C_1_im]Cl were smooth without visible pores, but their particle size increased compared to raw biomass and the lignin surface ratio decreased from 0.70 in the raw feedstock to 0.48 for the pretreated sample. On the other hand, the *Eucalyptus* recovered with [C_1_im]Cl showed a reduction in particle size, but again no visible pores were detected.

**Figure 17:**
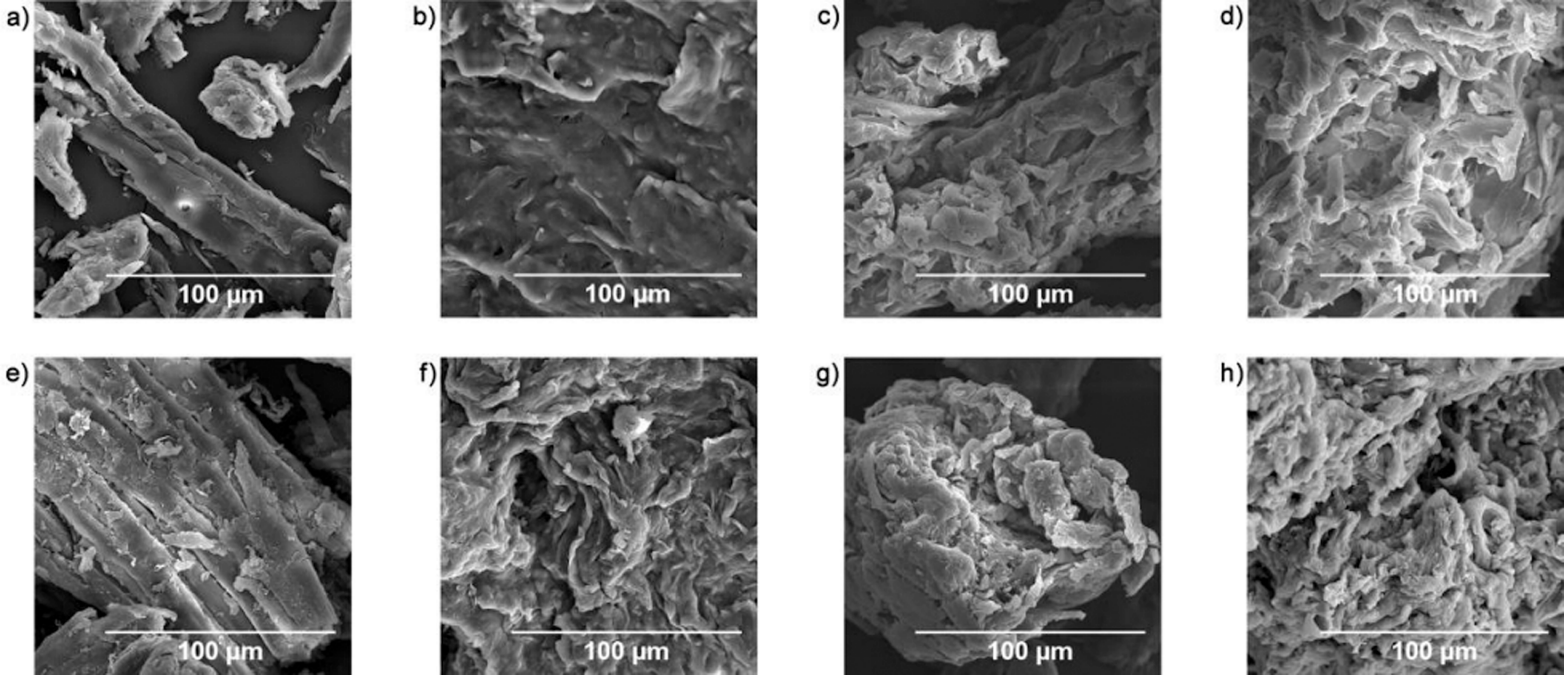
SEM micrographs of: (a) untreated pine; (b) pine pretreated with [C_1_im]Cl (c) pine pretreated with [C_2_C_1_im]­[C_1_CO_2_]; (d) pine pretreated with [Ch]­[C_1_CO_2_]; (e) untreated eucalyptus; (f) *Eucalyptus* pretreated with [C_1_im]­Cl; (g) *Eucalyptus* pretreated with [C_2_C_1_im]­[C_1_CO_2_]; (h) *Eucalyptus* pretreated with [Ch]­[C_1_CO_2_]. Adapted with permission from ref 81. Copyright 2019 American Chemical Society.

#### 2.6.5 Pretreatments with DESs

DESs also offer the possibility of using renewable feedstocks for their preparation, such as sugar-derived compounds, AAs, fatty acids, and even lignin monomers.^297^ Pretreatment processes with DESs are often similar to ionoSolv processes, where only the lignin and hemicellulose fractions are dissolved. The pretreatment of biomass with DESs usually enhances the digestibility and solubility of lignocellulose, reduces its resistance to enzymatic digestion and fragments the biomass selectively into its primary components.^373^ DES pretreatments can expedite the decomposition of constituents into a diverse array of chemicals with added value, such as phenolic compounds.^374,292,375^ The application of DES for delignification of lignocellulosic biomass reduces the crystallinity of cellulose and generates cracks in the pretreated biomass, thereby enhancing the subsequent processing of the entire bioprocess.

[Ch]Cl is one of the components frequently used to produce DESs for biomass pretreatment. Since it has a remarkable ability to receive H-bonds, it is used as an HBA.^376^ In the context of biomass pretreatment, investigated HBDs include acids, polyols, amides, monosaccharides and phenolic compounds. The pretreatment effectiveness was significantly impacted by the HBA/HBD combination, the molar ratio and the DESs/biomass mass ratio.^377,378^

The process of delignifying biomass and extracting value from lignin has garnered a lot of attention in recent years. The use of DES for delignification promotes a decrease in cellulose crystallinity and the formation of microvoids and fissures in the pretreated solids, thereby enhancing the downstream conversion.^379^ Pretreatment with DES can enhance the translocation of DES from the cytoplasm to the cell wall, where it can then make lignin in the secondary wall more easily accessible.^380^ The solubilization of lignin can be enhanced by modifying the chemical composition of DES while bearing in mind the novel lignin-first biorefinery approach. Because of this, several different HBAs, including [Ch]­Cl, betaine (bet), acetamide, and urea, among others, have been investigated for their potential to work in conjunction with different HBDs.^381,382^ The mixture of [Ch]Cl and organic acids has led to greater delignification rates than other HBAs, such as acetamide.^379^ For delignification, numerous carboxylic acids, including formic acid (FA), acetic acid, etc., as well as their combinations, have been utilized.^379,383^

Xu *et al.* investigated the first one-pot pretreatment of lignocellulosic biomass using DESs as novel solvent media, taking this into account as an alternative to conventional multistep procedures (Figure 18). The one-pot process is based on the use of biocompatible ILs/DESs that requires no solid–liquid separations or extensive pH adjustments between the unit operations of biomass pretreatment, enzymatic saccharification and fermentation. Biocompatible and biodegradable ILs are highly desirable for use in biorefinery applications due to their intrinsic compatibility with both downstream processes and ecosystems. Many imidazolium-based ILs are inhibitory to the commercial cellulases/hemicellulases and biofuel producing microbial strains, such as yeast and *Escherichia coli*, thus costly sugar separation out of the IL stream is required.^384,385^ For this reason, a lot of research efforts are being made towards the design of ILs that are compatible with the microbes and enzymes used for the processing of the biorefinery streams.^386–388^ Thus, complete biomass conversion could be theoretically completed using one reactor, or at least fewer tanks than the conventional biorefinery would otherwise require. The study was conducted and primarily focused on the introduction of a set of biocompatible DESs whose application in the one-pot conversion of biomass to biofuels appeared promising. The work focuses primarily on describing the one-pot pretreatment method that provides improved delignification efficiency. The delignified material was further valorized into benzoic acid and *p*-coumaric acid, which were then analyzed as the predominant lignin degradation compounds detected in the hydrolysates. It was demonstrated that a DES system based on [Ch]Cl and glycerol is an effective pretreatment solvent that enables the consolidation of saccharification and fermentation into a single-pot process that generates high yields of ethanol from corn stover biomass. The approach is promising because it reduces the need for pH adjustment or dilution between stages and reduces operational costs and environmental impacts.^142^

**Figure 18:**
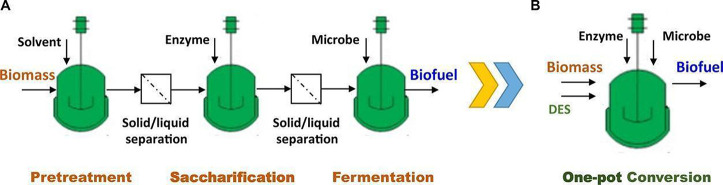
One-pot cellulosic ethanol production and delignification with biocompatible DES. Adapted with permission from ref 142. Copyright 2018 American Chemical Society.

Numerous studies on the nontoxicity of [Ch]Cl and glycerol have led to the adaptation of DESs as potential catalytic solvent media for a variety of applications. Biomass pretreatment for their valorization into biofuel is said to be one of the processes where DESs and ILs are able to be utilized and altered according to the process's specifications. In parallel, the role of water in DES pretreatment is significant. Control of the amount of water allows adjusting the characteristics of DES, including reducing viscosity, hence enhancing its efficiency in pretreatment.^389–391^

One-pot DES pretreatment studies on switchgrass biomass were conducted within the context of a multifunctional biorefinery employing an acidified and an aqueous DES comprising [Ch]Cl and glycerol (Gly), and the system was found to be highly effective under benign conditions. The authors described the composition of acidified DES as a mixture of [Ch]­Cl:Gly, combined with 0.9 wt% H_2_SO_4_. Meanwhile, the aqueous DES system was comprised of [Ch]­Cl:Gly, with the addition of 20–40 wt% water. The acidified DES resulted in a complete enzymatic hydrolysis of cellulose, whereas the aqueous DES containing 20% water produced a slightly lower glucose yield. The inclusion of water resulted in a drawback: a greater amount of energy was necessitated to recover and recycle the DES.^392^ Similar DES system ([Ch]­Cl:Gly aided with Ca­(OH)_2_) was applied to a novel one-pot consolidated pretreatment of *Saccahrum* spontaneous biomass (SSB), followed by enzymatic hydrolysis and ethanol fermentation. Ca­(OH)_2_ assisted [Ch]­Cl:Gly pretreatment reduced biomass recalcitrance and increased enzymatic saccharification, mainly due to its increased basicity. Following meticulous optimization of consolidated bioprocessing (CBP) for pretreatment and enzymatic saccharification, a significantly increased sugar yield of 372.3 mg/g biomass (approximately five-fold) was observed. Subsequent fermentation of the sugars with *Scheffersomyces stipitis* and *Saccharomyces cerevisiae* under CBP culminated in an ethanol content of 173.61 mg/g of feedstock, which accounted for 60.28% of the theoretical yield. It was observed that an increase in biomass loading in the tested DES systems increased viscosity and confined mixing, resulting in inefficient contact between the solvent and biomass particles and a less efficient process. The work performed at 5 wt% biomass loading resulted in a very low extraction of hemicellulose and lignin, which hinders the application of the studied DES systems in biorefineries.^393^

The recycling of [Ch]­Cl/Gly is one of the most significant obstacles to its industrial implementation. Abbott *et al*. studied the purification and recycling of DESs formed by different quaternary ammonium salts/glycerol that were originally employed to extract residual glycerol from biodiesel.^394^ Recrystallization of the salt ([Ch]­Cl), was attempted, with little success. The best efficiency, only a 25% was achieved when combined the addition of an antisolvent. However, distillation of glycerol may present several challenges, due to the high boiling point of glycerol, making vacuum distillation the only convenient solution. Needle-shaped crystals were observed when a mixture of [Ch]Cl and glycerol in a 1:2 ratio was subjected to cooling in an ice bath. The recovery of [Ch]Cl using this method was found to be only 4 wt%, as determined by electrochemical tests. To facilitate the separation of the salt upon cooling, an additional method utilizing a co-solvent, 1-butanol, was employed. Nevertheless, this strategy yielded a meager 25% recovery of salt from the mixture and was unoptimized; further efforts are necessary to devise a more effective method for salt recycling and subsequent glycerol recovery.

Concerns regarding [Ch]­Cl:Gly-based DESs motivate researchers to investigate alternative compositions by altering HBAs and HBDs.^395,396^ In this context, carboxylic-based HBDs have been studied for the conversion of individual biomass components to fermentable sugars and cellulosic ethanol. On the other hand, the application of DESs for improving delignification efficiency, for modification of the lignin moieties, and for enzymatic hydrolysis in a single-pot biomass pretreatment is still in its infancy. The delignification and enzymatic hydrolysis of cellulose are greatly influenced by the acid concentration and strength and by the nature of the HBDs. Hou and colleagues compared the pretreatment efficiency of acidic DESs using FAc and lactic acid (LA) as HBDs with that of neutral DESs using 1,4-butanediol as HBDs. The study revealed that DESs with fewer hydroxyl groups and a weaker intermolecular hydrogen bond (H-bond) strength exhibited superior biomass deconstruction properties.^397^ In another work, the application of a one-pot pretreatment using a betaine:lactic acid DES was utilized for the pretreatment of residues obtained from the production of xylose from corncob, leading to the successful separation and concentration of cellulose and lignin components. The enzymatic digestibility of the cellulosic residue obtained from the betaine/LA pretreatment at a temperature of 120 °C was found to be significantly higher, reaching 96.8%. Lignin was successfully extracted through precipitation using water, resulting in the presence of minimal quantities of neutral sugars and low molecular weight compounds. However, the authors demonstrated an inability to effectively recycle the used DES systems. The potential enhancement of profitability in the integrated process would be substantial if the recovery of studied DES systems were feasible.^398^ Temperature plays a crucial role in enhancing the conversion and delignification efficiencies of biomass pretreatment. However, the relationship between the operating temperature and the combination of HBAs and HBDs in DES is not straightforward. Kwang *et al*. attempted to compare the efficacy of [Ch]Cl and bet (HBAs)-based DESs combined with glycerol (HBD) in one of their recent studies. At elevated temperatures (180 °C), [Ch]­Cl:Gly performed better than bet:Gly in converting poplar biomass into fermentable sugars and fractionated lignin via a single-pot pretreatment (Figure 19). The effectiveness of the previous solvent can be attributed to the presence of hydrogen bonds between [Ch]Cl and glycerol. The formation of strong H-bonds between the OH groups of [Ch] and glycerol is facilitated by the chloride anion, which is responsible for the complex structure of [Ch]Cl and glycerol. Bet, a zwitterion with both positive and negative charges, establishes a eutectic mixture by interacting exclusively with the HBD. Indeed, the greater stability and stronger interactions between the DES components in bet:Gly could clarify why [Ch]­Cl:Gly outperformed bet:Gly in terms of the release of fermentable sugars.^399^

**Figure 19:**
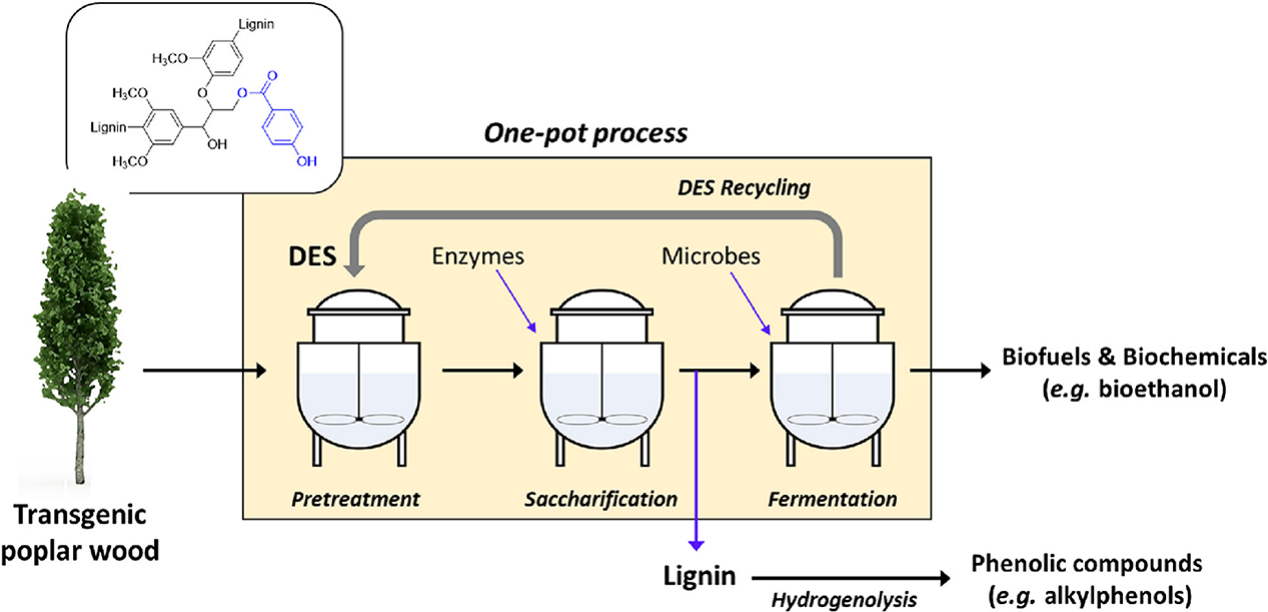
One-pot conversion of biomass into bioproducts using engineered poplar wood and biocompatible DESs. Adapted with permission from ref 399. Copyright 2022 Royal Society of Chemistry.

Previous research indicated that the use of [Ch]­Cl and polyol-based DES as a standalone pretreatment method has proven to be ineffectual in achieving satisfactory sugar yields, unless harsh conditions such as prolonged exposure to high temperatures (150 °C) are employed. In a general sense, it has been observed that acidic DESs, such as [Ch]­Cl:LA, exhibit greater effectiveness compared to non-acidic DESs, such as LA [Ch]­Cl:Gly, when subjected to equivalent levels of pretreatment severity.^389,400^ It is probable that acidity enhances the efficacy of pretreatment for non-acidic DESs under mild operating conditions. According to the study by Xu *et al*., 30–40% of the xylan was lost in the liquid phase following pretreatment, resulting in a significant loss of xylan mass. Consequently, the difficulty of obtaining a high xylose yield during the pretreatment and saccharification of biomass persists using a neutral solvent.^142^ Using a [Ch]­Cl:LA -based DES for pretreatment and saccharification of rice straw in a one-pot process, a significant xylose yield was documented. Huang *et al*. reported the one-pot pretreatment and saccharification (PSOP) process and asserted that it was advantageous and more effective than conventional one-pot pretreatment processes. With the proper concentration of [Ch]­Cl:LA-based DES, the study concluded that a total sugar yield of 75.7% could be attained due to the excellent xylose yield. Based on the reported sugar yield and delignification efficiency, it was established that the PSOP procedure was 13–15 times more energy efficient than conventional pretreatment methods.^401^ DES based on quaternary ammonium cations as HBA, coupled with highly basic anions such as chloride, acetate, etc., can be attributed to the improved results of sugar production with a tremendous xylose yield. According to a study conducted by Zhu *et al.*, it was observed that ammonium cations enhance the coordination ability of chloride anions. The charge transfer mechanism within HBA and HBD facilitates the formation of strong hydrogen bonds between −Cl of [Ch]­Cl and −OH of LA, which disrupts the hydrogen bonding between the original biomass and promotes its dissolution.^402^

The robust hydrogen bonding interaction between HBDs and HBAs imparts significant solvent strength, enabling it to effectively compete with the internal linkages present in biomass. Consequently, this leads to the disruption of hydrogen, glycosidic and ether bonds, thereby facilitating the fractionation of lignocellulose. Despite the efficient removal of lignin and hemicellulose by DESs, certain intrinsic challenges continue to impede their potential for widespread industrial utilization.^403^

A one-pot method utilizing a ternary DES consisting of [Ch]­Cl, ethylene glycol and AlCl_3_ was proposed, which was both fast and recyclable due to microwave assisted reaction.^404^ The primary objective of this approach was to effectively decrease the inherent resistance of biomass, commonly referred to as “biomass recalcitrance.” This reduction in recalcitrance resulted in the generation of lignin nanoparticles and facilitated the enzymatic saccharification of cellulose. The enzymatic saccharification efficiency of the pretreated poplar wood was extremely high (95.4%) and the DES lignin fractions displayed less-cleaved structural characteristics with homogeneous morphology, including nano-sized lignin with low molecular weights, limited distribution within the range of polydispersity index (PDI) values of 1.28–1.38, controllable size of 100 nm (Figure 20), and exceptional antioxidant activity.^404^ The inefficiency of feeble competing interactions between binary DESs and biomass linkages is the impetus for the design of ternary DES systems.^297^ In certain instances, it was discovered that the K-T parameters of [Ch]­Cl:polyol-based DESs, especially α and β values, were substantially smaller than those of acidic-based DESs.^405,406^ In this regard, a molecular design for a ternary DES was proposed based on an acidic multisite coordination strategy, in which metal compounds were chosen as anion donors and active acidic site holders.^403^ Wang *et al*. have also investigated the Lewis acid-catalyzed and polyol-based DES pretreatment, which has the potential to significantly improve fractionation ability. Nevertheless, the lignin valorization process was not given enough attention throughout these DES pretreatment procedures.

**Figure 20:**
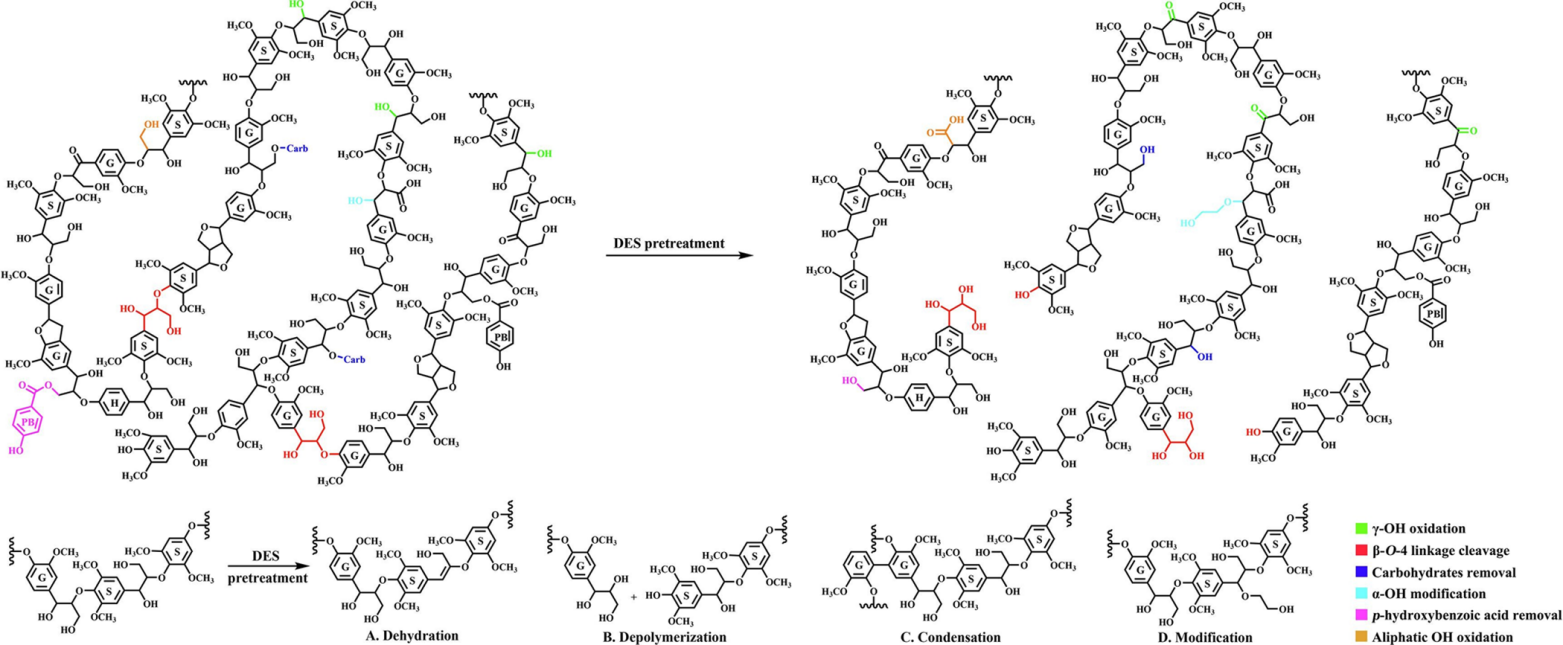
Proposed structural evolution of lignin during the DES pretreatment. Adapted with permission from ref 404. Copyright 2022 Elsevier Ltd.

It could be argued that lignin isolation and its utilization have become a major barrier to large-scale DES implementation.^407^

Recent research work has demonstrated the synergistic effect of digestible substrate and lignin microspheres using a single-step DES pretreatment at low temperature and large solid loading. The optimal condition yielded 70% lignin removal and over 90% recovery yield, with 100% enzymatic saccharification after pretreatment. DES solution recycling retrieved above 90% DES. The recovered lignins had a consistent microspherical shape and a controlled diameter of 1100–6182 nm. The unique biorefinery paradigm for producing many products in one pot could help develop a green and sustainable biorefinery sequence from economic and ecological perspectives.^408^

DES has shown promising effectiveness in fractionating unprocessed lignocellulosic biomass, extracting lignin, and upgrading. This permits the autonomous enhancement of biopolymers based on their intrinsic properties. Despite this, the DES type that is best suited for the conversion of biomass is not yet known, and it might vary depending on the desired product, the starting feedstock and the process conditions. Moreover, there is a paucity of product diversity in the downstream conversion of DES-fractionated products. Several aspects require additional research: (1) elucidation of the mechanisms of DES fractionation of lignocellulosic biomass, (2) understanding the properties of various DES lignin, (3) correlation of measurable parameters with DES delignification performance, (4) development of effective DESs recycling technologies and (5) design/exploration of DESs compatible catalysts, such as biocatalysts and electrochemical catalysts. To advance DES fractionation technology, extensive research must be performed on solvent selection, bioproduct diversification, and techno-economics. It would be reasonable to anticipate the development of biomass utilization and lignin-upgrading DES systems with adjustable properties if research expenditures increase in the near future. Successful development of high performance, DES-compatible biocatalysts, and catalysts would allow for the consolidation or sequential extraction and refining of lignin, resulting in cost-effective lignin valorization.

### 2.7 Models of Interaction between ILs and Biomass

The main characteristic of an IL aimed for biomass pretreatment is its capacity to solubilize at least some of the components of the biomass. There are two main model types that describe the interaction between IL and biomass, namely polarity models like the K-T model, which are the most employed, and computational models.^409,410^

#### 2.7.1 The Kamlet–Taft Model

The K-T model uses three parameters: H-bond donating ability or acidity (α), H-bond accepting ability or basicity (β) and polarity and polarizability (π*).^297^ These have been widely applied to elucidate the effect of acidity of the ILs and DESs on biomass pretreatment and their ability to dissolve cellulose and to extract lignin. It’s been observed that ILs with high β values (higher than 0.82) have great capacity of breaking cellulose hydrogen bonds and thus solubilize the polymer.^409^

Furthermore, several studies have suggested that solvents with high β and high π* can dissolve lignin due to their ability to disrupt the H-bond network of lignin.^411^ On the other hand, different analysis have suggested that the α and β values of DESs are more strongly related to the lignin extraction ability than the π*.^297^ This would imply that the lignin-dissolving ability of an IL would be related to the difference between the values of α and β (α–β), which reflects the net H-bond-donating ability of the solvent. ILs and DESs with high α–β values could form strong H-bonds with lignin.^297^

#### 2.7.2 Computational Tools for the Selection of ILs for Biomass Processing

To synthesize ILs for industrial applications, it is key to comprehend how the anion, cation, and side chains on the cation influence their physicochemical properties. This can be accomplished via experimentation or by computational methods. Given the vast number of candidates, it would be difficult to select an optimal IL through experimentation. Scientists have developed systematic computer simulation methodologies, such as computer-aided molecular design (CAMD) and process design simulations that can help design ILs with the best properties for a given application. Computer-aided methods allow investigation of more IL solutions in a fraction of the time it would take for an experimental assessment. However, computer-assisted methods cannot supplant experiments, as the performance of the most promising discovered candidates must be verified through experiments.

CAMD approaches are effective for designing products based on chemical, biological and material chemistry. It can create molecular structures with a specific property in mind. The availability and reliability of prediction models are essential to the use of CAMD in the development of ILs. Only after characterizing thermophysical parameters such as density and viscosity (which impact mass transfer rates), the formation of suitable ILs and/or processes can be accomplished.^412^ Although accurate predictive models for the majority of thermophysical properties are still being developed,^413^ the free ILThermo database contains thermophysical property data for a wide variety of ILs.^414^ Predictive models aid in the design/development of new processes, the improvement of operational conditions and the reduction of energy consumption.^413^ Using various techniques, thermophysical and transport property prediction models for ILs have been developed.

The most prevalent development in CAMD is the utilization of COSMO (conductor-like screening model for real solvents) based methods, as binary interaction parameters are not required.^415,416^ COSMO-RS^417^ and COSMO-SAC^418^ are the two most frequently used CAMD methods.^415^ For thermodynamic calculations, these methods only require the estimation of molecular volumes and sigma profiles.

COSMO-RS is a tool widely used for identification of solvents. As input, the model only needs molecular information about the system. With that, it is able to provide direct and efficient pathways for the prediction of thermodynamic parameters of a mixture, including the limiting activity coefficient (γ^∞^) or excess enthalpy (*H* ^E^).^419–423^ Cellulose and lignin solubilities have been studied with the aid of COSMO-RS, which enables rapid identification of the most suitable cation and anion combinations from large sets of ILs for biomass processing. This approach increases the opportunities for identifying potential solvents that would otherwise be experimentally impossible to check, due to the enormous number of ILs that can be tested. Another important feature of this computational approach is that it can quantify the different intermolecular interactions, such as hydrogen bonds and and electrostatic forces, which enhances the understanding of the solubilization process and contributes to a better selection of ILs with the desired characteristics for specific applications.^424^

Activity coefficient was first employed as an indicator of cellulose and lignin dissolution, where the more negative the value of the activity coefficient of cellulose in the IL at infinite dilution, the higher the solubilization ability.^425^ Then, the excess enthalpy of the cellulose or lignin + IL mixtures was introduced as another reference property to predict solubility. It presents the same trend as the activity coefficient, leading to the same results, but has the advantage of giving quantitative description of the intermolecular interactions, enhancing the understanding of the mixture behavior.^420^ Higher cellulose/lignin solubilities are achieved at more exothermic behavior of excess enthalpy.^420,426^

ILs are relatively easy to implement in COSMO-RS and other computational models, but the incorporation of the solute has always been a challenge for effective prediction of biopolymers solubility. Glucose and simpler cellulose-like glycosides were first used as models for cellulose. These are crude models since they fail to account for the dominant intramolecular forces in the cellulose structure but do result in a reasonable calculation efficiency.^420,425^ More complex cellulose structures were then employed to better understand the interactions between ILs and cellulose and to predict more accurately cellulose solubility. These include cellobiose, cellotriose and higher size glucose oligomers, as well as tridimensional glucose structures.^420,425,427–432^

Kahlen *et al.* (2010) employed COSMO-RS to screen more than 2000 kinds of ILs to investigate how these interacted with a trisaccharide as a model for cellulose. They observed that ILs anions quickly accepted hydrogen bonds, thereby dominating the dissolution process.^425^ Casas *et al.* (2012) investigated the solubility of cellulose and lignin in 780 ILs by employing COSMO-RS calculation method of activity coefficients and excess enthalpy, showing that when the dissolution process is exothermic and cellulose and lignin in ILs have low activity coefficients, ILs could always solubilize cellulose to a high extent.^420^ They then expanded the system to a 3 × 3 cellulose monolayer model and calculated the optimal configuration, the activity coefficient, and excess enthalpy after mixing the cellulose model with 12 different ILs, getting consistent results with those from experimental work.^427^ Liu *et al.* (2016) employed COSMO-RS to predict new IL structures for cellulose dissolution, the first work guiding the design of new ILs for cellulose solubilization with computational methods. The experimental solubility of cellulose in these ILs was consistent with the model predictions.^433^ They identified H-bonds between anions and cellulose as the governing factor in the dissolution process, and the design of the anions, cation, and cationic substituents was refined using data from the prediction model. More recently, Chu *et al.* (2018) used COSMO-RS to test cellulose solubility based in four cellulose models (glucose, cellobiose, cellotriose and cellotetraose) with more than 350 ILs and showed that, in general, activity coefficient at infinite dilution gave better results in the prediction of cellulose solubility and the excess enthalpy was also able to predict solubility with halogen-based ILs.^434^ They also showed that the cellobiose was the best model out of the four tested due to a better conformational structure optimization, proving that COSMO-RS is very structure optimization-dependent, thus highlighting the importance of properly studying the geometry optimization methods.^434^

Lignin structure is more complex than cellulose, which has led to difficulties to predict lignin solubility in ILs. Lignin monomers, such as *p*-coumaryl alcohol, coniferyl alcohol and sinapyl alcohol were first employed as models for the computational calculations.^435^ With that, Balaji *et al.* (2012) could first successfully analyze the solubilization of those three model compounds with 1156 ILs, identifying potential candidates for lignin dissolution. However, they could not give validation data from experiments, since those three models, although present in the lignin network, are not fully representative of the intramolecular forces. Consequently, more complex lignin structures that consider lignin interunit linkages, such as pinoresinol or guaiacylglycerol-2-coniferyl ether, were incorporated into the COSMO-RS calculations successfully.^420,427^ It was demonstrated that acetate, formate, and chloride anions were beneficial for the solubilization of lignin, as the excess enthalpy and activity coefficient at infinite dilution were the lowest of the tested ILs and, as happened with cellulose, the anion effect was predominant over the cation effect.^420^ They also demonstrated that the high solubility of lignin in acetate and chloride-based ILs (high H-bond acceptor character) was due to a favorable H-bond interaction between solute and solvent, and, on the other hand, solute–solute interactions were stronger by H-bonding in the cases lignin wasn’t soluble in IL. This happened with large anions with a dispersed charge, such as [PF_6_]^−^ and [(CF_3_SO_2_)_2_N]^−^.^427^ Nevertheless, the experimental results did not perfectly match with the computational calculations. This was mainly attributed to the lack of more realistic models for lignin, as the employed ones were small fragments from the complex lignin structure, not taking into consideration the effect of intramolecular forces and structural impediments. More recently, Yu *et al.* (2022) tried 19 different lignin models formed by different combinations of the main lignin interunit linkages (β-O-4′, β–β′, and β-5′) with the three main lignin units (S, G, H).^423^ They revealed that the ILs with strongly polar anions and weakly polar cations were more likely to solubilize lignin and pointed out the critical effect of H-bonds in dissolution, as ILs are involved in dissolution as H-bond acceptors. Strangely, the best lignin model to predict lignin solubility according to the experimental results was sinapyl alcohol (S), a small molecule that cannot be considered fully representative of the lignin network or capable of capturing the entropy effects involved in biopolymer dissolution.

The lignin molecule is normally not well described by computational methods, making it difficult to make general rules to summarize and predict lignin solubility.^426^ Efforts are recently being made in this regard, employing larger lignin models that consider all the possible linkages and units in the same molecule, describing lignin solubility parameters by molecular simulation strategies and COSMO-RS in the range of 23–27 MPa^1/2^ and comparing them with the solubility parameters of ILs.^436^ They concluded that ILs with solubility parameters closer to that of lignin were more suitable solvents and were able to correlate the computational results with experiments. In addition, the solubility parameters calculated from their lignin model were close and applicable to lignin from hardwood and grasses, a huge and successful attempt to correlate computational results with real world lignins. In addition, recent studies are also focusing on explaining lignin dissolution by means of other IL properties, such as viscosity, dissociation constants and interaction energy between cation and anion. ILs with weaker interactions with the counter ion, low viscosity, high H-bond basicity and close solubility parameters were found to be better solvents for lignin.

Density functional theory (DFT) is another widely used computational method for solubility modeling, playing an important role in understanding the intricate interactions between cellulose and ionic liquids, providing insights into the mechanisms behind cellulose dissolution. First studies focused on understanding these cellulose–IL interactions, quickly realizing that H-bonds play a crucial role in cellulose dissolution. This fact was described by different authors like Zhang *et al.* (2005), Remsing *et al.* (2006) and Youngs *et al.* (2007), where they could establish a preferential binding between the IL anion (more remarkable Cl^–^) and the hydroxyl protons of cellulose by H-bonds, being even stronger that those formed inside the cellulose molecule, resulting in a more stable configuration for the system, where the anion-hydroxyl group H-bonds can replace the original H-bonds in the cellulose.^428,437–440^ In addition, some findings suggested that the free cations form complexes with the hydroxyl oxygen, disrupting hydrogen bonds within cellulose and facilitating its dissolution.^440^ Guo *et al.* (2010) also incorporated other anions for studying cellulose solubility, concluding that the strength of these interactions follows the order: acetate anion > alkylphosphate anion > tetrafluoroborate anion > hexafluorophosphate anion.^441^ Later studies, using more complex systems, like cellobiose to simulate the cellulose molecule, showed that not only the anion plays a role in cellulose dissolution, but also the cation, reacting with the cellulose and promoting its dissolution^437^ A study by Wei *et al.* (2013) reported that the cation (triethylamine) helped in ring-opening of the glucose molecule, thus facilitating cellulose solubilization in the IL.^442^ Similarly, Du and Qian (2011) investigated the ring-opening reaction through *ab initio* calculations, revealing that the breaking of the C–O bond within the glucose ring was a crucial step.^443^ An in-depth study of the ring-opening reaction was reported by Yao *et al.* (2015), revealing that the cleavage of cellobiose in ILs required low energy to overcome the activation barrier, owing to a synergistic effect between the cation and anion, being also affected by them.^444^ The Cl^–^ anion exhibited a noticeable effect on different catalytic sites, whereas the influence of the [OAc]- anion was minimal. In the case of the ring-opening reaction of cellobiose in acetate-based ILs, computational analysis suggested that the formation of carbene in ILs facilitated the ring-opening process by attacking the monosaccharide ring, resulting in the formation of a covalent bond between cellulose and the imidazolium (cation) core, leading to ring opening. Apart from hydrogen bonding, it seems that interactions between cellulose and IL play also a role in cellulose solubility, as reported by Cao *et al.* (2016) employing cellobiose as model system using DFT method.^445^ DFT calculations have been also used to investigate the regeneration of cellulose from cellulose/IL/water mixtures. Fu *et al.* (2022) reported that the addition of water to the IL system promotes cellulose regeneration by forming hydrogen bonds between water, cellulose hydroxyl groups, and IL oxygen atoms, precipitating the cellulosic fraction.^446^

Lignin solubility has also been investigated by DFT methods. Janesko (2011) reported that the interactions between lignin and the IL cation are influenced by both hydrogen bonding and π stacking interactions, showing comparable interactions with the cation and the anion.^430^ In addition, they also suggested the possibility of adjusting the relative degrees of lignin versus cellulose dissolution by manipulating the π-stacking (would favor lignin solubility) versus hydrogen bonding properties of the IL cation (would favor cellulose solubility.^430^ It has been found that the interactions between lignin and ILs involve both the anion and cation of the IL, with hydrogen bonding playing a significant role in the interaction between lignin and ILs, with anions forming stronger hydrogen bonds with the lignin model compound than cations.^447^ In contrast to the prevalent hydrogen bonding in lignin–anion interactions, interactions between the cation and lignin involve a combination of hydrogen bonding and π-stacking. These cation–lignin interactions significantly alter the intramolecular hydrogen bonds within lignin oligomers, thus promoting lignin solubilization. Zhu *et al*. (2018) also reported the importance of both anion and cation in lignin solubilization, where the cation acts as a Brønsted acid, protonating the α-hydroxyl group of lignin and then, in its deprotonated form, acting as a base accepting protons from lignin C_β_ and water.^448^ However, and as it happened with cellulose, hydrogen-bonding and anion interactions with lignin are more predominant than cation effect and π-stacking interactions in lignin dissolution.^449^ Therefore, in the creation of an effective IL solvent for lignin, anions characterized by strong hydrogen bond basicity, smaller volume, and the capacity to form multiple hydrogen bonds are advantageous for promoting lignin dissolution. Conversely, longer alkyl substituents may lead to increased viscosity and steric hindrance, potentially reducing dissolution capability. Cations possessing excessively short alkyl chains tend to form strong associations with anions, necessitating a careful balance between chain length and solubility in practical applications.^449^

All in all, computational methods are a powerful tool for predicting cellulose and lignin solubilities in ILs, as they increment the screening possibilities to a large number of cation–anion combinations and explain some of the mechanisms governing solubilization. However, the main limitation is found on the complex structure of biopolymers, especially lignin, which leads to inaccuracy compared to experimental results. Although some impressive progress has been made, there is still work to be done to fully understand and predict solubilities. Another important factor to consider is that these methods are being employed with isolated lignin or cellulose models, not considering the complex structure of lignocellulosic biomass and the interaction between lignin and cellulose (and hemicellulose) within the lignocellulosic matrix.

### 2.8 Effects of Pretreatment with ILs and DESs on Lignin

In traditional biorefining processes lignin is seen as an undesirable component of biomass that hinders access to the valuable polysaccharides, increasing the processing needs and associated costs. Due to the increase in production of biofuels, it is expected that by 2030 the annual production of lignin will reach 225 million tons/year.^450^ Valorization of this stream of material is necessary to improve the green credentials of biorefineries by reducing the amount of waste produced, which can hinder their environmental friendliness. It would also increase the versatility of biorefineries and improve the economics by turning a waste stream into valuable products. Furthermore, lignin is the only major renewable source of aromatics and the second most prevalent biopolymer globally. Hence, it has a huge potential as a versatile feedstock for materials and chemicals. However, at present, less than 2% of lignin is employed as a feedstock for the production of dispersants, surfactants, wood adhesives and other specialty products.^450,451^ In first-generation biomass processing plants, it is partially burnt for energy production (∼40%), with the rest being discarded (∼60%).^76^ For these reasons, there is a need for developing new processes that can produce valuable products from lignin. To address these concerns, the "lignin-first" biorefinery approach has been coined, where the valorization of lignin is one of the main goals.^452^

Hence, the process of delignification holds significant importance within the biorefinery framework, as it plays a pivotal role in facilitating the effective transformation of lignocellulosic biomass into bio-based commodities and biofuels.^441^ It is imperative to acknowledge, though, that the viability of biorefineries utilizing lignin as a primary component relies heavily on continuous research and development efforts aimed at improving delignification techniques and identifying novel applications for the resulting lignin streams.^453,454^ For this, understanding the nature of lignin is key. However, the structure and physicochemical properties of lignin recovered from biorefining processes differs from that of the native lignin, as it is found in plants and are highly dependent on the biomass processing and lignin recovery conditions. The majority of lignin residues recovered from biorefining processes are referred to as “technical lignins” due to different reactions that occur during pretreatment and other unit operations. During these reactions, naturally occurring carbon–oxygen bonds (C–O) are cleaved, followed by rapid carbon–carbon (C–C) bond formation. Due to the abundance of C–C bonds, which have a greater bond-dissociation energy than C–O bonds, “technical lignins” are challenging to depolymerize.^452,455^ Hence, to be able to tailor biorefining production processes to yield lignin streams aimed to be used as a feedstock for valuable products, first we need to understand how lignin interacts with the solvents and what transformations it suffers during such processes. Therefore, the recent biomass pretreatment methods have mostly focused on modification of lignin structure in conjunction with the pretreatment conducted on the raw biomass.

#### 2.8.1 Delignification of Biomass with ILs and Interactions of Lignin with ILs

Integrated biorefinery processes should aim for an appropriate and efficient valorization of the feedstock materials to be competitive. This includes the transformation of lignin, which can account for up to 35 wt% and 40% of the energy potential of the feedstock, into valuable products.^456^ For this reason, in addition to the efficiency of lignin removal, the effect of the pretreatment on lignin structure is also of primary importance. Understanding how the pretreatment parameters affect the physicochemical properties of lignin will allow one to fine-tune biorefining processes to maximize the value of the lignin output.

Independent of the process employed for lignin isolation, lignin recovered from biomass processing suffers chemical alterations with respect to its native form. These alterations can be notable and include depolymerization, recondensation and functionalization reactions. Therefore, the recovered fractions can present significant differences in their physicochemical properties depending on the processing conditions, which can hinder their downstream valorization. These lignins fractions isolated from biomass processing are referred to as technical lignins and usually receive the name of the processing technology (*e.g.*, Kraft lignin or ionoSolv lignin). In literature, milled wood lignin is often regarded as the technical lignin that most resembles native lignin, and is often employed as a model for comparison against other technical lignins.^457^ Many studies have shown that lignin isolated after pretreatment with ILs show traces of contamination with the IL, often detected by HSQC by characteristic IL peaks, by elemental analysis by traces of sulfur and/or nitrogen content and Py-GC-MS analysis.^83,350^ Levels of IL contamination seem to be generally low (values up to 2.5 wt% have been reported), and it does not seem to increase significantly with pretreatment severity.

Many studies use technical lignins isolated from Kraft, alkaline, or Organosolv processes to study lignin solubility and reactivity in different media, including ILs and DESs. These studies can provide useful information regarding dissolution mechanisms and lignin reactivity in ILs. This information can help in making informed decisions when designed processes aimed to valorize the final lignin streams into different products. However, the solubility of these lignin preparations in a given IL does not necessarily translate into the ability of the same IL in solubilizing native lignin from lignocellulosic biomass during pretreatment due to the disparate nature of native and different technical lignins.

#### 2.8.2 Lignin Solubility in ILs

The efficiency of delignification and the chemical changes suffered by lignin are dependent on the physicochemical properties of the solvent of choice and their hydrogen bonding capabilities. Lignin has amphiphilic behavior due to the presence of moieties with different polarity in its structure. The best candidates for lignin solubilization are solvents capable of disrupting both the hydrogen bonding and the hydrophobic interactions between lignin molecules.^297^ In particular, hydrogen bonding between lignin and solvent has a key role in the removal of lignin from lignocellulosic biomass, and it has been suggested that the solubility of lignin could be related to the total amount of solvent–lignin hydrogen bonds (H_T_).^458^ ILs are capable of many different interactions, including coulombic, hydrogen bonding, π–π stacking, ion-dipole or VdW interactions, and present nanostructuralization in polar and apolar regions of different size depending on the IL structure.^459–461^ All of this makes them ideal candidates for lignin solubilization.

##### 2.8.2.1 Effect of the Anion in Lignin Solubility and Chemistry

Anion choice appears to have the biggest impact in the solubility of lignin in ILs. For 1,3-dialkylimidazolium based ILs, anions with intermediate values of hydrogen-bond basicity (β), such as trifluoromethyl sulfonate (triflate, [CF_3_SO_3_]^−^), methyl sulfate ([C_1_SO_4_]^−^), chloride (Cl^−^), bromide ([Br]^−^) and acetate ([C_1_CO_2_]^−^), seem to give higher lignin solubilities.^39^ Results from different studies on Kraft lignin suggest that ILs with small anions such as formate ([HCO_2_]^−^), methyl sulfate ([C_1_SO_4_]^−^), dicyanamide ([N­(CN)_2_]^−^) or chloride ([Cl]^−^), are capable of establishing attractive forces with the −OH groups of lignin, resulting in a much higher ability to solubilize it than ILs with bulky, noncoordinating anions such as [(CF_3_SO_2_)_2_N]^−^, [BF_4_]^−^ or [PF_6_]^−^. Furthermore, ILs with alkyl sulfate anions have been found to be effective for lignin depolymerization, followed by ILs with lactate, acetate, chloride and phosphates, with the cations having little or no effect. This suggests that the anions act as nucleophiles during the depolymerization of lignin, which is further supported by the reported increased sulfur content in lignins recovered after treatment with ILs with sulfur containing anions (*e.g.*, sulfates, sulfonates, and sulfamates).^39^

Biomass delignification is directed not only by the lignin solubilization ability of the IL but also by its ability to cleave the covalent linkages between lignin and carbohydrates. This cleavage is directed by the anions, acting as nucleophiles.^164^ Therefore, ILs containing anions with higher β values yield better delignification (*e.g.,* ILs with [Lys]^‑^ anions have been reported as more effective than [C_1_CO_2_]^–^ anions).^,317^

Analysis of the lignin obtained with different types of ILs in the treatment of post-consumer waste woods (wood contaminated with the presence of heavy metal content) showed that lignin obtained with three [HSO_4_]^−^ based ILs showed higher degrees of condensation and ether cleavages than lignin recovered with ILs based on the anions [C_1_CO_2_]^–^ and Cl^–^, likely due to the acidity of the [HSO_4_]^–^ anion. However, this did not correlate with the delignification ability, suggesting there are other factors in play.^462^

##### 2.8.2.2 Effect of the Cation in Lignin Solubility and Chemistry

Although the nature of the anion is the main factor determining the solvation chemistry in ILs, the nature of the cation also plays a role. It has been described that ILs containing cations with high hydrogen bond acidity (α) are capable of effective delignification by the formation of hydrogen bonds with ether and hydroxyl groups of lignin.^317^ It has been also suggested that stronger cation–anion interactions in the IL led to weaker the interaction of the anion with the lignin solutes.^344^

The number and length of alkyl chains on the cation also plays a crucial role in pretreatment effectiveness. Early research by George *et al*. demonstrated that for a series of PILs based on the hydrogensulfate ([HSO_4_]^−^) anion, with a variety of ammonium-based cations selected for their cost and availability, the diethyl-, triethyl-, and diisopropylammonium ILs achieved the better delignification (Switchgrass, *Panicum virgatum*), surpassing that of the AIL [C_2_C_1_im]­[C_1_CO_2_].^168^ The comparison of [C_4_C_1_C_1_N]­[HSO_4_] with PILs with lower number of alkyl chains and, hence, higher acidity (*i.e.,* ethyl­ammonium hydrogen­sulfate, [C_2_N]​[HSO_4_]; 2-hydroxy­ethyl­ammonium hydrogen­sulfate, [(OH)^2^C_2_N]​[HSO_4_]; and butyl-*N*-methyl­ammonium hydrogen­sulfate, [C_4_C_1_N]​[HSO_4_]) showed that the PILs with fewer alkyl chains accelerated the delignification due to their higher acidity. However, this led to the early formation of pseudo lignin by recondensation reactions between lignin and sugar degradation products, showing that pretreatment conditions need readjustment.^168,462^ It has been also suggested that cations containing hydroxyl groups can enhance biomass deconstruction contributing to hydrogen bonding synergistically with the anions.^164^

Work by Gschwendt *et al*. on the pretreatment of the softwood species *Pinus sylvestris*, showed that lignin recoveries and saccharification yields obtained with [C_4_C_1_C_1_N]­[HSO_4_] doubled those obtained with [C_2_C_2_C_2_N]­[HSO_4_] (72% and 74% vs 30 and 36%, respectively).^340^ [C_4_C_1_C_1_N]­[HSO_4_] also yielded lignins with the lowest amount of β-O-4′ linkages and Mn and the highest Mw and PDIs, suggesting improved mass transfer rates and higher reactivities. These striking differences are more surprising considering that both ILs are isomers, with the same molecular formula and mass, and showed similar effectiveness with grass and hardwoods. However, both cations have different alkyl chains and symmetry. [C_4_C_1_C_1_N]­[HSO_4_] has a highly asymmetric cation and so it has lower melting point and lower viscosity than [C_2_C_2_C_2_N]­[HSO_4_]. Moreover, the aggregation of the longer butyl chain in [C_4_C_1_C_1_N]­[HSO_4_] allows the formation of a bigger structured nonpolar domain in the bulk of [C_4_C_1_C_1_N]­[HSO_4_] compared to [C_2_C_2_C_2_N]­[HSO_4_], which only has short ethyl groups. The formation of these nanoregions in the IL media could help stabilize lignin oligomers, which have an amphiphilic nature.^460,463^ The benefits of using [C_4_C_1_C_1_N]­[HSO_4_] only become apparent for softwoods due to their higher recalcitrance, while the dissolving ability of [C_2_C_2_C_2_N]­[HSO_4_] is enough to effectively dissolve the easier to remove lignin of grasses and hardwoods.

#### 2.8.3 Bulk Changes in Lignin during Pretreatment

##### 2.8.3.1 Influence of Severity (Temperature, Time, and Acidity)

The final structure and physicochemical properties of the recovered lignin can be tuned by varying the conditions of the pretreatment. Residence time and temperature are fundamental parameters for pretreatment. Nakasu *et al*. have shown that the correlation of the effect of these two parameters, pretreatment residence time and temperature, on the parameters chosen to evaluate the pretreatment efficiency, such as delignification, can be visualized using response surface graphs (*e.g.*, a response surface for delignification vs time and temperature is shown Figure 21). Hence, these are useful tools to show the trends and selecting optimal pretreatment conditions.^164^

**Figure 21:**
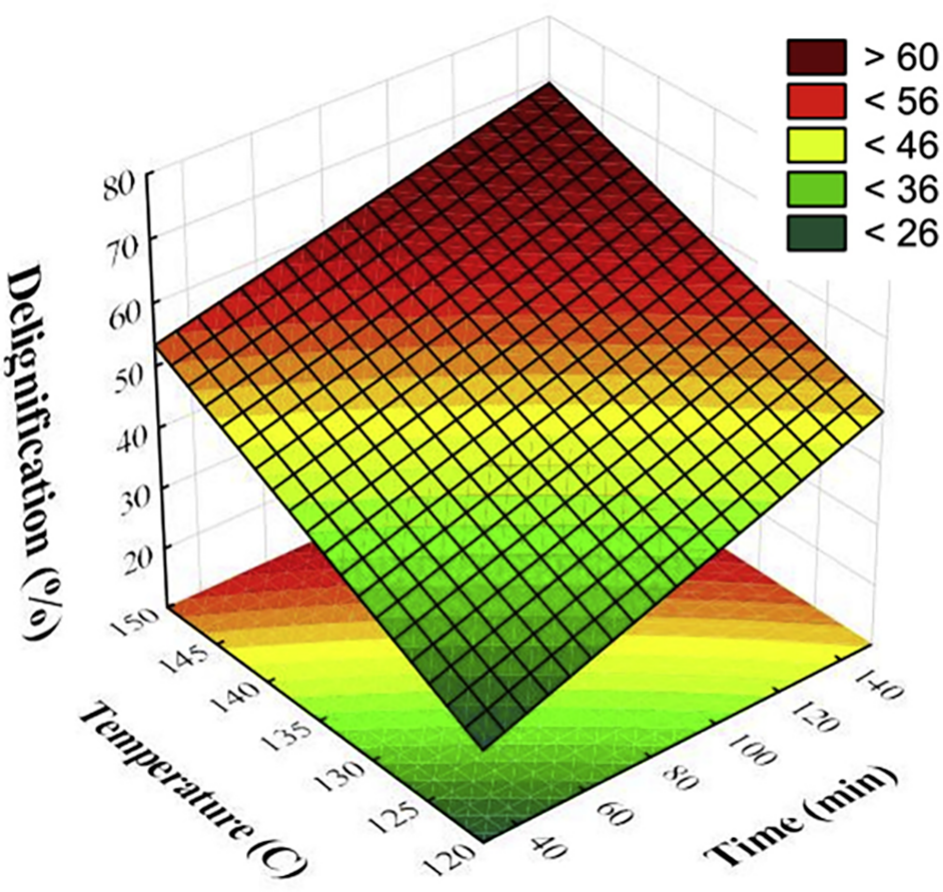
Response surface for the delignification of sugarcane bagasse in time–temperature experiments with the PIL mono-ethanol ammonium acetate, [(OH)^2^C_2_N]­[C_1_CO_2_], for a temperature range of 120–150 °C at residence times ranging from 30 to 150 min. Adapted with permission from ref 164. Copyright 2022 Elsevier Ltd.

Additionally, the concept of pretreatment severity, introduced as a function of temperature, residence time and acidity, can be used to compare and predict the performance of pretreatments that use different conditions.^464^ The concept of pretreatment severity factor (*R*_0_) was firstly introduced in 1987 for steam-aqueous-based pretreatments by Overend *et al*.^465^ Time (*t*) and temperature (*T*) were combined into a single factor to express the reaction ordinate, the *P*-factor, which was later renamed *R*_0_. Hence, *R*_0_ can be used as a simplified approach to predict the simultaneous effects of temperature and time on the pretreatment performance.^464^ Assuming a constant acidity, *R*_0_ can be calculated according to the following equation:

$$R_{0}=t\cdot e^{\frac{T-T_{\mathrm{ref}}}{\omega }}$$ 1

where *T* is the temperature of the reaction medium in °C, *T*_ref_ is a reference temperature in °C, *t* is the time in minutes, and ω is a parameter expressing the effects of temperature in the specific reaction considered. ω is related to a pseudo-activation energy and its value should be evaluated for each biomass fraction (activation energies for cellulose hydrolysis range between 87 and 179 kJ·mol^‑1^, for hemicellulose hydrolysis around ∼130 kJ·mol^‑1^, and for delignification between 89 and 131 kJ·mol^‑1^).^464^ Typical values used to evaluate the severity factor during biomass fractionation are *T*_ref_ = 100 °C and ω = 14.75. This value of ω represents a rate of reaction that doubles for every 10 °C increase above the *T*_ref_. Hence, according this approximation, to keep the severity factor, residence time needs to be halved by every 10 °C increase in temperature.^466,464^ Since temperature profiles are not isothermal, severity factors can be calculated by integrating the temperature profiles over time:

$$R_{0}=\int_{0}^{\mathrm{time}}e^{\frac{T-T_{\mathrm{ref}}}{\omega }}$$ 2

Which is analogous to the H-factor equation developed for delignification in Kraft pulping:

$$H=\int_{0}^{\mathrm{time}}e^{43.2-\frac{16115}{T\;\left(K\right)}}$$ 3

For example, the pretreatment of the hardwood *Eucalyptus* with the IL, delignification shows maximum values in the range of *R*_0_ 1300–6900 and H 6900–16900 (Figure 22). Below this range, the lignin removal is insufficient and above, the condensation reactions and pseudo-lignin formation increases the lignin content in IL pretreated pulps.^464^

**Figure 22:**
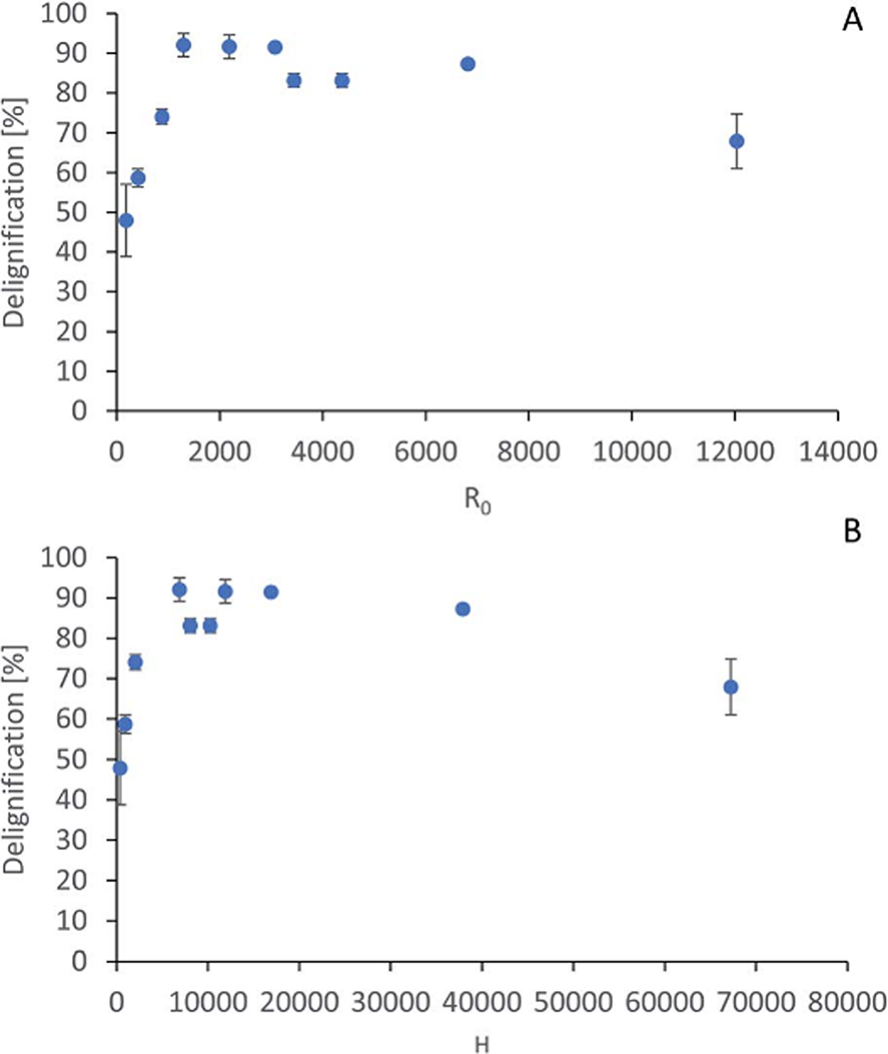
Predictive models for lignin removal from *Eucalyptus red grandis* with [C_2_C_2_C_2_N]­[HSO_4_]_80%_. (A) Lignin removal as a function of *R*_0_. (B) Lignin removal as a function of H-factor. Adapted with permission from ref 464. Copyright 2020 Royal Society of Chemistry under CC BY 4.0 (https://creativecommons.org/licenses/by/4.0/).

Further development of this concept was made considering the role that the acidity of the medium has on biomass pretreatment. A factor that considers proton concentration in the pretreatment medium, the combined severity factor *R*_0_* was developed, initially for acid-catalyzed Organosolv pretreatments.^467^

$$R_{0}^{*}=R_{0}\cdot \left[H\right]$$ 4

Its logarithmic expression can be used to evaluate and predict the performance of different pretreatment processes:

$$\log\;R_{0}^{*}=\log\left(R_{0}\cdot \left[H^{+}\right]\right)=\log\;R_{0}-\mathrm{pH}$$ 5

However, pH measurements cannot be used for ILs, hence, expressing the pretreatment severity using this expression is not possible. Instead of pH, *H*_0_, an extension of the pH logarithmic scale to measure Brønsted–Lowry acidity beyond dilute aqueous solutions, can be used to determine the acidity of ILs. *H*_0_ measures the degree of protonation of an indicator (In) such as *p*-nitroaniline in solution, according to the following expression:

$$H_{0}=pK_{a}\left(\mathrm{In}\;nH^{+}\right)+\log\left(\frac{\left[\mathrm{In}\right]}{\left[\mathrm{In}\;H^{+}\right]}\right)$$ 6

where p*K*_a_(In H^+^) is the p*K*_a_ value of the protonated indicator in aqueous solution, while [In] and [In H^+^] are the molar concentrations of its deprotonated and protonated forms in the IL.^468^ *H*_0_ can be calculated for ILs at certain water concentrations, and there are a few studies that do so.^468,469^ Then, the H_0_ function of the IL solutions can be incorporated into the combined severity factor logarithmic expression (log *R*_0_*), resulting in a modified logarithmic expression for the severity factor:

$$\log\;R_{0}^{*}=\log\;R_{0}-H_{0}$$ 7

The use of the logarithm values of the classical and modified severity factors *R*_0_ and *R*_0_* make it easier to compare the resultant numerical values. Furthermore, delignification data has shown better fitting when plotted against log *R*_0_* than log *R*_0_, which highlights the importance of considering the IL acidity (Figure 23).^468^ The introduction of H_0_ reflects the improved sensitivity of the delignification to the acidity of the pretreatment medium.

**Figure 23:**
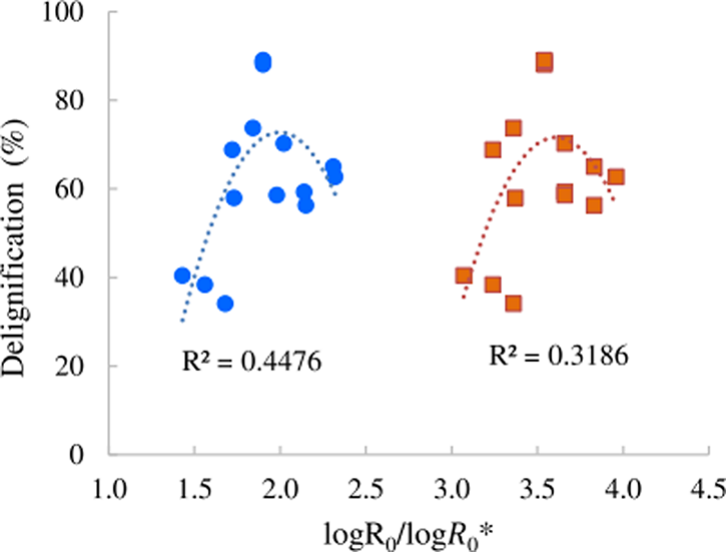
Fit of the classical severity factor log *R*_0_ (in orange, right) and the modified severity factor log *R*_0_* (in blue, left) with delignification, expressed as quadratic fits considering the results obtained from a BBD-RSM analysis where delignification showed a steep curvature. Adapted with permission from ref 468. Copyright 2023 American Chemical Society under CC BY 4.0 (https://creativecommons.org/licenses/by/4.0/).

##### 2.8.3.2 Influence of Time

Time course experiments have shown that there is a strong correlation between delignification, precipitated lignin yields, and saccharification yields (Figures 24 and 25).^340,464,468^ The positive correlation between lignin extraction and glucose yield can be related to the higher surface area, therefore, exposure of cellulose substrate, allowing easier access of enzymes. However, very high saccharification values have been obtained from certain pretreatment conditions that do not yield maximized delignification. This suggests that other factors, as physicochemical properties of the residual lignin on the cellulose pulp (*e.g.*, degree of condensation, impacted by the pretreatment severity) also play a role.^468^

**Figure 24:**
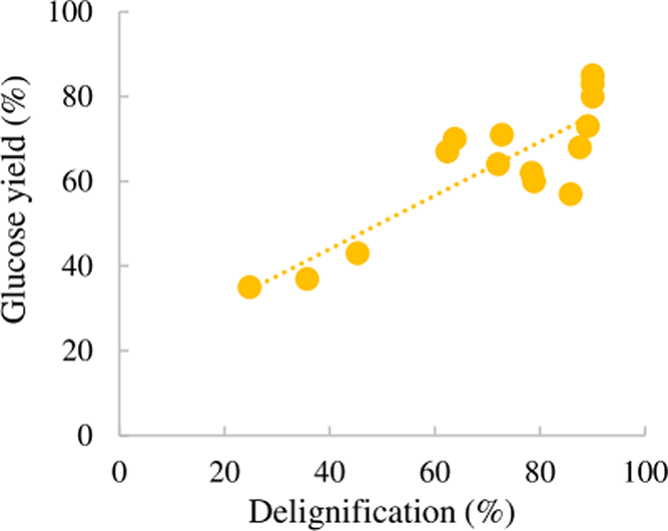
Correlation between glucose yield and delignification enzymatic hydrolysis was conducted for 72 h and pretreatment conditions (160–180 °C, 20–40 min, 10–30 wt% of water). Adapted with permission from ref 468. Copyright 2023 American Chemical Society under CC BY 4.0 (https://creativecommons.org/licenses/by/4.0/).

**Figure 25:**
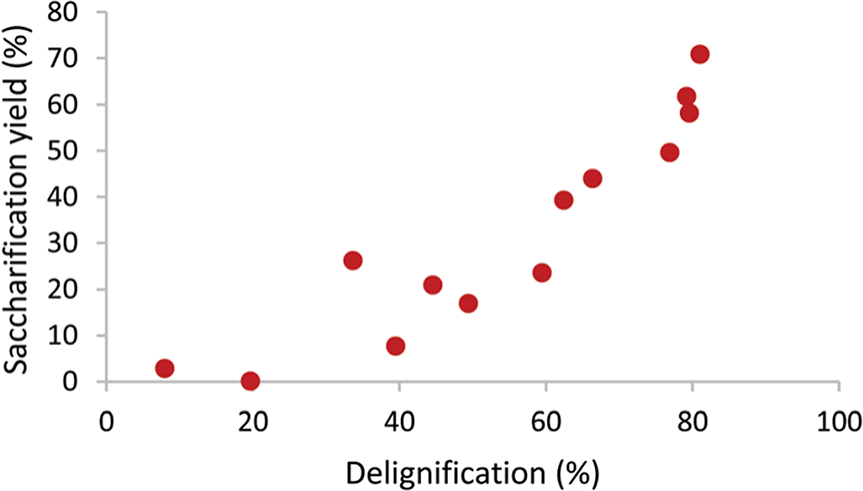
Correlation of enzymatic glucose release and delignification of *Pinus sylvestris* after pretreatment with [C_4_im]­[HSO_4_]_80%_ at different temperatures and times with a solid loading of 10 wt%. Adapted with permission from ref 340. Copyright 2019 Royal Society of Chemistry.

Furthermore, lignin yields reach maximum values at times above the maximum delignification due to condensation of lignin fragments in the IL, which leads to the formation of oligomers with higher weight averaged molecular weight (*M*_w_) and lower solubility. The formation of lignin-like polymers from sugar degradation products, referred to as humins or pseudo-lignin, can also contribute to precipitate yields.^8^ At long pretreatment times, the redeposition of recondensed lignin fragments, pseudo lignin and/or humins on the pulp surface leads to decreasing delignification and lignin yields.^174^ It should be noted that the standard work up procedure followed by studies conducted at lab scale to facilitate the separation of the pulp from the IL is based on the addition of ethanol to the IL after the pretreatment is complete. This can induce lignin precipitation over the pulps, which would be particularly noticeable at longer pretreatment times due to the presence of longer, condensed polymers. However, this is not expected to be implemented at industrial scale, where pulp washing with hot fresh IL is favoured and could help mitigating this undesired effect.^340^

Different studies with a variety of PILs ([C_4_im]​[HSO_4_]_80%_, [C_2_C_2_C_2_N]​[HSO_4_]_80%,_ [C_4_C_1_C_1_N]​[HSO_4_]_80%_) have reported that the composition of lignins recovered from pretreatment of wide range of biomass (*e.g.*, *Miscanthus*, switchgrass, willow, pine, rice husk, *Eucalyptus*, waste woods, poplar, etc.) at different time points (from 15 min up to 24 h) differ significantly. These observations show a consistent behaviour of lignin dissolution and reactivity over the course of pretreatments. General changes over time included the removal of carbohydrates, decrease in S units (where present), PCA and FA contents and increase in H and G unit content.^464,174,83,353,341,339^ According to these observations, a 4-stage model has been proposed to explain the lignin fate during the course of pretreatments of biomass with ILs (Figure 26):^350^

**Figure 26:**
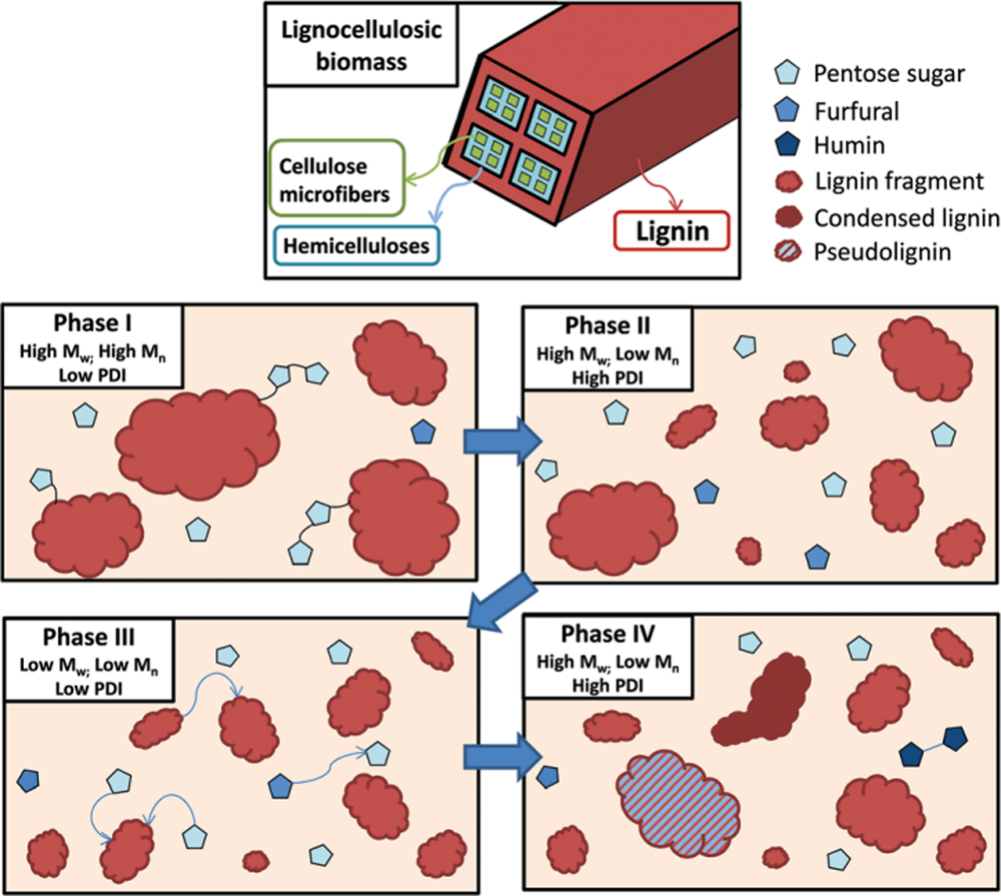
Four-stage model for lignin extraction during ionoSolv-type pretreatments. Adapted with permission from ref 350. Copyright 2018 Royal Society of Chemistry under CC BY 4.0 (https://creativecommons.org/licenses/by/4.0/).

- Phase I: Early extraction and precipitation. Lignin isolated at this stage is mostly uncondensed and yields are low, since only a small proportion of lignin is extracted initially. PDI of lignin obtained during this phase is low, and their number averaged molecular weight (*M*_n_) and *M*_w_ are consistently lower than those of ball milled and OrganoSolv lignin. This suggests that a certain degree of cleavage inside the lignocellulose matrix is needed before lignin can be solubilized in the IL.^174,83^ However, the *M*_n_ and *M*_w_ are higher than in later phases, showing that these fragments still contain a significant number of cleavable ether linkages. Indeed, these initial fractions preserve β-O-4′, β–β, and β-5 linkages and have high S/G ratios so their composition is similar to that of ball milled lignin. Also, significant amounts of PCA and arabinose are commonly detected, suggesting the preservation of LCC linkages at early stages of pretreatment and suggests a concerted extraction mechanism involving hemicellulose and lignin.^83^
- Phase II: Extraction of lignin fragments from the biomass continues and overlaps with the hydrolysis and depolymerization of the already extracted fragments. This gives a decrease of *M*_n_ while *M*_w_ remains mostly unchanged, which combined results in an increase of the PDI. Lignin yield remains relatively low due to the abundance of small fragments that do not precipitate well. The intensities of the peaks of β-O-4′, β–β′ and β-5′ linkages decrease over time.^1^ However, only β-O-4′ linkages are largely hydrolyzed. For β–β and β-5 bonds, the decreased intensity is more likely related to chemical modifications rather than bond breakage, since C–C bonds are more difficult to break under pretreatment conditions and evidence of hydrolysis and substitution of the five-membered tetrahydrofuran (THF) rings of those structures have been found.^174,83^ PCA content also decreases over time, while H units content increases due to PCA conversion into H type structures. Gradual disappearance of carbohydrate signals suggests that, although lignin solubilizes into the IL attached to carbohydrate moieties, LCC linkages are then gradually hydrolyzed in the solution.
- Phase III: lignin extraction from the biomass stops and lignin yield reaches its maximum. Most of the extracted lignin fragments have been broken down to smaller fragments. This is reflected in a further decrease of both *M*_n_ and *M*_w_, which also gives a lower PDI. The content of condensed structures increases rapidly.^464,350^

- Phase IV: Lignin hydrolysis ceases and there is a high rate of recondensation of lignin fragments, which may include carbohydrate degradation products. Big, recondensed polymers co-exist with small, unreactive lignin fragments, increasing *M*_w_ while the *M*_n_ remains unaffected, resulting in higher PDI. Eventually, the rate of recondensation might surpass the rate of depolymerization, leading to an increase in *M*_n_, *M*_w_, and PDI. Lignin yield decreases from its maximum in phase III, as recondensed polymers too large to remain soluble re-precipitate onto the surface of the pulp, compromising their further valorization.

It has been observed that lignin recovery can eventually exceed delignification, suggesting that non-lignin components, likely carbohydrate degradation products, can participate in lignin recondensation reactions and become incorporated into the polymers.^350^ This is supported by reports of unexpected increases in the G5 signal intensity, ascribed to carbohydrate-derived degradation products.^340^ It has been proposed that fast extraction of lignin from the cellulose pulp is particularly beneficial for softwood delignification, since its guaiacyl-rich lignin is particularly prone to condensation reactions under pretreatment conditions.

Although S-rich lignin is easier to extract because it is less cross-linked than G-rich lignin, it has been reported that for hardwood pretreatments (*e.g.*, *Eucalyptus* and willow) relatively G-rich lignins are isolated at short pretreatment times, with a gradual increase in S/G ratios over time until they start to level off or decrease. This can be explained by the fact that ethers in the α-position of G-type units break faster than ethers linked to S-type units. Therefore, a low initial S/G ratio can be achieved due to the faster ether cleavage of G units, leading to a quick solubilization in the IL. Then, the S/G ratio increases as most of the lignin is extracted, resulting in an S/G ratio similar to that of the native lignin. Then, the S-derived condensation products start degrading, leading to a more G-rich lignin.^464,341^

##### 2.8.3.3 Influence of Temperature

Biomass fractionation with ILs requires relatively high temperatures. Increased temperature reduces IL viscosity, which helps with diffusion and mass transfer rates and can promote destabilization of the H bonding between ILs and biomass. It has been suggested that lignin extraction is more favorable if its glass transition temperature (which ranges between 130 and 150 °C) is surpassed.^341^ This is particularly important for more recalcitrant feedstocks that contain higher amounts of lignin that is more difficult to remove and more prone to recondensation reactions, as softwoods.

However, extended pretreatment times at elevated temperatures can lead to overtreatment of the biomass. Overtreating conditions can be identified by increase in pulp recovery and decrease in lignin recovery, indicators of lignin re-deposition onto the pulp. Increasing the temperature can accelerate lignin extraction but also promotes lignin recondensation reactions, leading to reprecipitation onto the pulp surface. This has a negative impact on its further valorization, and the optimal point needs to be carefully balanced.^341,350^ High temperatures and prolonged pretreatment times can also lead to the formation of carbohydrate degradation products (*e.g.*, furfural, HMF, acetic or formic acid), mainly originating from hemicellulose sugars, which can react with lignin molecules forming polymers that resemble lignin (pseudo-lignin and humins). When they become insoluble in the reaction media, they can precipitate onto the surface of the recovered pulps and are detected as acid-insoluble lignin when running composition analysis of those pulps.^470^

Studies comparing pretreatments with PILs at different temperatures have found that the minimum lignin content is usually achieved at the highest temperature, suggesting that higher temperatures facilitate extraction of lignin more than they accelerate lignin condensation reactions. Operating above the glass transition temperature (*T*_g_) of lignin could be one of the factors playing a role.^462^ The comparison of the lignin fractions recovered at different temperatures and time points showed that the samples pretreated at higher temperatures changed their structure more rapidly. Temperature had a bigger effect than residence time in the reduction of β-O-4′, β–β′, and β-5′ linkage content and S and G uncondensed units and PCA and in the increase in H units and G condensed units. The authors claim that the benefits of increasing the temperature of pretreatment overcome those of prolonged reaction times, improving delignification and lignin depolymerization at higher rates than the increase in degradation and recondensation reactions. Also, that it is possible to adjust pretreatment conditions to favor both lignin and cellulose valorization. Another interesting finding was that the lignin recovered at overtreated conditions had a significant higher amount of phenolic −OH content that all the other samples, suggesting this could be used as an indicator of overtreatment when optimizing pretreatment conditions.^350^ On the other hand, comparison of the lignin samples isolated from pine with [C_4_C_1_C_1_im]­[HSO_4_]_80%_ at the optimum pretreatment times at different temperatures (0.5 h at 170 °C, 1 h at 150 °C, and 4 h at 120 °C) showed that this lignin had a similar degrees of condensation and quantities of unhydrolyzed ether linkages, even when lignin and saccharification yields were substantially different. This could be due to the different composition of grass and softwood lignins.

Temperature control of the pretreatment can be used to alter the lignin composition to favor certain structures. For example, the cleavage of the methyl ketone group from vanillin at high temperature leads to guaiacol formation. Hence, lowering the process temperatures to 120 °C allows generating more vanillin.^471^

Three-level Box–Behnken design combined with response surface modelling (BBD-RSM) using the key pretreatment parameters: residence time, temperature, and IL concentration can be used to predict the delignification response for a given IL and feedstock. The resulting 3D response surfaces and contour plots can be used to visualize the synergetic effect of each variable. For example, Abouelela *et al.* used this approach to evaluate the effect of pretreatment severity on the delignification of pine during pretreatments with the PIL [C_4_C_1_C_1_N]­[HSO_4_] (Figure 27).^468^ In this particular case the region where high delignification is achieved is small, suggesting a high sensitivity to the experiment variables. This agrees with previous findings suggesting that optimal delignification can only be achieved in a narrow window in acidic PILs; with lower severity conditions being inefficient to remove enough lignin and higher severity conditions leading to recondensation reactions, formation of pseudo lignin and humins and reprecipitation of polymers onto the pulp surface.

**Figure 27:**
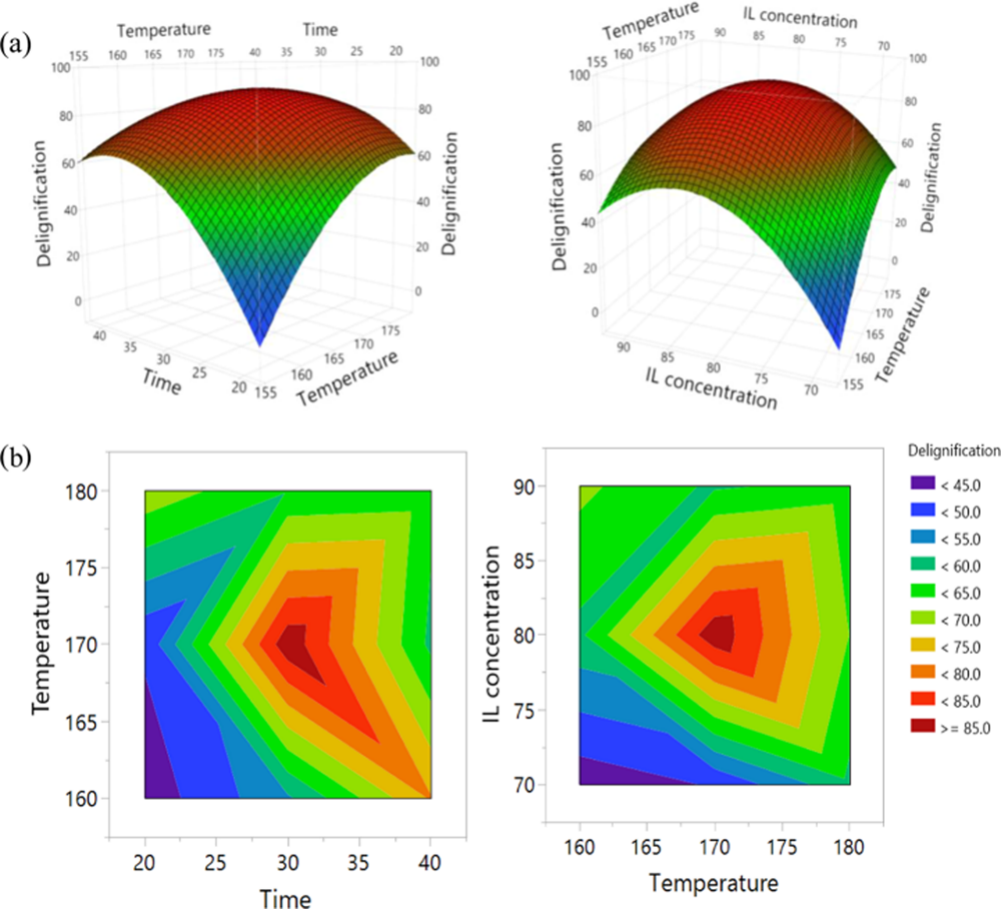
(a) BBD-RSM response surface graphs and the (b) corresponding counter plots at the center point (left, IL concentration = 80 wt%; right, time = 30 min) for the pretreatment of pine with [C_4_C_1_C_1_N]­[HSO_4_]. Adapted with permission from ref 468. Copyright 2023 American Chemical Society under CC BY 4.0 (https://creativecommons.org/licenses/by/4.0/).

In this example, the best delignification that could be achieved for pine softwood with [C_4_C_1_C_1_N]­[HSO_4_] would be of ∼88%, using a temperature of 170 °C, a residence time of 30 min, and a water content of 20 wt%. Decreasing water content to 10 wt% and residence time to 20 min or increasing the temperature to 180 °C with a residence time to 20 min also would allow keeping high delignification performance (>70%).^6^ The 30% residual lignin remaining on the pulps can correspond with both non-extracted native lignin and the redeposited humins and pseudolignin.

##### 2.8.3.4 Influence of Acidity

The severity of the pretreatment can be adjusted by controlling the acidity of the pretreatment medium. The choice of ILs based on their alkaline, neutral, or acidic character is a powerful tool. For pretreatments using acidic PILs the acidity of the medium can be controlled with precision by adjusting the acid–base ratio of the IL. Further tuning of the acidity of the pretreatment media can be also achieved by adjusting its water content. For example, mixtures of [C_4_C_1_C_1_N]­[HSO_4_] with water show the lower acidity with 20–30 wt% water content (Figure 28).^468^ Higher water concentrations increased proton transfer ability of the medium, while lower concentrations increase the acidity due to lower solvation of the IL ions by water molecules. The hydration limit for this IL with water is around 20% water content, which corresponds with 75 mol%, and falls within the lower acidity range. As mentioned previously, pH values cannot be used for ILs, and _0_ is used instead to determine the acidity of ILs. Lower *H*_0_ values are associated with more acidic solutions, due to the higher tendency to donate protons.^300^

**Figure 28:**
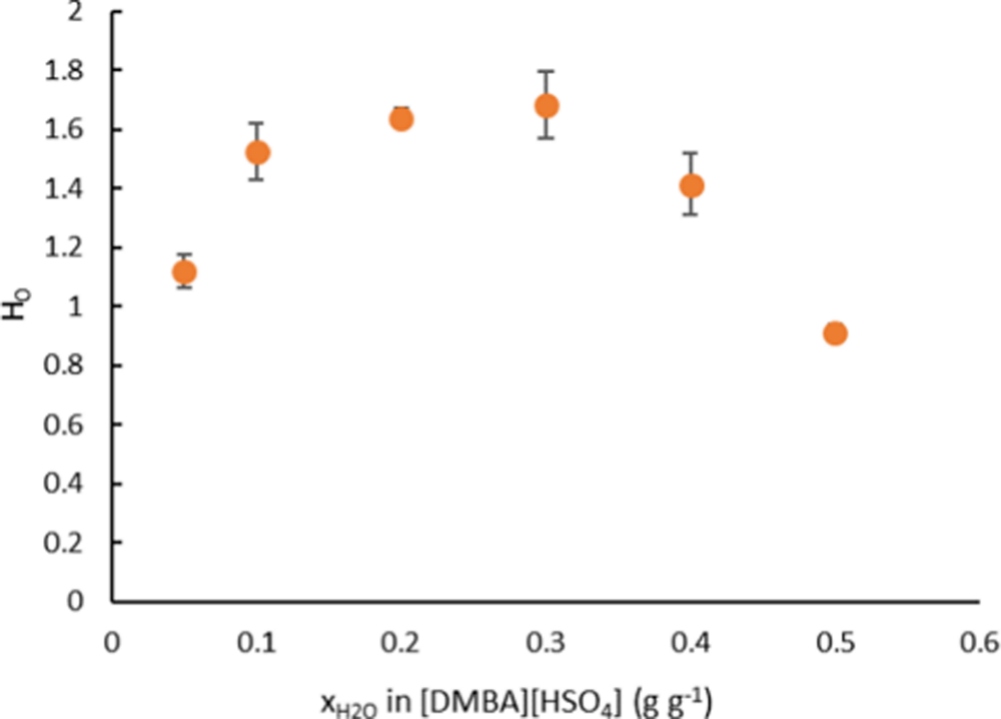
H_0_ values for mixtures of [C_4_C_1_im]­[HSO_4_] with water at different water concentrations. Adapted with permission from ref 468. Copyright 2023 American Chemical Society under CC BY 4.0 (https://creativecommons.org/licenses/by/4.0/).

2.8.3.4.1. *ILs of Different Acidity*. Lignin recovery from alkaline ILs such as [Ch]­[Lys] or [C_2_C_1_im]­[AcO] has been reported as significantly lower than the biomass delignification during the same process. Strong hydrogen bonding between lignin and alkaline ILs blocks lignin reprecipitation. Therefore, successful lignin recovery from these IL needed acidification with a HCl solution, which has a negative implication for the recycling of the IL.^336,329^

For PILs, differences in acidity of the IL leads to different behavior during delignification and in the properties of the recovered lignins. For example, when two PILs of different acidity [C_4_C_1_C_1_N]­[HSO_4_] and [C_1_im]Cl (pH of a 1% aqueous solution = 1.2 and 4.2, respectively) were compared for the delignification of post-consumer waste wood at 170 °C, the more acidic [C_4_C_1_C_1_N]­[HSO_4_] reached an optimum delignification point (53% after 45 min) before dropping down (35% after 80 min), while the delignification with the less acidic [C_1_im]Cl remained fairly constant at ∼50% even under high pretreatment severity conditions (up to 170 °C for 80 min or 150 °C for 150 min).^462^ Furthermore, the lignin recovered with [C_4_C_1_C_1_N]­[HSO_4_] had a much higher degree of condensation, almost doubling that of the lignin recovered with [C_1_im]Cl and less degree of preservation of the β-O-4′ ether linkages, signs of higher cleavage, and overall pretreatment severity. This shows that the increased acidity of the IL promotes recondensation reactions and reprecipitation of the extracted lignin fragments on the cellulose pulp, leading to a decrease in the delignification value. In this case, the less acidic IL, [C_1_im]­Cl, was effective over a wider range of severity conditions which can be advantageous.

2.8.3.4.2. *Different a:b Ratios*. Comparison of pretreatments with the same PIL synthesized with different acid base ratios also led to similar findings. For example, pretreatments of willow with two batches of the PIL [C_2_C_2_C_2_N]­[HSO_4_]_80%_, the first prepared with 2% excess acid (acid base ratio a:b = 1.02) and the second with 2% excess base (a:b = 0.98) showed that under mild pretreatment conditions (*e.g.*, pretreatments at 120 °C and short pretreatment times at 150 °C and 170 °C) the more acidic solution (a:b = 1.02) achieved higher lignin recoveries. However, for longer pretreatment and high temperatures (150 and 170 °C), lignin yields were higher for the less acidic solution (a:b = 0.98). This again suggests that higher IL acidity accelerates the occurrence of recondensation reactions, leading to more condensed lignin fractions with higher molecular weights and, hence, redeposition of pseudo lignin onto the pulp.^472^ The more acidic IL (a:b = 1.02) was also more effective breaking the β-O-4′ linkages, while the more basic IL (a:b = 0.98) allowed the preservation of these linkages even at high temperatures and prolonged reaction times. Under the same pretreatment conditions, the amount of β-O-4′ linkages detected from lignins recovered from the more basic IL doubled the amount detected for the more acidic IL.^341^ Similar findings have been confirmed with other PILs (*e.g.*, [C_2_C_2_C_2_N]­[HSO_4_]) and feedstocks (*e.g.*, *Miscanthus*).^174,339^ The S/G ratio of the recovered lignins were also influenced by the acidity of the IL. Lignins isolated with the acidic IL showed higher S/G ratios, which were stable at most severity conditions. Only pretreatments at 170 °C for more than 30 min showed a decrease. It has been suggested that this might imply that S-rich lignin is more easily extracted under milder reaction conditions because it is less cross-linked. Only more severe conditions, starting at 40 min at 170 °C with an excess acid content in the IL (a:b = 1.02) is also capable of extracting the G-rich fraction of lignin, leading to a decrease in the S/G ratio. For the more basic IL (a:b = 0.98), the S/G ratio increases gradually up to the maximum value, suggesting that the lower acidity affects the kinetics of the extraction of S-units.

Regarding acetate-based ILs,^178^ studied the impact of varying acid-base ratios of [(HO)^2^C_2_N]­[C_1_CO_2_] (from 0.1 to 10) on the lignin extraction, lignin recovery, solvent recovery, and enzymatic saccharification yield. They found that the lowest acid-base ratios, 0.1, led to increased lignin extraction and glucose release during enzymatic saccharification, with up to 84% and 96%, respectively, after 72 h of saccharification. Similarly, to the Brønsted acidic ILs (section 2.1.2), a higher acid content led to increased hemicellulose extraction into the liquid phase but with reduced IL recovery due to the volatility of acetic acid.

Different studies have also reported improved delignification ability for DESs aided with different acids (*e.g.*, AlCl_3_, FeCl_3_, and CuCl_2_, *p*-toluenesulfonic acid, or silico-tungstic acid). The added acids provide active protons and acidic sites that facilitate proton-catalyzed bond cleavage in lignin.^297,25^ Finally, it should be noted that the acidity of the pretreatment media also plays a big role in the degradation of the hemicellulose sugars and the formation of humins, which has a significant effect on the properties of the precipitated lignin. Attempts to correlate the formation of humins with the modified severity factor have been unsuccessful to date. This suggests that lignin extraction, depolymerization and recovery can be accelerated by the presence of acid. However, the consequent acceleration of pseudo lignin formation and condensation reactions requires careful control of reaction conditions.

##### 2.8.3.5 Effect of Catalysts

Catalysts can also be added to the solvent media during pretreatment of biomass with ILs and DESs to further improve delignification and/or to promote specific reactivity during pretreatment. For example, vanadium polyoxometalates (POMs) have been used to oxidize lignin extracted from biomass during pretreatment in one-pot processes. The use of POMs with a current of O_2_ has shown high efficiency in catalyzing the delignification of pine in different ILs, including [C_2_C_1_im]­[C_1_CO_2_] and [C_4_C_1_C_1_N]­[HSO_4_], allowing the separation of hemicellulose and simultaneous oxidation of lignin.^473,474^ An added benefit for the pretreatment in [C_2_C_1_im]­[C_1_CO_2_] is that extraction with benzene and THF allowed recovery of oxidized lignin derived aromatics from the IL liquor, facilitating its separation from the other biomass components, a challenging task for this type of pretreatment.^473^ In this case, methyl vanillate was the main lignin oxidation product, other oxidized aromatics recovered included acetovanillone, vanillic acid, methyl 3-(3-methoxy-4-hydroxyphenyl) propionate and methyl 4-hydroxybenzoate. Pretreatments of willow and pine with [C_4_C_1_C_1_N]­[HSO_4_] aided with a vanadium based POM using O_2_ or H_2_O_2_ as oxidizing agents, under oxygen rich conditions allow one to obtain specific products from lignin. Vanillin and syringaldehyde were the main products, and their relative distributions reflected the S:G ratio on the original samples. For pine, lacking S units, vanillin was the only recovered aldehyde. The presence of POM not only increased vanillin yields up to 20-fold, but prevented further oxidation into vanillic acid, as observed in the absence of the catalyst.^474^

##### 2.8.3.6 Effect of Water

Other important parameters that need to be considered in the optimization of pretreatment are those related to mass transfer properties, such as solids loading and water content. The influence of water content is not easily predictable and is highly dependent on the IL of choice and its pretreatment mechanism.^164^ Results from Shi *et al*. on the pretreatment of switchgrass with mixtures of [C_2_C_1_im]­[C_1_CO_2_] and different amounts of water confirmed these findings.^475^ They reported that increasing water content in [C_2_C_1_im]­[C_1_CO_2_] leads to decreases in both β and π and while increasing the *α*, which was reflected in the crystallinity index (being the lowest for the pulps recovered from the pure IL, 16%, and increased to 31% for the pulps recovered with the mixture IL:water = 80:20), cellulose digestibility, xylan content (which increased from 8.7% for the pure IL to a 17.8% for the IL:water = 50:50 mixture), and lignin content (which increased from 13.7% for the pure IL up to 21.3% for the mixture IL:water = 20:80). Molecular dynamics simulations found that concentrations of water above 50% enhance the interaction between water and the IL, reducing the interactions between the IL and the cellulose.

The influence of the IL type and its water content on the pretreatment mechanism has been highlighted by studies comparing pretreatment of various feedstocks with different IL families and water contents.^329,81^ For example, pretreatment with the AIL [C_2_C_1_im]­[C_1_CO_2_] at low water contents (0–10 wt%) allows for high saccharification yields without reducing the hemicellulose and lignin content of the recovered pulps to any significant extent. Only when water content increased to 20% pretreatment with [C_2_C_1_im]­[C_1_CO_2_] some degree of lignin and hemicellulose removal was achieved (30% and 61%, respectively). Furthermore, due to the strong hydrogen bonding character of the acetate anion, reprecipitation of lignin upon the addition of water was hindered. This shows that acetate based AILs are not efficient for separating a valuable lignin fraction unless the water content is high enough to change the pretreatment mechanism. On the other hand, the PIL [C_2_C_1_im]­[HSO_4_] achieved effective lignin and hemicellulose dissolution at all the investigated water contents, leaving a solid cellulosic pulp with preserved crystallinity and producing a recoverable lignin that precipitates upon water addition to the pretreatment liquor. The presence of water affects the hydrogen bonding interactions between the IL and cellulose, ultimately maintaining a high degree of crystallinity (Figure 29).

**Figure 29:**
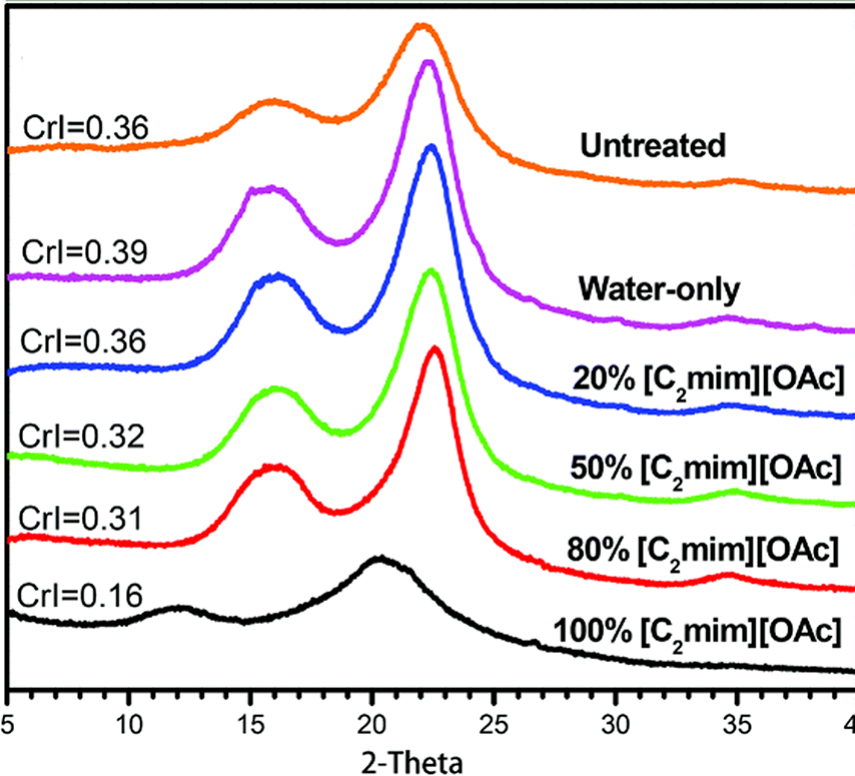
CrI of pulps recovered after pretreatment of switchgrass with [C_2_C_1_im]­[C_1_CO_2_] and increasing water contents. Adapted with permission from ref 475. Copyright 2014 Royal Society of Chemistry.

In the case of PILs, different effects have been observed depending on the feedstock employed. Abouelela *et al.* compared the delignification of various feedstocks (*Miscanthus*, pine, treated timber, and waste wood) using mixtures of [C_4_C_1_C_1_N]­[HSO_4_] and water at water concentrations between 5 and 50 wt% (at 170 °C for 30 min with solid loadings of 1:5 g g^–1^). Delignification for *Miscanthus* and waste wood remained fairly constant at all water concentrations but was higher for *Miscanthus* (∼86%) than for waste wood (∼44%), likely due to the complex composition and the level of contamination of the latter, which consists of a mixture of different woods, engineered wood, sand, etc. (Figure 30). On the other hand, delignification from pine wood (*Pinus sylvestris*) and treated timber (both softwoods) decreased significantly when increasing the water concentration, with the highest delignification at the lowest water content (5 wt%).^476^ This can be attributed to the higher recalcitrance of softwoods, and their lignin composition, mostly formed by G units but also differences in the composition of hemicellulose and LCCs. Kinetic studies have shown that the cleavage of the β-O-4′ linkages of softwoods require a dehydration reaction that is inhibited by excess quantities of water.^476^ Increasing water concentrations decreases reaction rate for the hydrolysis step of the dehydration reaction of lignin in ILs as [C_4_C_1_im]­[HSO_4_], suggesting that water is implicated in the reaction prior to the rate-determining step and has a competing effect beyond its role in proton transport. The lack of direct correlation between H_0_ and rate constant implies water has multiple roles, by influencing acidity but also in impeding substrate reactivity. A mechanism that involves an initial protonation of the substrate followed by a dehydration step where the presence of high quantities of water slows the reaction progress has been proposed. This is then followed by the hydrolysis of the intermediate in the presence of water.^344^

**Figure 30:**
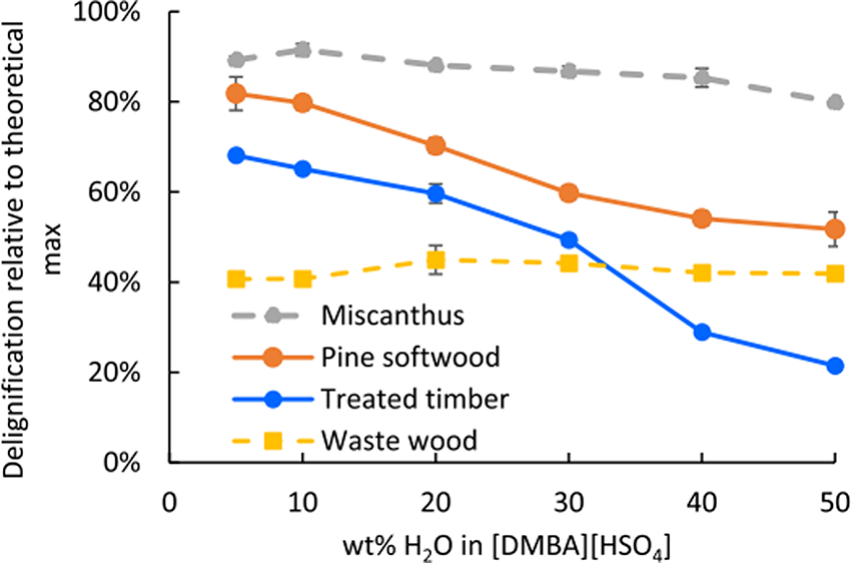
Delignification degree for four different feedstocks (*Miscanthus*, *Pinus sylvestris*, treated timber and waste wood) pretreated with [C_4_C_1_C_1_N]­[HSO_4_] containing different water concentrations at 170 °C for 30 min with a solid loading of 1:5 g·g^−1^. Adapted with permission from ref 476. Copyright 2021 American Chemical Society.

Delignification of a hardwood (poplar) with the IL [C_2_C_1_im]­[HSO_4_] with varying water contents between 0 and 40 wt% also remained constant. However, the precipitated lignin yields were nearly four times larger at 15 wt% water content than at 0 wt% water, suggesting that the water content also has a significant effect on lignin separation from the IL solution.^329^ The nature of this effect is unclear, but it has been suggested factors affecting the chemistry of lignin extraction and conversion such as availability of water and solvent acidity could be involved. For example, the anion of the IL could react with −OH groups forming sulfate esters at very low water contents, making the extracted lignin more water-soluble. At higher water contents, the extraction of more water-soluble lignin fragments could be favoured while more hydrophobic fragments would remain within the biomass matrix, resulting in lower precipitation yields upon water addition. The formation of water-insoluble humins could also be playing a role.

##### 2.8.3.7 Effect of Solids Loading

Studies conducted on the effect of biomass loading during pretreatment with the PILs [C_2_C_2_C_2_N]­[HSO_4_] and [C_4_im]­[HSO_4_] with grassy and softwood biomass feedstocks (*Miscanthus* and pine) found that increasing solid loadings (ranges between 5 and 50 wt% were investigated) led to apparent increases in ether cleavage and condensation degree and apparent decreases in *M*_n_, *M*_w_, and PDI of the recovered lignin fractions. Higher degree of lignin reprecipitation onto the pulp surface and a slight increase in the pseudolignin content were also reported.^182^ Although these observations could be related to the higher concentration of lignin and hemicellulose in the liquor, it has also been reported that protic ILs can dissolve up to 70 wt% lignin, which makes saturation unlikely. Instead, it has been proposed that these small differences could be related to the pulp washing processes, based on the addition of ethanol to dilute the IL and facilitate the separation of the pulp. Since lignin solubility in ethanol is lower than in the IL, its addition can promote the precipitation of the larger lignin fragments. This effect would be more pronounced at higher concentrations, causing lignin fragments with lower *M*_w_ to precipitate after addition of the same amount of methanol when more lignin is present in the liquor. These results suggest that lignin chemistry in the pretreatment media is not altered at higher loadings, but instead, the washing protocols need to be improved to either avoid lignin reprecipitation or to remove the reprecipitated lignin from the pulp surface. This has been supported by experiments comparing different washing solvents (*i.e.*, DMSO and ethanol). It should be noted that although pulp washing with ethanol or DMSO can be implemented easily for lab-scale operations, they are unlikely to be employed at industrial scale facilities. Alternative procedures (*e.g.*, washing stages with fresh IL) need to be investigated for scaled up operations.

##### 2.8.3.8 Effect of Recycling the IL

Recycling of IL is key for the viability of IL-based biorefineries. Even the synthesis of low-cost PILs is still higher than those of conventional acidic and basic compounds (such as sulfuric acid and sodium hydroxide). Therefore, efforts should also be made to simplify and enhance the efficacy of IL recovery procedures. In general, recovered ILs can be utilized multiple times without compromising the efficacy of pretreatment; therefore, it is essential to prevent recovery procedures from affecting the functionality of ILs.^477^

For this, the IL needs to be stable at the pretreatment conditions and, since biomass processing with ILs typically involves high temperatures, the thermal stability of the ILs must considered a key operational parameter. Many AILs employed biomass processing under dissolution conditions that are based on acetate anions present stability issues. For example, dealkylation reactions have been observed for [C_2_C_1_im]­[C_1_CO_2_] at temperatures as low as 120 °C, and anion decomposition has been reported after 4 cycles of pretreatment with [C_4_C_1_im]­[C_1_CO_2_] at 120 °C for 4 h.^168,478^ PILs based on the acetate anion also have stability issues due to incomplete protonation and displacement of the IL formation equilibrium towards the acid–base pair and subsequent volatilization of the amine.^81,168,349^ The stability of PILs is related to the Δp*K*_a_ between the forming acid and base. Values of Δp*K*_a_ > 10 have been reported as a necessity for the preparation of temperature stable PILs.^479,480^ Decomposition of different PILs based on acetate anion after biomass processing, such as [(OH)^2^C_2_N]­[C_1_CO_2_] or [Ch]­[C_1_CO_2_], has been reported.^81^

Recovery of BILs can be also problematic due to thermal stability. BILs based on AA-derived anions are highly alkaline (and hence dealkylate) but also inherently unstable; decarboxylation will set in around the pretreatment temperature, so long-term use is limited. On the other hand, it is an absolute calling card for the one-pot approach, as the lower temperature solves this problem. For example, decomposition of [Ch]­[Lys] after pretreatment at low severity conditions (120 °C for 6 h) has been reported.^81^ Furthermore, since lignin precipitation from [Ch]­[Lys] needs acidification with a HCl solution, the recycling of this IL needs a neutralization stage (usually with a solution of NaOH). This produces NaCl, which needs to be precipitated and filtered off after water evaporation and dissolution of the IL in ethanol. Further ethanol evaporation allows the recovery of the IL.^336^ The addition of the extra stages for IL recovery are a serious drawback for this process.

PILs based on hydrogen sulfate ammonium are more thermally stable due to the strong acidity of sulfuric acid and weaker nucleophilicity of the [HSO_4_] anion. Decomposition temperatures for alkylammonium hydrogen sulfate ILs have been reported ranging from 280 to 322 °C (*T*_peak_ of [C_2_C_1_im]­[C_1_CO_2_]: 215 °C), with higher *T*_peak_ for the less substituted cations. This type of PIL follow a different decomposition mechanism in which the main reaction is the dealkylation of the cation. The fact that mono- and dialkylated ammonium hydrogen sulfate are ILs also capable of fractionating biomass minimizes the potential issues derived from the presence of small amounts of dealkylated IL.^168^

Here, we are providing a table summarizing decomposition measurements found in bibliography for the ILs most employed for biomass deconstruction (Table 2). Thermal stability of ILs is commonly studied by TGA analysis. However, it should be noted that the values obtained in bibliography for a given IL can vary significantly, up to a 20%, due to different experimental conditions (*e.g.*, sample mass, heating rate, gas flow, or the type of sample pan); by the mass loss threshold chosen as decomposition temperature (*T*_dec_, usually one of these: 1%, 2%, 5%, or 10%) or by the parameter chosen as reference (*T*_dec_, *T*_start_, *T*_onset_, *T*_peak_).^481^ *T*_start_ is defined as the temperature at which the sample starts losing mass, *T*_onset_ is the intersection of the baseline weight with the tangent of the weight dependence on the temperature curve, and *T*_peak_ is the temperature at which the sample shows the highest degradation rate, obtained from the peak of the DTG curves (if there are more than 1 peak in DTG curves, the one selected is usually the more intense). Furthermore, some ILs, in particular protic ILs for which the forming equilibrium can revert to the forming species, can show mass loss events resulting not only from decomposition, but also from evaporation. For some of those, boiling temperatures are also given (*T*_boil_). *T*_dec_ values obtained in these cases are highly dependent on the heating rate. It should also be noted that ILs start decomposing at temperatures significantly lower than that measured as *T*_onset_. Also, that thermal degradation occurs at lower temperatures than that measured as *T*_start_ when subjected to elevated temperatures for prolonged periods of time. Therefore, it has been suggested that a *T*_x/z_ parameter, reflecting the temperature at which ILs suffer a *x*% mass loss over a *z* length of time, should be used instead for a better representation of thermal stability of ILs aimed for biomass processing. *T*_0.01/10_ (*x* = 1%, and *z* = 10 h) has been proposed as an optimal parameter for this purpose.^39,482,483^ However, there is insufficient data available in literature to give a comprehensive list of *T*_0.01/10_ values for a variety of relevant ILs. Nevertheless, it has been determined that for a wide range of ILs based on imidazolium, pyrrolidinium, and phosphonium ILs, *T*_0.01/10_ is usually around 110 °C lower than the measured *T*_onset_.^482^ For example, *T*_0.01/10_ for [C_4_C_1_im]­[C_1_CO_2_] has been measured as 102 °C and, although we could not found *T*_onset_ values for this particular IL, *T*_onset_ for [C_2_C_1_im]­[C_1_CO_2_], which shares a similar structure with the same anion and same cation type, has been reported ranging between 214 and 221 °C (Table 2).^39,481,483^ A final consideration to be made is that, usually, IL decomposition assessment experiments are carried out under inert atmosphere of N_2_ while biomass processing is performed at open atmosphere containing O_2_, which typically lowers the temperature of decomposition of the ILs.

**Table 2:** Decomposition Temperatures of Some of the Most Relevant ILs Employed in Biorefining and Discussed in This Review^a^

IL	*T*_boil_ (°C)	*T*_dec_ (°C)	*T*_start_ (°C)	*T*_onset_ (°C)	*T*_peak_ (°C)	ref
[C_1_C_1_im][(C_1_O)_2_PO_2_]		268^a^				^484^
[(C_1_=C_2_)C_1_im]Cl		256^b^	180^c^	254^c^	270^c^	^481,485^
[C_4_C_1_pyrr][CF_3_SO_3_]		397^d^				^486^
[C_4_C_1_im][(CF_3_SO_2_)_2_N]			330^c^, 336^c^	419^c^, 423^c^	453^c^	^481,487,488^
[C_4_C_1_im]Br			215^c^, 224^c^	273^c^, 272^c^	300^c^	^481,487^
[C_4_C_1_im]Cl		262^b^	150^c^, 208^c^	264^c^, 257^c^	285^c^	^481,487^
[C_4_C_1_im][N(CN)_2_]			240^c^	300^c^		^487^
[C_4_C_1_im][PF_6_]			235^c^, 329^c^	373^c^, 421^c^	461^c^	^481,487^
[C_4_C_1_im][BF_4_]			285^c^, 290^c^	380^c^, 399^c^	440^c^	^481,487^
[C_4_C_1_im][CF_3_SO_3_]			340^c^, 354^c^	392^c^, 393^c^	426^c^	^481,487^
[C_2_C_1_im][C_1_CO_2_]			140^c^	214^f^, 221^c^	244^c^	^481,483^
[C_2_C_1_im]Br				301^f^		^483^
[C_2_C_1_im]Cl		256^a^		285^a^		^484,489^
[C_2_C_1_im][(C_1_O)_2_PO_2_]				286^c^		^490^
[C_2_C_1_im][CHO_2_]		212^b^				^485^
[C_2_C_1_im][C_1_SO_4_]				390^c^		^491^
[C_2_C_1_im][CF_3_SO_3_]				172^c^		^483^
[C_6_C_1_im][BF_4_]			262^c^	420^c^	465^c^	^481^
[(HO_3_S)^3^C_3_C_1_im][HSO_4_]			333^c^			^492^
[C_8_C_1_im]Cl			165^c^	276^c^	249^c^	^481^
[C_8_C_1_im][PF_6_]			334^c^	407^c^	443^c^	^481^
[C_8_C_1_im][BF_4_]			313^c^	397^c^	438^c^	^481^
[(OH)^2^C_2_N][C_1_CO_2_]				169^c^	285^c^	^493,494^
[(HO)^2^C_2_N][(HO)^1^C_2_CO_2_]				205^a^	270^a^, 398^c^	^494,495^
[(OH)^2^C_2_N][HSO_4_]			138^a^		157^a^	^496^
[((HO)^2^C_2_)_2_N][C_1_CO_2_]					423^c^	^494^
[C_4_im][HSO_4_]					365^c^	^497^
[C_4_C_1_N][HSO_4_]	286^c^					^498^
[Ch][C_1_CO_2_]				200^c^	222^c^	^499^
[Ch][Arg]				163^e^		^199^
[Ch]Cl				300^e^		^500^
[Ch][Gly]				150^e^		^199^
[Ch][Lys]				165^e^		^199^
[Ch][Try]				174^e^		^199^
[Ch][Ser]				182^e^		^199^
[C_2_C_2_N][HSO_4_]	302^c^		262^c^		301^c^	^501,498,502,501^
[C_2_N][HSO_4_]	296^c^		262^c^, 254^c^		292^c^	^501,498,502,501,503^
[(OH)^2^C_2_C_1_N][C_1_CO_2_]			61^f^	69^f^	88^f^	^504^
[Py][C_1_CO_2_]			51^c^	59^c^		^504^
[Py][NO_3_]			122^c^			^505^
[Py][H_2_PO_4_]			129^c^			^505^
[C_2_C_2_C_2_N][HSO_4_]		263^c^	270^c^	260	265^c^	^501,498,502,501^
[C_6_C_6_C_6_ C_14_P][C_1_CO_2_]		259^a^		276^e^		^506^
[C_6_C_6_C_6_C_14_P]Cl				320^g^, 341^f^, 355^c^, 365^a^		^507^

a All the listed measurements were performed under different flows of N_2_, with pans made of Al or Pt. In most cases, the detailed experimental procedures can be found in the corresponding references.

b Heating rate of 20 °C·min^–1^.

c *T* for 10% weight loss, heating rate of 10 °C·min^–1^.

d Heating rate of 10 °C·min^‑1^.

e *T* for 5% weight loss, heating rate of 20 °C·min^–1^.

f Heating rate of 5 °C·min^–1^.

g Heating rate of 2 °C·min^–1^.

h Heating rate of 1 °C·min^–1^.

Once a pretreatment stage has been performed, even after the stage of lignin precipitation by the addition of an antisolvent, a lignin fraction remains dissolved in the IL solution. Identifying and quantifying the lignin fragments dissolved in the IL solution is a challenging task. A common assumption is that the amount of lignin in solution would be the difference between the initial lignin content in the biomass and the sum of the lignin found in the pulp and the precipitated lignin. Regarding its structure, the lignin fraction that remains in the IL solution must have a sufficiently high solubility in the antisolvent (usually water). Small hydrophilic fragments are the most likely culprits.^174^

Changes occurring in the lignin structure during ionoSolv pretreatments with IL recycled over the course of various pretreatment cycles have been reported for different biomass types (*Miscanthus*, pine, CCA treated wood).^174,353,476^ In general, the effect of recycling the IL on lignin properties resembles that of a prolonged pretreatment. Lignin recondensation tends to increase as small lignin fragments that did not precipitate and remained in the solution after the first cycle have more opportunities to suffer condensation reactions in the subsequent runs.^353^ Also, the amount of β-O-4′ ether linkage and the amount of PCA units decrease with recycling, while and the amount of H-type subunits increase, consistent with the same PCA transformation detected for increased pretreatment severities. The presence of lignin that was not precipitated in the previous cycles has also led to peak lignin recovery yields after the second or third cycle of IL usage, on occasion exceeding significantly the level delignification achieved during the corresponding cycle and leading to lower saccharification results with similar levels of delignification.^174,476^ It is worth mentioning that the lignin recovered at this maximum has been proven to have higher *M*_n_, *M*_w_, and PDI than lignins recovered from both previous and subsequent cycles. This fraction also showed the highest chemical diversity. Usually, after reaching that maximum precipitate yield, the lignin yield recovered from the subsequent runs decreases slowly, but it still remains higher than that of the initial cycle. Furthermore, cumulative lignin yields after several cycles have been found to exceed the total amount of lignin extracted from the biomass in those cycles, which hints to the contribution of carbohydrate degradation products in the precipitate yields.^8^

Reducing the amount of water employed for the precipitation of lignin is of key importance, since the separation of the IL and water during the IL recycling stage is one of the main energetic and economic drawbacks for this type of process. On the other hand, reducing the amount of water during the precipitation stage could lead to a higher lignin concentration in the IL for the next cycle of pretreatment, which could increase lignin recondensation reactions and reactions with carbohydrate degradation products in the liquor, with negative implications for the quality of both the lignin and cellulose-rich streams. Abouelela *et al*. investigated the effect of reducing the amount water employed for the precipitation of lignin from 3 to 1 equiv in the reuse of the PIL [C_4_C_1_C_1_N]­[HSO_4_] over six consecutive pretreatment cycles using 20 wt% solid loading at 150 °C for 1 h. No significant changes were observed in the delignification ability in either case. Lignin precipitation with 1 water equiv resulted in lower lignin yields than using 3 equiv of water for the first and second cycles, with higher yields for the second cycle with both amounts of water. Also, the lignin recovered with 1 equiv of water has higher Mw and PDI. Lignin precipitation peaked in the third cycle for both water amounts, exceeding 100% when using 1 water equiv and reaching 84% with 3 water equiv, although the level of delignification of the pulps remained similar to previous cycles. Mw of the lignin recovered with 1 equiv in the third cycle showed a significant increase in *M*_w_ but decreased in the subsequent cycle. Lignin yields lowered in the next cycle to rise again for the last two cycles, and this variation was more pronounced for the samples recovered with only 1 equiv of water (Figure 31).^476^

**Figure 31:**
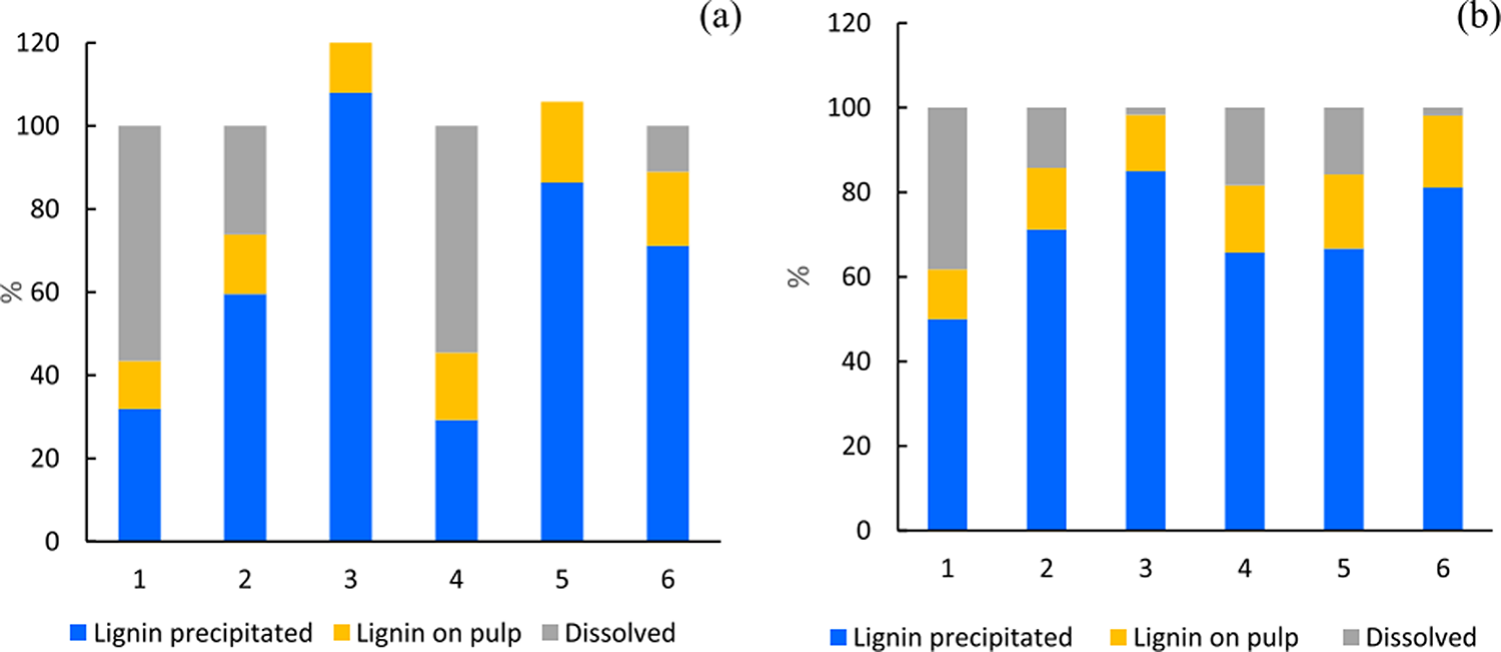
Lignin mass balances across six pretreatment cycles. (a) Using 1 water equiv as an antisolvent and (b) using 3 water equiv as an antisolvent. Adapted with permission from ref 476. Copyright 2021 American Chemical Society.

It should be noted that a small amount of the IL is being trapped or bound within the precipitated lignin, which leads to some degree of IL loss. Moreover, it has been reported that recycled PIL shows decreasing concentrations of [H^+^]. Losses of protons ranging from 11% for 10 wt% loading pretreatments to 26% for 50 wt% loading experiments have been reported for pretreatments of *Miscanthus* with [C_2_C_2_C_2_N]­[HSO_4_]_80%_, suggesting that the acidity of the recycled IL might need to be readjusted by the addition of small proportions of sulfuric acid.^182^ This, combined with the imbalanced sulfur:nitrogen ratio detected in the pulps, also indicates that the major source of PIL loss is neutralization of the acidic anion, and therefore the make-up stream is likely to be predominantly sulfuric acid.

##### 2.8.3.9 Delignification with Mixtures of IL and Other Solvents

Wu *et al.* reported an innovative approach for biomass fractionation by mixing an IL, [C_2_C_1_im]­[C_1_CO_2_], with an organic solvent, DMSO, with the goal of improving the economics of the process. They applied this system to the pretreatment of *Eucalyptus*, finding improvements in the digestibility of the recovered pulps. They reported an increase of 100% in specific surface area of the recovered pulps when mixtures of IL with DMSO with a ratio of 2:3 were used, compared to the pretreatment with the pure IL.^508^ Weerachanchai *et al.* studied the mixture of the ILs [C_2_C_1_im]­[C_1_CO_2_] and [C_4_C_1_im]­Cl, with dimethylacetamide (DMA) and ethanolamine in the pretreatments of corncob and rice straw. They found that mixtures of [C_2_C_1_im]­[C_1_CO_2_]:DMA with a ratio 60:40 resulted in higher delignification (98%) and sugar yields (95%) than the pretreatment with the pure IL. The lower viscosity of the resulting mixtures also offered advantages in facilitating the pulp washing procedures and allowing higher solids loadings.^509^

To overcome safety concerns related to the use of DMSO and DMA, Lynam *et al.* studied the addition of glycerol to different ILs, such as 1-ethyl-3-methyl­imidazolium formate ([C_2_C_1_im]​[CHO_2_]), [C_2_C_1_im]​[C_1_CO_2_], 1,3-dimethyl­imidazolium dimethyl­phosphate ([C_1_C_1_im]​[(C_1_O)_2_PO_2_]) and [C_2_C_1_im]​[(C_2_O)_2_PO_2_], in different proportions and used to pretreat different biomasses, including rice hulls, loblolly pine, and corn stover. They found that the addition of glycerol to [C_2_C_1_im]­[CHO_2_] gave the most relevant improvements in cellulose digestibility. They proposed that for this IL, the glycerol is more effective disrupting the anion–cation interactions, reducing the IL viscosity and enhancing the ions mobility, thus improving its accessibility to the biomass structure.^510^ According to their results, pretreatments of rice hull and corn stover with mixtures of [C_2_C_1_im]­[CHO_2_] containing 50 wt% and 75 wt% of glycerol gave the best results, improving glucose yields up to 40% relative to the pure IL, which they explained by the observed removal of lignin.^510,511^ For pine, they demonstrated that after a 3 h pretreatment at 140 °C, a mixture of 50 wt% of [C_2_C_1_im]­[CHO_2_] and 50 wt% of glycerol was effective removing lignin, recovering a pulp-like structure with only a 5% lignin content, where the individual fibres have been exposed, and greatly improving enzymatic hydrolysis yields of glucose and hemicellulosic sugars.^352^

Chen *et al.* extended this concept designing an hybrid ionoSolv–OrganoSolv fractionation by mixing PILs with varying amounts of different organic solvents (*i.e.*, ethanol, butanol, and acetone). Lignin fractions recovered after pretreatment of *Miscanthus* and pine using these mixtures were found to be less condensed than pure ionoSolv lignins. Intensity of the G_2cond_ signal relative to the G_2_ signal decreasing from 53% for the pure ionoSolv pretreatment to as low as only 16% for a mixture of IL with 80% butanol.^352^ S/G ratios also increased from 0.55 up to 0.72 and conversion of the PCA units into H units was prevented. *M*_w_ of the lignins recovered after pretreatment with the aid of ethanol or butanol also increased due to α-alkoxylation of the lignin fractions with the alcohol (this was not observed with acetone). The same has been observed for pretreatments of *Broussonetia papyrifera* using DESs ([Ch]­Cl/lactic acid) also assisted by the addition of ethanol. The presence of ethanol resulted in the incorporation of ethyl groups in the lignin structure, inhibiting the cleavage of β-O-4 bonds and reducing the condensation degree of the resulting lignin fractions.^512^

##### 2.8.3.10 Delignification of Contaminated Wood

Some PILs can extract contaminants from lignocellulose during the fractionation process.^38^ Purification of biomass containing heavy metals and polyaromatic compounds (PAHs) has been reported. In the case of extraction of heavy metal contamination from waste wood materials, [C_1_im]Cl showed the highest ability, followed by [C_1_im]­[HSO_4_], [C_2_C_1_im]­[C_1_CO_2_] and [(OH)^2^C_2_N]­[C_1_CO_2_].^353,462^ Once dissolved in the IL, the heavy metal contaminants can be recovered by electrochemical processes. Due to the ability of lignin to chelate with metals during the fractionation, the electrochemical stage should be implemented before the lignin precipitation stage to avoid the recovery of lignin contaminated with the extracted metals. It should be noted that complete de-metallization of all the recovered fractions is challenging and the overall processes could be considered as metal minimization and lignin valorization.^462^ PAHs extracted into the IL during the biomass fractionation have a high tendency to precipitate with lignin during the lignin precipitation stage due to their hydrophobicity. The partitioning of PAHs between IL liquor and lignin has been found to correlate with their degree of hydrophobicity and solubility in water.^466^ The hazardous nature of PAHs limits further valorization of the lignin fraction recovered from wood contaminated with them, which might be limited to energy generation in more costly WID compliant waste boilers.

##### 2.8.3.11 Process Intensification Effects

Since size reduction is an energy intensive process, the ability to pretreat particles of the largest possible size could help reduce energy demands of the whole process. A study conducted by Chambon *et al*. investigated this effect and showed that particle size also influences delignification. Their results showed that too large particles with lower surface area to volume ratios have a detrimental effect on delignification due to mass transfer issues. On the other hand, too small particles suffer from high lignin reprecipitation onto the pulp surface due to larger surface area, also impacting negatively on the delignification. The sweet spot where the two effects are balanced and maximum delignification is achieved was found to be intermediate sizes of ∼3 × 0.02 × 0.01 cm^3^ for *Miscanthus* pretreated with [C_2_C_2_C_2_N]­[HSO_4_] at 120 °C for 6 h and solids loading of 20 wt%.^182^ The properties of the recovered lignin are also affected as a result of the particle size. The largest particles produce less condensed and less polymerized lignins, due to the slower diffusion of the IL into the biomass and of the lignin out of it. As a result, increased β-O-4′ linkage concentrations and S/G ratios and higher *M*_w_ and PDIs were observed with increased particle size. Since comminution is highly energy-intensive and costly, pretreating larger particles would reduce energy costs during biomass processing.

Process intensification studies have reported that increasing pretreatment scale by 100-fold, from 10 mL to 1 L, resulted in higher lignin recoveries and slightly improved delignification. These were attributed to the employment of stirring at the higher scale, which facilitated heat and mass transfer processes. It should be noted that solids loadings above 20 wt% impeded the mechanical stirring, marking the solids loading upper limit. At that concentration, a decrease in delignification and lignin recovery with respect to the experiments at 10 wt% solids loadings were observed, due to the increased surface area for lignin reprecipitation. Increasing the stirring speed also improved lignin extraction, resulting in higher lignin yields, an effect more acute with bigger particles. Again, this hints to mass and heat transfer being key. Also, lignins recovered at higher mixing speeds showed slight decrease in the abundance of β-O-4′ linkages and a slight increase in condensation.^182^

#### 2.8.4 Influence of Hydrogen Bonding on Delignification

In systems with more than 1 solvent, the value of total hydrogen bond of IL-lignin (*H*_T_) would be equal to the contribution of solvent–lignin hydrogen bonds by both solvents (*H*_T_ = *H*_solvent1–lingin_ + *H*_solvent–2‑lignin_). Increasing the contribution by any of the solvents would improve the solubility of lignin in the system. Therefore, the use of co-solvents with high H bond capability with lignin can increase lignin solubility significantly. Alcohols with high boiling points (HBS) such as ethylene glycol (EG), 2,3-propanediol (PG), glycerin (Gly) and 2,3-butanediol (But) have been proposed and both computational and experimental results have shown the efficiency of this approach.^458^ It has been suggested that not only the HBS have strong H bonding interactions with lignin on their own, but their presence also decreases the interaction between the cation and the anion of the IL, leading to an increase in the interaction between lignin and the IL ions. Similarly, a hybrid pretreatment using the IL [C_4_C_1_im]­[HSO_4_] together with ethanol or butanol allowed for increased delignification with increases up to 11% and lignin removals up to 89%. Also, an increase in lignin yields of up to 20% higher than without cosolvents was observed, with lignin yields exceeding delignification rates. This has been ascribed to the increase in *M*_w_ of the lignin fractions due to alkoxylation reactions with the alcohols, and to the presence of hemicellulose moieties, as LCC are better preserved when ethanol and butanol are added to the system.^352^ On the other hand, the use of acetone instead of the alcohols did not lead to significant improvements in delignification, due to the poor lignin solubility in acetone. Increased lignin yields than delignification was also observed. In this case, the explanation remains unclear, but the formation of pseudo-lignin and reactivity with sugar degradation products has been suggested.^352^

Furthermore, the addition of co-solvents leads to reductions in pH and viscosity of the systems, which can help mitigate the formation of pseudo-lignin. Also, the use of co-solvents could be beneficial for the economics of processes where the IL is more expensive than the cosolvent. It should be noted that ethanol and butanol can be produced in situ in biorefineries, which can facilitate the implementation of this approach, reducing capital costs. Furthermore, the removal of solvents with lower boiling points can reduce the energy input requirements for the recycling on the ILs.^352^

It was found that, in protic ILs, H bonding has a big effect on anion–cation interactions, altering the solvation of the protonated starting material and therefore the overall rate of reaction. Comparison of reaction rates in these ionic liquids with results within aqueous or aqueous/organic media indicate that the ionic liquids facilitate more rapid cleavage of the β-O-4 ether linkage even under less acidic conditions. All the reported results give a complete overview of both the mechanistic and solvation effects of acidic ILs on lignin model compounds and provide scope for the appropriate selection and design of ILs for lignin processing.^39^

For DESs the presence of extensive H-bonding networks can increase solvent viscosity, causing low rates of heat and mass transfer during pretreatment, hence hindering delignification capability.^297^ DESs have strong H-bonding networks, which contribute to high interactions with lignins. DESs can be very efficient for biomass delignification thanks to synergistic effects of HBDs and HBAs. HBAs usually consist of ammonium, phosphonium, or sulfonium salts. It has been found that the coordinating capability of the cations in the HBA is related to the delignification capability of the corresponding DESs. For example, DESs with C=C groups in the HBA dissolved more lignin than HBAs with −OH, with HBAs with benzene groups giving the lower delignification. Furthermore, HBAs with similar structure and shorter alkyl lengths also led to increased lignin removal, likely due to having less steric hindrance.

The type and number of functional groups present in the IL or DES has a big influence in their capability to solubilize lignin. For example, the presence of carboxyl groups (*e.g.*, formic acid, oxalic acid, and lactic acid) in the HBD of a DES enhances its delignification performance more than other functional groups like alcohol, amine or amide groups, with values of >90% for most types of lignocellulosic biomass. This is ascribed to the presence of acidic protons that can catalyze the cleavage of ether linkages of lignin and ester linkages in LCCs. Also, it has been proposed that C–H···π and O···H bonding interactions between the acid and lignin enhances its dissolution. Interestingly, increasing the number of carboxylic groups in the HBD reduced the delignification ability, in the order [Ch]­Cl:LA > [Ch]­Cl:malic acid (MA) > [Ch]­Cl:citric acid. It has been proposed that the extra carboxyl groups in HBDs can form H-bond networks between HBAs and HBDs, weakening the ability of the HBA to compete with intramolecular bonding in the biomass components, reducing the mass transfer rates in the system. The same effect has been found for polyalcohol HBDs (*e.g.*, the delignification ability of polyalcohol based DESs with lactic acid as HBA decreased in the order of ethylene glycol > glycerol > xylitol). Nevertheless, the presence of hydroxyl groups in HBDs that also contain carboxylic groups improves their delignification capability. For example, [Ch]­Cl:LA achieved higher lignin yield (33.5%) than [Ch]­Cl:propionic acid (20.4%), and [Ch]­Cl:MA achieved higher lignin yield (22%) than [Ch]­Cl:succinic acid (6%). HBDs with amine/amide groups, such as urea, imidazole, and ethanolamine promote the deprotonation of the phenolic hydroxyl groups of lignin, resulting in good lignin extraction ability. Furthermore, increasing basicity of the HBD increases lignin extraction yields (*e.g.*, lignin removal yield of [Ch]­Cl:monoethanolamine 81.0%, [Ch]­Cl:diethanolamine 73.5%, and [Ch]­Cl:*N*-methyldiethanolamine 44.6%, to [Ch]­Cl:urea 27.7%). As for HBDs with hydroxyl groups, the presence of multiple amine or amide groups in a HBD has a detrimental effect on the delignification capability due to the formation of more intermolecular H-bonds with HBAs (*e.g.*, LA/formamide achieved a higher lignin removal rate from rice straw than LA/urea). Length of the alkyl chain of the HBD also affects the delignification ability, mainly for a steric hindrance effect. However, electron-donating effects and interference in hydrogen bonding have been suggested.^297^

Xia *et al.* showed that the DES [Ch]­Cl:gly (1:2) had weak competing interactions towards the linkages in the LCC network because of its intramolecular H-bonds. Furthermore, due to the absence of active protons and acidic sites, the DES was unable to cleave ether bond linkages in the LCCs. Adding AlCl_3_·6H_2_O to the DES allowed to cleave both the intramolecular H-bonds of the DES and ether bonds in LCCs improving the lignin fractionation from 3.61% to 95.46%.^403^

#### 2.8.5 Lignin Solubilization: Chemical Mechanisms

##### 2.8.5.1 Depolymerization Reactions

During pretreatment, lignin can experience depolymerization and recondensation reactions and the balance between both together with the particular chemical pathways are strongly influenced by the pretreatment severity.^297^ The analysis of the lignin fractions recovered after pretreatment offers key information to elucidate its reactivity and can help to tune pretreatment conditions to yield lignin fractions with optimal properties for further valorization.

In typical delignification processes, including those with ILs and DESs, the main driving force for lignin solubilization is the cleavage of β-O-4′ ether bonds and 4-*O*-methylglucuronic acid ester bonds between lignin and hemicellulose.^352^ Degradation of β–β′ linkages and reduction in the methoxy content have been also observed in some cases, and it has been proposed that some ILs attack the oxygen-containing groups and the aryl ether bonds in the lignin side chain.^513^ These reactions lead to a reduction of the molecular weight of the resulting lignins by the depolymerization of the lignin chains. The nature of the IL or DES plays a key role in determining the chemical pathways followed by lignin molecules during pretreatment.

##### 2.8.5.2 Mechanistic Comparisons: Acidic vs Basic Pretreatments

Dutta *et al*. compared the solubility of Kraft lignin in three different types of ILs: the AIL [C_2_C_1_im]­[C_1_CO_2_], the PIL [C_2_C_2_C_2_N]­[HSO_4_] and the BIL [Ch]­[Lys]. Treatment with the PIL [C_2_C_2_C_2_N]­[HSO_4_] showed the highest level or breakdown of β-O-4′ linkages, as well as the highest amount of dehydration and recondensation reactions and the lowest decrease in molecular weight, producing guaiacyl acetone as the main product. Treatment with the AIL [C_2_C_1_im]­[C_1_CO_2_] showed the lowest degree of breakdown of β-O-4′ linkages, the highest decrease in molecular weight and the highest amount of monomeric depolymerization products with high product diversity. Finally, the BIL showed an intermediate behavior, with moderate rates of β-O-4′ linkages breakage, dehydration, and recondensation, producing similar proportions of guaiacylacetone and guaiacol (Figure 32).^513^

**Figure 32:**
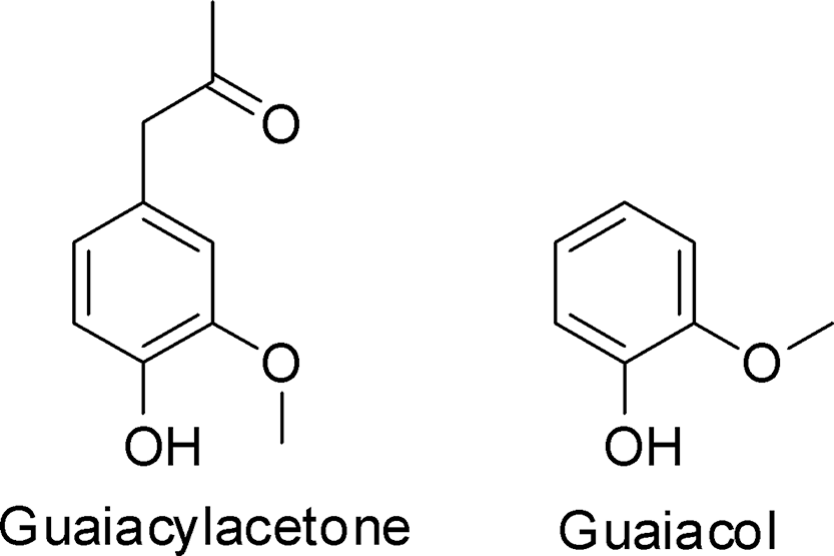
Structures of guaiacylacetone and guaiacol, products from the breakage of lignin β-O-4′ linkages.

It has been reported that the α,β-dehydration reaction of a lignin dimeric model compound with a β-O-4 aryl ether linkage (guaiacylglycerol-β-guaiacyl ether) is faster in the alkaline IL [C_2_C_1_im]­[C_1_CO_2_] than in the acidic [C_2_C_1_im]Cl due to a higher alkalinity and affinity to water of the acetate anion (Scheme 1).^39,514,513^

**Scheme 1:**
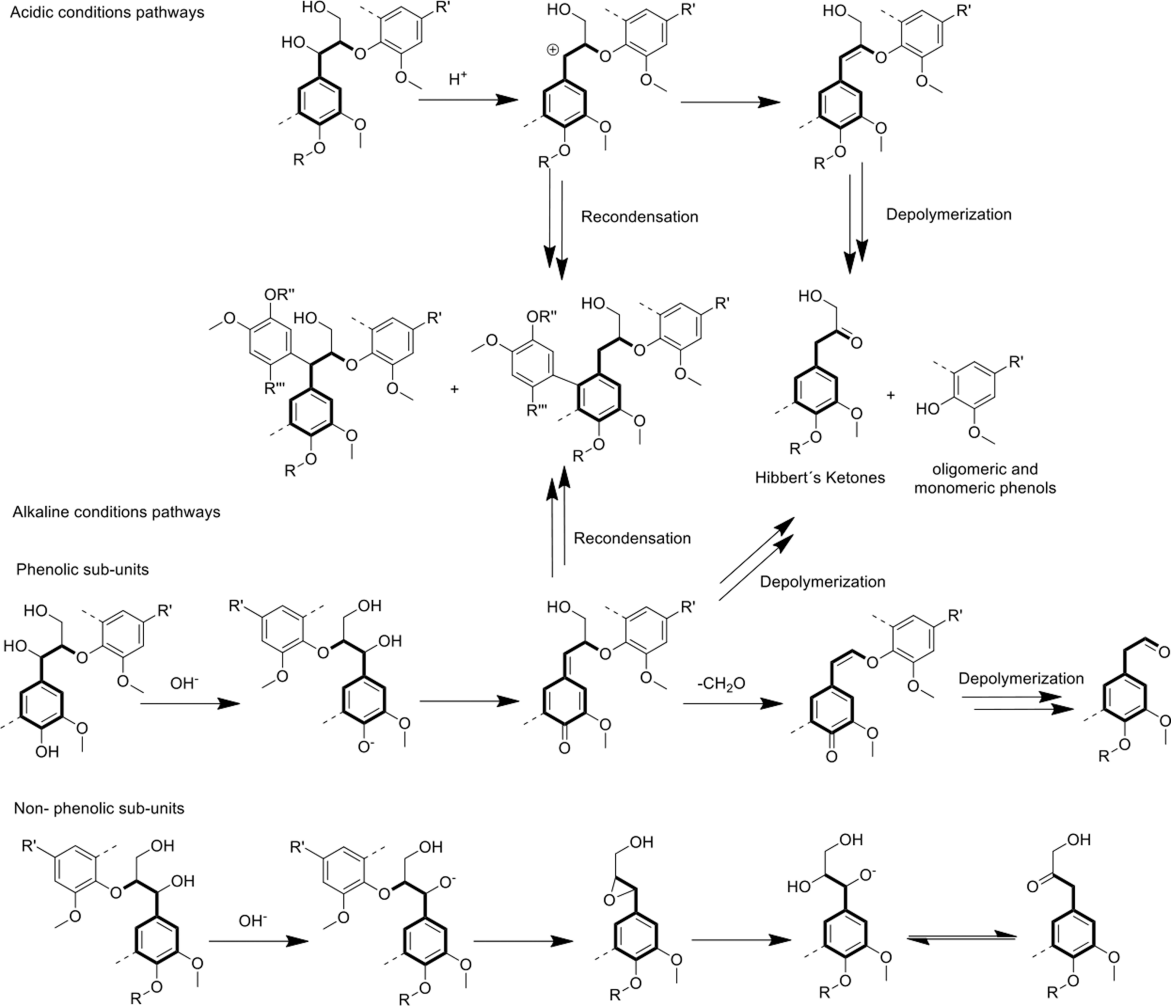
Proposed Lignin Reactivity Pathways under Acidic and Alkaline Conditions^457^

2.8.5.2.1. *Acidic Pretreatment*. The main depolymerization pathway during pretreatments with acidic ILs and DESs is an acid catalyzed hydrolysis of the β-O-4′ linkages by an E1 dehydration mechanism, which is the rate-controlling step for the reaction. Investigation of reaction kinetics using lignin models with systems based on a protic IL ([C_4_C_1_im]­[HSO_4_]) with different acid and water content showed that the rate of ether cleavage increases with the acidity of the system, but the presence of excess water can slow the dehydration step, as detailed in the mechanism shown in Scheme 2.^344^

**Scheme 2:**
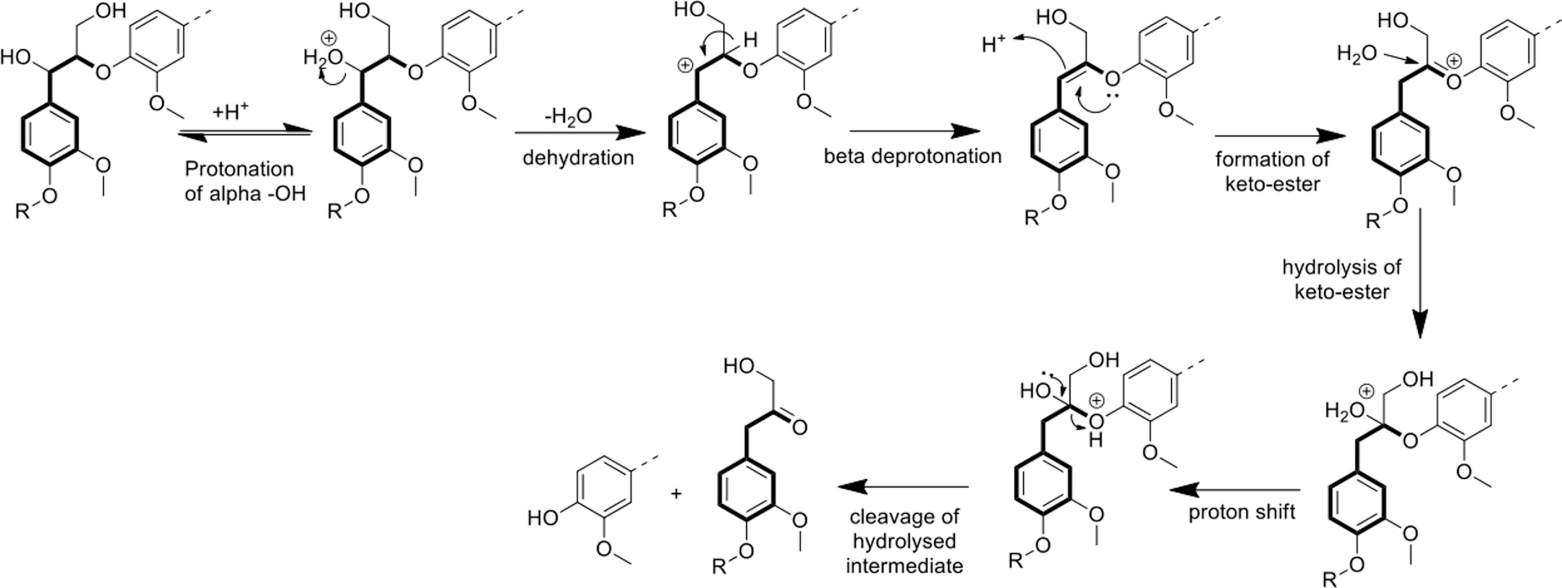
Acid Catalyzed Mechanism for Hydrolysis of Lignin Leading to the Formation of the Hibbert Ketone^344^

Furthermore, it has been suggested that the association between the ions of the IL has a strong influence on the lignin solvation and the reaction rates and that ILs with strong cation–anion association (strong hydrogen bonding between anion and cation) could favor lignin depolymerization at milder conditions. It has been reported that for a series of ILs based on the anion [HSO_4_]^−^ and different cations, both protic and aprotic, stronger interactions of the anion with the cation weaken the interaction of the anion with the oxonium intermediates, decreasing its activation enthalpy (Figure 33). Also, these results and the values of enthalpy and entropy obtained with all the ILs are consistent with the proposed E1 mechanism.^344^

**Figure 33:**
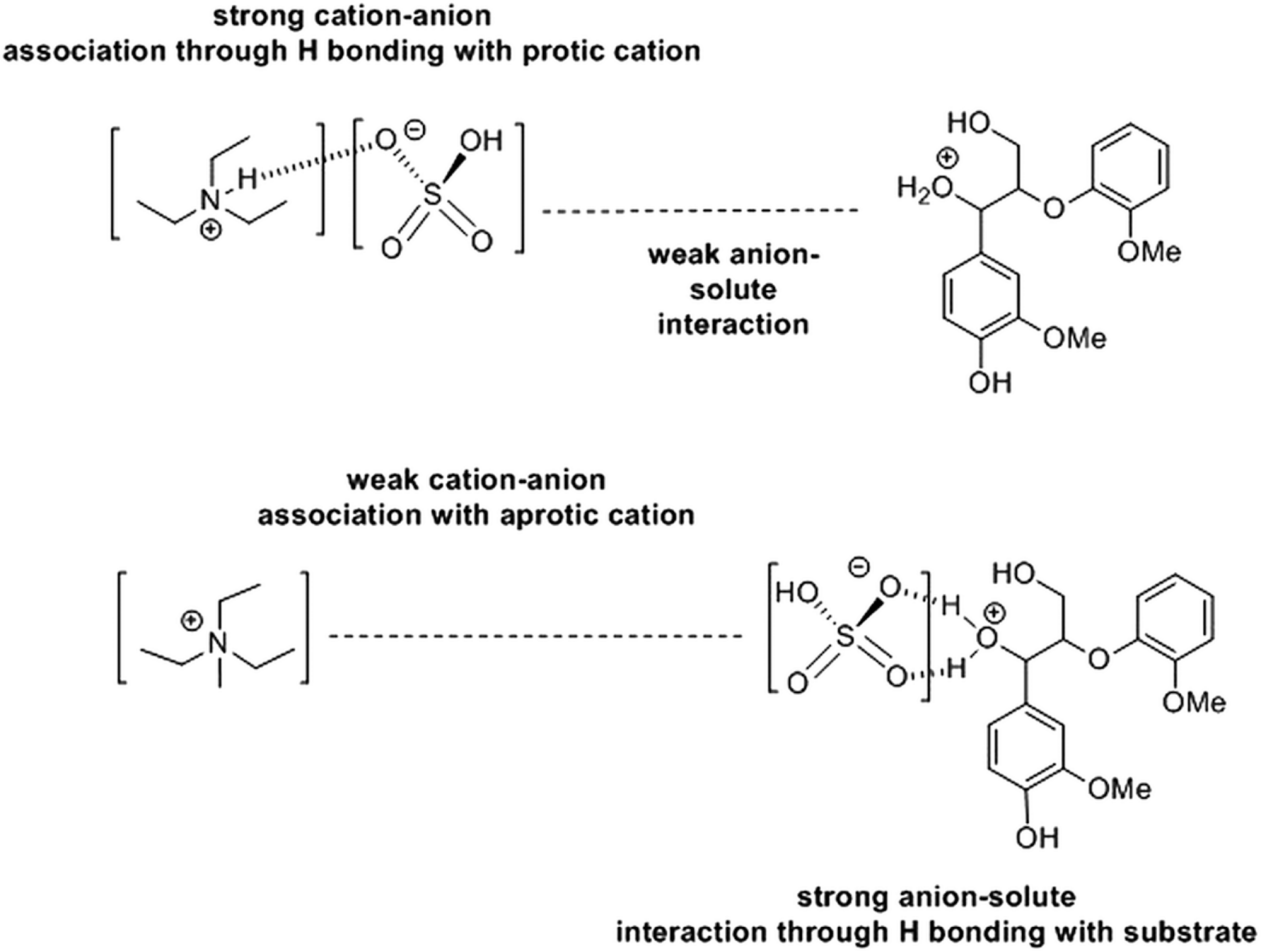
Effect of cation–anion interactions within [C_2_C_2_C_2_N]­[HSO_4_] ILs on the solvation of the oxonium intermediate, before the water dissociation to form the activated complex. Adapted with permission from ref 344. Copyright 2016 Royal Society of Chemistry.

2.8.5.2.2. *Influence of the Anion*. It has been suggested that the anions can act as nucleophiles during lignin depolymerization and have a deterministic influence in determining the chemical pathways during lignin depolymerization. It has also been suggested that in DESs with halogen anions, the formation of lignin–halogenide intermediates increases the rate of cleavage reactions.^25^ For pretreatments with ILs with coordinating anions (stronger H bond basicity, *e.g.*, Cl^−^, Br^−^, and [HSO_4_]^−^) and DES with anions capable of interacting with the γ-OH group (*e.g.*, a halide anion) lignin depolymerization proceeds via an elimination reaction between the α-carbocation and β-H, yielding an enol ether intermediate. Direct acid hydrolysis of the acid-labile enol ethers similar to that of ILs with coordinating anions yields guaiacol and Hibbert′s ketone derivatives (Scheme 3, Route I).^297,344,352,39^ It has been shown that the ability of the anion to interact with the γ-OH group helps prevent the loss of the γ-hydroxymethyl group as formaldehyde. However, certain degree of loss of the γ-methylene group has been reported, suggesting that deformylation might still occur.^344^

**Scheme 3:**
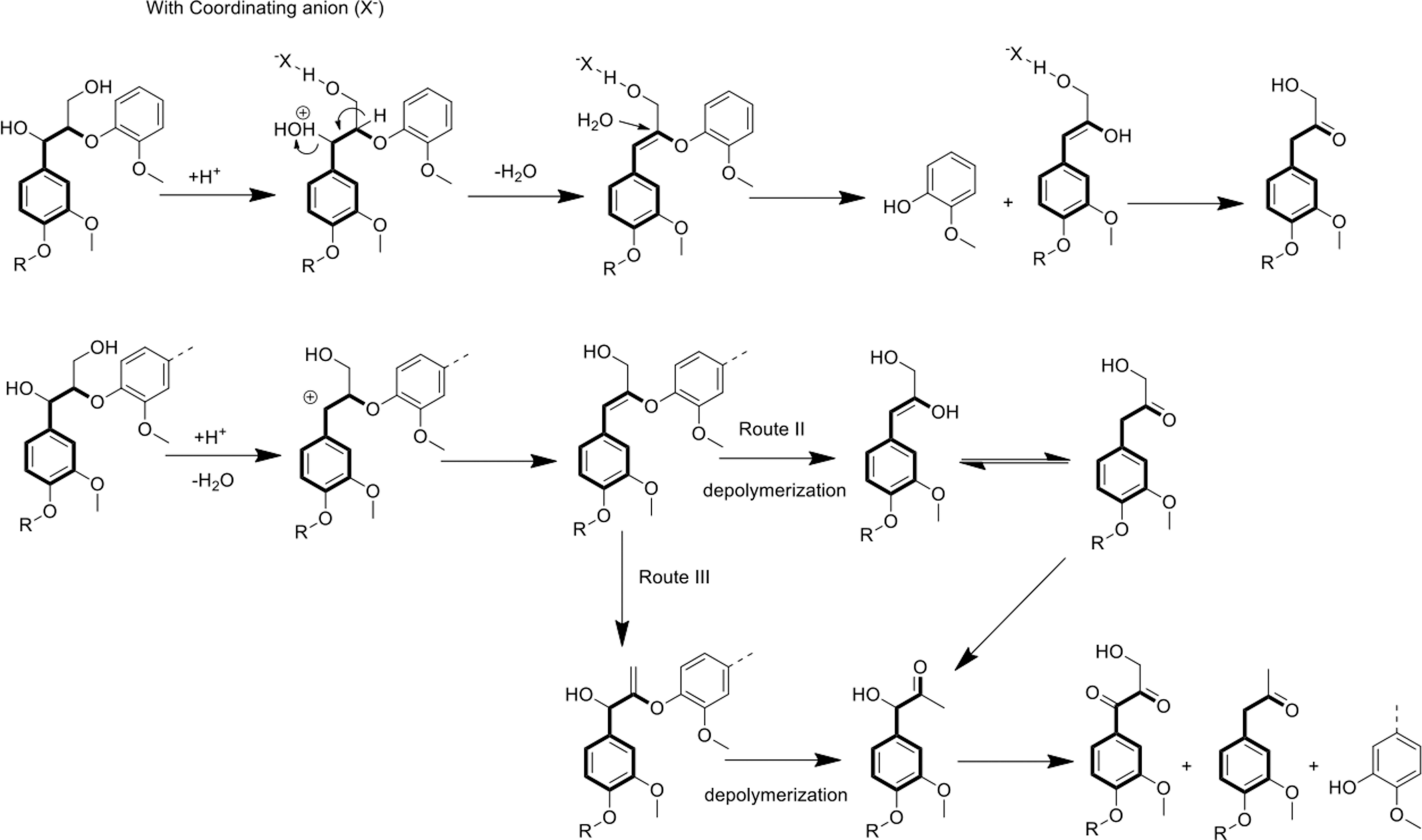
Mechanism of β-O-4′ Aryl Ether Bond Cleavage in ILs and DESs with Coordinating Anions^297^

For DES pretreatment another pathway has been also reported, via an allylic rearrangement of an enol ether intermediate followed by hydrolysis (Scheme 3, route II).^297^ In both cases, β-O-4′ bonds are cleaved and modifications of the Hibbert’s ketone lead to depolymerized compounds, including mono- and diketones, the latter having been only found in lignin recovered from DES pretreatments (Scheme 4). Alvarez-Vasco *et al*. reported that when the same compound was treated with the acidic DES [Ch]­Cl/lactic acid, it got converted into guaiacol and Hibbert’s ketone following the same reaction mechanism.^411^ It is also worth noting that lignin recovered from DES pretreatments show very little by-products or phenolic recondensation products, which could be due to the employment of milder pretreatment conditions, showing an advantage for the potential development of high value applications.

**Scheme 4:**
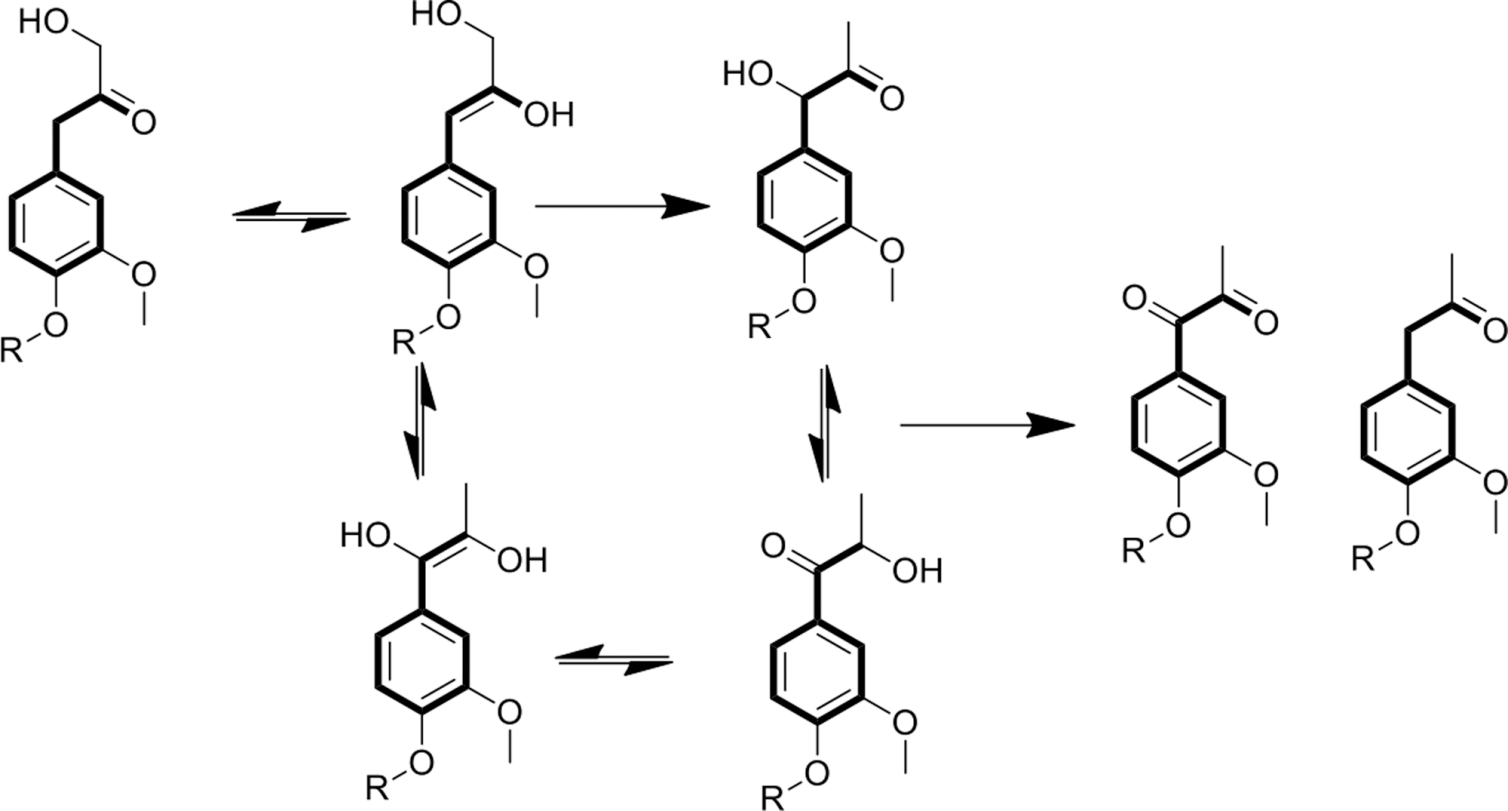
Mono- and Diketones Observed from Lignins Cleaved with Acidic DESs^297^

For ILs and DESs with less coordinating anions (*e.g.*, [BF_4_]^−^), the breakage proceeds by the formation of a stable benzylic carbocation by the protonation of the α-OH group followed by the elimination of the terminal γ-CH_2_OH group to generate α–β unsaturated compounds and formaldehyde, which further promotes the cleavage of ether bonds similar to that of ILs without coordinating anions (Scheme 5).^297,515,516^ Another route proposed for the cleavage of ether bonds with DESs containing lactic acid as HBD involves the oxidation of the Cα position and the acylation of the Cγ position (Scheme 6). The Cα ketone is key to promote the cleavage of β-O-4′ linkages and the mechanism for C–O cleavage involved in formylation, elimination and hydrolysis.^297^

**Scheme 5:**
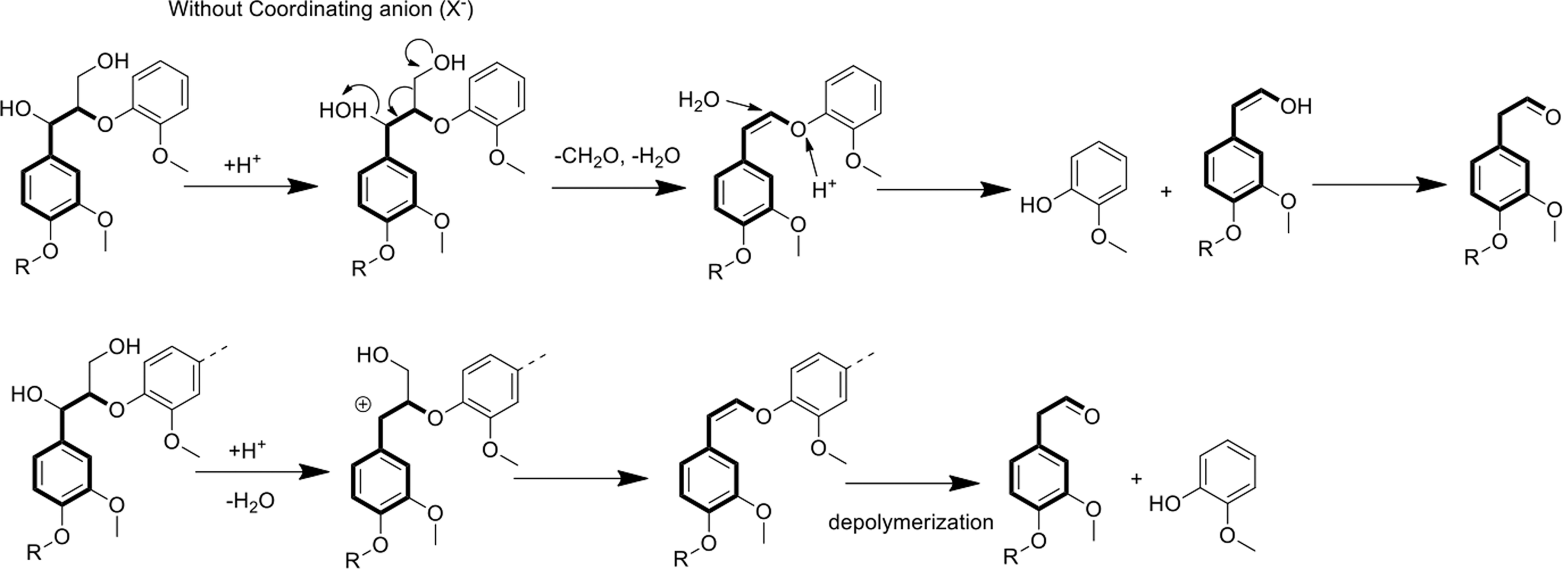
Reaction Pathway Proposed for Acidic Pretreatment in ILs and DESs without Coordinating Anions

**Scheme 6:**
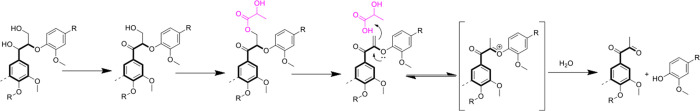
Possible Pathway for the Cleavage of β-O-4′ Linkages during Pretreatment with Lactic Acid as Proposed by Hong *et al*.^297^

Acidic DESs can also degrade a small portion of C–C bonds as β-5′ and β–β′, facilitating lignin separation from lignocellulose (Figure 34).^297^

**Figure 34:**
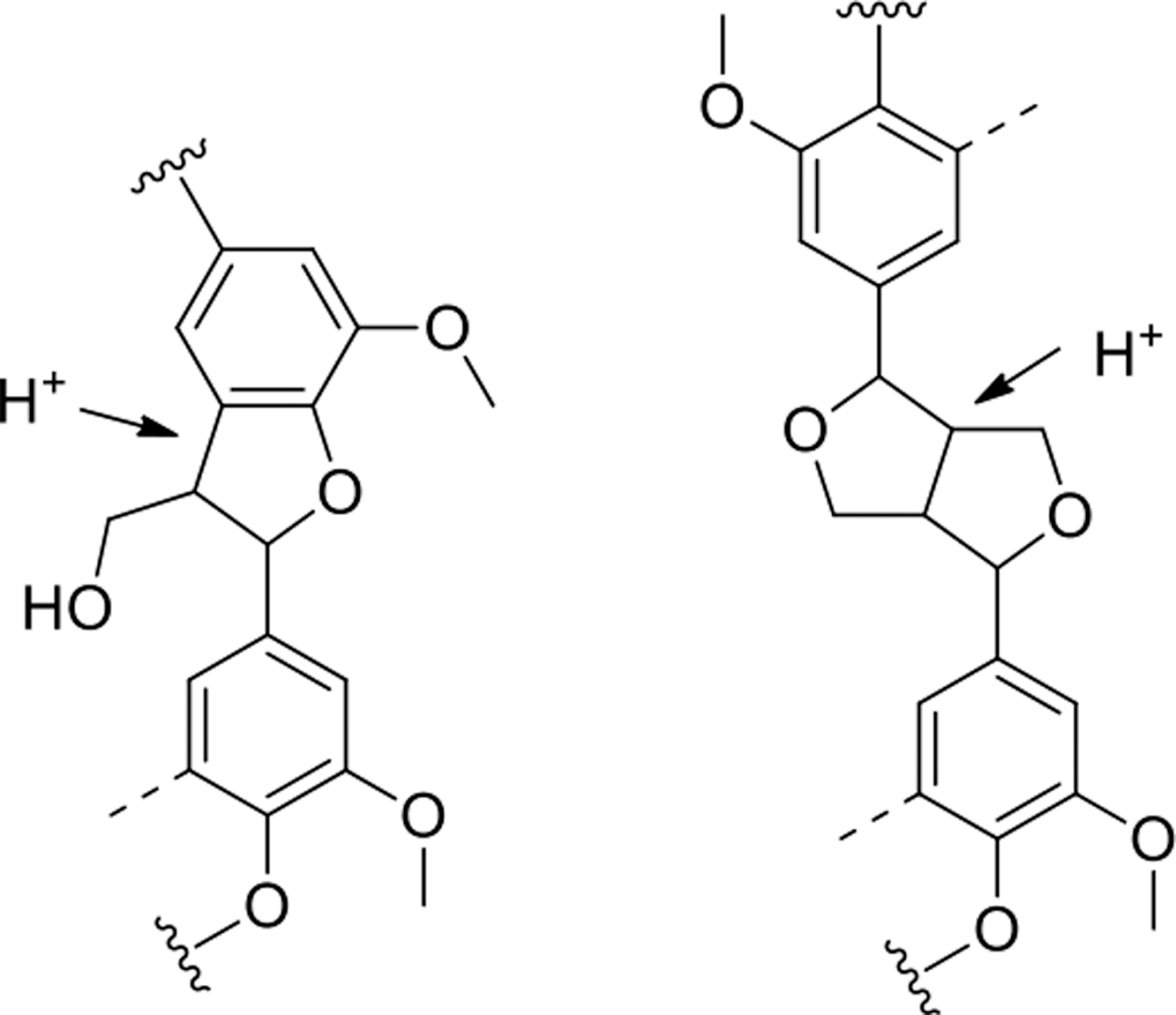
Acid attack on β-5′ and β–β′ bonds.

2.8.5.2.3. *Alkaline Pretreatments*. Lignin is alkaline-soluble, and pretreatment with basic ILs and DESs can also lead to the cleavage of its ether bonds as well as to demethoxylation and dehydration reactions. Repolymerization can also happen, mainly through the formation of C–C β–β′ and β-5′ linkages.^516^

Varanasi *et al.* investigated the ability of the IL 1-ethyl-3-methylimidazolium acetate ([C_2_C_1_im]­[C_1_CO_2_]) to depolymerize lignin.^471^ They compared technical lignins (kraft and alkali lignin) with switchgrass, pine and *Eucalyptus* during pretreatment. Lignin samples recovered after 6 h at 160 °C were recovered and analyzed. Kraft lignin produced guaiacol and vanillin. switchgrass produced syringol, guaiacol, allyl guaiacol and a variety of minor products (methyl guaiacol, ethyl guaiacol, vinyl guaiacol, guaiacyl acetone and acetosyringone), but no vanillin. *Eucalyptus*, with lignin than contains higher amount of S than G units, produced syringol, allyl syringol, guaiacol, allyl guaiacol, vanillin, with ethyl guaiacol, vanillin, guaiacyl acetone and acetosyringone produced as minor components. Since pine lignin is mainly formed by G units, it produced guaiacol and allyl guaiacol as main products and minor amounts of methyl guaiacol, ethyl guaiacol, vinyl guaiacol and guaiacyl acetone were detected. For both technical lignins and lignocellulosic biomass samples, it was found that decreasing the pretreatment temperature and/or time resulted in a higher quantity of unsaturated guaiacols and aldehydes.

Lignins obtained with basic DESs have lower purity and are more recondensed and have smaller particle size than those recovered with acidic DESs. Plus, DESs with acidic HBDs show better lignin removal performance.^25^ The reactivity of the β-O-4′ bonds within the lignin substructure highly depends on the type of the lignin subunits. For pretreatments with alkaline DESs, different mechanisms have been proposed for subunits with free phenolic OH groups and subunits with etherified phenolic OH groups (Scheme 7).^297^ For non-phenolic subunits the transformation occurs via the formation of an epoxide intermediate. On the other hand, lignin with subunits with free phenolic OH groups under pretreatment conditions with alkaline ILs (*e.g.*, choline lysinate, [Ch]­[Lys]) and DESs resulted in the deprotonation of phenolic OH groups, leading to the formation of a quinone methide-type intermediates that then underwent depolymerization and/or repolymerization reactions (Scheme 7).^297,513^

**Scheme 7:**
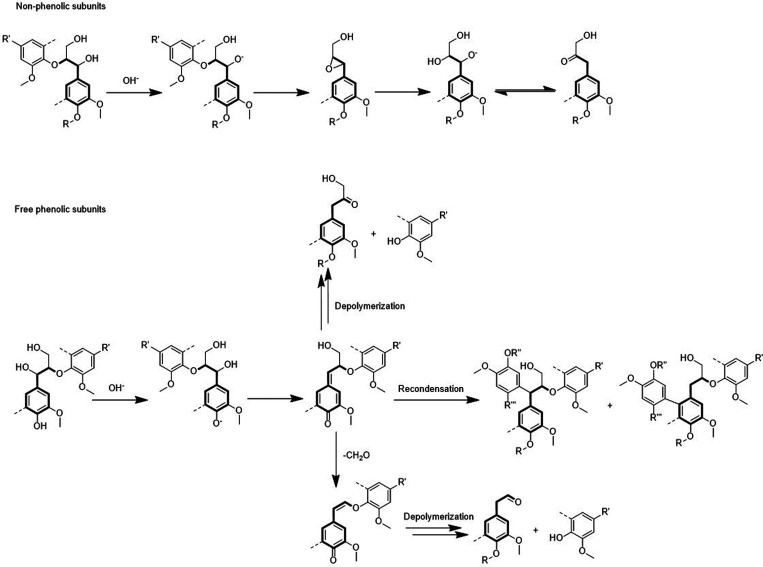
Proposed Reaction Pathways for Lignin during Alkaline Pretreatment for Nonphenolic Subunits (above) and for Free Phenolic Subunits (below)^297^

2.8.5.2.4. *Neutral DES Pretreatment*. Pretreatment with neutral DES has less impact on lignin extraction and structural transformations. Several studies have found that lignin recovered after pretreatments with neutral DESs, the β-O-4′ linkages are mostly preserved, with higher β-O-4′ content and lower degree of recondensation that lignins recovered after pretreatments with either acidic or alkaline conditions.^513,517,392,518^ According to Yu *et al*., this can be due to the strong competition with the intramolecular H-bonds between the components of the DES that could weaken its interactions with the linkages of the lignin–carbohydrate complex.^403^

##### 2.8.5.3 Lignin Degradation Pathways

Many studies have highlighted that two main pathways can be described for lignin degradation during pretreatment with ILs, the first being the preferential degradation of G units via demethoxylation and dehydration reactions. This pathway is typical of alkaline pretreatments and has been observed with basic AILs and with PILs under low severity conditions (typically with temperatures up to 120 °C).^360^

It has been reported that during the dissolution of lignin in alkaline ILs, it suffers the removal of oxygen-containing groups, such as the aryl methoxy group, demethylation reactions and the formation of unsaturated bonds. The positive charge of the IL cation can put a strong force, similar to hydrogen bonding, on the methoxy group of the side chain of lignin, weakening its binding force with the main unit of lignin structure, weakening the interaction between oxygen and carbon atoms in the methoxy group and resulting in the removal of the methyl group and the formation of a new phenolic hydroxyl group and leading to an increase in the G/S ratio. ILs with shorter alkyl chains in the cation have stronger alkalinity, leading to more degradation of the oxygen-containing groups in the lignin side chain. Alkaline treatment of wood preferentially breaks the G-lignin aryl ether bonds due to the presence of increased phenolic moieties when compared to ethylated moieties.

The second pathway consists of the selective degradation of S-units. This is typical of acid pretreatments and has been observed for IL pretreatments under higher severity conditions (temperatures above 120 °C). This leads to the acidic cleavage of ether linkages, mainly β-O-4′, with predominant removal of S units.

Examples of dual pathway behaviour depending on the temperature chosen for the pretreatment have been reported for the pretreatment of different biomass types including *Eucalyptus*, oak, poplar and switchgrass (*Panicum virgatum*) with different types of ILs and DESs, including the AIL 1-ethyl-3-methylimidazolium acetate ([C_2_C_1_im]­[C_1_CO_2_]), the PIL [C_1_im]­Cl, and the DES [Ch]­Cl:LA (1:10).^360,295,519^

It should be noted that discrepancies regarding the trends in the physicochemical properties of the resulting lignins are found in the literature. Some authors claim that the S-degradation pathway resulted in more heterogeneous lignins with higher molecular weights than the G-degradation pathway. In this case, the acidic conditions achieved with PIL [C_1_im]Cl and the high temperatures, allowed for C–C repolymerization reactions in the reaction media to overcome the depolymerization via cleavage of β-O-4′ linkages, resulting in more heterogeneous lignins with higher *M*_w_, lower *M*_n_, and much higher PDI.^360^ On the other hand, other authors reported that lignin recovered from the pretreatment of *Eucalyptus* with [Ch]­Cl:LA (1:10) suffered the cleavage of aryl–ether and C–C bonds and demethoxylation reactions at higher temperatures, leading to a decrease in the molecular weights. The proposed chemical transformations are shown in Scheme 8.^519^

**Scheme 8:**
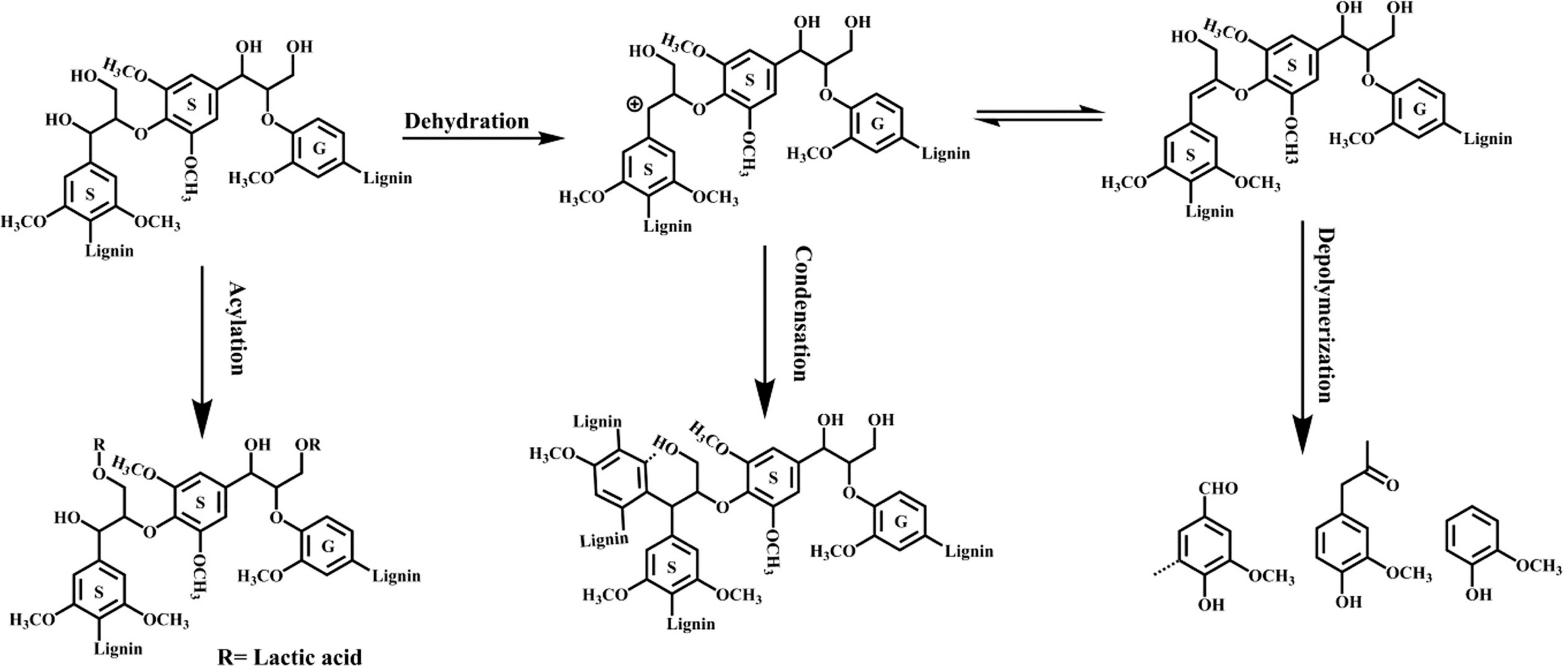
Proposed DES with [Ch]­Cl:LA (1:10), Pretreatment Mechanism of Lignin^519^

2.8.5.3.1. *Recondensation Reactions and Pseudolignin Formation*. As discussed earlier, lignin repolymerization often occurs during pretreatment of biomass. The most common recondensation reactions are the formation of covalent bonds between the α-C of the benzylic carbocation and electron-rich positions on aromatic rings, yielding diphenylmethane compounds, and the coupling between electrophilic positions on the aromatic rings of benzylic carbocations and electron-rich positions of aromatic rings to form biphenyl compounds (Schemes 9 and 10, route 1). Other reactions can also happen at the carbon α of lignin structures. For example, guaiacyl groups can form new C–C bonds with other units at the C-5 position (Scheme 10, route 2). For syringyl units, where the C-5 is protected by the presence of a methoxy group, this recondensation is not possible.

**Scheme 9:**
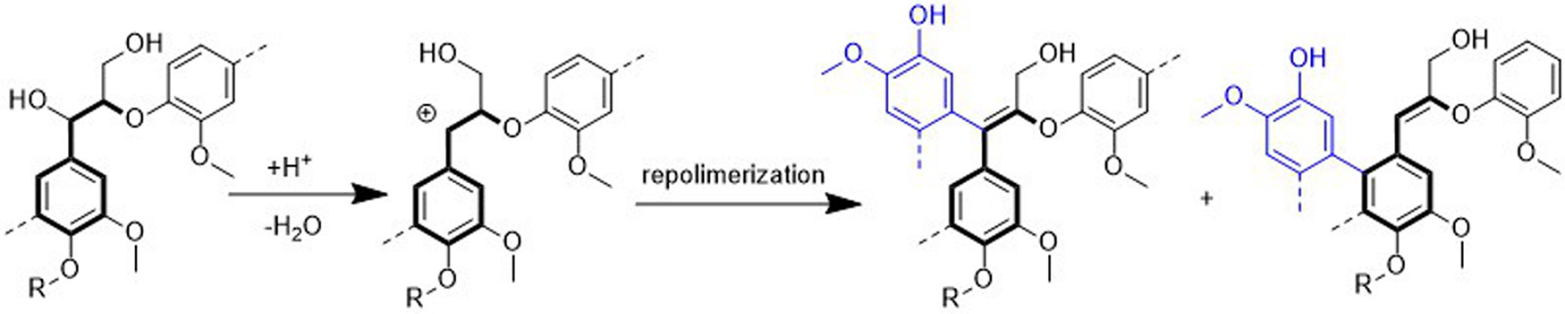
Proposed Reaction Pathway for Repolymerization of Lignin to Form Diphenylmethane and Biphenyl Compounds^297^

**Scheme 10:**
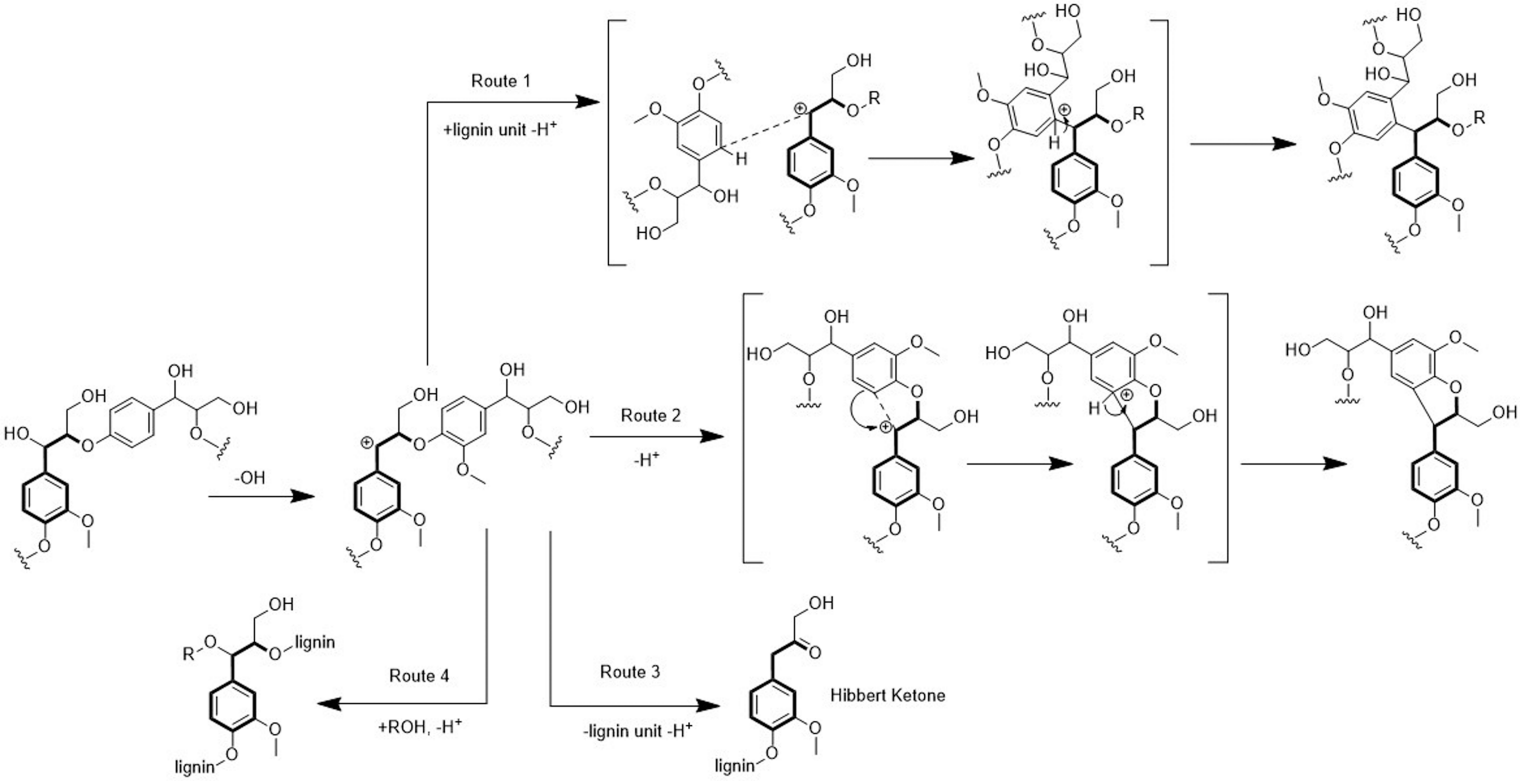
Possible Routes for Modifications at Side Chain Α Carbon of Monolignols during OrganoSolv–IonoSolv Pretreatment^a^ ^352^

a Route 1: interchain condensation with carbon 5 of adjacent G subunit forming a phenylcoumaran-like degraded structure; route 2: interchain condensation with electron-rich carbon of an unattached monolignol forming a new C–C bond; route 3: hydrolysis to Hibbert ketone; route 4: highly reactive benzylic cations are trapped by the alcohol solvent molecules forming ethers that suppress the lignin degradation at the α-positions.

2.8.5.3.2. *Humins Formation*. It has been hypothesized that hemicellulose sugars may be incorporated into the precipitate fractions. This is supported by the hemicellulose mass balances that showed significant proportions of hemicellulose carbohydrates could not being traced as sugars or furfural, in particular at higher severity conditions.^174^ The C5 and C6 sugars undergo dehydration into furfural and 5-HMF. Then, furfural and 5-HMF and their dehydration products (*e.g.*, formic and levulinic acids) can participate in polymerization reactions with other aromatic compounds including lignin fragments (Scheme 11).^468^

**Scheme 11:**
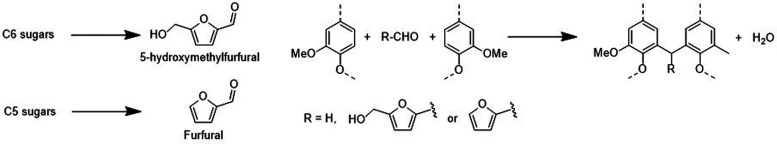
Condensation of Aldehydes with Lignin Free-Phenolic Units^468^

##### 2.8.5.4 Strategies to Overcome Recondensation

It has been reported that for OrganoSolv and hybrid OrganoSolv/ionoSolv pretreatments that employ both organic alcohols and ILs, the alcohol used in the solvent systems can form new ether bonds at the α position, protecting the lignin molecule from recondensation and even improving its solubility (Scheme 10, route 4). Nevertheless, the levels of β-O-4 cleavage were comparable to that of pure IL pretreatments, with a 50% reduction in signal intensity compared to the native lignin. Conversely, the addition of organic solvents to the IL for pretreatment led to increased signal intensities of β–β and β-5 linkages, which hints to better preservation of resinol and phenylcoumaran units.^352^ Furthermore, increased signals of hemicellulose carbohydrates including xylose and arabinose were detected when the pretreated biomass was the grass *Miscanthus*, suggesting the preservation of LCC linkages. The lignin–carbohydrate bonds in *Miscanthus* connect ferulic acid/*p*-coumaric acid units on the lignin with hemicellulose arabinosyl units. However, when the same pretreatment methodology was applied to pine (a softwood), no carbohydrate signals were found, indicating the breakage of the lignin–hemicellulose linkages. It should be noted that for softwoods the linkage of hemicellulose with lignin happens via mannose units instead of arabinose like in grasses. Finally, when acetone was used as the organic component, it did not react with the lignin subunits but instead formed acetonide with selective *cis*-vicinal hydroxyl groups in the carbohydrate subunits. In certain acidic DES systems, carboxyl groups in the DESs (*e.g*., from LA) can undergo acylation at the γ-OH group of the lignin substructures (Scheme 12).^297^ It has been suggested that DESs containing boric acid (*e.g.*, [Ch]­Cl:boric acid), the formation of a cyclic ester between boric acid and the hydroxyl groups can protect the positions α and γ of the lignin molecules from oligomerization and repolymerization reactions, allowing the recovery of lignin fractions with low molecular weight and high level of preservation of β-O-4′, β–β′, and β-5′ bonds (Figure 35).^520^

**Scheme 12:**
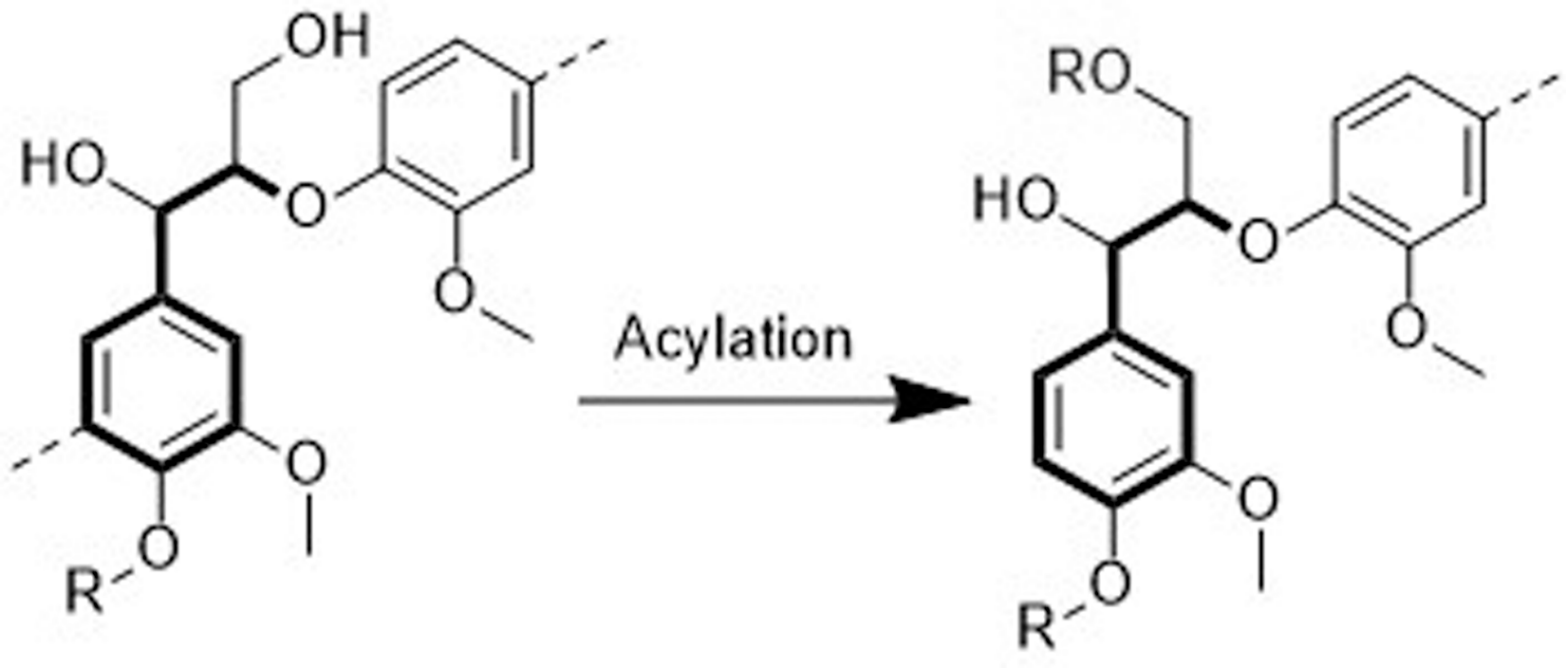
Proposed Reaction Pathway for Acylation of Lignin^297^

**Figure 35:**
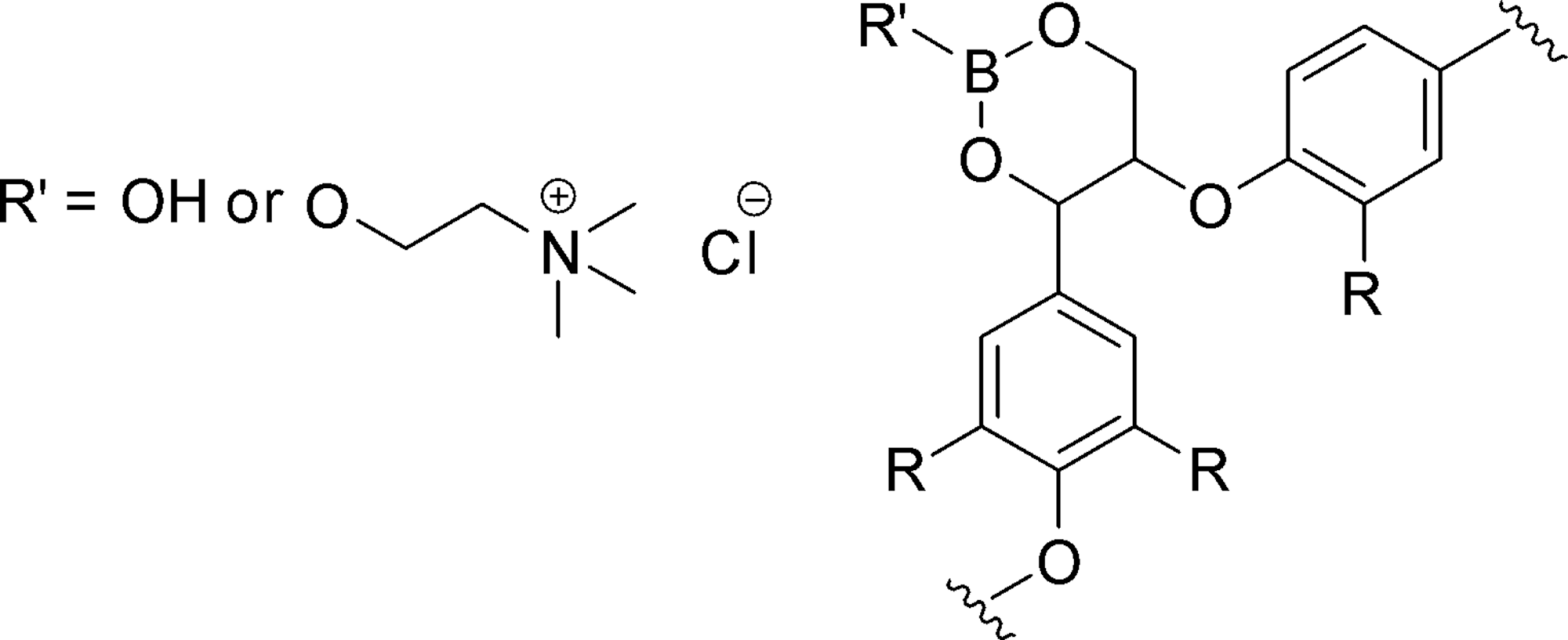
Cyclic ester formed between boric acid and lignin at the positions α and γ.

2.8.5.4.1. *Overcoming Formation of Humins by Autohydrolysis*. Since the hemicellulosic fractions are typically difficult to recover from the IL media, hampering the efficiency of the fractionation process and the presence of hemicellulose derived degradation products in the IL can lead to the formation of humins; it has been proposed that these issues could be tackled by the incorporation of an autohydrolysis stage prior to the delignification stage. Autohydrolysis allows the extraction of hemicelluloses from lignocellulosic biomass using only water, which makes it attractive from an economic and environmental perspective. Hemicellulose sugars are solubilized with the aid of hydronium ions from water and acetic acid released from the hemicellulose itself. This allows recovery of up to 90% of the hemicellulosic fraction.^347^

Several studies combining an initial autohydrolysis stage combined with a IL pretreatment report partial lignin recondensation during the autohydrolysis step at severe autohydrolysis conditions.^347,521,522^

SEM and confocal fluorescent microscopy imaging of *Eucalyptus* and pine before and after the autohydrolysis and IL pretreatment stages have been taken and compared by Rigual *et al*. (Figures 36–40).^522,523^ SEM shows that the raw, untreated materials had smooth surfaces without visible pores, while confocal fluorescent microscopy shows that the distribution of lignin and holocellulose on the surface is ordered and crosslinked. Autohydrolysis under mild conditions (150 °C) showed some degree of rearrangement of lignin and holocellulose in *Eucalyptus*,^522^ while intermediate conditions (175 °C) led to removal of hemicellulose only in the case of *Eucalyptus*, for which the surface is composed mainly of lignin. Harsher autohydrolysis conditions (200 °C) led to the agglomeration of small particles on the surface of the *Eucalyptus* pulps, where large numbers of pores were also formed. Furthermore, little lignin is detected, with the pulp consisting mostly of cellulose. On the other hand, for the pine pulps, mostly lignin is detected covering the surface of the pulp, confirming the recondensation and reprecipitation of softwood lignins.^523^ Both raw biomass types, *Eucalyptus* and pine, were also pretreated in [C_2_C_1_im]­[C_1_CO_2_] at 120 °C during 50 min using microwaves. In both cases, homogeneous macrostructures with observable porosity and modification of both lignin and holocellulose were observed, indicating partial dissolution and regeneration of wood fibres.

**Figure 36:**
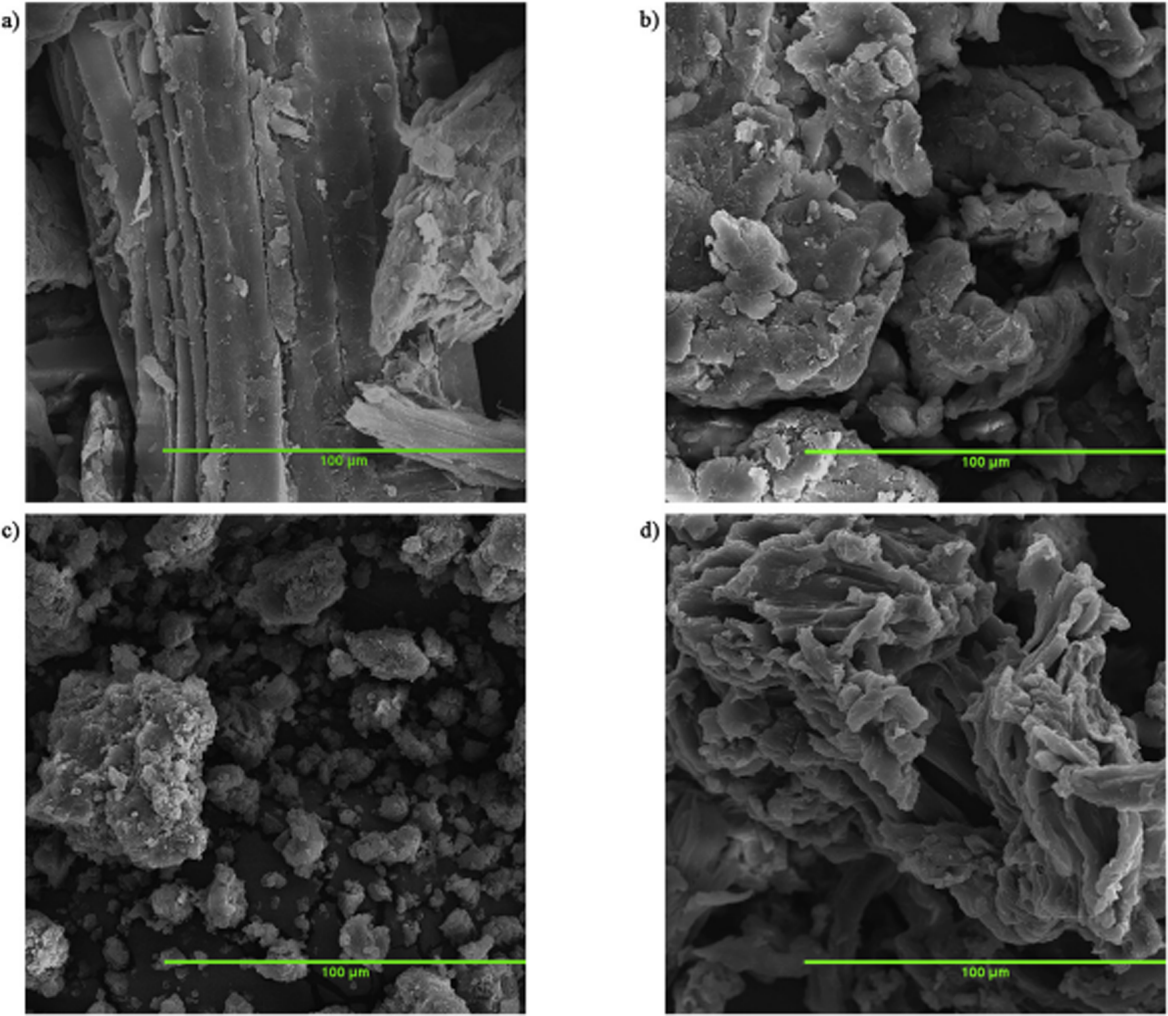
SEM micrographs of (a) untreated *Eucalyptus*, (b) *Eucalyptus* after auto hydrolysis at 175 °C, (c) *Eucalyptus* after auto hydrolysis at 200 °C, and (d) *Eucalyptus* after pretreatment with [C_2_C_1_im]­[C_1_CO_2_] at 120 °C. Adapted with permission from ref 523. Copyright 2018 Elsevier Ltd.

**Figure 37:**
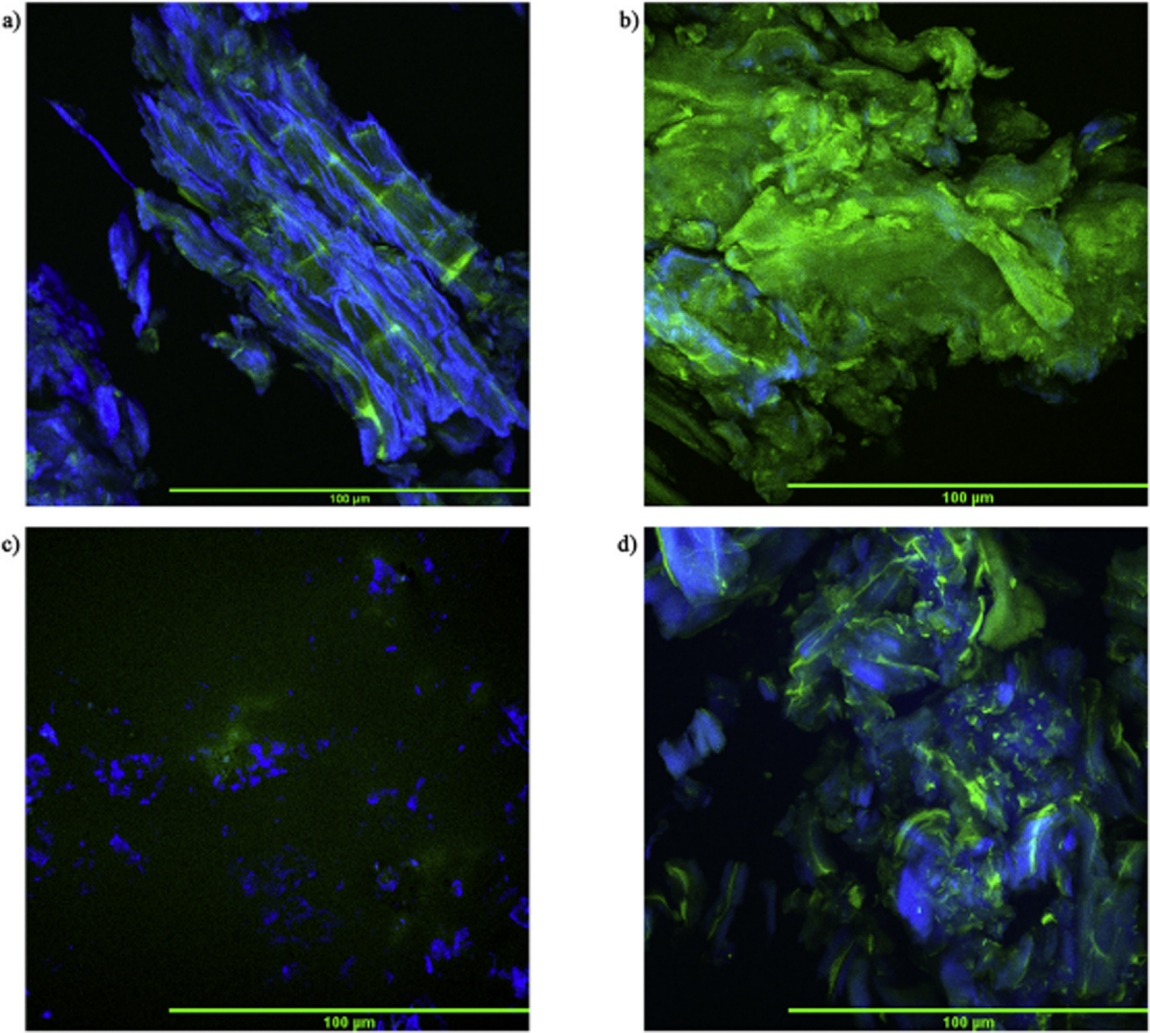
Confocal fluorescence microscopy images of (a) untreated *Eucalyptus*, (b) *Eucalyptus* after auto hydrolysis at 175 °C, (c) *Eucalyptus* after auto hydrolysis at 200 °C, and (d) *Eucalyptus* after pretreatment with [C_2_C_1_im]­[C_1_CO_2_] at 120 °C. Hemicelluloses (in blue) dyed with calcofluor are observed (Figures 37a and 39a) on the surface as well as lignin (in green). Adapted with permission from ref 523. Copyright 2018 Elsevier Ltd.

**38:**
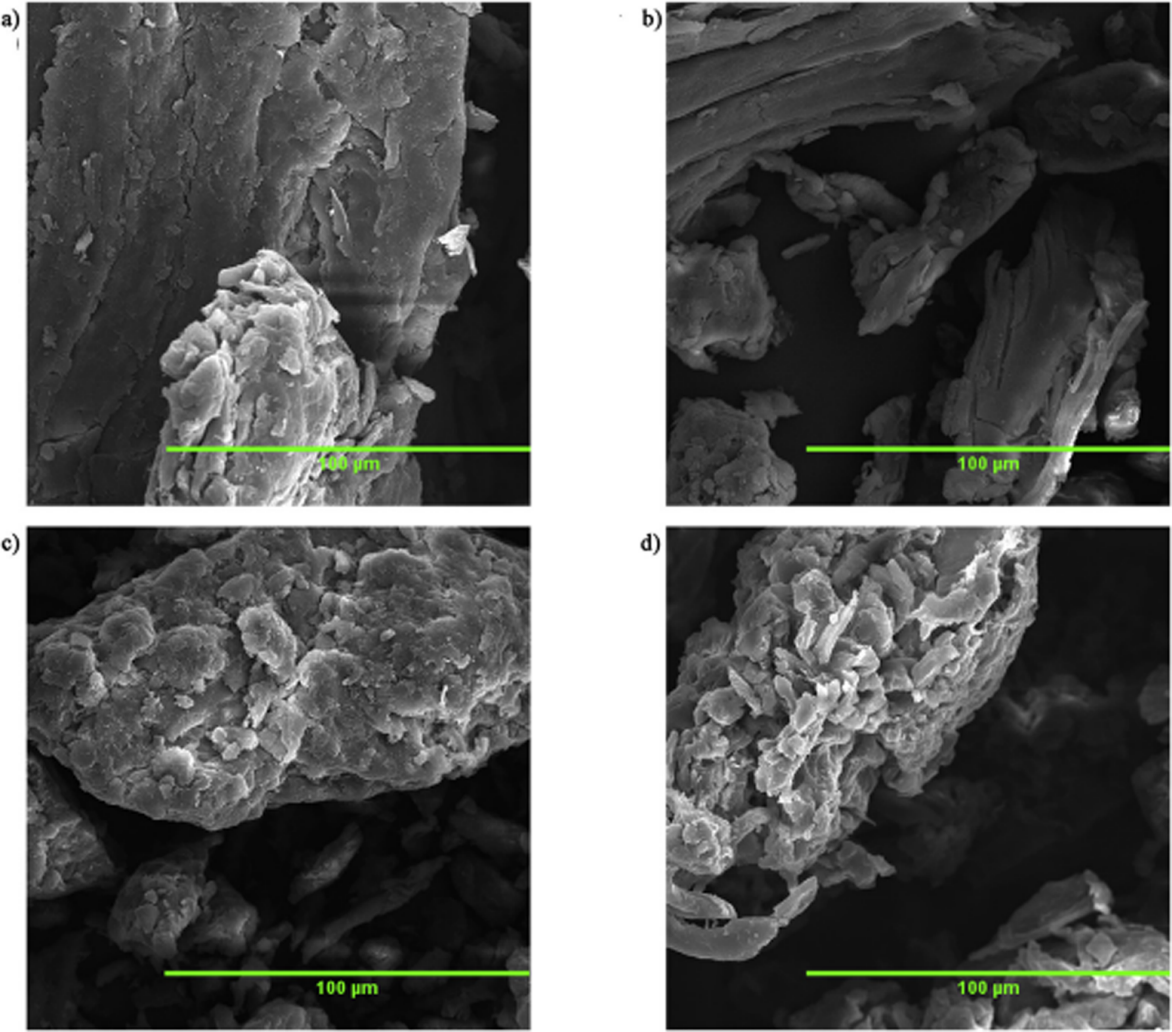
SEM micrographs of (a) untreated pine, (b) pine after auto hydrolysis at 175 °C, (c) pine after auto hydrolysis at 200 °C, and (d) pine after pretreatment with [C_2_C_1_im]­[C_1_CO_2_] at 120 °C. Adapted with permission from ref 523. Copyright 2018 Elsevier Ltd.

**Figure 39:**
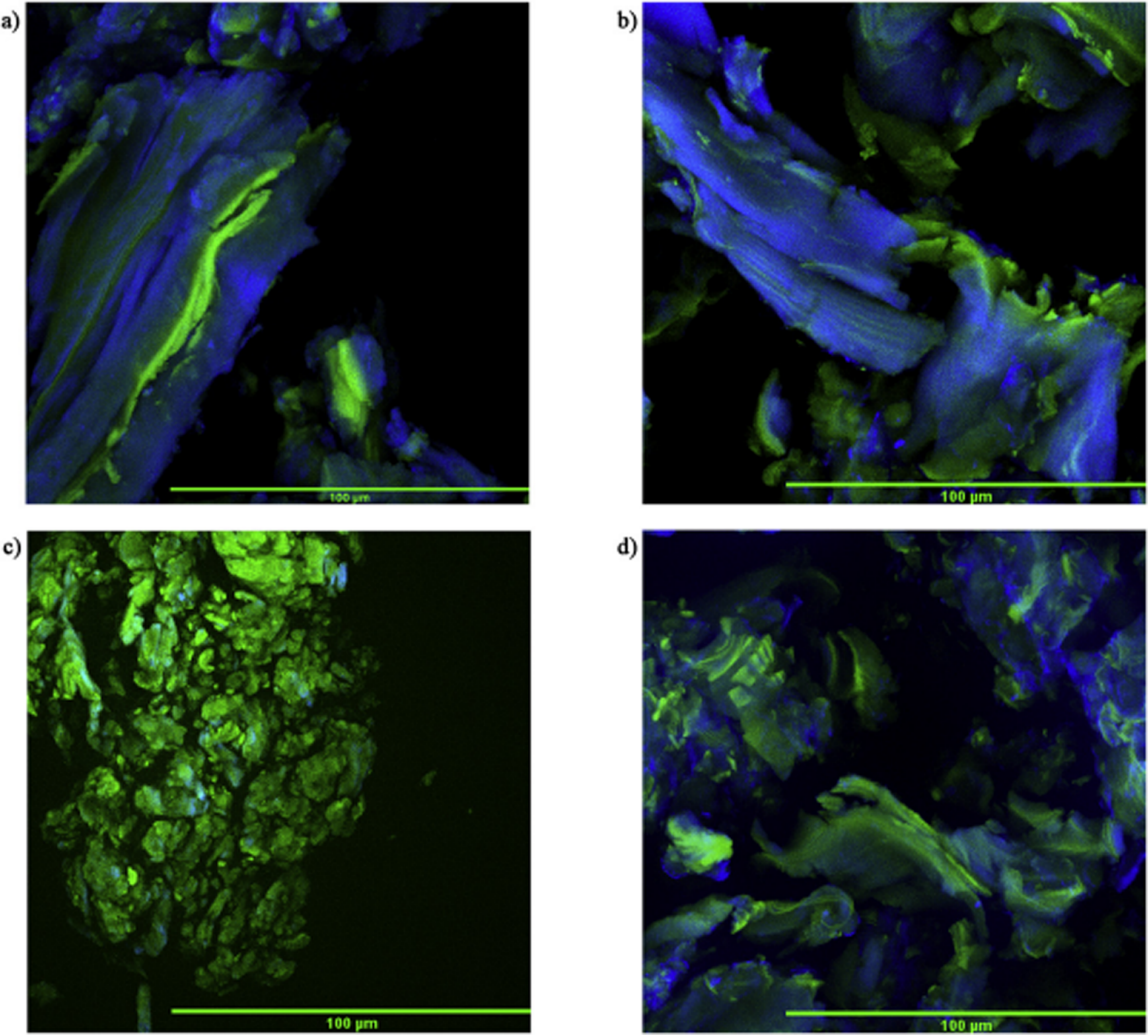
Confocal fluorescence microscopy images of (a) untreated pine, (b) pine after auto hydrolysis at 175 °C, (c) pine after auto hydrolysis at 200 °C, and (d) pine after pretreatment with [C_2_C_1_im]­[C_1_CO_2_] at 120 °C. Adapted with permission from ref 523. Copyright 2018 Elsevier Ltd.

**Figure 40:**
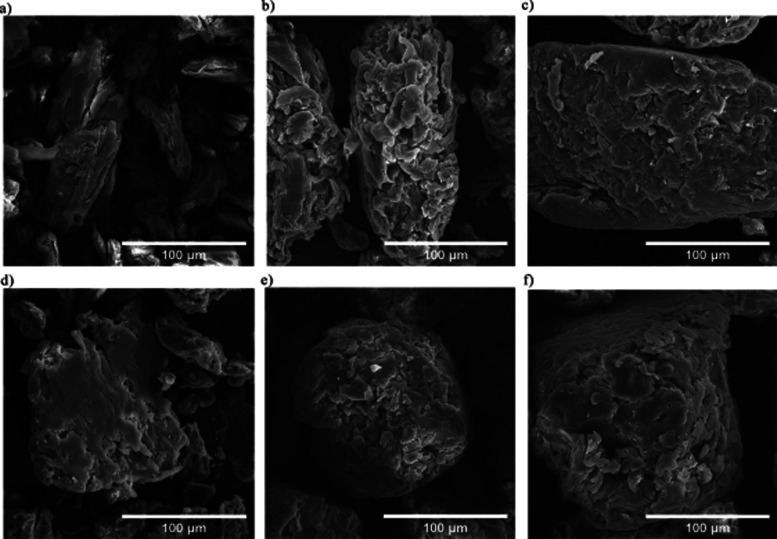
SEM microscopy images of (a) untreated pine wood, (b) pine pulp after autohydrolysis at 150 °C followed by pretreatment with [C_2_C_1_im]­[C_1_CO_2_] at 120 °C, (c) pine pulp after autohydrolysis at 150 °C followed by pretreatment with [C_2_C_1_im]­[C_1_CO_2_] at 150 °C, (d) pine pulp after autohydrolysis at 175 °C followed by pretreatment with [C_2_C_1_im]­[C_1_CO_2_] at 120 °C, (e) pine pulp after autohydrolysis at 200 °C followed by pretreatment with [C_2_C_1_im]­[C_1_CO_2_] at 80 °C, and (f) pine pulp after autohydrolysis at 150 °C followed by pretreatment with [C_2_C_1_im]­[C_1_CO_2_] at 150 °C.^521^ Adapted with permission from ref 521. Copyright 2019 Elsevier Ltd.

Further investigation on the effects of the combination of autohydrolysis and pretreatment with [C_2_C_1_im]­[C_1_CO_2_] on pine showed that pretreated particles grew in size and had different morphology, with significant increase in porosity with respect to the feedstock particles due to the dissolution, followed by regeneration when mild autohydrolysis conditions were employed. However, when harsh conditions were employed (150 °C in both stages), a very closed structure with no visible pores was recovered (Figure 40).^521^

Treatment of *Eucalyptus* with a two-stage process consisting of an autohydrolysis stage with a residence time of 30 min followed by ionoSolv pretreatment with [C_1_im]Cl at 135 °C found the optimal lignin recovery and cellulose purity with a autohydrolysis temperature of 184 °C and ionoSolv residence time of 3.5 h.^347^ It was observed that *M*_w_ of the recovered lignins increased with ionoSolv time, due to recondensation reactions. At short ionoSolv times, increase in *M*_w_ was also observed with increasing autohydrolysis temperatures, probably due to the recondensation of lignin on the surface of the autohydrolyzed material, so the lignin extracted during the ionoSolv stage was already recondensed. However, PDI does not depend on the autohydrolysis temperature, as the autohydrolysis alters the *M*_n_ as it does with the *M*_w_. This suggests that the autohydrolysis stage leads to the deposition of recondensed and heterogeneous lignin particles on the surface of the pulps. The autohydrolysis step had no influence on the S/G ratio at the temperature conditions employed.^347^

#### 2.8.6 Lignin Recovery and Fractionation

##### 2.8.6.1 Recovery of Lignin from ILs and DESs

Lignin can be recovered from the pretreatment liquor using an antisolvent, usually excess water, an organic solvent, or mixtures of water and another solvent. The addition of the antisolvent decreases the solvation power of the IL allowing for lignin precipitation. Different strategies need to be developed for dissolution-type processes where both cellulose and lignin are dissolved on the IL liquor and ionoSolv type processes where only the lignin is dissolved while the cellulose remains as a solid pulp.

For liquors from dissolution-type processes, two-stage recoveries are the most efficient separating cellulose and lignin. This type of process involves a first stage where only the cellulose is precipitated using a mixture of organic solvents or an organic solvent and water, followed by a second stage where lignin is precipitated. Mixtures containing at least one protic solvent are generally preferred. Using a mixture of an organic solvent (*e.g.*, acetone) and water allows the selective precipitation of cellulose. Once the cellulose has been separated, the evaporation of the organic component resulted in the precipitation of lignin from the IL-water mixture.^39^ Control of the organic solvent:water ratios can also allow for lignin partitioning, producing lignin fractions that are separated by their molecular masses into different streams.^25^ The separation of lignin from alkaline ILs as [C_1_C_2_im]­[C_1_CO_2_] often requires neutralization of the IL prior to the lignin precipitation stage. This can lead to issues related to acid accumulation on the IL if the IL is to be recycled. Although this could be overcome by neutralization of the acid, this raises economic and environmental concerns.

Lignin in ionoSolv liquors is usually recovered by the addition of water as an antisolvent, which causes lignin precipitation. For precipitation from ILs based on the [HSO_4_]^–^ anion a ratio of 3 g of water per g of the IL–water mixture (referred to as “equivalents”) is usually employed. However, due to the energy expenditure needed to remove the water from the IL for its recycling, minimizing the amount of water used for lignin reprecipitation is of key importance for the successful implementation of biorefineries based on ILs for biomass fractionation. Abouelela *et al*. have reported that reducing the water equivalents by half, from 3 to 1.5, had virtually no impact when precipitating pine lignin from [C_4_C_1_C_1_N]­[HSO_4_]_80%_, with recoveries of around 80 wt% in that range. Further reductions of the water equivalents of led to a sharp decrease in the lignin yields (Figure 41).^476^

**Figure 41:**
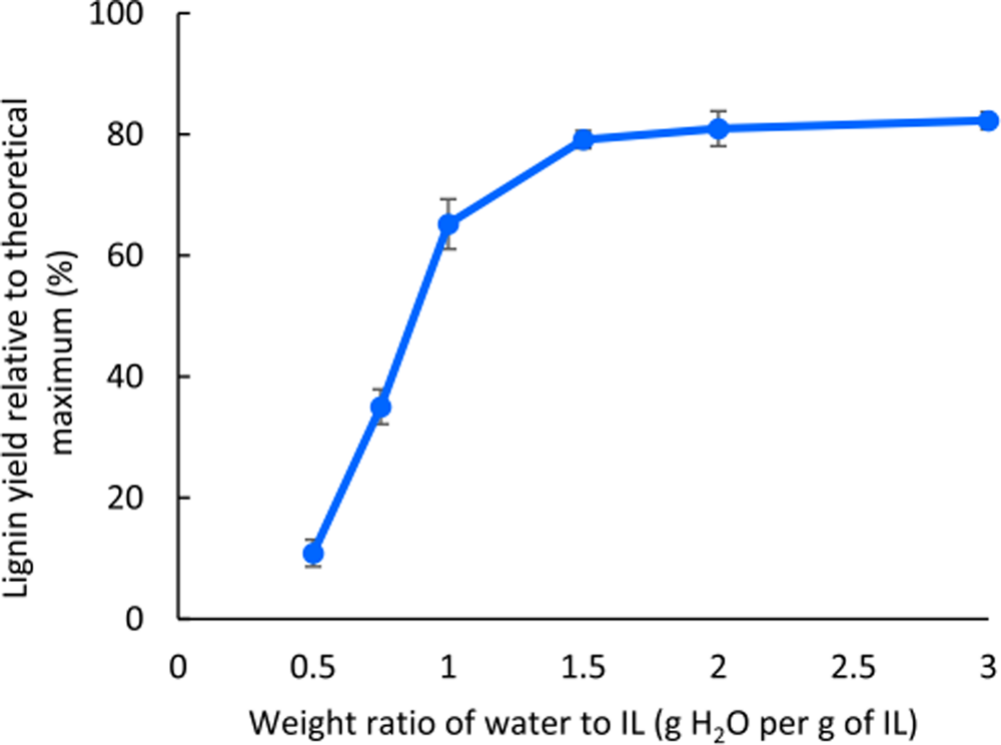
Lignin yield recovered at different water equivalents. Fractionation experiments were conducted on pine using [C_4_C_1_C_1_N]­[HSO_4_]_80%_ at 170 °C for 30 min using a 1:5 g·g^–1^ solid loading. Adapted with permission from ref 476. Copyright 2021 American Chemical Society.

Furthermore, lignin precipitated using water equivalents in the range of 1.5–3.0 showed the same structural characteristics. On the other hand, the lignin fractions recovered using lower amounts of water showed increasing degrees of condensation, suggesting that more condensed and crosslinked lignin fragments are more prone to precipitation upon the addition of small water equivalents.^476^ Exploring this effect further, Chambon *et al*. showed that sequential antisolvent addition allows for lignin fractionation, where the molecular weight of the recovered lignin polymers can be controlled by tuning the amount of water added to the IL liquor (Figure 42).^366^ Addition of minimal water volumes resulted in the isolation of fractions with high *M*_w_, PDI, thermal stability, and *T*_g_ (178 °C). Further addition of water allowed the separated isolation of lignin fractions that were more monodisperse, with high phenolic and total hydroxyl content and lower thermal stabilities and *T*_g_ (136 °C).^366^

**Figure 42:**
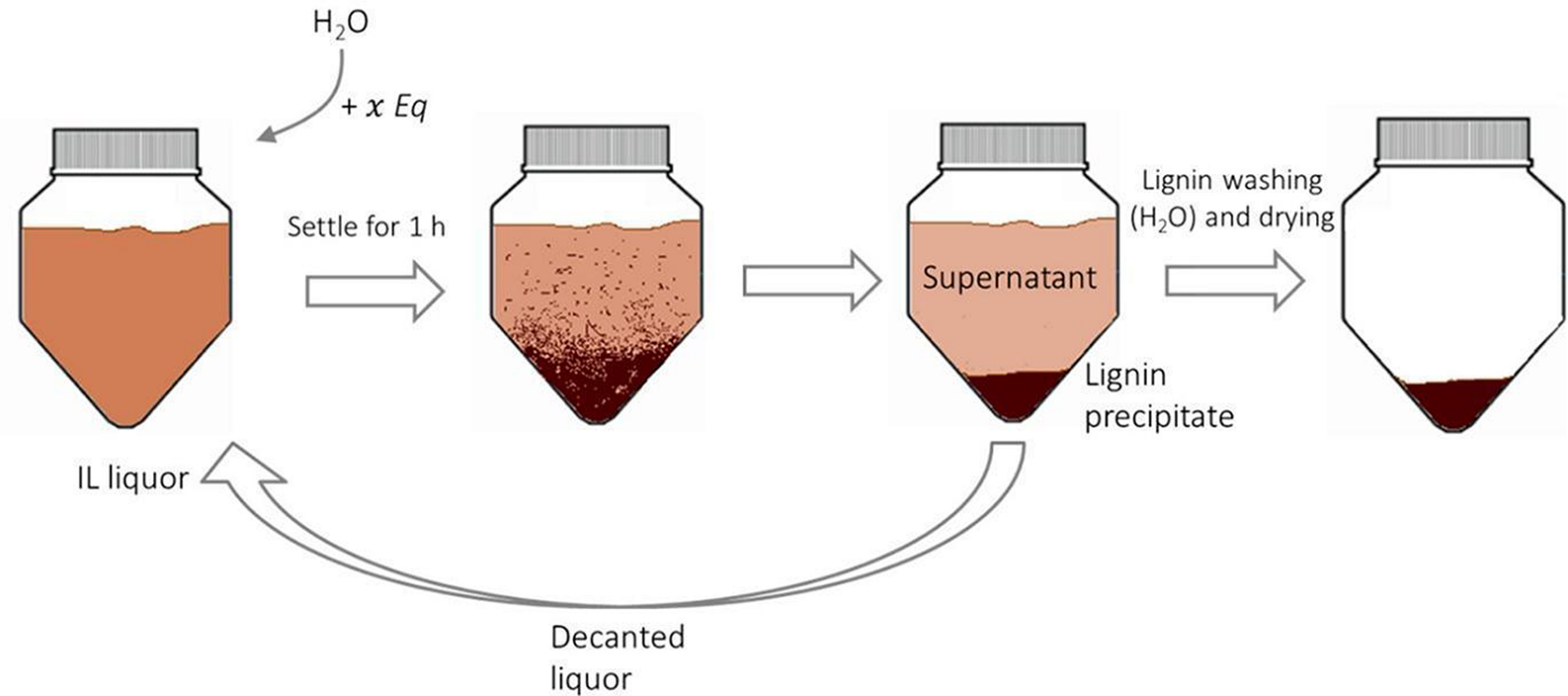
Lignin fractionation process by which lignins were precipitated and recovered using sequential antisolvent addition. Adapted with permission from ref 366. Copyright 2021 American Chemical Society.

The mass balance of lignin fractions precipitated after the addition of different amounts of water for *Miscanthus*, willow, and pine pretreated with [C_4_C_1_C_1_N]­[HSO_4_]_80t%_ showed that for *Miscanthus* and pine, lignin precipitation started after the addition of 0.5 equiv of water and 90 wt% of lignin had precipitated after the addition of 1 equiv of water, while for willow larger amounts were needed (Figure 43). The difference could be due to the high content of S units in willow lignin which results in less condensed and less hydrophobic structures.^366^

**Figure 43:**
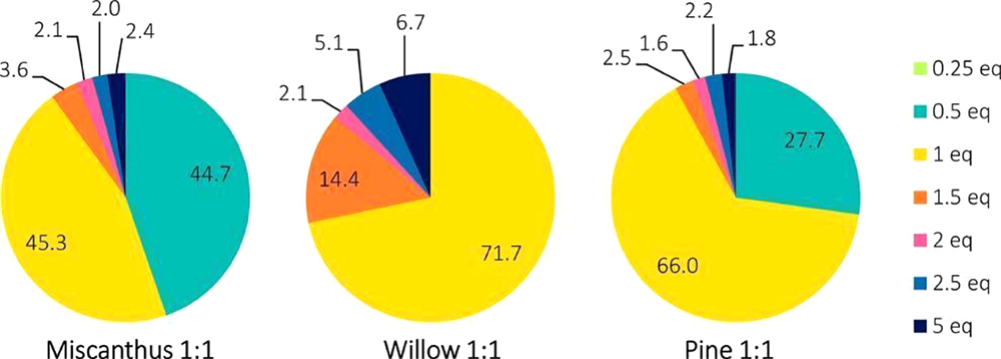
Mass balance of lignins isolated by sequential precipitation after fractionations of *Miscanthus*, willow, and pine with [C_4_C_1_C_1_N]­[HSO_4_]_80t%_ at 150 °C for 120, 90, and 90 min, respectively. Adapted with permission from ref 366. Copyright 2021 American Chemical Society.

Lignin fractionation is mostly determined by molecular weight, with a marked shift toward lower molecular mass material with increasing antisolvent addition. Nevertheless, small molecular mass species also precipitated with the largest molecules, producing fractions with high polydispersities. In contrast, fractions recovered after the addition of larger proportions of water showed narrow molecular weight profiles (Figure 44).^366^ Overall, sequential addition of water allowed the isolation of different lignin fractions that were more homogeneous than lignin obtained from a single precipitation stage. The combination of sequential fractionation of lignin with further process control opens the possibility of tuning up lignin properties, recovering more monodisperse lignin fractions with better properties for specific applications. Fractions with higher *M*_w_ and thermal stability could be used as feedstocks to produce carbon fibers, while fractions with low *M*_w_ and PDI could be aimed for polymeric or adhesive applications.

**Figure 44:**
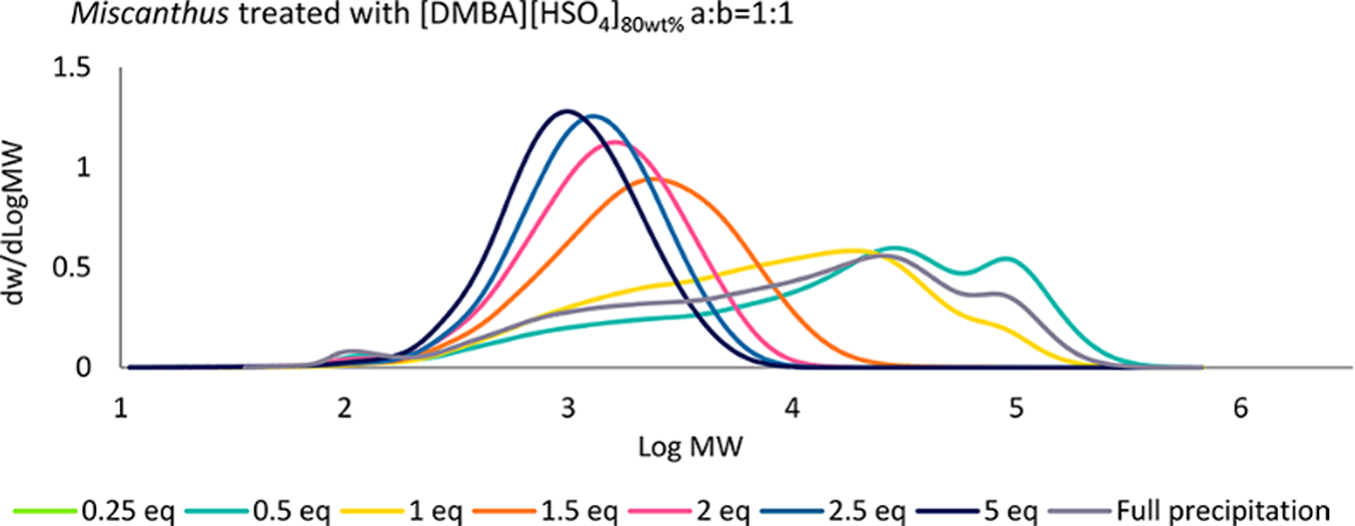
Molecular weight distribution of full precipitation and sequential fractionated lignin as a function of water amount added for *Miscanthus* lignins with [C_4_C_1_C_1_N]­[HSO_4_]_80wt%_. Adapted with permission from ref 366. Copyright 2021 American Chemical Society.

##### 2.8.6.2 Lignin Washing

Lignin recovered from IL liquors by the addition of an antisolvent usually containing non-negligible amounts of IL. Lignin fractions recovered from more acidic pretreatments tend to show more IL contamination. Washing with water has been proven insufficient to remove all IL from lignin precipitates.^338^ In the case of pretreatments with DESs based on [Ch]­Cl, co-precipitation of DESs with lignin has been observed upon water addition. To reduce the DES lost in the lignin fraction, the use of mixtures of solvents has been studied, and it has been reported that water:ethanol mixtures in the range of 90:10 to 60:40 v:v can reduce the DES coprecipitation and yield lignins with good purity (∼95%) after two washing cycles with the same mixture.^411^

##### 2.8.6.3 Lignin Recovery after Saccharification from One-Pot Processes

One-pot pretreatment and saccharification process enables a facile recovery of lignin by simple solid–liquid separation.^524^ In a typical one-pot process, the enzymatic saccharification is performed sequentially after the pretreatment step. Most cellulase and hemicellulose enzyme cocktaILs employed have an optimum operating pH of 5 usually achieved by adjusting pH of the pretreated slurry with an acid or an alkali.^330,332^ Owing to the p*K*_a_s of lignin, most of the polymeric lignin remains insoluble in the aqueous solution and is recovered by simple solid–liquid separation. Depending on the biomass employed and process intensity, the purity of the lignin obtained from one-pot process can range from 30% to 80%. It should be noted that pretreatment with IL/DES can alter the structure and molecular weight of the lignin in biomass depending on the nature of IL/DES and/or process severity. A wide molecular weight distribution can be realized for such lignin fractions with comparatively lower weighted average molecular weight are obtained due to limited or negligible recondensation into higher molecular weight fragments (Table 3).^524,525^

**Table 3:** Molecular Weight Distribution of Lignin Fractions after One-Pot Process

sorghum type	*M*_w_ (Da)	*M*_n_ (Da)	PDI
WT	780 ± 19	235 ± 6	3.3 ± 0
engineered	749 ± 11	206 ± 5	3.6 ± 0

### 2.9 Valorization of Biomass via the Conversion of Carbohydrates into Furan Building Blocks

#### 2.9.1 Furan Building Blocks

Furan building blocks are chemical compounds that have the potential to be used on a large scale in many applications, including the production of polymers, surfactants and commodity chemicals.^526,527^ They represent the most valuable options for providing environmentally friendly chemical aromatics to replace the fossil fuel-derived analogues of benzene, toluene and xylene that now dominate the plastics and surfactants markets (Figure 45).^527–529^ It has been shown that the substitution of furans for aryl moieties is not only environmentally friendly, but can also bring interesting properties in the end-use applications.^530–532^ For example, substituting the benzene ring in polyethylene terephthalate (PET) with a furan ring leading to polyethylene furoate (PEF) results in a plastic resin with better gas permeability and glass transition temperature, making it more suitable for recycling and long-term food storage.^533–535^ Other examples include the use of biosurfactants as an aromatic component, which can give better hard water properties and solubility when used in detergents.^534,536^

**Figure 45:**
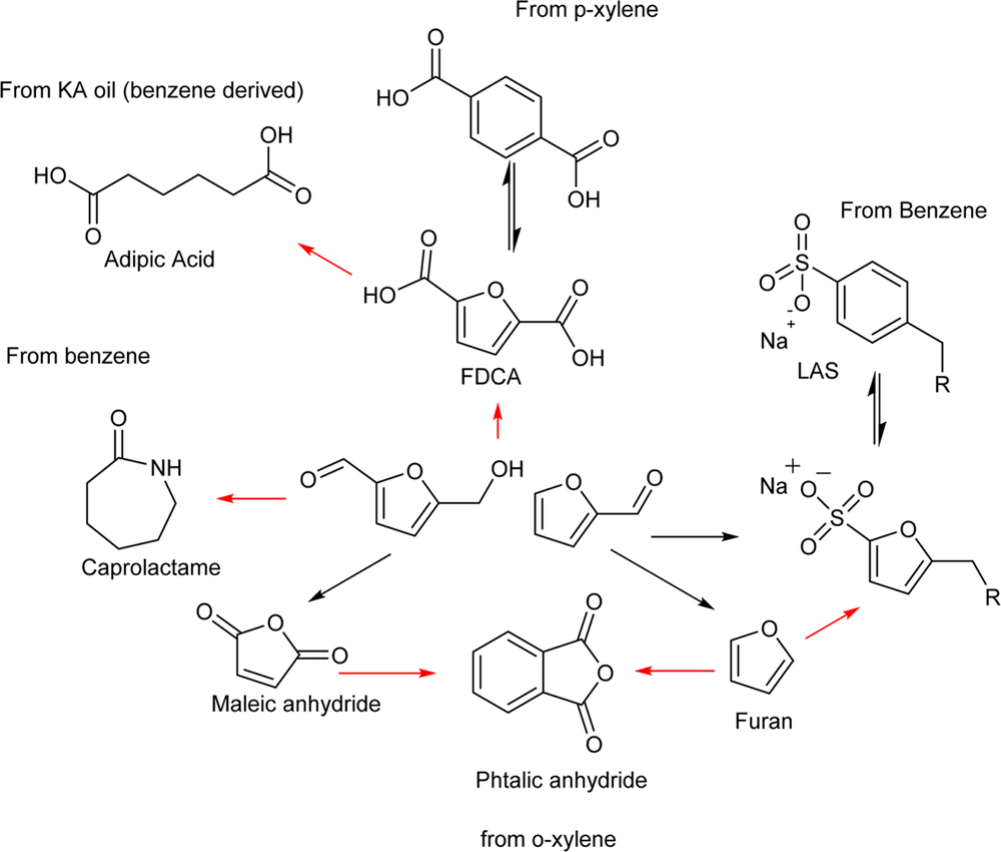
Chemical paths to substitute different fossil fuel-based chemicals with furan based. Adapted with permission from ref 527. Copyright 2023 Royal Society of Chemistry under CC BY 4.0 (https://creativecommons.org/licenses/by/4.0/).

The conversion of biobased furans requires the implementation of processes that can perform the conversion of sugars with high yield and selectivity, while respecting the engineering principles of waste minimization and solvent and catalyst recyclability.^526,537,538^ The major furan building blocks are 5-hydroxymethylfurfural (HMF) and furfural, which are derived from the dehydration of the C5–C6 sugar components of hemicellulose and cellulose.^531,532,539^ They can be used as starting material in the synthesis of a variety of products that can substitute many aromatic fossil fuel analogues for different applications (Figure 45). In this section, we review the use of ILs for the preparation of these molecules from lignocellulose, highlighting how the use of ILs can favour their synthesis and conversion.

#### 2.9.2 Furfural

Furfural is obtained from the hydrolysis of the pentose fractions of biomass and represents one of the few examples where the production of a compound from biobased sources is more economically feasible than petro-based raw materials. Overall, the traditional processes to obtain furfural are considered quite efficient, making it an ideal product to implement in a biorefinery.^540,541^ The first technologies to produce furfural were established in 1921 with the Quaker Oats process, characterized by high reaction yields through acid and high efficiency in product separation.^540,542^ To maximize productivity, biomass with a high content of hemicellulose is desirable, such as corncobs and sugarcane bagasse, the main feedstocks to produce furfural nowadays.^543–546^ It is used in applications such as lubricating oil recovery, as monomers for polyfurfuryl alcohol and as a chemical intermediate for various applications (*e.g.*, preservatives and pharmaceuticals).^547,548^

Nevertheless, its application on a large scale in the commodity product sector is not yet fully established.^549,550^ One reason is the low functionalization of the furan ring, compared to HMF, which makes it unsuitable in the polymer field. On the other hand, many efforts are directed in establishing new chemical reactions that can span more applications, especially in the production of surfactants.^536,538,551,552^ Another important drawback of the traditional processes to produce furfural is the production of excessive amounts of acidic wastewater, which needs to be treated to be disposed safely in the environment. While this aspect does not represent a major concern for the current furfural supply chain due to its small production scale (400 ktons/year, with few plants with capacity over 20 ktons/year), it can represent a barrier for future implementation in larger scale for the commodity chemical industry.^526^ In this scenario, the implementation of a furfural production technology which can avoid the formation of wastewater will possess strong environmental benefits. Examples using ILs for this purpose have been reported in literature with major focus on parameters optimization.^553,554^ Several studies report advantages in using ILs as Brønsted acid catalysts in water systems, but these are beyond the scope of this review as this approach does not offer advantages in terms of process economics.^554,555^

From a chemical point of view, the use of a solvent which can dissolve the biomass, or at the very least the hemicellulose fraction, could lead to more favorable kinetics due to reduced mass transfer limitations.^556,557^ However, no demonstrated advantages in this area have been reported to date, despite many mechanistic studies on this reaction. Control studies have shown that the addition of [C_4_C_1_im]Cl or [C_4_C_1_im]Br to produce furfural from xylose as substrate in dimethylacetamide (DMA) is not efficient, compared to when these were used to produce HMF from glucose and fructose. This indicates that different phenomena are in place for the dehydration process.^558^ Studies of the salt effect in water have shown that small cations such as [Li]^+^ and large anions such as I^–^ direct the reaction towards a more favourable pathway passing through the intermediate 1,2-eneldiol, establishing a limitation in the use of ILs.^559–561^ Indeed, the use of different imidazolium based ILs with [HSO_4_]^−^ and Cl^–^ led to yields below 50% and catalytic systems which were efficient for glucose dehydration (1-ethyl-3-methylimidazolium bromide, [C_2_C_1_im]­Br, and SnCl_4_) resulted in lower yields when xylose was used as a substrate.^562–566^ Applying microwave heating at low substrate concentrations improved the efficiency up to 90% from hemicellulose, but yields from biomass remained poor (<15%).^567^ Functionalization of the cation with hydroxy groups improved xylose conversion, enabling operation at high loading (>50%).^568^ While various studies have looked at the optimization starting from xylose, the conversion of raw biomass has so far proved to be unsatisfactory.^569^ A recent publication reported the use of a glycine based IL which can achieve 80% yield in a two-step reaction starting from biomass, but microwave heating and extracting organic solvent are required.^570^

Overall, the advantages of converting biomass to furfural in ILs needs to be further explored and more studies are needed to demonstrate important aspects such as solvent reusability and minimization of by-products to ensure further development of the technology.

#### 2.9.3 HMF

HMF is obtained from C6 sugars, with a reaction that is catalyzed by acids. Salts and solvent compositions have a strong influence on the selectivity of the reaction. Therefore, the implementation of ILs, with tunable chemical properties, can be potentially advantageous.^571–573^

Due to its high market potential, HMF is often referred to as a “sleeping giant”. The main application of HMF is the production of FDCA, used to produce polyethene furoate polymers that can replace PET.^574,575^

Although the conversion of HMF into 2,5-furandicarboxylic acid (FDCA) is seen as an easily achievable reaction, little commercial activity has followed. This is mostly due to difficulties in the isolation of HMF, its instability during the reaction and in its isolated form, difficulties in establishing an environmentally friendly process and low versatility in the sugar substrate used.^576,577^ Indeed, while furfural can be produced efficiently starting from raw biomass, the production of HMF can only be achieved efficiently using fructose, questioning the sustainability of the process (food chain impact) and setting limits on the economic competitiveness.^578^ Much R&D has been addressed in developing processes based on cheaper substrates such as biomass, cellulose and glucose, but often these are not selective, compromising the recycling of the catalytic system.^579^ Moreover, HMF undergoes too many side reactions, which can lead to humin formation through self-condensation, condensation with the substrate or intermediates, and overdehydration to levulinic and formic acid.^580,581^

The use of ILs in this field has given an outstanding boost for the overall transformation of sugar substrates compared other systems based on water and organic solvents, thanks to the suppression of side products, the stabilization of HMF and the high dissolution ability of ILs.^539^

##### 2.9.3.1 Dehydration of Fructose

ILs are very efficient when fructose is used as a substrate. They allow higher substrate loading, maximizing productivity per solvent used, which is their biggest advantage over water and organic solvent-based systems.^537,582^ From a chemical point of view, ILs can provide different interactions, which can favour the overall kinetics and thermodynamics of the dehydration, thus enabling high selectivity at high substrate loading.^583–585^ Many studies have analyzed the nature of the benefits in using ILs for this type of reaction. According to the solvent design strategy, the ILs can behave as both solvent and catalyst. Optimum cation and anion choices can strongly favour this reaction, with solvents such as 1-butyl-3-methylimidazolium bromide ([C_4_C_1_im]­Br), reported to carry out this transformation at temperatures lower than 100 °C at 30% substrate loading with yields over 90% without the addition of any acid, even if the system proved to be strongly affected by water.^586^ This could be further improved using the pyridinium IL 1,10-decane-1,10-diylbis­(3-ethylpyridinium) dibromide ([(C_2_^3^py)_2_^1,10^C_10_]­Br_2_) which can catalyze the dehydration resulting in 89% yield at a molar loading of 1:1 with the IL.^587^

Catalytic systems more tolerant towards water content can be designed using chloride anions, but these require an acid catalyst or microwave heating.^586,588^ Dehydration in [C_4_C_1_im]Cl can achieve yields over 80% at a fructose loading of 40%, which distinguishes ILs from organic solvents or aqueous systems which generally require lower loadings or the addition of an extracting liquid.^589^ Simulations and spectroscopic studies performed in a system formed by the ILs [C_1_^2^Pyrro]­[C_1_SO_3_] and [C_4_C_1_im]Cl have shown that acid is involved in the first step of dehydration. When [C_1_^2^Pyrro]­[C_1_SO_3_] was not present, the first intermediate was not detected, while it formed very fast in its presence even in the absence of [C_4_C_1_im]­Cl, although in the absence of [C_4_C_1_im]Cl the conversion of the first intermediate to the next intermediate was very slow. When both ILs were present, the formation of the second intermediate was enhanced and its transformation to HMF was fast. The data collected by the authors suggests that the [C_1_^2^Pyrro]^+^ cation is the main catalyst of the dehydration of fructose.^585^ Generally, for the dehydration of fructose, the cation needs to have H-bond donor ability while the anion acts as an H-bond acceptor, to allow an optimum coordination with the hydrogen and electron doublets in the hydroxy group (Figure 46).^583,585^ On the other hand, the anion has a major role in defining the selectivity. The substitution of Cl^−^ or Br^−^ anions with triflate and bis-triflimide resulted in a drop in yield of over 80%, while changing the anion from butyl to ethyl imidazolium, which increases the energy of the interaction of the cation with the hydroxyl group by twofold, resulted in a change in yield of only 10%.^590–592^ Cations and anions participate in the second and third steps and these interactions are further favoured by the B-fructofuranose conformation that fructose adopts in ILs with Cl^−^ or Br^−^ anions.^585^

**Figure 46:**
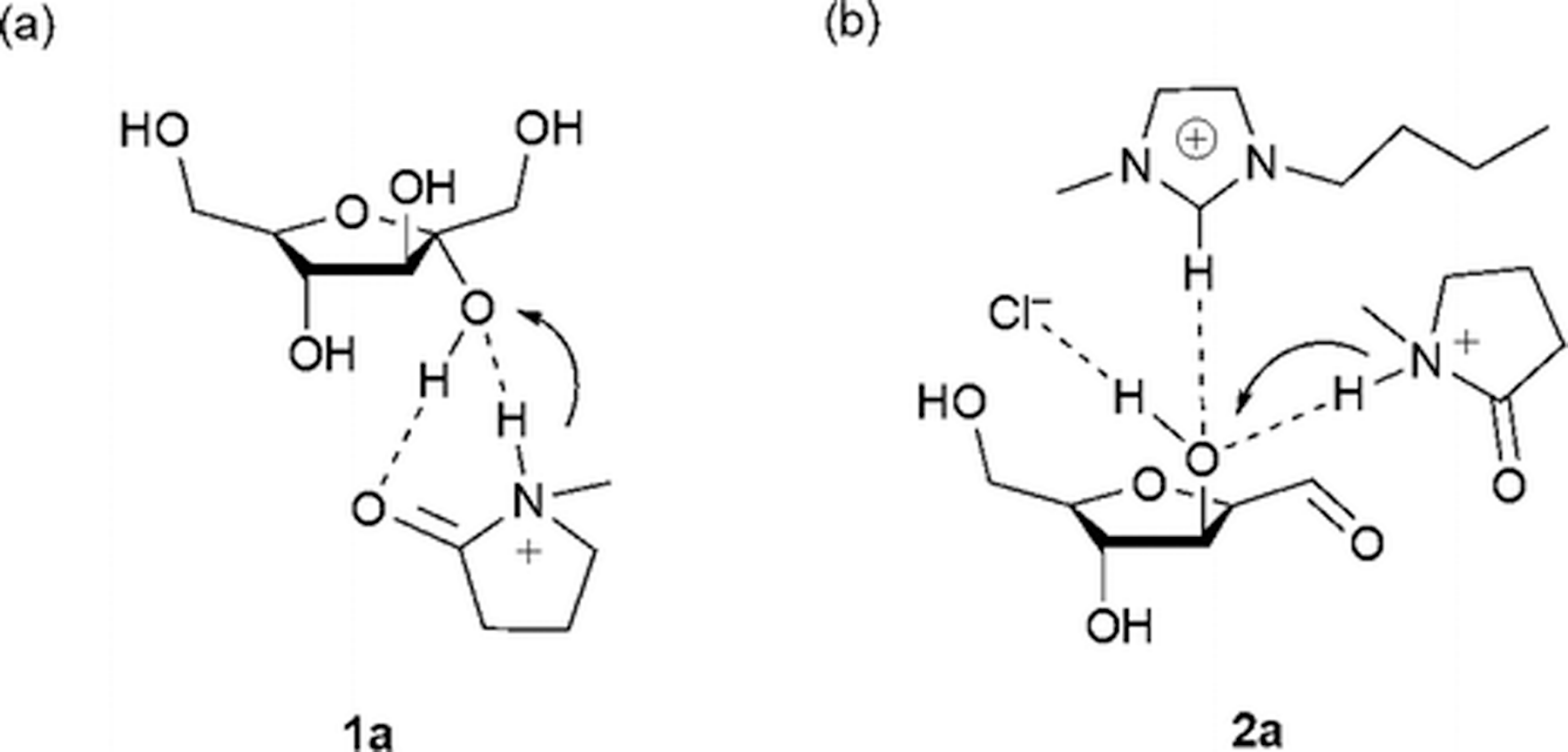
Coordination of the anion, cation, and Brønsted acid catalyst with the hydroxyl group of fructose.^585^

Protic ILs, such as [C_1_im]­Cl, led to high yields of over 90% at high fructose loading (30%) in short reaction times (30 min) at 90 °C.^593^ Other studies tried to modify the physicochemical properties by mixing different ILs. The mixture of *N*-methyl-2-pyrrolidonium methylsulfonate ([C_1_^2^Pyrro]­[C_1_SO_3_] and [C_4_C_1_im]Cl with 86% molar composition of Cl^–^, decreased the melting point of [C_4_C_1_im]Cl to r.t. while obtaining over 90% yield.^594,595^ The reaction proved to be less efficient when the acidity is provided by the anion. The IL [C_4_C_1_im]­[HSO_4_] led to low yield mainly due to the instability of HMF in this solvent, but the reaction could be improved by the addition of an extracting solvent (methyl isobutyl ketone, MIBK) or a co-catalyst as CrCl_3_ which has been reported to impart stability to HMF.^594,596^

The high efficiency of the Brønsted acid catalysis with Cl^–^ anions allowed the implementation of switchable solvents systems based on [Ch] Cl/CO_2_, exploiting the inhibitory effect of CO_2_ towards forming oligomers and permitting the catalyst removal through pressure reduction. This system was able to improve the HMF yield up to 72% for over 100% fructose loading at 40 bars (1:1 molar).^597–599^ Other H donor agents such as Amberlyst and heteropolyacids also provide high efficiency in dehydration, allowing to achieve better separation of the catalyst in combination with high yields (over 80%) with high loading (>10%).^600–603^

The transformation of fructose in ILs forming weak or no H-bonds was also studied. Notwithstanding the thermodynamically unfavorable conditions, the possibility of carrying out this reaction in ILs containing [PF_6_]^–^, [BF_4_]^−^, [CF_3_SO_3_]^−^ and [(CF_3_SO_2_)_2_N]^−^ is desirable, as the separation of HMF can be carried out more efficiently.^582,590,591,604,605^

Vanadium phosphate proved to be an efficient catalyst when 1-butyl-3-methylimidazolium bis­(trifluoromethylsulfonyl)­imide, [C_4_C_1_im]­[(CF_3_SO_2_)_2_N], achieved a yield of 81% in a biphasic solvent system with water.^604^ In the hydrophilic IL 1-butyl-3-methylimidazolium triflate, [C_4_C_1_im]­[CF_3_SO_3_], the optimization of the water content at low concentration (3.5%) was necessary to guarantee high reaction efficiency. This allowed the loading to be increased to 14%, and the yield remained above 80% in only 10 minutes with catalytic amounts of HCl. As only HCl was suitable as a catalyst, this confirmed that the reaction requires a coordinating anion to carry out the reaction efficiently.^605^

Other metal catalysts based on Ru, Pt, Fe, Al, Ge, and Cr in chloride-based ILs have also been tested, but in general the yield is lower compared to Brønsted acid-based ILs, and the effect varies depending on the metal used, which is affected differently by the anions.^606,607^ Chromium chloride proved to be more efficient when [HSO_4_]-based ILs were used, while a ligand was required when [C_4_C_1_im]Cl was used.^594^ A mechanistic study carried out in [C_4_C_1_im]Cl analyzed the influence of the Lewis acidity of the different corresponding metals on the conversion of fructose to HMF and showed that NbCl_5_ gave the highest yield, indicating that there is an optimal Lewis acidity to ensure high selectivity.^608^ However, this evaluation can be optimized differently depending on the anions and reaction conditions used.^609,610^

##### 2.9.3.2 Glucose Conversion

The conversion of glucose requires a system capable of both isomerization of glucose to fructose and dehydration. Although both steps are catalyzed by a Brønsted acid, one-pot yields using this catalyst are typically low because of the occurrence of side reactions that usually result in humic substances.^606,611^ Brønsted acid catalysis also proved inefficient when ILs were used, requiring a more complex approach to improve the catalytic selectivity of the process.^606^ However, selectivity can be enhanced exploiting the high boiling point of ILs. For example, by using the Brønsted acid IL [C_1_im]­[HSO_4_] as solvent and catalyst, and applying vacuum (1 mbar), high T (180 °C) and water stripping, the yield of the process could be increased from 17.3% to 76.1%, although the glucose concentration was low.^612^

ILs can activate Lewis acid catalysts such as CrCl_3_ and SnCl_4_ by forming metal anion complexes leading to higher yields without complex systems. Their planar configuration and the optimal weak interactions of these catalysts with the substrate leads to more favorable coordination ability with the glucose molecule towards the β-glucopyranose form. This allows to obtain yields of over 70% in halogenated environments.^613–615^ The use of microwaves has allowed to further increase yields above 90%, while also reducing reaction times down to 2 min.^616^ In homogeneous catalysis, these catalysts proved superior in ILs to all other metal chlorides such as Ge, lanthanide and Al chlorides, which barely reach 50% yield at low substrate loading, as these tend to form tetragonal complexes with the anions of the ILs, which have less favorable interactions.^607,617,618^

The involvement of the cation in the dehydration of glucose is less clear than that of the anion. High activity has been often observed for both imidazolium and ammonium ILs, suggesting that neither hydrogen bonding nor polarizability have a clear influence.^619,620^ DFT calculations found that cations with higher acidity can promote the dehydration reaction by acting as a proton bridge.^621^ A strong influence of the alkyl chain of the cation on the yield of the reaction has been also found, with yields decreasing threefold when moving from [C_4_C_1_im]­Cl to [C_8_C_1_im]­Cl.^622^

Solvent modification through the addition of organic solvents or water has shown a favorable effect on the reaction.^623–625^ However, the effect of the co-solvent is strictly related to the type of catalyst used and the IL chosen, therefore a general conclusion on co-solvent effects in ILs cannot be derived.

Many studies have focused on the development of heterogeneous catalysts in [C_4_C_1_im]Cl and [C_4_C_1_im]­Br, with the implementation of Brønsted and Lewis acidic character to efficiently perform multiple reaction steps, representing one of the main topics of heterogeneous catalysis development in ILs. The main challenge in this area is to develop a robust catalyst that is not affected by leaching, as halogenated ILs are good solvents for metal extraction. Different studies have tried to exploit the high efficiency of chromium and stabilize the metal in different supports. Stabilized chromium nanoparticles with ligands and mineral or polymeric supports proved generally inefficient to give a good compromise between leaching and high yield.^626–632^ Hydroxyapatite has been reported as an active catalyst in [C_4_C_1_im]Cl that prevents chromium leaching and provides high recyclability, but microwave heating is required.^633,634^ Functionalized Al_2_O_3_ was used as support for both the dehydration of fructose and glucose with yields over 70% in [C_4_C_1_im]­Cl.^635^ Other studies have focused in the immobilization of tetrahedral tin oxide which exhibits lower leaching compared with chromium showing that the phosphate form and support based on mesoporous silica and metal oxides can avoid leaching while maintaining high performance.^636–638^

##### 2.9.3.3 Cellulose Conversion

The ability of some ILs to dissolve cellulose and the easy accessibility of the β-1,4-glycosidic linkages in solution has led to many studies attempting to obtain HMF directly from cellulose in one pot.^639–641^ However, the development of one catalyst capable of performing the three required steps has proven to be quite complex. Therefore, multi pot syntheses have predominated thus far.^642^

Mechanistic studies have shown that under acidic conditions in [C_4_C_1_im]Cl the hydrolysis of cellulose to glucose takes place in short reaction times, and HMF is observed only after a longer time and in low yield.^643^ Since Lewis acids used for glucose dehydration are not efficient for cellulose hydrolysis, which often requires large amount of catalyst or excessive dilution of the substrate, a good trade-off between Brønsted acidity to hydrolyze cellulose and Lewis acidity to convert glucose into HMF is needed.^644–646^ Moreover, the reaction requires water, which act as an antisolvent for cellulose.^644,647^ Studies of the effect of water content in [C_4_C_1_im]­Cl, have shown that the selectivity towards HMF needs to be optimized at low water contents, while high water content increases the selectivity towards sugars in acid hydrolysis.^644^ A gradual increase of the water composition up to 35% leads to the highest sugar yields of up to 83%. Sugar was then converted into HMF by the addition of CrCl_3_.^648^ In another optimization study, high yields of sugars were obtained by using HCl as a catalyst. Subsequent addtion of CrCl_2_ resulted in an 89% yield of HMF.^649^ However, the main disadvantage of these systems is the incompatibility of the two catalysts for the hydrolysis step, which necessitates a complex separation in the downstream process. Other systems combining both Brønsted and Lewis acids achieve the transformation in one pot through functionalization of the ILs cation. For the transformation with MnCl_2_, acid functionalized IL cations such as 1-(4-sulfonic acid)­butyl-3-methylimidazolium performed better compared with inorganic acids or [HSO_4_]^−^ ILs.^650^ A metallic bifunctional IL synthesized by substituting the hydrogen atom in the 1-(3-sulfonic acid) propane-3-methylimidazole hydrosulfate with Cr or Cu was reported as catalyst in [C_4_C_1_im]­Cl and [C_2_C_1_im]­[C_1_CO_2_] providing yields over 50%, distinguishing its efficiency from other catalytic systems.^651–653^ The high efficiency of sulfonating groups in performing these reactions has been studied through DFT simulations. Results of these simulations showed that the sulfonated group favors the formation of an 8-membered ring, which can reduce the energy of the transition state, favoring both the steps of isomerization of glucose to fructose and dehydration to HMF.^621^ Further functionalization of this cation by introducing aryl units at the C2 position of the imidazolium ring also improved the selectivity of the hydrolysis towards glucose.^654^

Other studies have reported combinations of metal chlorides to achieve the one pot conversion of cellulose into HMF. The addition of salts of Ru, Pd, and Al together with CrCl_3_ proved to enhance the yield of reaction towards HMF.^655–657^ Combinations of Cu with Cr and Fe with Cu lead to a mixture of furanic compounds such as furylhydroxymethyl ketone (FHMK) and furfural alongside HMF, which could increase the total furan yield above 70%.^658–660^ The same approach was used in [C_4_C_1_im]Cl through the use of a high surface area zeolite in combination with LiCl obtaining similar yield at short reaction time.^661^

In another study, heteropolyacids based on tungsten (H_3_PWO_4_) revealed a unique aggregation behaviour in [C_4_C_1_im]­Cl, forming micelles of 10 nm which encapsulate the cellulose molecules, improving the kinetics of the reaction with high yields (75%).^662^

##### 2.9.3.4 Downstream Processing of HMF

The difficulty in separating HMF from ILs has been the subject of several studies. The largest reason is usually seen in the strong interaction between the IL anions (Cl− and Br−) and the hydroxyl group on HMF, increasing the affinity of HMF for ILs and making liquid–liquid extraction difficult due to poor partition coefficient for those ILs that give high HMF selectivity.^581,614,615,619,663,664^

The isolation of HMF from ILs is considered one of the major challenges for the scale-up of production of this compound. Various approaches have been proposed, including separation approaches and “*in situ*” conversion into other compounds which are easier to separate (Table 4).

**Table 4:** Different Strategies for HMF Isolation from ILs

IL anion	separation method	issues	advantages	ref
Cl^‑^	crystallization	extensive use of organic solvents, high selectivity of reaction required	easy operation in small scale	^600^
Cl^‑^	distillation	sever process conditions, low HMF purity	allow to increase the overall yield of reaction	^665^
Cl^‑^	recirculation with acetone and CO_2_	usage of organic solvent and high-pressure operations (undermines ILs benefits)	increase overall yield, simplified HMF isolation	^669^
Cl^‑^	*in situ* conversion into FDCA	low catalyst recyclability	allow to obtain directly a high value product with high purity	^680^
[CF_3_SO_3_]^‑^, [(CF_3_SO_2_)_2_N]^‑^	*in situ* conversion into DFF	low catalyst recyclability	allow to achieve efficient product separation with high purity of the final product	^681^
[HSO_4_]^‑^	stripping with water	very low substrate loading, low yield of reaction	allow to obtain HMF in water phase directly from glucose to be directly converted into FDCA	^612^

Improving reaction selectivity is one of the main routes to improving HMF recovery since side-products can have similar physical behaviour to HMF. A study on the precipitation of HMF from [C_4_C_1_im]Cl through solvent addition (ethanol and ethyl acetate) showed that the purity can vary between 70% and 90% and is correlated to the yield of reaction since by-products can co-crystalize alongside HMF.^600^ This was further observed when the dehydration of fructose was performed at different substrate loadings. A decrease of the HMF purity and lower reaction selectivity were observed when extracted with MIBK if high loadings of fructose were used.^599^

Distillation of HMF can be carried out at low pressure and high temperature, exploiting the high boiling point of ILs. It has been shown that HMF can be boiled out of the reaction mixture at 300 Pa, using temperatures over 180 °C, and through gas or organic compound stripping. At these conditions, the separation occurs at the same rate of the dehydration of fructose into HMF, allowing yields and purities of 90%.^665^ This concept was also used to improve the Brønsted acid catalyzed dehydration of glucose in [C_1_im]­Cl, where HMF was stripped with vapor to improve the yield.^612^ This system has the main advantage that HMF could potentially be converted into FDCA, since its conversion in water is highly favored. Swapping the Cl^−^ and Br^−^ anions with non-coordinating options such as [CF_3_SO_3_]^−^ and [(CF_3_SO_2_)_2_N]^−^ can partially improve the partition coefficient of the ILs, but the choice of the organic solvent is much more limited due to higher miscibility.^604^ The transformation of sugars in hydrophobic [(CF_3_SO_2_)_2_N]^−^ ILs can allow the extraction with water, which is a good candidate solvent to produce FDCA in a second step.^666^ However, hydrophobic ILs do not perform well for this type of reaction and they leach into in the water phase, which is highly undesirable due to their high cost and potential toxicity.^667^

CO_2_ extraction was studied to simplify the HMF isolation and avoid the usage of organic solvents. A minimum of 20 bar pressure was needed to achieve 70% separation, which is still a condition too severe for scale-up.^668^ Other studies used CO_2_ as a phase separator to improve liquid–liquid extraction for the 1-methyl-3-octylimidazolim chloride [C_8_C_1_im]­Cl/acetone system, allowing recirculation of the organic solvent and maximizing the recovery during the reaction. Although this improved the yield, the final product was contaminated due to the partial solubility of sugars in the CO_2_–acetone mixture and product purity did not exceed 90%.^669^

Another approach is the direct conversion of HMF in the IL into molecules that are easier to separate. Some HMF derivatives such as 2,5-diformylfuran (DFF) and 2,5-furandicarboxylic acid (FDCA) have been found to be easier to separate, which can lead to much more favorable process economics. Performing a multiple pot reaction can be beneficial since ILs can solubilize the acid form of FDCA, allowing the reaction to be performed at higher loadings compared to water, and reducing catalyst deactivation by suppressing the formation of humins, which poison the catalyst.^582,670^ FDCA can be then separated through water addition, exploiting the low solubility of this compound in water.^582^ Detailed process economics showed that the development of a catalyst capable of performing the oxidation of HMF to FDCA in ILs would lead to remarkable advantages over other solvents in terms of CO_2_ emissions and operating costs of the process.^537^ However, the development of an efficient catalyst in neoteric solvents remains a barrier to further development. Indeed, noble metal catalysts, which proved to be efficient in the oxidation of HMF into DFF and FDCA in organic solvents and water systems, changed the selectivity towards etherification of the hydroxy groups in Cl^−^ and Br^–^ based ILs, making them unsuitable for catalyst development in this environment.^671^ Ru­(OH)_2_ proved to be active catalyst in [C_2_C_1_im]­[C_1_CO_2_] at severe conditions (20 bar O_2_, 140 °C) but the reaction was slow (24 h) with low yield (<40%). This study demonstrated that while Ru proved to be an outstanding catalyst to oxidize HMF to both DFF and FDCA at high yields in organic solvents or water, this was not the case when ILs were used, indicating a strong influence of the ions on the mechanism of the reaction.^672,673^ Moreover, it is well known that dialkylimidazolium acetate ILs form carbenes above 60 °C, which in this case could be the main species responsible for the oxidation.^618^ It has already been demonstrated in many reports that carbenes are very good catalysts for aldehyde oxidation. Therefore, the role of Ru in the system may be marginal. Leaching of the catalyst, separation of FDCA, low yield, harsh conditions, low HMF loadings, and poor solvent stability are the main issues in this catalytic system.^674,675^ Fe-based catalysts showed activity in [C_4_C_1_im]Cl with a yield of about 60% and up to 6-fold recyclability with no loss in activity. However, these catalysts were tested with a low HMF loading (<1%), so the separation of FDCA was not possible.^676,677^ Another study reported a vanadium/molybdenum-based heteropoly acids catalyst that could achieve a yield of up to 90% in [C_4_C_1_im]­Cl, but with a low substrate loading and extensive leaching of vanadium into the IL.^678^ The development of a manganese-based catalysts has been reported to perform the transformation in [C_4_C_1_im]Cl with the peculiarity of forming the imidazolium salt of FDCA, which is more easily precipitated at low concentration by ethanol addition. This system allowed the implementation of a two-step process where first the sugars are converted into HMF and then converted into FDCA salt through addition of MnO_2_, representing one of the main examples where the advantages of ILs in product separation and sugars dehydration are fully exploited. Although high isolated yields from glucose were achieved (over 85%) with efficient separation at low loading (<2%), the process suffers from catalyst leaching and deactivation, further compromised by water removal between the two steps, which necessitates an expensive, high-energy step to dry the IL.^679,680^

Another study reported the conversion of HMF to DFF. This selective conversion can break the strong interaction between the hydroxyl group and the anions, ensuring easier separation. One of the unusual aspects of this system is the separation of DFF by sublimation, which led to isolation of a product with high purity.^582^ The use of the CuCl/TEMPO system in [C_4_C_1_im]­[CF_3_SO_3_] made it possible to ensure isolated yields of over 80% at a substrate loading of over 30% of HMF.^681^ A two-step process where the conversion of fructose was optimized in [C_4_C_1_im]­[CF_3_SO_3_] with HCl and then converted into DFF by the addition of CuCl/Tempo was also proposed.^582,600,681^ However, even in this case, the metal catalyst proved to be poorly recyclable, which was compounded by the difficulty of removing the deactivated form of copper from the reaction mixture.

### 2.10 Products from Cellulose

#### 2.10.1 Cellulose Solubilization

Cellulose has been employed in material formulations for centuries, thanks to its availability, mechanical properties and environmental friendliness. However, processing, derivatizing and converting cellulose presents a significant hurdle due to its highly ordered structure and robust hydrogen bond network, rendering it insoluble in traditional solvents. Cellulose dissolution is a challenging task and researchers have explored various approaches to find suitable solvents.^39,70^ The industrial production of regenerated cellulose primarily relies on the conventional viscose method, which involves hazardous substances such as strong bases, CS_2_ and sulfuric acid and contributes to severe pollution through the release of H_2_S and SO_2_.^682^ To address this issue, new solvent systems have been developed for dissolving and processing cellulose. A select few molecular solvents, including *N*-methylmorpholine oxide (NMMO), *N*,*N*-dimethylacetamide/lithium chloride (DMA/LiCl) and 1,3-dimethyl-2-imidazolidinone/lithium chloride (DMI/LiCl), among others, can dissolve cellulose. Nevertheless, these conventional methods possess considerable disadvantages, including complex operations, significant pollution, high consumption of reagents and energy and inefficient solvent recycling or recovery due to evaporation, restricting their industrial-scale application.^682,683^ Prompted by these drawbacks, new solvents, such as ILs or DES, have been thoroughly investigated. In 2002, a breakthrough was achieved when certain imidazolium-based ILs were identified as effective solvents for cellulose by Rogers and co-workers.^17^ Notably, [C_4_C_1_im]Cl emerged as one of the most efficient ILs for this purpose. Due to its potential use in sustainable processes and the creation of innovative materials, cellulose dissolution with ILs has attracted a lot of recent attention since then. ILs provide several benefits, including the ability to dissolve cellulose without the need for harsh chemical steps such as derivatization and the possibility of being reused and recycled.^684^ As a result, the process becomes more effective and sustainable, consuming less energy and producing less residue. Additionally, ILs can be tailored by choosing particular cations and anions to enhance their efficiency in solubilizing cellulose.

However, the solubilization mechanism of cellulose in ILs was not fully understood at that time. Researchers conducted in-depth studies to explore the interactions between ILs and cellulose based on NMR studies on IL solutions of glucose and cellobiose and molecular dynamics studies of the interaction of [C_2_C_1_im]­[C_1_CO_2_] or [C_4_C_1_im]Cl with cellulose oligomers.^685,686^ These results agree with empirical measurements of solvent polarity, which are used to forecast solvent parameters including rate constants and solubility, among others. The solubility of biomolecules and biopolymers in solvents has been explained and predicted using different empirical and semi-empirical polarity scales, such as the COSMO-RS and K-T polarity parameters developed by Hansen.

The ability of an IL to dissolve cellulose depends on the hydrogen-bond basicity of its anion, given by the K-T β parameter (Figure 47), and the structure of its cation. ILs with anions that possess strong hydrogen-bonding capability, such as chloride, carboxylates (*e.g.*, formate, acetate, lactate), AA derivatives, phosphate or phosphonate, are effective solvents for cellulose. On the other hand, cations such as tetralkyl-ammonium, dialkyl imidazolium, morpholinium and alkylpyridinium have been showing good cellulose dissolving ability. In contrast, ILs with anions like hexafluorophosphate or tetrafluoroborate are ineffective in dissolving cellulose.

**Figure 47:**
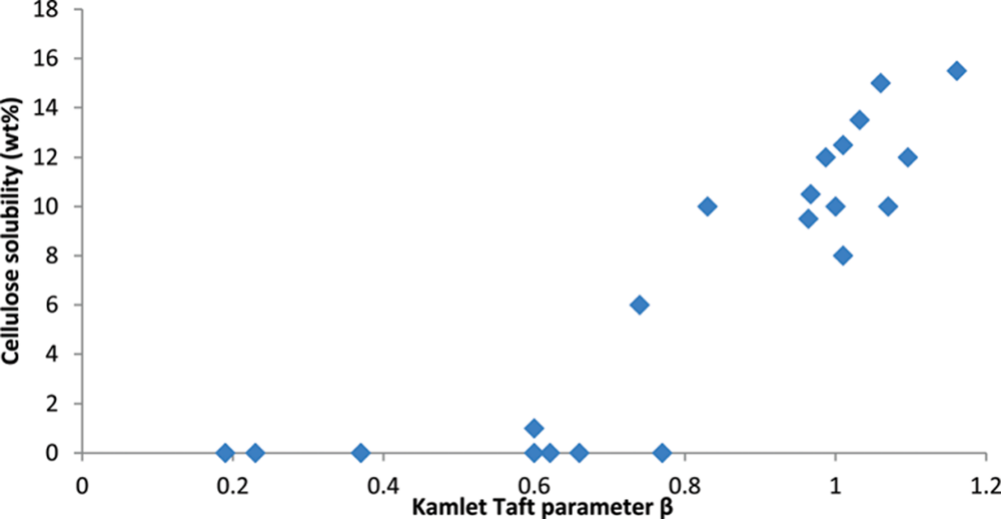
Relationship between the K-T β parameter of several ILs and their cellulose dissolving ability.

Several factors influence the solubility of cellulose in ILs. For instance, the degree of polymerization of cellulose, the temperature of the dissolution process and the presence of impurities. The addition of polar aprotic co-solvents such as DMSO or DMF, to ILs can further enhance cellulose solubility once it lowers the viscosity of the solution, thereby improving the system’s mass transport properties. Moreover, the inclusion of certain additives, such as solid acids (*e.g*., Amberlyst 15) or metal chlorides that help breaking hydrogen bonding (*e.g.*, ZnCl_2_, LiCl, or NaCl), can enhance cellulose dissolution, allowing higher cellulose loading, shorter dissolution times or lower temperatures.^70^

Hydroxyl groups on the alkyl chains of the IL, or functionalities that increase the hydrogen-bond acidity of the IL, also reduce the solubility of cellulose. The same effect is seen when a hydroxyl group is situated on the anion or by the presence of water molecules. A protic cation such as monoethanolammonium prevents cellulose solubilization entirely in many cases. This could be due to stronger interactions between cations and anions, making the IL less able to dissolve cellulose.

Apart from traditional ILs, researchers have investigated more exotic alternatives, such as ILs derived from polycyclic amidine bases or organic superbases such as 1,5-diazabicyclo-[4.3.0]­non-5-ene (DBN) and 1,8-diazabicyclo[5.4.0]­undec-7-ene (DBU).^70^ When paired with carboxylic acids, such as acetate or propionate, cellulose solutions with relatively low viscosities could be obtained. These ILs have shown promising results in dissolving cellulose and are notable for their low cost and easy recyclability through distillation.^687^

Nevertheless, the choice of specific anions and cations in ILs is crucial to avoid undesired side reactions during cellulose dissolution. Some ILs may lead to depolymerization or other chemical transformations of cellulose, limiting their practical application. While ILs offer great promise as solvents for cellulose due to their good solubilities, enhanced stability of cellulose in solution, and low toxicity (*e.g.*, [C_2_C_1_im]­[C_1_CO_2_]), there is still ongoing debate and research on the dissolution mechanism of cellulose in ILs. Interested readers are encouraged to explore the comprehensive reviews by Brandt *et al*.,^39^ Szabo *et al*.,^70^ Bodachivskyi *et al*.,^688^ and Verma *et al*.^328^ for a deeper understanding.

#### 2.10.2 The Sugar Platform for Bioconversion of Carbohydrates into Platform Chemicals

The “two-platform concept” is one that considers both the production of synthesis gas from biomass (syngas platform) and the synthesis of designated functional platform chemicals (sugar platform) (Figure 48).^689^ Under the thermochemical route, syngas can be catalytically converted into methanol, which may then be transformed into a number of derivatives. Fischer–Tropsch technology makes it possible to obtain hydrocarbons utilized as fuels. However, Fischer–Tropsch liquid synthesis from biomass is not currently economically feasible, posing a difficult problem for research and development.^690^ Furthermore, the thermochemical method of biomass gasification is beyond the scope of this review and will not be addressed here.

**Figure 48:**
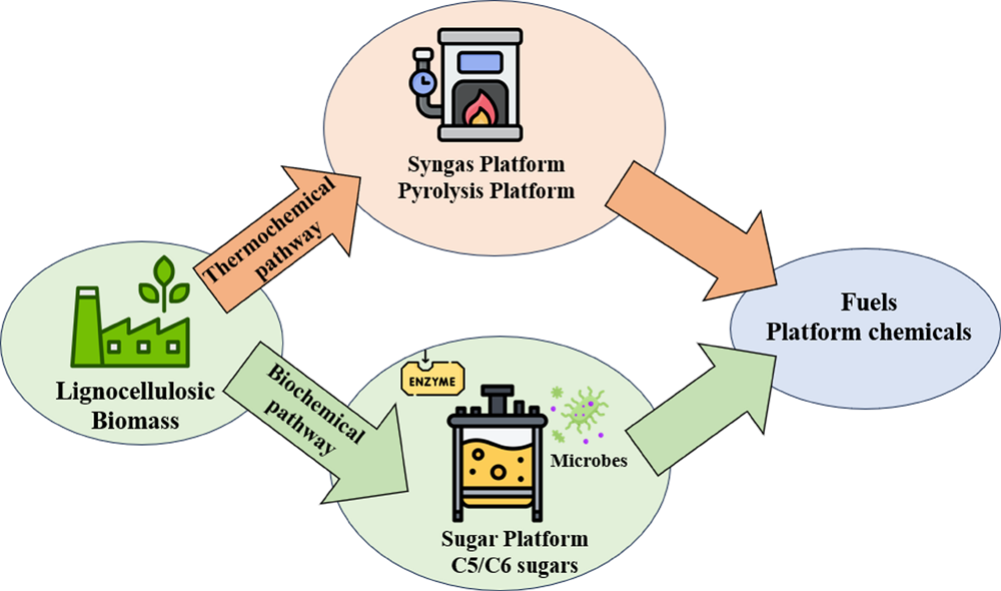
The two-platform concept of utilization of lignocellulosic biomass (adapted from Santos *et al.* ^691^).

The sugar platform is one of the most important chemical platforms and is currently thought to be the largest platform for the synthesis of chemicals from biomass based on volume.^692^ Many well-established businesses and conventional biorefinery technologies are based on sugars. The sugar platform in a lignocellulosic biorefinery frequently combines glucose (predominantly from cellulose) with various sugars generated from hemicellulose. Alcohols, organic acids, lipids, and hydrocarbons are among the important chemical building blocks that can be accessed through many biological fermentation processes.^693^ However, glucose also provides access to a number of very valuable fine chemicals and products, such as AAs, vitamins, antibiotics and enzymes.^692^ In a lignocellulosic biorefinery, the mixed sugar platform has the potential to create compounds similar to glucose. However, before these intriguing prospects can be exploited, a variety of technological, biological and economic barriers must be overcome.

The sugar platform route has cellulosic or second-generation 2G ethanol as one of the main bioproducts. In terms of processing, there are several ways of producing 2G ethanol (Figure 49). After the pretreatment step, enzymatic hydrolysis and fermentation are undertaken, they can be done by separate hydrolysis and fermentation (SHF), simultaneous saccharification and fermentation (SSF), simultaneous saccharification and co-fermentation (SScF), hybrid saccharification and fermentation (HSF) or CBP.

**Figure 49:**
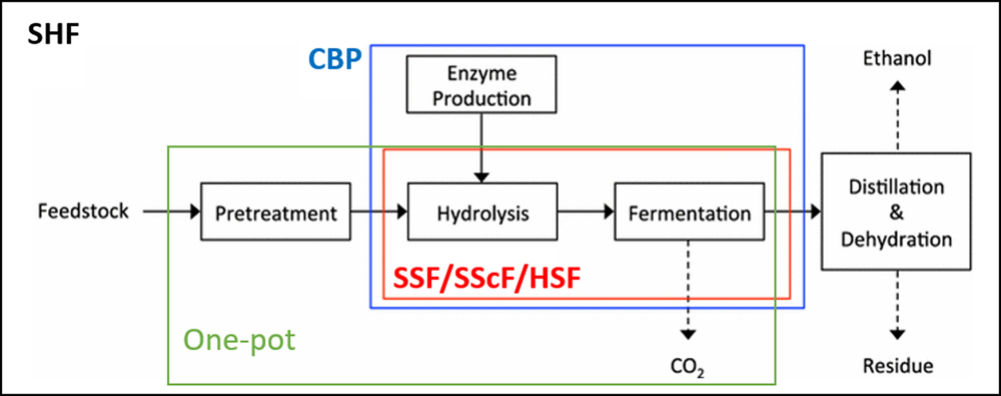
Cellulosic ethanol production process. SHF, separate hydrolysis and fermentation; SSF, simultaneous saccharification and fermentation; SScF, simultaneous saccharification and co-fermentation; HSF, hybrid saccharification and fermentation; CBP, consolidated bioprocessing. Adapted from ref 694. Copyright 2016 Springer Nature.

In SHF, hydrolysis and fermentation can each be performed at their optimal conditions in terms of temperature, and the yeast either can be recycled or possibly used as revenue as a protein source. However, two drawbacks are the end-product inhibition and sugar losses during lignin separation before fermentation, that ultimately decrease ethanol yields.^694^ To overcome this, hydrolysis and fermentation operations can be combined in a single reactor in the SSF process mode. Nevertheless, this does not necessarily decrease CAPEX, as the residence times may not be similar. Since the temperature in SSF is not optimal for cellulases, the rate of hydrolysis is slow, but hydrolysis products can be consumed as they are produced during fermentation, avoiding the inhibition found in SHF.^695^ Ethanol yields are usually higher in SSF compared to SHF, but a higher enzyme loading is required and yeast cannot be reutilized.^694^ When engineered yeasts or wild type yeasts that can ferment C5 and C6 sugars are used in SSF, the process is termed as SScF. HSF was created to leverage SHF and SSF. In HSF, samples are incubated with cellulases under ideal conditions before SSF. The one-pot strategy integrates pretreatment and saccharification, followed by fermentation by the direct extraction of sugar and recovery of lignin as a by-product of the process, avoiding the liquid–solids separations and washing steps after the pretreatment, reducing capital costs and eliminating sugar losses during these separations.^315,332,330^ Finally, the CBP concept is similar to the one-pot, but it also employs microorganisms that are engineered to produce the needed hydrolytic enzymes. In the following sections, the production of fermentation products by SHF will be discussed for 2G ethanol and other biobased platform chemicals such as butanol, lipids and succinic acid.

#### 2.10.3 2G Ethanol

The most used liquid biofuel, ethanol, is typically manufactured from feedstocks based on starch and sugar (1G fuel ethanol) for blending with petrol due to its high-octane number. The US and Brazil are the top producers of 1G ethanol from corn and sugarcane, producing nearly 70 billion and 30 billion liters of ethanol in 2022.^696,697^

A number of studies have been dedicated to explore ILs as pretreatment agents for 2G production (Table 5). A wide variety of feedstocks have been employed, ranging from grasses such as rice straw, corn stover and sugarcane bagasse, hardwoods such as oak, *Eucalyptus* and aspen, and softwoods such as spruce. Two studies employed category I ILs (neutral AILs) which is characterized for not significantly alter the biomass structure.^698,699^ It could be seen that the ethanol yield from [C_4_C_1_im]Cl pretreatment of spruce and hornbeam was quite low (below 40%).^699^ The reason why the ethanol yields were high is because the authors employed microcrystalline cellulose, which facilitates the subsequent enzymatic hydrolysis. The majority of the studies employed category II ILs (alkaline ILs), which are delignifying agents, leaving a carbohydrate-rich pulp with hemicelluloses and celluloses. Therefore, after enzymatic hydrolysis, a C5+C6 mixed hydrolysate will be generated, which implies the need of either recombinant *S. cerevisiae* or bacteria such as *E. coli* or wild type yeasts such as *S. passalidarum*. However, some studies did not employ such type of microorganisms, meaning that the C5 sugars were underutilized. There were also two studies that employed category III ILs (Brønsted acidic ILs) that produce a high purity cellulose pulp, which avoids the need to use C5 fermenting microorganisms, but then poses a problem of C5 sugar recovery from the IL liquor.^700,701^

**Table 5:** Summary of the Yields, Conditions, IL Systems, and Feedstock Reported for the Production of 2G Ethanol with ILs

IL	biomass	PT conditions	saccharification and fermentation conditions	yields	ref
[C_2_C_1_im][(C_2_O)_2_PO_2_], [C_2_C_1_im]Cl, [C_2_C_1_im][C_1_CO_2_]	microcrystalline cellulose Avicel	80 °C, 30 min, acetate buffer as antisolvent	recombinant *S. cerevisiae* (with cellulase expression), 96 h, 30 °C	90% EtOH	Nakashima *et al.* 2011 ^698^
[C_2_C_1_im][C_1_CO_2_]	cotton stalks	140 °C, 30 min, 30 wt% loading	10 wt% solids, 48 h, 50 °C; recombinant *S. cerevisiae*, 30 °C, 120 h	74.1% EtOH	Haykir and Bakir, 2013 ^702^
[C_2_C_1_im][C_1_CO_2_]	spruce sawdust	120 °C, 15 h, H_2_O as antisolvent	5 wt% solids, 72 h, 45 °C; *S. cerevisiae*, 32 °C, 24 h	81.5% EtOH	Shafiei *et al.* 2013 ^703^
[C_2_C_1_im][C_1_CO_2_]	aspen wood	120 °C, 5 h, 10 wt% loading, H_2_O as antisolvent	3 wt% solids, 72 h, 30 °C; *S. cerevisiae*, 35 °C, 30 h	81.2% EtOH	Goshadrou *et al.* 2013 ^704^
[C_2_C_1_im][C_1_CO_2_]	corn stover	140 °C, 3 h, 10 wt% loading	10 wt% solids, 72 h, 50 °C; recombinant *S. cerevisiae*, 30 °C, 120 h	93% EtOH	Uppugundla *et al.* 2014 ^705^
[C_2_C_1_im][C_1_CO_2_]	rice straw	120 °C, 5 h, 5 wt% solids, H_2_O as antisolvent	10 wt% solids, 72 h, 45 °C, 20 FPU/g DM, 30 IU/ g DM; *S. cerevisiae*, 38 °C, 48 h	80% EtOH	Poornejad et al. 2014 ^706^
[Ch][C_1_CO_2_]	sugarcane bagasse	ultrasonication at 25 °C for 1 h, H_2_O as antisolvent	3.3 wt% solids, 48 h, 50 °C; *S. cerevisiae*, 30 °C, 24 h	75% EtOH	Ninomiya et al. 2015 ^707^
[C_2_C_1_im][C_1_CO_2_]	*Eucalyptus*	150 °C, 30 min, 1:3 solid liquid-ratio, H_2_O as antisolvent	10 wt% solids, 72 h, 45 °C, 37 FPU/g DM, 4.9 CBU/ g DM, *S. cerevisiae*, 40 °C, 72 h	38% EtOH	Lienqueo *et al.* 2016 ^708^
[C_2_C_1_im][C_1_CO_2_]	agave	120 °C, 3 h, 10 wt% loading	10 wt% solids, 72 h, 50 °C, 8 FPU g/DM, 15 CBU g/DM; *E. coli*, 30 °C, 120 h	81.6% EtOH	Perez-Pimienta *et al.* 2017 ^709^
[C_2_C_1_im][C_1_O(H)PO_2_]	spruce sawdust	110 °C, 40 min, 2 wt% loading, H_2_O as antisolvent	10 wt% solids, 80 h, 37 °C; *K. marxianus*, 35 °C, 72 h	84.3% EtOH	Mehmood *et al.* 2018 ^710^
3-methylmorpholinium chloride [C_1_^3^Morph]Cl	rice straw	120 °C, 5 h, 5 wt% loading, H_2_O as antisolvent	5 wt% solids, 72h, 45 °C; *S. cerevisiae,* 30 °C, 24 h	64% EtOH	Mohammadi *et al.* 2019 ^711^
[C_4_C_1_im][C_1_CO_2_]	sugarcane bagasse	120 °C, 2 h, H_2_O as antisolvent	10 wt% solids, 48 h, 47 °C; *S. cerevisiae*, 36 °C, 96 h	89.3% EtOH	Smuga-Kogut *et al.* 2019 ^712^
aqueous [C_4_C_1_im]Cl + HCl	mixed softwood, hardwood, sugarcane bagasse	130 °C, 3 h 15 wt% solids, 20 wt% H_2_O	10 wt% solids, 72 h, 50 °C, 17.25 FPU/g DM, 6.26 CBU/ g DM, 25 FXU (xylanase); *S. cerevisiae*, 30 °C, 48 h	21.86–29.56 g/L EtOH, up to 99% EtOH	Trinh *et al.* 2019 ^700^
[C_4_C_1_im]Cl	spruce	150 °C, 1 h, 20 wt% loading, H_2_O as antisolvent	5 wt% solids, 72 h, 28, 37, or 50 °C; *S. cerevisiae*, 37 °C, 72 h	32% EtOH	Dotsenko *et al.* 2018 ^699^
[C_4_C_1_im]Cl	hornbeam	150 °C, 1 h, 20 wt% loading, H_2_O as antisolvent	5 wt% solids, 72 h, 28, 37 or 50 °C; *S. cerevisiae*, 37 °C, 72 h	36% EtOH	Dotsenko *et al.* 2018 ^699^
[C_2_C_1_im][C_1_CO_2_]	oak and spruce	45 °C, 40 min, 2 wt% solids, H_2_O as antisolvent	80 h, 40 °C, cellulases; *S. cerevisiae*, 72 h, 30 °C	53–54% EtOH	Alayoubi *et al.* 2020 ^713^
[C_2_C_1_N][C_1_CO_2_]	sugarcane bagasse	150 °C, 2 h, 15 wt% loading, 30 wt% H_2_O	10 wt% solids, 72 h, 50 °C, 15 FPU/g DM; *S. passalidarum*, 28 °C, 72 h	87.4% EtOH	Nakasu *et al.* 2021 ^714^
[(H_2_N)^2^C_2_N][C_1_CO_2_]	sugarcane bagasse	140 °C, 3 h, 15 wt% loading, 30 wt% H_2_O	10 wt% solids, 72 h, 50 °C, 15 FPU/g DM; *S. passalidarum*, 28 °C, 72 h	85.3% EtOH	Pin *et al.* 2021 ^715^
[C_2_C_2_C_2_N][HSO_4_]	wheat straw	130 °C, 3 h, 20 wt% solids, 20 wt% H_2_O, H_2_O as antisolvent	72 h, 50 °C, 28 FPU/g DM; *S. cerevisiae*, 96 h, 30 °C	84% EtOH	Ziaei-Rad *et al.* 2021 ^701^

Cellulosic ethanol production and therefore commercialization has been lagging due to technoeconomic issues especially related to the pretreatment step;^716^ such issues include equipment corrosion due to high abrasiveness of biomass and soil particles, solvent recovery and recycle, and lignin encrustation.^717^ ILs offer superior performance in terms of pretreatment efficacy compared to conventional pretreatments such as hydrothermal, dilute acid or ammonia fiber expansion (AFEX). However, its technology level is still incipient and there is uncertainty regarding several of the aforementioned issues, especially equipment corrosion, since IL pretreatment entails using highly concentrated salts solutions. Recent advances in the use of distillable ILs may represent a significant inflection point in the development of commercial IL-based biorefineries due to efficient solvent recovery and recycling.^718,719,249^

IL pretreatment for 2G ethanol production also faces competition with already well-known pretreatment methods such as steam explosion, dilute acid, and AFEX.^720,721^ Such methods can partially remove hemicelluloses and lignin and produce a carbohydrate-rich material suitable for further biochemical conversion. Companies like Abengoa and DuPont have employed the aforementioned pretreatment methods with nominal capacities of 25 and 30 million gallons a year, respectively.^722^ More recently, Shell has entered into a significant agreement with Brazil’s Raízen to purchase 3.25 billion litres of sugarcane cellulosic ethanol.^723^ This 2G ethanol will be produced in five new plants that Raízen plans to construct in Brazil, thereby expanding its portfolio of cellulosic ethanol facilities to nine. The partnership leverages Shell's contribution of the cellulosic ethanol technology to Raízen, a joint venture formed with Cosan SA in 2011, which has since then advanced the method of producing ethanol from sugarcane waste. The initiative is set to bolster the global supply of sustainable fuels, noting the increasing demand for such fuels and the advantage of combining Raízen’s innovative technology with Shell's extensive distribution network. Raízen’s second-generation ethanol (E2G) technology, although not disclosed, it is more likely a non-IL pretreatment (such as dilute acid) that optimizes the use of sugarcane waste, allowing for a 50% increase in ethanol production from the same land area without competing with food crop cultivation. With an investment of around $1.5 billion, the new facilities are projected to start production by 2025 and fully operational by the end of 2027 at the latest, marking a significant milestone towards large-scale production of sustainable, waste-based, low-carbon fuels that contribute to global decarbonization efforts.^723^ Given the magnitude of Raízen’s E2G process, IL technology still seems to be still at its dawn, and therefore, its commercial implementation needs to be assessed by pilot and demonstration plants, which, until this moment, has only been probed by Barcelos *et al*. (2021) at a 680 L scale in a one-pot pretreatment of California woody biomass with choline lysinate, [Ch]­[Lys].^183^

#### 2.10.4 Other Fermentation Bioproducts

The sugar platform is not limited to 2G ethanol only. Other fermentation bioproducts can be obtained by microorganisms such *Clostridium* (butanol), *Rhodosporidium*, *Rhodococcus*, *Trichosporon* (lipids) and *Actinobacillus* (succinic acid) (Table 6). It can be noticed that most of the studies employ either category I or II ILs, which produce mainly carbohydrate-rich pulps that can be hydrolyzed into a C5/C6 mixture of sugars. Most also only employed grassy feedstocks which are easier to pretreat compared to wood.

**Table 6:** Different Products Obtained from IL-Based Sugar Platform Processes and Process Conditions

IL	bioproduct	biomass	pretreatment conditions	microorganism	yields (g/g total-sugar^–1^)	ref
[C_4_C_1_im]Cl	butanol	corn stover	130 °C, 0.5 h, 5 wt% loading, H_2_O as anti-solvent	*Clostridium saccharobutylicum* *DSM 13864*	0.21	^724^
[C_2_C_1_im][C_1_CO_2_]	butanol	rice straw	150 °C, 0.5 h, 10 wt% loading, H_2_O as antisolvent	*Clostridium beijerinckii* *TISTR 1461*	0.47	^725^
[C_2_C_1_im][C_1_CO_2_]	butanol	Napier grass (*Pennisetum purpureum*)	150 °C, 3 h, 3% (w/v) loading, 1:1 IL:H_2_O ratio, H_2_O as antisolvent	*Clostridium beijerinckii* *JCM 8026*	0.23	^726^
[C_2_C_1_N][C_1_CO_2_]	butanol	rice straw	150 °C, 2 h, 15 wt% loading, 4:1 IL: H_2_O ratio, H_2_O as antisolvent	*Clostridium beijerinckii* *DSM 6422*	0.08	^727^
[C_2_C_1_im][C_1_CO_2_]	lipids	corn stover	140 °C, 2 h, 9 wt% loading, 4:1 IL: DMSO ratio, methanol as antisolvent	*R. toruloides Y4*	51%	^728^
[C_2_C_1_im][C_1_CO_2_]	lipids	corn stover	140 °C, 1 h, 9 wt% loading, 4:1 IL: NMP ratio, 40 wt% K_3_PO_4_ as antisolvent	*R. toruloides Y4*	36.4%	^729^
[C_1_C_1_im][(C_1_O)_2_PO_2_]	lipids	corn cob	130 °C, 20 min, 3 wt% loading, H_2_O as antisolvent	*Rhodococcus opacus* strain *ACCC41043*	41–43%	^730^
[C_4_C_1_im][C_1_CO_2_]	lipids	rice straw	135 °C, 1 h, 50 wt% loading, H_2_O as antisolvent	*Trichosporon fermentans*	28.1%	^731^
[(C_1_=C_2_)C_1_im]Cl	succinic acid	pinewood	90 °C, 1 h, 7 wt% loading, 17 wt%: DMSO, H_2_O as antisolvent	*Actinobacillus succinogenes*	0.37	^732^
[Ch][Gly]	succinic acid	mulberry stem	90 °C, 6 h, 10 wt% loading, H_2_O as antisolvent	*Actinobacillus succinogenes* *ATCC55618*	0.89	^733^

Butanol, a main ABE (acetone, butanol, and ethanol) fermentation product, is used in a variety of sectors as a solvent for hormones, medicines, and cosmetics production. It is also gaining popularity as a possible direct replacement for gasoline or as a fuel additive. Butanol is deemed a superior fuel to ethanol due to its higher heating value, lower volatility and lower corrosiveness. Historically, issues related with process cost and development forced the closure of commercial ABE fermentation plants.^734^ Concerns over over environmental impact and the drive toward carbon neutrality, have reignited interest in producing butanol from sustainable and economical feedstocks. In China, the ABE fermentation process, a key biofuel manufacturing technology, has been widely used.^724^

However, producing butanol from microorganisms has some drawbacks that must be carefully considered. One key problem is achieving high butanol yields and productivity rates, as microorganisms frequently have complex metabolic pathways that might produce undesired by-products.^735^ Furthermore, ABE fermentation requires precise control of environmental parameters such as temperature, pH and nutrient availability, which can be resource-intensive and potentially impair the economic feasibility of large-scale production.^735^ As most ABE-fermenting microorganisms are sensitive to the presence of chemicals in the medium, ILs can pose an extra metabolic burden and negatively affect butanol production.^725,726^

The use of oleaginous microorganisms such as microalgae, bacteria, yeast, and fungi has led to the development of 3G biofuels.^736^ Through biorefinery processes, this lipid-rich biomass can be turned into useful products such as biodiesel, bio-oil, bio-ethanol and bio-hydrogen.^737^ Many oleaginous bacteria, however, cannot metabolize xylose. The increasing interest in xylose utilization has prompted extensive research on the yeast *R. toruloides*, including proteomics and genome-scale metabolic models, to better understand the regulation of lipid and carotenoid formation from the pentose.^738^ Once *R. toruloides* offers potential for mixed C5/C6 utilization, it is important to understand the impact of IL on the lipids production. Huang *et al.* (2013) evaluated the impact of imidazolium-based ILs on lipid production by *R. toruloides* AS 2.1389.^739^ They found out that maintaining low IL concentration was crucial to maintain high lipid production. As they increased the IL concentration from 30 to 60 mM inhibition was largely dependent on ILs, with [C_2_C_1_im]­[C_1_CO_2_] being the worst. They report that the acetate anion of [C_2_C_1_im]­[C_1_CO_2_] was being assimilated, leading to a rapid alkaline-pH shift and enhanced inhibition on cell growth and lipid production.

Succinic acid occupies a pivotal position as a moderately high-value chemical, underpinning the production of more than 30 commercially significant products like 1,4-butanediol (BDO), THF, adipic acid and γ-butyrolactone (GBL).^740^ Its chemical versatility finds applications across a multitude of industries, ranging from food and pharmaceuticals to polymers, paints, cosmetics and inks. It even serves as a surfactant, detergent extender, antifoam and ion-chelator. Given the escalating global demand for succinic acid, the imperative to achieve cost-effective production through biomass-based means becomes increasingly evident. This cost efficiency is crucial for the compound to competitively displace chemicals currently derived from petroleum feedstocks. In a pioneering study on succinic acid production via [(C_1_=C_2_)­C_1_im]Cl pretreatment of pinewood and corn stover, Wang *et al*. (2014) showed that from 5% (v/v) [(C_1_=C_2_)­C_1_im]Cl started to inhibit bacterial growth, and from 0.01% (v/v) it inhibited succinic acid production showing that this IL is toxic toward the microorganism.

#### 2.10.5 Cellulosic Materials

One of the uses of the cellulose pulp or cellulose-rich material (CRM) derived from lignocellulosic biorefineries lies in the fabrication of materials. ILs and DESs have been found to dissolve up to 30 wt% of cellulose, and using them, a range of cellulose-based materials could be produced (Figure 50). ILs containing dissolved cellulose can be further processed to create useful products with desired qualities depending on the type of processing, including films, fibres, and gels.^70^ Techniques such as wet and electrospinning, casting, coagulation and 3D printing have been used to produce these novel materials. Besides the established production of cellulosic products, interesting technical applications are developed such as super microfilament fibers, cellulose/chitin blend fibers, precursors for carbon fibers and all-cellulose composites.^684^ The distinct mechanical and rheological features of the cellulose-IL solutions enable the creation of materials with adaptable qualities for use in biotechnology, nanotechnology and green chemistry.^314^ The method of formulating cellulose and regenerated cellulose materials through IL dissolution is well-established and has been thoroughly researched. Numerous studies have utilized a variety of ILs to dissolve cellulose from diverse sources including cellulose kraft pulp, microcrystalline cellulose (MCC), CRM, cellulose derivatives, etc., thereby creating new materials with a wide range of properties and application profiles. The most commonly used ILs for cellulose dissolution are those based on imidazolium, pyridinium, morpholinium and pyrrolidinium cations, with the most frequent anions being Cl^−^, [C_1_CO_2_]^−^, [HCO_2_]^−^, and phosphate anions such as [(C_2_O)_2_PO_2_]^−^ or [(C_1_O)_2_PO_2_]^−^, among others and the super base ILs, *e.g.*, [DBNH]­[C_1_CO_2_].^683,741^

**Figure 50:**
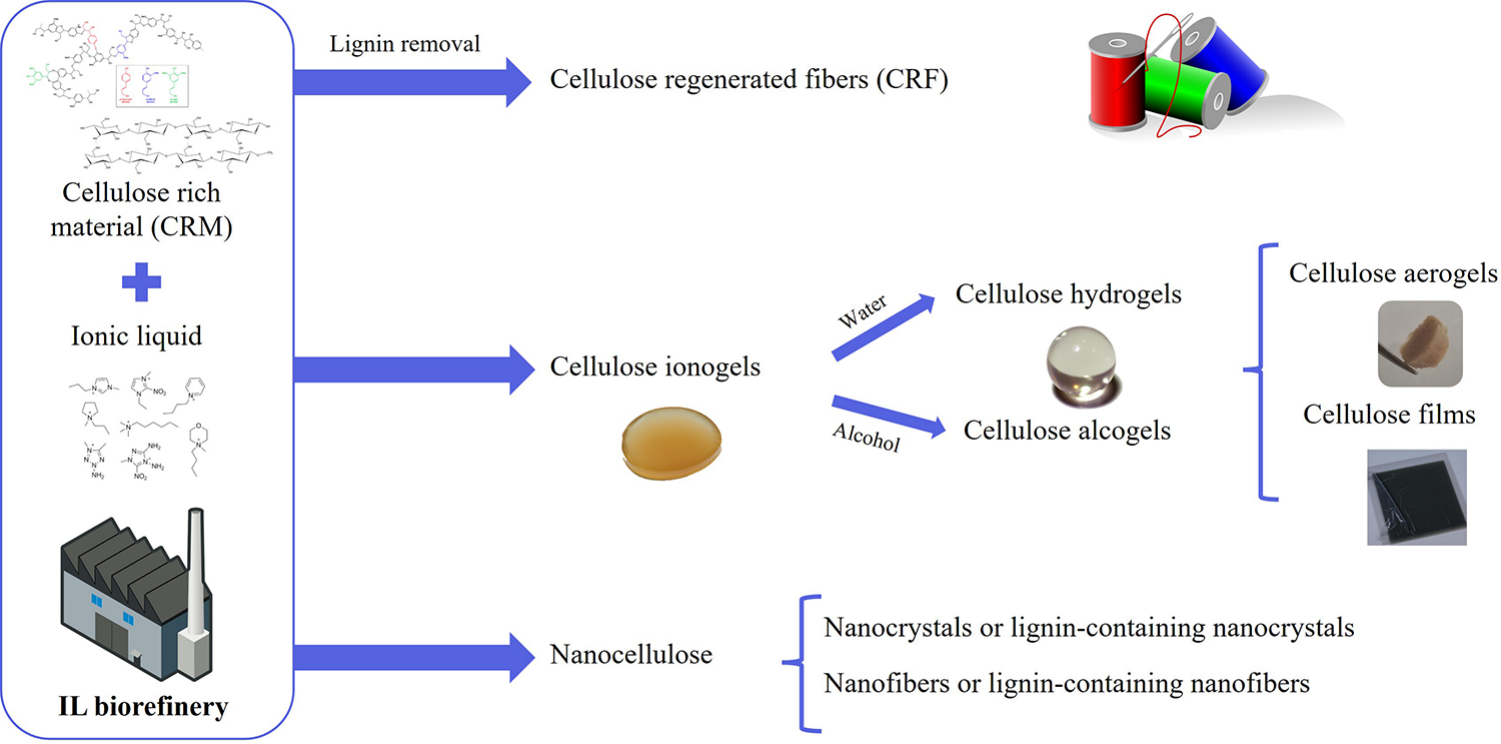
Cellulose materials that can be formulated using CRM.

However, the industrial application of these materials remains challenging. This is not solely due to the costs or toxicity of the ILs, as is generally the case with biorefinery processes, but also due to issues of scalability and the lack of research on production beyond milligrams (mg) of these materials to study process feasibility and overall costs. Only the production of regenerated fibers (IONCELL) and films (NILCELL) are being produced on pilot scale, and, in those cases, a cellulose kraft pulp is used. Furthermore, the utilization of CRM or cellulose pulp from biorefinery streams, which involves the use of cellulose with varying lignin amounts, has not been as extensively studied as the use of cellulose kraft pulp, MCC, or cellulose derivatives. Therefore, assessing the viability of these processes for industrial application becomes a difficult endeavor, but the development of high-value specialty products can catalyze technological advancements before they can be applied to the production of large-scale platform chemicals. This section offers an overview of the processes employed in the formulation of various materials using cellulose and ILs. It is followed by an examination of potential industrial challenges that may emerge, along with proposed solutions to address them.

##### 2.10.5.1 Cellulose Fibers

For countless millennia, the utilization of fiber was constrained exclusively to natural fibers, depending on their specific applications to fulfill essential requirements such as clothing, storage, building materials and everyday items like ropes and fishing nets. Natural fibers such as cotton or silk, are generally environmentally friendly, but the consumption of huge amounts of water and the requirements of high-grade arable lands that compete with the cultivation of edible goods increase the necessity of more ecological options. Moreover, the rising demand, coupled with limited reserves and fluctuations in production, has led to an upward trajectory in their prices.^742,743^ The alternative for those natural fibers are the fibers manufactured by humans, termed man-made fibers. Those can be synthetic or cellulosic fibers.^744^ The production of synthetic fibers is easy, cheaper, and more versatile than natural fibers, but many are non-biodegradable, they need fossil fuels for their production, and due to their intrinsic characteristics, such as breathability or wearing comfort, they fail to adequately replicate natural fibers.^745^ Man-made cellulose fibers or regenerated cellulose-based fibers (RCFs) have gained attention as a means of addressing the limitations of synthetic fibers and incorporating the advantageous properties of natural fibers.^743,746^ Currently, two of the most important processes to produce cellulose fibers are the viscose process (which implies cellulose derivatization) and the Lyocell process (without cellulose derivatization, Figure 51).^363^

**Figure 51:**
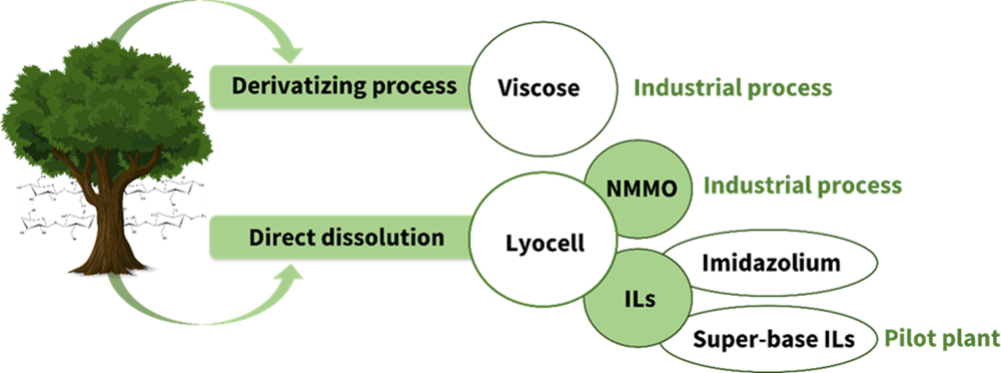
Methods to produce cellulose fibers.

The viscose process has been used for several decades and is well-established in the textile industry, producing fibers with good drape, softness, and dye affinity. The regenerated fibers are used in home textiles, clothing, and industrial textiles. However, the process involves the use of harsh chemicals and has environmental concerns, such as the generation of harmful effluents, as mentioned above. Under these circumstances, the Lyocell process appears as an eco-friendly alternative. It consists in the direct dissolution of cellulose in *N*-methylmorpholine *N*-oxide (NMMO). The fibers are made by dry-jet wet spinning, where the liquid fibers (dope) pass through the spinneret and air gap and then are immersed in a aqueous coagulation bath where the RFCs are obtained.^741,363^ Lyocell fibers stands out from cotton and viscose fibers due to its exceptional mechanical properties, positioning it as an ideal choice that bridges the gap between natural and synthetic fibers. As a result, lyocell is projected to emerge as the future generation of sustainable cellulose fibers for industrial use. There are several brands that produce RCFs based on this process: Birla Cellulose or Excel, Tencel, or Cell Solution CLIMA (that use the ALCERU process), among others. For more detailed information on these commercial processes, and others that were studied and used in the production of cellulose-based regenerated fibers (cellulose acetate, cuprammonium, or LiCl/DMAc (*N*,*N*-dimethylacetamide)) processes and their respective advantages and limitations, we recommend referring to specialized sources.^742,363^ However, despite its advantages, the Lyocell process has a limited production in the world due to the very high industrial production requirements needed to avoid problems generated by the occurrence of secondary oxidative reactions, thermal instability, high temperatures for the dissolution process (∼120 °C), and uncontrolled fibrillation with the NMMO solvent.^747–749^

##### 2.10.5.2 ILs for Cellulose Fiber Production

To address the issues associated with the Lyocell process, the use of ILs in producing cellulose-based fibers has emerged as an alternative method for RCF production. This approach can mitigate some of the primary concerns associated with the NMMO solvent, such as side reactions and thermal stability. The ILs investigated for cellulose fiber production have been primarily selected based on their ability to dissolve cellulose, regardless of the technique utilized. The most studied are imidazolium-based ILs ([C_4_C_1_im], [C_2_C_1_im] or [(C_1_=C_2_)­C_1_im]) combined with acetate, chloride, or phosphate groups as anions and a special class of PILs called superbase protic ILs.^750–754^ Although PILs are normally used in the pretreatment of biomass because of their good lignin solubility, superbase PILs dissolve cellulose as efficiently as AILs and have been used to produce RCFs. More concretely, those PILs used are based on superbases combined with carboxylic acids, such as [DBNH]­[C_1_CO_2_], 1,8-diazabicyclo[5.4.0]­undec-7-ene acetate ([DBUH]­[C_1_CO_2_]) or 1,1,3,3-tetramethylguanidine acetate ([(C_1_)_4_Gua]­[C_1_CO_2_]), among others, which showed cellulose dissolution higher than 10 wt%. The lower viscosity of [DBNH]-based ILs relative to [DBUH]^−^ or [(C_1_)_4_Gua]-based ILs, made it the first non-imidazolium-based IL used for cellulose spinning and RCF production. The first work on ILs and RCFs production employed [(C_1_=C_2_)­C_1_im]Cl and [C_4_C_1_im]­Cl.^440,755–757^ Using those ILs the obtained fibers had similar properties as those obtained by other processes, such as Tencel (NMMO), but the main problem of Cl^–^ anion ILs is that they are corrosive and degrading toward the cellulose during the dissolution and spinning. To avoid those problems, [C_2_C_1_im]­[C_1_CO_2_] or [C_1_CO_2_]^−^ anion ILs were selected as a non-corrosive solvent for cellulose fibers production. Another advantage of using [C_2_C_1_im]­[C_1_CO_2_] is that it requires lower dissolving temperature and spinning temperature than [C_4_C_1_im]­Cl, need less energy for the fiber formation and the RCFs obtained had similar properties than those obtained using [C_4_C_1_im]Cl or the Lyocell process.^755,758^ [C_2_C_1_im]­[(C_2_O)_2_PO_2_] spinning system was also studied.^752,759^ The cellulose fibers had better properties than NMMO fibers and by varying the draw ratio they could obtain fibers with an elongation of 11% and a break strength higher than 900 MPa.^760^ This IL offers the benefit of minimal cellulose degradation at temperatures ranging from 90–100 °C. This contrasts with the [(C_1_=C_2_)­C_1_im]Cl spinning system, where significant cellulose degradation is observed as time and temperature increase. This is crucial for industrial applications and continuous fiber production, as the dope will be subjected to high temperatures for long periods of time. Additionally, it has a low viscosity and its synthesis is simplest and cheaper than that of other imidazolium ILs.^760,761^

To improve the dissolution process, ILs are mixed with co-solvents, such as DMSO or DMF to reduce the viscosity of the dope, the dissolution time, and the temperature.^762–765^ Recent work published by Zhao *et al*. studied the preparation of cellulose spinning dopes using DMSO and imidazolium ILs ([C_2_C_1_im]­[C_1_CO_2_], [C_2_C_1_im]Cl and [C_2_C_1_im]­[(C_2_O)_2_PO_2_]).^766^ They obtained fibers with high tensile strength and elongation using [C_2_C_1_im]­[C_1_CO_2_] and DMSO as the solvent mixture, discarding the use of [C_2_C_1_im]Cl and [C_2_C_1_im]­[(C_2_O)_2_PO_2_] because they have higher viscosities. Lee *et al*. compared the RCFs obtained using [C_2_C_1_im]­[C_1_CO_2_] with and without co-solvent.^764^ They observed that the fibers obtained using a co-solvent had higher values of elongation and tensile strength. Despite the improved properties of the fibers, the industrial implications of adding a co-solvent should be evaluated. Factors such as increased reagent costs, separation processes, recyclability, etc., could add complexity to the procedure and potentially hinder its industrial application.

To date, as mentioned above, [DBNH]­[C_1_CO_2_], a non-imidazolium superbase-derived IL, has proven to be the most successful for producing synthetic cellulose fibers. Ioncell is a process based on Lyocell that employs [DBNH]­[C_1_CO_2_] to generate synthetic cellulose fibers. This procedure involves dry-jet wet-spinning technology, where solutions of dissolved cellulose are extended in an air gap prior to being regenerated in a water coagulation bath. The work of Michud *et al.* showed the production steps and the properties of the produced fibers using this IL and different prehydrolysis kraft pulps.^767^ This process was called Ioncell-F, and it was developed at Aalto University in collaboration with the University of Helsinki. The obtained fibers at 75 °C spinning temperature and a draw ratio of 14 had better properties than viscose fibers. Those had higher tenacities, Young’s modulus three times higher than viscose fibers, and a homogeneous and dense fibrillar structure. Since then, improvements to the spinning and process parameters to obtain better cellulose fibers have been published.^768,769^

The advantages of these ILs are that they reduce the process temperature (dissolution and spinning) and can dissolve a wide range of biopolymers. This implies that cellulose pulp with lignin or hemicellulose could be used to produce cellulose fibers.^743,770–773^ Elsayed *et al.* studied the production of cellulose fibers using different super-base ILs 7-methyl-1,5,7-triazabicyclo[4.4.0]­dec-5-enium acetate ([mTBDH]­[C_1_CO_2_]), [DBNH]­[C_1_CO_2_] and [DBUH]­[C_1_CO_2_]), and NMMO as a reference. They observed that the fibers obtained using super-base ILs had similar mechanical properties than NMMO fibers, but fibers obtained using [DBNH]­[C_1_CO_2_] were the best.^753^ Moreover, those fibers also exhibit higher crystallinity and orientation than viscose fibers. Currently, a brand named Ioncell has built a pilot plant and established a start-up company aiming to introduce this process into the fiber market. This shows that the production of cellulose fibers using ILs is feasible and is being scaled up to higher levels.

Although dry-jet wet-spinning is the most promising method for processing IL–biopolymer solutions, adequate viscoelastic properties of the solution are a mandatory requirement. Another advantage of using ILs that could increase its application and large-scale production possibilities is the versatility of processes that can be employed to produce RCFs: dry-jet wet spinning, wet-spinning or electrospinning (Figure 52).^774–777^ For a deeper understanding of the ILs used in fiber production and the associated processes, the following publications are recommended.^363,778,779^

**Figure 52:**
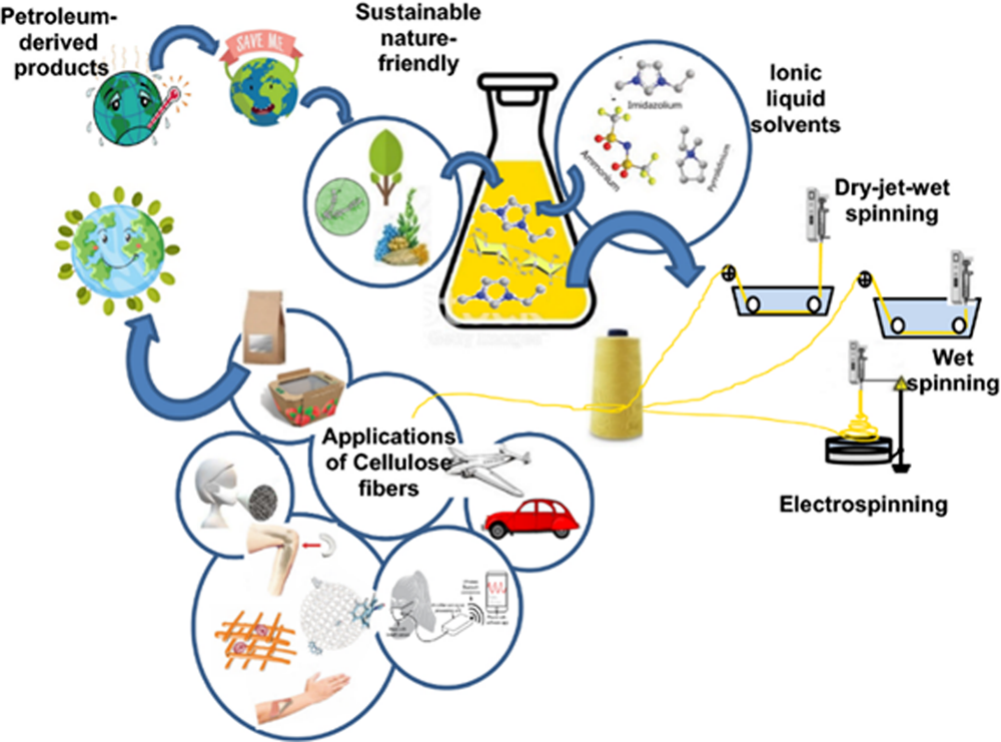
Opportunities of ILs in the production of RCFs. Adapted with permission from ref 363. Copyright 2022 Springer Nature under CC BY 4.0 (https://creativecommons.org/licenses/by/4.0/).

ILs could be employed for the extraction of RCFs from cellulose pulp streams, bringing with them multiple advantages such as low vapor pressure, versatility, and high cellulose dissolution rates. The primary concern about ILs usage lies in its toxicity and recyclability. This is a central issue associated with ILs in all aspects of material production and wood pretreatment, as previously mentioned. Typical methods for recycling ILs encompass techniques, such as evaporation, membrane separation, adsorption, and induced phase separation, among others. Of these, evaporation is considered particularly appealing for separating IL-water at an industrial scale due to its high recovery yields and straightforward operation. Recently, Zheng *et al.* studied the recyclability of 1,8-diazabicyclo[5.4.0]­undec-7-enium methoxyacetate ([DBUH]­[(C_1_OC_1_)­CO_2_]), 1,8-diazabicyclo[5.4.0]­undec-7-enium ethoxyacetate ([DBUH]­[(C_2_OC_1_)­CO_2_]), and [(C_1_=C_2_)­C_1_im]­Cl.^780^ For the IL recovery, they used a rotary evaporator and studied the properties of the RCF extracted using the recovered IL. The recovery yields were in the range of 95–97% for all the ILs. The differences were in the reuse cycles. The [DBUH]­[(C_1_OC_1_)­CO_2_] could be reused up to 10 times, while the others less than 5 times. On the other hand, the fibers obtained using the recycled ILs had similar DP and thermal stability but lower crystallinity index (CrI) with recycling. In the case of the [(C_1_≡C_2_)­C_1_im]­Cl, the thermal stability and the CrI vary considerably with more cycles, which indicate that the [DBUH]­[(C_1_OC_1_)­CO_2_] is more thermally and chemically stable and could be recyclable, underscores its immense potential for commercial application. Elsayed *et al.* also studied the recycling of the super-base ILs [DBNH]­[C_1_CO_2_] and [mTBDH]­[C_1_CO_2_], but in this case the recovery took place through a set of thermal treatments operations using a centrifuge evaporator and an agitated thin-film evaporator.^781^ It was observed that [mTBDH]­[C_1_CO_2_] could be recycled and reused without compromising its properties, and the properties of the cellulose fibers were also maintained. In contrast, [DBNH]­[C_1_CO_2_] lost its dissolution capability after just one cycle. Additionally, the toxicity of these ILs, both in their fresh state and in their hydrolyzed products, was found to be >1000 mg/L, classifying them as harmless. The recyclability and recovery of these ILs are still under investigation.^782,783^

##### 2.10.5.3 Cellulose Rich Materials (CRMs) for RCF Production

In all the processes discussed so far, the cellulose employed is invariably of high purity, originated from sources as bleached cellulose pulp or cellulose kraft pulp, among others. These cellulose sources are characterized by extremely low content of lignin and hemicellulose. The process differs significantly when a CRM is utilized. CRMs contain a variety of lignin and hemicellulose derived materials crosslinked with cellulose and depending on the process. This endows CRMs with distinct characteristics that could influence their processing methods and final properties. Consequently, the use of CRM derived from an IL biorefinery stream may pose certain challenges and, typically, require the inclusion of another processing stage. Therefore, it is important to consider the purification or bleaching of cellulose during planning for industrial production of RCFs.

Fibers composed of cellulose–lignin are typically employed in the production of carbon fibers. In such instances, the lignin is externally integrated with the cellulose, allowing to control the lignin percentage and to study its effect on the final carbon fiber.^771,784–786^

There have been few published studies on the spinnability of unbleached pulps that observed that lignin has a noticeable effect on the fiber orientation and the fiber properties. The presence of lignin reduces the crystallinity and the DP of the CRM and decreases the tensile strength and the mechanical properties of the fibers. The presence of lignin also disturbs the ordered structure formed by cellulose fibrils and reduces the total orientation of the fibers.^787,788^ Moreover, some lignin could be lost in the bath or reprecipitate during the washing stage.^784^ This could hinder the recycling of the ILs, requiring intermediate steps to remove the lignin from the coagulant bath. Apart from the lignin content, cellulose properties (*e.g.*, the type of pulp, the initial DP, or the cellulose concentration in the dope) also have a big effect on the final mechanical properties of RCFs.^741,749,682,789^

2.10.5.3.1. *Industrial Production of RCFs*. For the industrial implementation of this process, it is imperative to establish and comprehensively understand all associated parameters. Pre-treating the CRM, such as employing bleaching, might be necessary before initiating RCFs production. This step could substantially reduce the lignin content, leading to cellulose with enhanced crystallinity and DP values. Oxidizing agents, like hypochlorite and peroxide, are frequently employed as bleaching agents. Typically, the bleaching process works by oxidizing water-insoluble lignin to break specific bonds (such as aryl ether bonds, carbon–carbon bonds, or β-O-4 bonds), producing water-soluble byproducts including aromatic aldehydes and carboxylic acids. These water-soluble products can then be removed by washing.^790,791^ Such pre-processing would lower the T required for dissolution, obviate the necessity for a separate lignin extraction procedure from the coagulant bath and enhance the uniformity of the end products. Though these oxidative bleaching agents can effectively remove lignin, they also risk degrading the cellulose substrate if the bleaching conditions are not properly managed. This can result in reduced yield and compromised strength of the cellulose fibers. Thus, while it is essential to consider the bleaching conditions, one must also weigh the economic consequences of introducing new reagents and equipment to the process. Equally important is evaluating the recyclability and environmental impact of these additions in achieving the desired cellulose purity. Securing a high-purity cellulose pulp from a biorefinery stream becomes pivotal to circumvent extra expenses and facilitates the production of more affordable cellulose fibers. Moreover, issues concerning toxicity and solvent recycling efficiency are paramount for the large-scale industrial production of RCFs using ILs, particularly when obtaining CRM from an IL biorefinery.

##### 2.10.5.4 Cellulose Gel-Like Materials

The use of ILs to develop cellulose gel materials has expanded over the years due to the versatility of these compounds and the variety of gel materials that can be produced. Different cellulose sources, such as pulp cellulose (kraft or bleached), CRM, cotton, MCC and cellulose derivatives, are utilized depending on the specific IL and the targeted properties of the end product. The formulation process begins with dissolving the cellulose in the IL. Once fully dissolved, there are two primary methods to produce cellulose gel materials: direct gelation or coagulating the mixture with a solvent, often water.

2.10.5.4.1. *Ionogels*. Ionogels are a class of materials with an IL encapsulated within a 3D network where a polymer, as cellulose, serves as the matrix, offering high mechanical properties. They combine key advantages of ILs, including high ionic conductivity, low volatility and high resistance to flammability with the properties offered by the presence of the polymer. More concretely, cellulose gives the material good mechanical properties and a stable 3D network, while the IL provides flexibility and electrochemical properties, opening avenues for diverse industries, including electrochemical applications (as electrolytes), medical uses (such as electronic skin applications), 3D printing and more.^792^

Cellulose ionogels have garnered interest due to their notable electrochemical and mechanical attributes.^793^ There are two approaches to gel the IL–cellulose mixture: physical and chemical. Physical gelation can be achieved simply by exposing the mixture to air (or in a controlled environment like a climate chamber). In this method, cellulose regeneration occurs as a result of ambient humidity. This happens due to the regeneration of the hydrogen bonds of the cellulose that is insoluble in water. The inter- and intra-chain bonds of the cellulose are regenerated and the IL gets trapped in the structure, forming VdW and electrostatic linkages with the cellulose, leading to the formation of the ionogel.^794–796^ On the other hand, the chemical gelation is due to chemical reactions, polymerization, or radiation.^792,797^ The resulting gel, independent of the method used, is referred to as an ionogel (also known as iongel or IL gel).

The ILs most employed up to date in the formulation of cellulose ionogels are based on imidazolium, with anions such as halide (Cl^–^, Br^–^, I^–^), acetate and phosphate. At the same time, there are other ILs that are also used, but as additives, such as ILs based on [(CF_3_SO_2_)_2_N]^−^ or [BF_4_]^–^, also known as non-dissolving ILs (ILs used to improve the ionic mobility, without interacting with the cellulose).^795^ Studies have also included the formulation of ionogels using ILs such as [Ch] based-IL combined with AAs as anions to obtain biodegradable and biocompatible ionogels.^796^

In physical cellulose ionogels, the interactions between the cellulose and the IL are noncovalent. Specifically, these interactions consist mainly of H-bonds, VdW, forces and/or electrostatic forces.^793^ Due to these characteristics, their preparation does not demand a complex medium, specialized reagent or intricate reaction mechanisms. The most crucial steps in the formulation of physical cellulose ionogels are the dissolution of the cellulose and the gelation of the mixture. The dissolution mechanism has been commented on and studied. But the gelation is not as well-known as the dissolution. The ionogel formation does not always occur. It depends on the IL, the cellulose concentration and the cellulose source. Kadokawa *et al.* described the first ionogel formulation, leaving a MCC-[C_4_C_1_im]Cl mixture at r.t. for seven days.^794^ The mechanism was not specified, but since then, many ionogels have been formulated and studied. The H-bonds formed between the hydroxyl (−OH) groups of cellulose and the cation and anion of certain ILs initiate the dissolution process. However, the displacement of these IL–cellulose interactions by water–cellulose bonds might serve as the starting point for gelation.^798^ The gelation of the ionogel is mainly attributed to the re-establishment of intermolecular and intramolecular cellulose H-bonds and the breaking of some IL–cellulose linkages. The water present in cellulose ionogels suggests that this bond formation and breakage might result from water absorption. When exposed to environmental conditions, the cellulose–IL mixture absorbs ambient moisture, leading the cellulose to form water-insoluble aggregates. These aggregates serve as gel crosslinking points, promoting chain entanglement and supporting the restoration of the H-bonds of cellulose.^799^ Thus, the hydrophilicity of many ILs facilitates the formation of physical cellulose ionogels, making it a prerequisite for such applications. Physical cellulose ionogels obtained this way are reversible, *i.e.*, they can be reversed to its original fluid form by heating to 100–120 °C, which is an advantage because they could be reusable.^684^ Physical ionogels offer the benefit of straightforward production and acquisition. The process involves just a single step of dissolution, wherein cellulose and any additives are dissolved in the IL, followed by gelation. Depending on the specific procedure, this gelation might occur at r.t., within a climate chamber, or through water coagulation.^796,800–806^ From an industrial perspective, this simplicity is a significant advantage of using ILs. The dissolution and material formulation follow a seamless sequence, with the IL used entirely, leaving no residues during the process.

On the other hand, chemical ionogels are formulated generally by three primary methods: (1) cross-linking functional groups such as −OH, −COOH and −NH_2_ with cross-linking agents including aldehydes and carboxylic acids through covalent bonds, (2) using gamma, X-ray or e-beam radiation to modify polymers via free radicals, and (3) grafting/*in situ* polymerization.^795^ Chemical ionogels have the advantage of stability, rigidity and versatility. They are more stable materials and could be obtained following many different routes, that combined with the multiples ILs types, a wide range of ionogels could be formulated.^797,807–809^ Recently, a non-imidazolium IL was used for the formulation of a chemical ionogel. Seiler *et al.* found that *N*-butyl-*N*-methylpyrrolidinium hydroxide in an aqueous solution ([C_4_C_1_pyr]­[OH] aq) can dissolve up to 20 wt.% cellulose at rt and form an ionogel using ECH as the crosslinker.^810^ They formulated ionogels with good mechanical properties, reaching a maximum strength of 70 kPa, and with good antibacterial and antimicrobial properties. The main disadvantages of chemical ionogels are that they need more reagents than the IL, different dissolution and processing conditions, depending on the reaction, and the possibility of undesired side reactions during their preparation.

Physical and chemical cellulose ionogels have promising properties due to the combination of cellulose and IL properties. However, in many studies, to enhance the electrochemical, mechanical or other properties of the ionogels, additives or other ILs (non-dissolving ILs) are often added to the cellulose–IL mixture.^795,811,812^ An example is the work of Kasprzak and Galiński,^813^ where cellulose-based ionogels are formulated using [C_2_C_1_im]­[C_1_CO_2_], and then the ionogel is immersed in [C_2_C_1_im]­[BF_4_], a conductive IL, to improve the electrochemical properties of the ionogel.

Using cellulose ionogels opens avenues for creating a range of materials. By immersing cellulose ionogels or the IL–cellulose mixture in solvents, such as water or alcohol, the IL in the solid matrix gets replaced and initiates bonding with the cellulose. This approach can lead to the formation of hydrogels, films, aerogels or organogels. The cellulose structure can differ based on the antisolvents used during the coagulation of the IL–cellulose mixture or ionogel, as evidenced in various studies.^814,815^

2.10.5.4.2. *Hydrogels*. The process of formulating hydrogels from an IL–cellulose mixture follows a procedure similar to the RCF process. The mixture or ionogel is immersed in water, leading to the displacement of the IL within the 3D network of the gel, resulting in the formation of H-bonds with cellulose and its regeneration. The formulation of cellulose hydrogels using IL solutions or ionogels is a widely recognized method that has attracted substantial attention, as indicated by the recent review of Taokaew, where hydrogels obtained from ILs–cellulose mixtures are revised.^816^ Cellulose hydrogels find applications in diverse fields including medicine, agriculture, water treatment and 3D printing, to name a few.

The anion of the IL plays a role in the kinetics of coagulation during cellulose regeneration. For instance, with ILs containing the [C_1_CO_2_]^−^ anion, introducing water as an antisolvent leads to the breakdown of the H-bond between cellulose and the [C_1_CO_2_]^–^ anion. This simultaneously triggers the development of hydrogen bonds among cellulose molecules, while [C_1_CO_2_]^–^ establishes an H-bond with water. As cellulose chains come together, this prompts gelation, causing the cellulose to precipitate from the IL/protic solvent mixtures.^798,817–819^ Notably, this gelation occurs without the need for a chemical crosslinker, attributed to cellulose's high entanglement density. As occurred with ionogel formation mechanisms, the cellulose regeneration and hydrogel formation is also inherently difficult to study.

Numerous studies have produced cellulose hydrogels from IL–cellulose mixtures, wherein the ILs must be water-soluble. In such way, cellulose hydrogels were obtained from [C_2_C_1_im]­C], [C_2_C_1_im]­[C_1_CO_2_], [C_4_C_1_im]­Cl, [C_2_C_1_im]­[(C_x_O)_2_PO_2_] ILs, [(C_1_=C_2_)­C_1_im]­Cl, and mixtures of any of those ILs and co-solvents such as DMSO.^820–826^ In these studies, the procedure is largely consistent: the IL–cellulose mixture is immersed in water, and the water is replaced repeatedly until the IL is fully removed and the hydrogel is obtained. These hydrogels are called physical cellulose hydrogels, because no crosslinker or reagent was used for the hydrogel formation. In other work, chemical cellulose hydrogels are also produced from the IL–cellulose mixture adding crosslinkers.^827^ Cellulose hydrogels obtained through the use of ILs hold potential for diverse applications, as elaborated in the work by Taokaew.^816^ Similar to earlier methods, the IL–water mixture requires treatment, with the IL undergoing regeneration for reuse to make the process industrially feasible. As with RCFs, the IL can be recovered through an evaporation method. However, in this context, the the recovery and reusability of the IL have not been explored. This might be due to the perception that industrial production of cellulose hydrogels using ILs seems further away compared to the production of cellulose fibers.

2.10.5.4.3. *Organogels*. The obtention of organogels or alcogels from IL–cellulose mixture is similar but uses an alcohol in place of water.^828^ These materials have not been extensively investigated like hydrogels because the properties of the mixture after the coagulation with ethanol or another alcohol are inferior to those of hydrogels. The regenerated cellulose exhibits increased crystallinity and enhanced thermal stability when water is used as the solvent.^829^ As an antisolvent, water facilitates the reorientation of molecular chains and helps rebuild a structured cellulose framework. In contrast, using ethanol as an antisolvent causes the cellulose chains to cluster in a relatively loose and disorganized way.^815,819^

2.10.5.4.4. *Aerogels and Films*. Aerogels and films are commonly produced from cellulose hydrogels or alcogels through different drying methods. The primary technique for creating aerogels involves supercritical CO_2_ drying of the hydrogel. Since CO_2_ is not compatible with water, the aerogel's pore structure is at risk of collapsing. Therefore, the water in the hydrogel must be substituted with alcohol, usually ethanol, before the drying process. To ensure the hydrogel’s structure remains intact, a stepwise water replacement is performed using water–ethanol mixtures of gradually increasing ethanol concentrations.^830,831^ They also could be obtained directly from the alcogel by displacing the IL with an alcohol.^832^ Numerous research studies focus on producing cellulose aerogels from IL dissolution, driven by the potential to create a fully biodegradable material (porous cellulose structure). These aerogels, boasting a high specific surface area and porosity, hold promise for applications in areas such as acoustic and thermal insulation, water treatment and in the biomedical sector, all while utilizing a solvent that is easily recyclable.^833–841^ Négrier *et al.* demonstrated in their work that the formulation of aerogels, xerogels, and cryogels is possible using a mixture of an IL ([C_2_C_1_im]­[C_1_CO_2_] or [DBNH]­[C_1_CO_2_]) with DMSO (Figure 53).^831^ They found that a medium and low molecular weight (or DP) cellulose did not significantly affect the properties of the materials, but that in the case of high molecular weight cellulose, the phase separation highly affected the properties of the porous cellulose. They also found that the choice of IL did not affect the properties of the materials.

**Figure 53:**
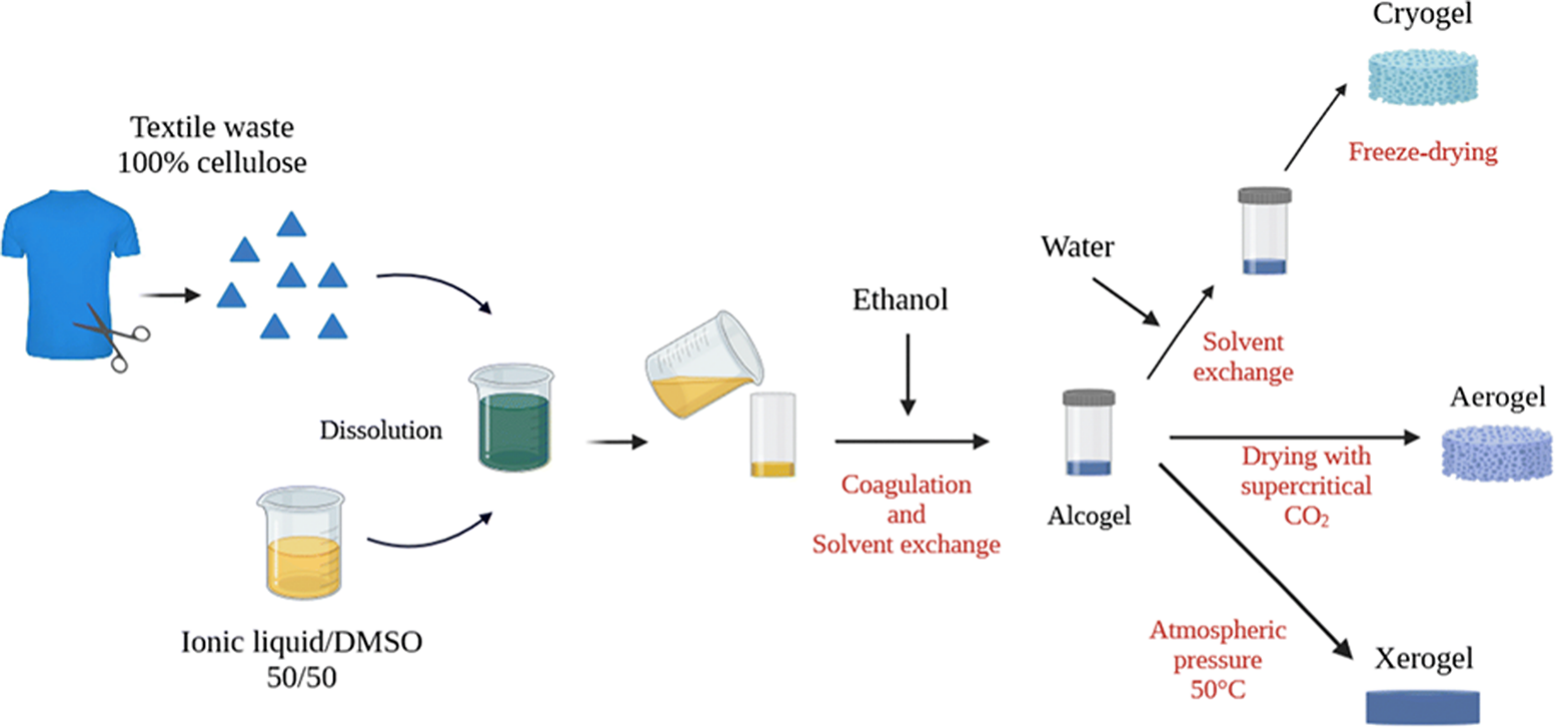
Example of cellulose dissolution and material formulation using ILs and DMSO. Adapted with permission from ref 831. Copyright 2023 Royal Society of Chemistry under CC BY 4.0 (https://creativecommons.org/licenses/by/4.0/).

The process of formulating cellulose films using ILs is well-established, with numerous recent studies exploring these materials. These investigations often delve into the relationship between the properties of the film and the coagulants or raw materials used in their production.^842–845^ Cellulose films are generally formulated by drying the hydrogel in a conventional oven or vacuum at 60 °C during more than 8 h, pressing the hydrogels (∼100 kPa) between two glass Petri dishes or drying them at rt.^846–849^ These films are commonly developed for use in food packaging, attributed to their mechanical, barrier and optical qualities. Additionally, their properties can be easily enhanced by incorporating additives like plasticizers to bolster barrier attributes or by undergoing post-modifications to yield cellulose films with antibacterial or antioxidant characteristics.^70,850^

##### 2.10.5.5 Cellulose Rich Materials for Gel Formulation

The objective of this section is to highlight the potential of cellulosic pulp, derived from an IL biorefinery, to serve as a raw material in industrial-scale production of novel materials. Typically, all the gel materials commented on in the previous section were formulated using MCC, cellulose derivatives or kraft pulp cellulose as the primary components. Therefore, they used highly pure cellulose sources with notable crystallinity or DP, depending on the origin, and the mechanical properties of cellulose gels are influenced by the initial cellulose material used. For example, cellulose gels derived from cellulose pulp with a high degree of polymerization can exhibit a notably higher compression modulus compared to those prepared from MCC.^70^ There have been some reports where cellulose gel-like materials are formulated using lignocellulose, cellulose and lignin, non-pure cellulose from waste newspapers, or a CRM from organosolv or ionoSolv processes, among others.^818,851–853,359,854–858^ The addition of lignin increases the dissolution time or temperature and, depending on the IL, sometimes the dissolution is not possible. However, retaining or adding lignin might present several advantages. For instance, ionogels crafted using a CRM from organosolv pretreatment, containing 10% lignin, demonstrated superior rheological properties compared to MCC ionogels.^359^ This was attributed to the presence of lignin, but was also likely influenced by the cellulose properties. MCC has a low DP compared with most pulp cellulose obtained by biorefinery or pulping processes, so the stiffness or strength of the material will be reduced. A comparable pattern was noted in hydrogels.^851,859^ The presence of lignin improved water permeation rates, swelling capacity, and compression modulus in hydrogels.^860^ Additionally, it diminished the interactions between cellulose and drugs, and release studies indicated more favorable outcomes compared to using pure cellulose alone.^861^ Furthermore, when it comes to cellulose films or aerogels, the inclusion of lignin not only enhances the mechanical properties but also introduces beneficial features such as antibacterial, antimicrobial and UV-shielding capabilities.^862–866^ It was also observed that these gels exhibit properties on par with others produced using cellulose and traditional physical or chemical methods, such as NaOH dissolution and polymerization.^838^

When utilizing CRM or pulp cellulose from biorefinery streams, the presence of lignin can pose challenges. It is not just the quantity of lignin that is of concern but also its type and how it is integrated into the solid. A clear distinction exists between an IL that treats wood by dissolving cellulose and one that dissolves lignin. In the former scenario, cellulose undergoes modification, while in the latter, delignification takes place. During biomass dissolution, the cellulose structure shifts from cellulose I to II. Conversely, when solubilizing lignin, the cellulose crystallinity generally remains unchanged.^362^ It is therefore imperative to investigate how these variations impact the IL dissolution of each type of CRM because it will affect the feasibility of the material formulation. Xia *et al.* formulated cellulose hydrogels and aerogels using LiCl/DMSO as the solvent with different amounts of lignin.^864^ They observed that endogenous lignin had different properties than exogenous lignin, for example, as occurred in the case of the viscoelastic properties. The explanation that they gave is that during the gelation of composite gels, exogenous lignin is regenerated as particles, settling on the surfaces of the cellulose chains within the gels. These encapsulated lignin particles limit the mobility of the cellulose chains, leading to an increase in the elastic modulus. In contrast, for cellulose gels, a higher concentration of endogenous lignin in lignocellulose can impose a pronounced constraint on the separation of cellulose and hemicellulose. This results in a film-like structure, decreasing the interlocking of the cellulose or hemicellulose chains during gel formation, resulting in a looser structure. This is important information for the formulation of CRMs where the lignin, depending on the biomass IL pretreatment, will have different interactions with the cellulose. More studies using a CRM should be performed, other than cellulose and lignin materials that generally used ECH for the cellulose-lining crosslinking.

2.10.5.5.1. *Industrial Scale Production of Cellulose-Based Gels and Films*. The industrial-scale production of ionogels remains largely unexplored, even on a pilot scale or comparable levels. Currently, their production is restricted to laboratory settings, without comprehensive assessments of their reusability or recyclability. This limitation arises from the absence of available data concerning the environmental and operational implications of their production. A similar situation is observed in the production of hydrogels, aerogels or films. Comprehensive analyses of the processes and in-depth studies on the environmental impacts and the effects of pilot or large-scale production for these materials are notably lacking. As the scale of production increases, the dissolution of cellulose becomes more intricate, demanding extended durations and elevated temperatures. This challenge hampers the scalability of small-scale methods. Consequently, there is a need for more comprehensive research involving greater volumes of IL and cellulose to discern a relationship. As a result, the potential for industrializing these materials remains uncertain, emphasizing the importance of more intensive experimentation on larger scales.

##### 2.10.5.6 Nanocellulose

Nanocellulose, derived from native cellulose, is distinguished by its nanoscale dimensions. When reduced to this scale, cellulose showcases impressive attributes, making it sought-after for numerous applications. Being lightweight and biodegradable, combined with its exceptional mechanical strengths; like high tensile strength and rigidity, nanocellulose is ideal for a range of sectors including electronics, food, paper, packaging and medicine. Cellulose can be sourced from a variety of organisms such as bacteria, plants and algae. The source not only affects the size and characteristics of the cellulose but also the energy consumption in the extraction process to produce nanocellulose.^867^ Nanocellulose comes in several distinct forms, each with unique properties and potential applications. Cellulose nanocrystals (CNCs) are small, rod-shaped particles that have high crystallinity and rigidity, with a diameter of 5–30 nm and length of 100–500 nm. Their properties make them useful for reinforcing composites and forming films and coatings. Cellulose nanofibers (CNFs) or nanofibrillated cellulose (NFC) are long, flexible fibers that can form a network of strong hydrogen bonds and they have a diameter of 5–70 nm and are a few micrometers in length. They are useful in applications where high strength and flexibility are required, such as in paper products, filters and certain types of composites. Finally, bacterial nanocellulose (BNC) is produced by certain types of bacteria. It forms a highly hydrated gel and can be produced in pure form without the need for chemical treatments to remove noncellulose components. All forms of nanocellulose have the same chemical composition and offer a combination of lightweight, high strength and biodegradability, which makes them attractive for a wide range of uses.^868^ Some properties such as morphology, crystallinity and particle size depend on the extraction method and the source.

The production of CNC and CNF using ILs was studied due to the necessity of solving some problems related to the conventional processes, such as the high energy consumption, low yields or environmental hazards associated with the use of concentrated acids and other chemicals (Figure 54).^869^ Moreover, acid hydrolysis, the most common method employed for nanocellulose extraction, needs multiple purification steps, which is a challenge for large-scale production. In contrast to traditional methods that require severe operating conditions, the remarkable capabilities of ILs, especially imidazolium-based ILs, in swelling, dissolving and hydrolyzing cellulose under standard environmental conditions, present an exciting opportunity for efficiently converting diverse lignocellulosic materials into nanocellulose.^870^

**Figure 54:**
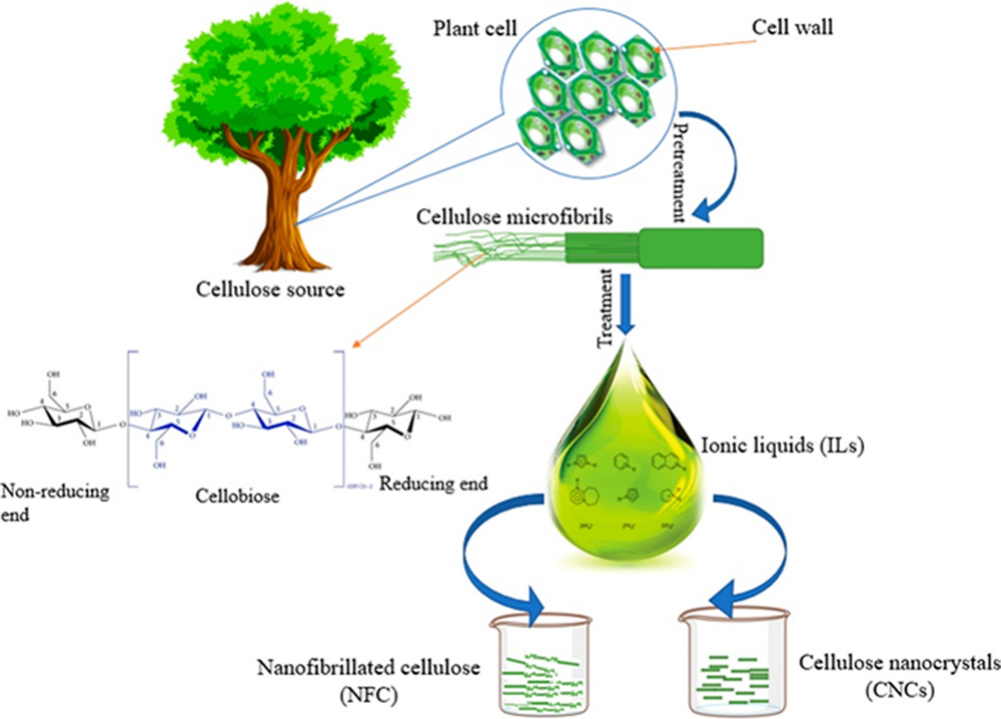
Nanocellulose and nanofibrillated cellulose obtained using ILs. Adapted with permission from ref 869. Copyright 2021 American Chemical Society.

Notably, ILs such as [C_4_C_1_im] Cl, [C_2_C_1_im]­[C_1_CO_2_] and [C_4_C_1_im]­[HSO_4_] have been commonly employed in nanocellulose production due to their exceptional capability to dissolve and/or hydrolyze cellulosic materials.^869,871,872^ Some advantages of ILs are their recoverability and the possibility to obtain NC with better properties than conventional methods. The recovery of the IL is a challenge and is the main drawback to be solved for large-scale production. Phanthong *et al*. produced NC following a one-step process by the combination of ball milling with [C_4_C_1_im]Cl at r.t.^873^ They studied the NC properties and the IL recovery. The NC produced had good properties such as high crystallinity, thermal stability and crystal size. They found that the [C_4_C_1_im]Cl could be reused four times without losing nanocellulose properties. This reusability was also observed by Paredes *et al*. using a PIL with an anionic cluster ([C_4_im]­[HSO_4_(H_2_SO_4_)_1_].^874^ They could reuse the IL four times without losing CNC yields and without any change in the IL composition. This was also observed in other work, where the recovery yield of ionic liquid was usually high, >90%. For example, in the recent work of Rasri *et al*., where the [C_4_C_1_im]­[HSO_4_] was recovered after nanocrystal extraction with a yield of 88%, indicating that it could be reused multiple times, reducing the overall costs of the process.^875,872,876,877^ Moreover, Paredes *et al.* demonstrated that NC properties were improved by using ILs.^874^ The IL was used as a solvent and catalyst, and they obtained NCs using different cellulose sources: MCC, cellulose extracted from corn husk, and cellulose powder. The properties of the CNC were similar or better than those obtained by acid hydrolysis extraction method, with high yields (>60%), CrI (>55%), good crystal dimensions, and high thermal stability, similar to what was obtained in other work.^875,877–882^ ILs have also been combined with other components, such as co-solvents (DMSO), other ILs, inorganic acids or with enzymatic pretreatments, to improve the process.^883–885,870^ In those cases, the IL is used to dissolve the cellulose and the mixture was formulated to improve the nanocellulose yields. The production of nanocellulose using ILs has many advantages and the improvement of the properties has been repeatedly demonstrated. The IL also plays a fundamental role by reducing the number of steps and the environmental impact of the process. More examples of processes using IL to obtain nanocellulose can be found in recent reviews.^869,872,886^

The use of ILs as solvent and catalyst combined with their easy recovery, reduces the steps needed for the nanocellulose obtention, *i.e*., the production of NC using ILs is simpler than conventional methods, where more intermediate steps are required. The use of DESs in the production of nanocellulose has also been widely studied. Their environmentally friendly and biocompatible nature, along with their low toxicity, ease of preparation, adjustability and recyclability, makes them a compelling substitute for isolating nanocellulose.^887^ The recovery yields and the reusability of DES after NC extraction should also be studied. The properties of the NC obtained using DES are like those extracted using ILs or acid hydrolysis. For DES extraction, there are two different pretreatments: non-derivatizing and derivatizing. Wu *et al.* performed CNC and CNF production from MCC using two DES: [Ch]­Cl:formic acid (FAc) and [Ch]­Cl:urea followed by ball milling.^888^ In this study, both pretreatments are present: non-derivatizing when they use [Ch]­Cl:urea and derivatizing when they use [Ch]­Cl:formic acid. Acidic DESs, made up of [Ch]Cl and carboxylic acids, can cause esterification of cellulose, which subsequently aids in the nanofibrillation process during mechanical fibrillation. Wu *et al.* obtained NC from both methods,^888^ and observed that the [Ch]­Cl:urea nanocellulose had higher yields, similar CNC dimensions, lower zeta potential, lower CrI, and higher thermal stability than [Ch]­Cl:FAc DES, as occurred in other work.^889–891^ In both cases, there are some advantages and disadvantages that could be useful depending on the application. The reusability of DES was also demonstrated. For [Ch]­Cl:urea and [Ch]­Cl:FAc DES, high recovery rates of >95%, and properties of the extracted NC (dimensions and yields) similar to those of fresh DES were observed.^888^

2.10.5.6.1. *Cellulose-Rich Materials (CRM) for Nanocellulose Production*. As with all cellulose materials, most of the research about nanocellulose utilizes cellulose that is free from lignin (or bleached) to produce nanocellulose. In the production of IL or DES nanocellulose, as well as in broader applications, various sources of cellulose are used. These commonly include MCC, α-cellulose, wood pulp, cotton cellulose and bleached Kraft pulps derived from different types of wood.^867^ However, unbleached fibers containing residual lignin, as the CRM or cellulose pulp obtained from biorefinery streams represent an alternative raw material to produce nanocellulose. Utilizing unbleached pulp, or pulp that has not undergone significant delignification, could lower the reliance on chemicals and energy consumption in nanocellulose production. In addition, the lignin has been reported to have several advantages such as high thermal stability, UV-blocking properties or antioxidant activity.^892–894^ The production of nanocellulose from cellulose rich materials with different bleaching treatments and lignin content have been extensively studied and are known as lignin-containing nanocellulose or LNC. Depending on the type, they could be lignin-containing cellulose nanofibers (LCNF) or lignin-containing cellulose nanocrystals (LCNC). Often the biomass is bleached to obtain a variety of lignin contents in the final pulp or CRM. The biomass is usually partially bleached, using different methods such as sodium chlorite, NaOH, alkaline hydrogen peroxide, ILs or DESs, among others.^876,893,895–902^ Lignin percentages vary from traces to 30–40 wt%. The production of those materials is the best approach to determine if the production of nanocellulose could be possible using a CRM stream from an IL biorefinery.

A reduction in the lignin content from the biomass is necessary to obtain high quality nanocellulose, with high yields and good fibrillation. When the lignin content increases, only some properties are improved. Thermal stability, UV-shielding, and hydrophobicity increase when lignin is not completely removed, but the nanocellulose production yield and the CrI decrease. At high lignin contents (>20 wt%), the mechanical fibrillation is not efficient because there are strong, cross-linked structures between cellulose and lignin, generating LCNFs with a weak fibril network.^903,904^ In the production of LCNC the lignin content reduces the yield of cellulose nanocrystals due to the reduction of the crystalline proportion in the cellulose material.^905^ There is therefore not an optimum lignin percentage to produce LNC. For example, in the work of Yuan *et al.*, they produced LCNF using a pulp bleached with sodium chlorite.^904^ The lignin content varied from 2.8 to 28.8 wt.%, and the nanofibers were obtained by mechanical fibrillation. In this work, the best LNCF was those with a lignin content of 6.8 wt%, which represents a lignin content that is sufficient to improve the defibrillation and produce long and flexible LCNFs. This increases the possibilities for the utilization of the biorefinery stream to produce LNC materials. Even though these lignin-containing materials are not perfect, they are promising and useful for diverse end-use applications.^898,899^ The production of films or nanopapers from nanocellulose is a typical final application and those are highly improved by the presence of lignin in their structure. The mechanical and thermal properties are improved, increasing the elongation resistance and the thermal stability of the films.^893^ The hydrophobicity, antibacterial, antioxidant and UV-blocking play an important role in the application of those materials in food packaging and is being widely studied.^898,906,907^ For further information, we recommend the review of Kumar *et al*.^904^

The use of ILs to produce LCNC or LCNF is less studied, and there are only few works where they are used.^881^ One example is that of Ferreira *et al.*, who studied the utilization of PILs, [(OH)^2^C_2_N]­[C_1_CO_2_], *N*-methyl-2-hydroxy-ethylammonium acetate ([(OH)^2^C_2_C_1_N]­[C_1_CO_2_]) and 2-hydroxy-diethylammonium acetate [((OH)^2^C_2_)_2_N]­[C_1_CO_2_], to produce LCN. They obtained maximum yields of 60%, zeta potential higher than 30 mV, and good thermal stability.^905^ The utilization of DES is more extended, and there are many more examples where LCN is successfully produced, sometimes directly from the pretreatment of the wood. Shu *et al.* produced LCN directly from the CRM extracted from the DES pretreatment of poplar wood.^901^ The CRM was directly washed and then disintegrated with a microfluidizer. In this case, the LNC showed cellulose I crystal structure yields higher than 60% with a lignin content of 27%, good dispersion in water, and high thermal stability.^900^ Xie *et al.* produced LCNF using a CRM directly obtained from DES pretreatment, but they also prepared LCNF films, with good mechanical and barrier properties, UV adsorption and thermostability.^907^ The recycling of DES was also successful, with a slight reduction in the pretreatment efficiency.^896^ An example of all of these processes is shown in Figure 55.^907^ For further information, we highly recommend the work of Almeida *et al*.^887^

**Figure 55:**
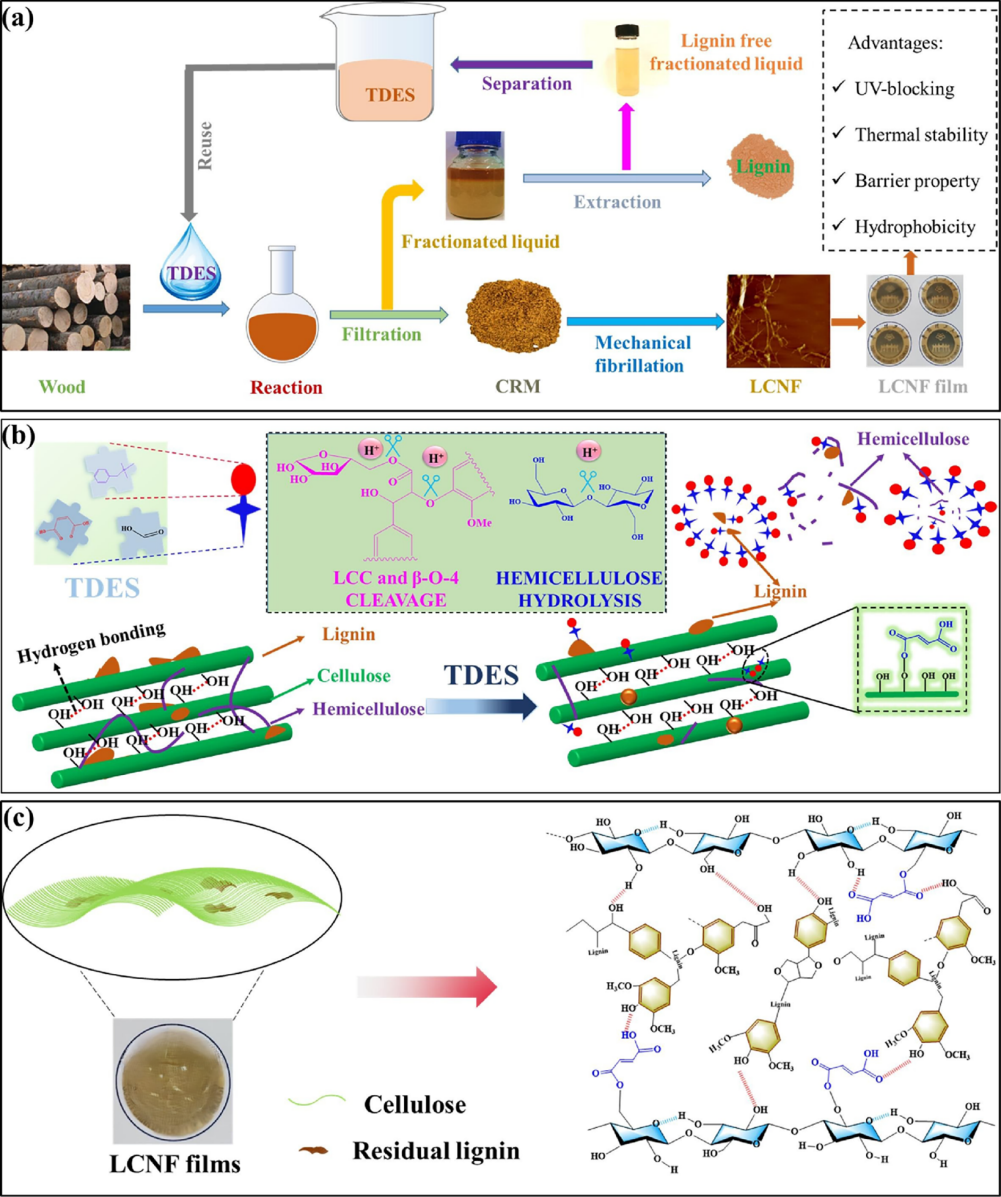
LCNF films production using DES. Adapted with permission from ref 907. Copyright 2023 Elsevier Ltd.

In conclusion, the prospect of producing nanocellulose (either CNF or CNC) using a CRM sourced from the IL biorefinery stream is immense. The application of ILs and DESs in this formulation has been extensively researched in recent years. The benefits of these methods include the potential for solvent reuse and a reduction in the number of steps required to procure these materials. The ability to produce nanocellulose directly from the fractionated stream, eliminating the need for a bleaching step, significantly reduces the resources, processing time, and reagents needed for production. Furthermore, it has been proven that DESs and ILs are reusable in almost all cases.^907,908^ The properties of nanocellulose are unaffected using recycled IL or DES and the pretreatment process is barely impacted by this recycling. The extended applications of these materials include their use as Pickering emulsifiers, in film production for food packaging, face masks, and within the paper and board industries.

2.10.5.6.2. *Industrial Scale Production of Nanocellulose*. The industrial application of LCN utilizing ILs or DESs as solvents holds great promise. However, as for ionogels, there is a notable lack of information concerning the scalability of these processes. Further research and experimentation are imperative in these domains to comprehend their behavior at larger scales and ascertain the feasibility of industrial-scale production of these materials. Simultaneously, the necessity for mechanical pretreatment of the CRM to obtain the final nanocellulose significantly amplifies the process costs, primarily driven by energy consumption and the scale and specifications of the required equipment. Therefore, it is crucial in this context to target high-value industries to ensure the profitability of the process.

#### 2.10.6 Conclusions on the Production of Cellulose-Based Materials from Lignocellulose Using ILs and DESs

IL technologies facilitate modifications to cellulose microstructure, yielding materials with innovative architectures and configurations. This presents an emerging field of research with vast potential for industrial applications. One of the primary challenges in crafting cellulosic materials from ILs revolves around understanding crystallization and cellulose regeneration. These factors directly influence the physical and chemical stability, as well as the mechanical strength of cellulose.^363^ When scaling up the production of hydrogels, aerogels, or films, there is a need to examine the mass transport diffusion between water and the IL–cellulose mixtures. Factors such as residence time in reactors and potential interactions between water, IL and cellulose become crucial.^909^

For RCFs, the challenge lies in the requisite pretreatments for CRM or cellulose pulps. Achieving high-purity cellulose devoid of lignin or hemicellulose is essential to match the properties of fibers produced through the viscose process. Consequently, the environmental and economic implications of these additional steps and reagents must be assessed to ensure a sustainable process. The production challenges of ionogels, hydrogels or aerogels lie in identifying a cost-effective end application that justifies the use of ILs in both pretreatment and material formulation. One potential solution might involve leveraging the same IL in both pretreatment and material production stages, aiming for a more integrated, circular and sustainable process.

### 2.11 Lignin Products

#### 2.11.1 Lignin First Biorefinery Concept

Traditional biorefining methods of lignocellulosic biomass involve partial destruction of the cell wall matrix and enzymatic conversion of cellulose and hemicellulose into sugars, generating a lignin-rich waste product. Unlike traditional biorefining methods, lignin first-biorefining processes are capable of selective depolymerization of lignin and leaving cellulose and hemicellulose intact.^452^ These methods of lignin-first biorefining focus on the active stabilization of lignin during biomass fractionation in addition to passive lignin stabilization by mild fractionation methods. Selective depolymerization of lignin from biomass prevents undesirable and irreversible condensation of lignin molecules during fractionation. Moreover, selective delignification eliminates the need for additional fractionation and purification steps, thus simplifying the operation and reducing production costs. Energy-intensive harsh fractionation methods used in traditional biorefining facilitate the cleavage of β-O-4 linkages and formation of C–C bonds, producing condensed lignin difficult to depolymerize in subsequent biorefining steps. Thus, lignin first-biorefining processes focus on adopting mild fractionation strategies to stabilize β-O-4 linkages and active stabilization of lignin monomers and intermediates (during fractionation) to prevent condensation of lignin. Active stabilization of the lignin monomers and other intermediates can be directed to produce desired lignin structures during the fractionation process. Such stabilization approaches can directly deliver unique target chemical molecules during the fractionation step without the need for further chemical alterations.

The mild fractionation methods commonly considered for the passive preservation of β-O-4 linkages in lignin structure including IL assisted fractionation may play an important role. For instance, future efforts on IL-assisted lignin first biorefining may focus on increasing the efficiency using both ionic components, discovery of metal containing systems, identifying the potential use of nanotechnology and high-performance computing in lignin dissolution and depolymerization.

#### 2.11.2 Biochemicals and Fuels from Lignin

The depolymerization of lignin into chemicals and fuel ensures near-complete utilization of lignocellulosic carbon and is deemed necessary for biorefinery economics and sustainability. Considering this, different strategies, including thermochemical, electrocatalytic, and biocatalytic, have been explored for the successful conversion and utilization of lignin. In this regard, ILs and/or DESs are attractive as they can be used as both solvent and catalyst for lignin depolymerization.

Compounds of various molecular weights were identified among the products from lignin degradation, offering potential as intermediates to produce fuels and chemicals.^910,911^ The primary challenge in achieving selectivity during lignin depolymerization arises from its intricate three-dimensional network connected by C–C and C–O–C linkages. Given that β-O-4 aryl ether bonds constitute 48–60% of lignin's structure, research into lignin depolymerization catalyzed by ILs has predominantly focused on cleaving these bonds.^912–915^ It has been observed that the type of anions in ILs, rather than their acid strength, significantly influences the depolymerization activity of lignin, as well as the distribution and composition of resulting products. Zakaria *et al*. investigated the depolymerization of regenerated lignin from rice husk using a range of ILs with different anions: 1-methyl-3-(3-sulfopropyl)-imidazolium chloride, [(HO_3_S)^3^C_3_C_1_im]­Cl, 1-methyl-3-(3-sulfopropyl)-imidazolium acetate, [(HO_3_S)^3^C_3_C_1_im]­[C_1_CO_2_], and 1-methyl-3-(3-sulfopropyl)-imidazolium hydrogen sulfate, [(HO_3_S)^3^C_3_C_1_im]­[HSO_4_]).^916^ They found that [(HO_3_S)^3^C_3_C_1_im]­[HSO_4_] exhibited superior capability with a depolymerization yield of 92% in breaking β-O-4′ linkages in lignin. Furthermore, numerous studies indicate that imidazolium-based ILs with [HSO_4_]^‑^ demonstrate high depolymerization activity, likely due to the strong resonance of negative charges at double-bonded oxygen atoms in [HSO_4_]^‑^.^917^ Singh and Dhepe anchored [(HO_3_S)­C_3_C_1_im]­[HSO_4_] onto a silica framework (loaded at 42.2 wt%) for the decomposition of macromolecular lignin (60,000 Dalton), achieving >90% yield of low molecular weight compounds.^918^ In ILs with the same anion, various cations also exhibit different catalytic effects on lignin depolymerization. Singh and colleagues investigated the performance of various ILs with different cations (imidazolium, benzimidazolium, ammonium, and phosphonium) for depolymerizing lignin into micromolecular aromatic compositions. Their results indicated that ILs with an imidazolium cation structure exhibited strong interaction with the substrate.^469^

One notable characteristic of lignin degradation is its mild operating temperature range (200–500 °C), although repolymerization and nondirectional conversion often occur during the depolymerization process. Hydrogenation effectively mitigates repolymerization.^469^ Hydrodeoxygenation (HDO) of lignin represents a significant method for its efficient, high-value utilization.^919,920^ A typical HDO process involves (i) metal catalysts for hydrogenation and (ii) acidic materials with sufficient acidity for dehydration.^921,524^ Metal catalysts such as Pd, Pt, Rh, and Ru are commonly used in ILs due to their effective catalytic performance in breaking C–O bonds in the presence of H_2_.^922^

##### 2.11.2.1 Focused Catalysis of Lignin

Lignin contains several linkages, among which the β-O-4 aryl–alkyl ether linkage is dominant. Various catalysis namely, acidic, alkaline, and oxidative biocatalysis, provides an array of opportunities for depolymerizing lignin. From the very beginning of identification of ILs, they have been suitable candidates for their application in catalysis due to their unique and highly tunable polarity/nucleophilicity properties.^19^ A majority of the catalysis applications of ILs are based on the two main concepts: (a) IL biphasic systems or (b) IL thin film systems.^344^ Table 7 enlists a few key lignin depolymerization studies using ILs/DESs.

**Table 7:** ILs/DESs Catalyzed Lignin Depolymerization

substrate	IL/DES	additives/ solvent	temp (°C)	product (% yield)	ref
guaiacylglycerol-β-guaiacyl ether, veratrylglycerol-β-guaiacyl ether	[C_4_C_1_C_1_im]Cl	1,5,7-triazabicyclo[4.4.0]dec-5-ene	150	β-O-4 ether bond cleavage (40%)	^923^
guaiacylglycerol-β-guaiacyl ether, veratrylglycerol-β-guaiacyl ether	[C_1_im]Cl	1,5,7-triazabicyclo[4.4.0]dec-5-ene	150	guaiacol (70%)	^924^
eugenol	[C_2_C_1_im][CF_3_SO_3_]		200	2-methoxyphenol (11.6%)	^925^
guaiacol	[C_1_im]Cl	microwave		catechol (81%)	^926^
*p*-benzyloxy phenol	[(OH_3_S)^4^C_4_C_1_im][CF_3_SO_3_]	electrochemical		benzyl alcohol (>80%)	^927^
2-phenoxyacetophenone	[Ch]Cl:EG	electrochemical		fucitol, *p*-coumaryl alcohol, bisphenol-A	^928^
2-phenoxy-1-phenylethanol	[Ch]Cl:pTSA		120	phenol, 2-phenylacetaldehyde	^929^

alkali lignin	[C_2_C_1_im][C_1_CO_2_]	water	100	guaiacol, phenol, vanillin	^930^
	[C_3_C_3_C_2_N][C_7_CO_2_]		170	mixture of guaiacols	^931^
	[(OH_3_S)^3^C_3_C_1_im] [HSO_4_]		100	guaiacol	^932^
	[(OH_3_S)^3^C_3_C_1_im][CF_3_SO_3_]		80	benzoic acid, phenol	^933^
	[C_4_C_1_im][CF_3_SO_3_]	UV (100 mW cm^–1^)	50	phenol, benzaldehyde	^934^
	[C_2_C_2_C_2_N][HSO_4_]	electrochemical		aromatic acids	^935^
	[C_4_im]_3_[PMo_12_O_40_]	water	80	vanillin	^936^
	[C_4_im][FeCl_4_]	water	rt	vanillin	^937^
	[Ch]Cl:pTSA		130	mixture of guaiacols	^938^
	[Ch]Cl:C_1_OH	Cu(C_1_CO_2_)_2_	60	acetovanillone	^939^

dealkaline lignin	[(OH_3_S)^3^C_3_C_1_im] [HSO_4_]		120	1,4-dimethoxybenzene, guaiacol	^940^
	[C_2_C_2_C_2_N][HSO_4_]	electrochemical		vanillin	^941^

organosolv lignin	[(OH)^2^C_2_N][CO_2_]	TEMPO/Cu(C_1_CO_2_)_2_	110	oxidized aromatics	^942^
	[(OH)^2^C_2_N][CO_2_]	MnO_2_	110	syringaldehyde	^943^
	[C_2_C_1_im][HSO_4_][C_2_C_2_PO_4_]		160	mixture of guaiacols (3.5%)	^944^

Kraft lignin	[C_2_C_1_im][C_1_CO_2_]	CoCl_2_	120	guaiacol, syringol, vanillin	^945^
	[C_2_C_1_im][C_2_SO_4_]	ABTS		nd	^946^
	[Ch]Cl:OA	H_2_SO_4_	80	vanillic acid	^947^

lignocellulose	[C_2_C_1_im][HSO_4_]	K_10_P_2_W_17_O_61_	100	aromatic acids	^948^

2.11.2.1.1. *Catalytic Conversion of Lignin with Acidic ILs*. Among acidic IL catalysis, both Brønsted and Lewis acid ILs have been studied for lignin depolymerization. For instance, Binder *et al*. explored reactions of lignin model compounds in ILs using Brønsted acidic and Lewis acidic catalysts at elevated temperatures (below 200 °C).^925^ The study focused on two key aspects of catalysis: (i) solubilization of reaction components and (ii) interaction between solvent and solutes driving solute reactivity. The efficiency of these reactions strongly correlated with the choice of ILs. ILs with moderately basic anions such as Cl^–^, Br^–^, [C_1_CO_2_]^−^ and [CF_3_CO_2_]^−^ inhibited dealkylation reactions, while ILs with very weakly basic anions including [BF_4_]^−^, [PF_6_]^−^, [CF_3_CO_2_]^−^ and [(CF_3_SO_2_)_2_N]^−^ favored such reactions.^925^ Up to an 11.6% molar yield of the dealkylation product, 2-methoxyphenol, derived from the model compound 2-methoxy-4-(2-propenyl) phenol and pre-cleaved 2-phenylethyl phenyl ether was demonstrated under optimized conditions. However, these acid catalysts were ineffective in the dealkylation of saturated-chain containing model compounds (4-ethyl-2-methoxyphenol) and in the depolymerization of technical lignin such as OrganoSolv lignin.

Cox *et al*. employed ILs based on 1-methylimidazolium cation with Cl^–^, Br^–^, [HSO_4_]^−^ and [BF_4_]^−^ anions, along with [C_4_C_1_im]­[HSO_4_], to degrade two lignin model compounds, guaiacylglycerol-β-guaiacyl ether (GG) and veratrylglycerol-β-guaiacyl ether (VG).^515^ They evaluated IL acidity using 3-nitroaniline as an indicator to measure H_0_. The ILs ranked in acidity as follows: ([C_1_im]­[BF_4_]) > [C_1_im]­[HSO_4_] > 1-methylimidazolium bromide ([C_1_im]­Br) > [C_4_C_1_im]­[HSO_4_]. Interestingly, relative acidity did not correlate with ILs' ability to catalyze the hydrolysis of β-O-4 ether bonds. Guaiacol recovery from each IL followed this order: [C_1_im]Cl > [C_4_C_1_im]­[HSO_4_] > [C_1_im]Br > [C_1_im]­[HSO_4_] > [C_1_im]­[BF_4_]. The reactivity of the model compounds in these ILs was dependent on both acidity and ion nature, and their interactions with the model compounds. GG, characterized by predominant inter-unit lignin linkages, converted into a glycerol-type enol ether (EE), 3-(4-hydroxy-3-methoxyphenyl)-2-(2-methoxyphenoxy)-2-propenol when heated in IL at 120 °C.^923^ EE was the primary product across all ILs used, although the rate and secondary decomposition products of GG varied with the specific ILs employed. A catalytic mechanism inspired by the effective performance of [C_1_im]Cl as both solvent and catalyst in hydrolyzing common β-O-4 linkages in lignin, extending this system to other lignin model compounds was proposed. [C_1_im]Cl enhanced the selectivity of lignin model compound hydrolysis to desirable chemicals, facilitated product separation, and allowed catalyst and solvent reuse. Furthermore, this catalytic system was extended to convert isolated lignin obtained from oak wood dissolution in [C_2_C_1_im]­[C_1_CO_2_] and subsequent precipitation.^924^ The depolymerization proceeded under mild conditions (110–150 °C) via a hydrolysis reaction that cleaved alkyl–aryl ether linkages, consistent with previous reports on acid-catalyzed lignin depolymerization. Given the acidity of the protic [C_1_im]­Cl, acid-catalyzed dehydration and coupling would be expected first to explain the hydrolysis of β-O-4 linkages in the absence of water. Water initiates attack on the β-carbon atoms of the proposed intermediates, leading to the cleavage of β-O-4 bonds. The application of [C_1_im]Cl not only amplifies the specificity of lignin model compound hydrolysis into desired chemicals but also streamlines the separation and reuse of catalysts and solvents.^949^ Additionally, this catalytic framework was extended to transform isolated lignin obtained by dissolving oak wood in [C_2_C_1_im]­[C_1_CO_2_] followed by precipitation.^950^ The findings illustrated that at mild temperatures (110–150 °C), lignin depolymerization occurred employing the acidic IL [C_1_im]Cl as both solvent and catalyst, involving hydrolysis to dismantle alkyl–aryl ether linkages. These results correspond with existing literature on acid-driven lignin depolymerization in conventional solvents and recent investigations involving GG and VG model compounds within the same IL types.

PILs demonstrated excellent performance in catalytically cleaving β-O-4 ether linkages in lignin superstructures. Hallett *et al*. explored ILs based on [C_4_C_1_im]­[HSO_4_] with varying acid and water concentrations across various lignin model compounds with different functionalities. They demonstrated correlations between H_0_, IL hydrogen bonding networks, IL cation structures and substrate reactivity. Hydrogen bonding in PILs significantly influenced anion–cation interactions, altering protonated starting material solvation and overall reaction rates. Increased water content was observed to decrease the cleavage rate of β-O-4 ether, despite its necessity for this reaction.^344^

In addition to Brønsted acids, Lewis acids have been utilized as catalysts in IL media for lignin model compound decomposition. Jia *et al*. reported on the hydrolytic cleavage of β-O-4 ether bonds in lignin model compounds GG and VG using [C_4_C_1_im]Cl with metal chlorides and water as catalysts. FeCl_3_, CuCl_2_ and AlCl_3_ were effective in catalyzing the cleavage of GG’s β-O-4 bonds, with AlCl_3_ outperforming FeCl_3_ and CuCl_2_, in cleaving VG’s β-O-4 bonds. GG achieved complete conversion with approximately 70% β-O-4 bond hydrolysis after 120 min at 150 °C in the presence of FeCl_3_ and CuCl_2_. With AlCl_3_, about 80% of GG's β-O-4 bonds were hydrolyzed with complete conversion. VG's β-O-4 bonds were 75% hydrolyzed with AlCl_3_ after over 240 minutes at 150 °C.^951^ Jie Chang *et al*. described a one-pot conversion of lignin and sugars using metal chlorides in [C_4_C_1_im]­Cl.^952^ Lignin and sugars from polysaccharide hydrolysis dissolved in IL were catalyzed by CrCl_3_ or CrCl_3_·6H_2_O to form insoluble products. Effective results were obtained with CrCl_3_·6H_2_O at 170 °C, achieving nearly complete removal of lignin and sugars from IL, with recyclable IL and catalysts maintaining their activity.

2.11.2.1.2. *Catalytic Conversion of Lignin by Alkaline ILs*. Besides the acidic catalysts, a series of organic bases with various basicities and structures have also been employed to investigate the cleavage of the β-O-4 bond in a lignin model compound GG with [C_4_C_1_C_1_im]Cl being the IL media. The results showed that 1,5,7-triazabicyclo[4.4.0]­dec-5-ene was the most active N-base among all the tested ones, resulting in a cleavage of more than 40% of the β-O-4 ether bonds. This implies that the higher activity might be associated with the accessibility of the N-atoms.

##### 2.11.2.2 Oxidative Depolymerization

Oxidative degradation and modification of lignin represent crucial strategies for its valorization and excellent IL focused reviews have been published.^949,953,78^ De Gregorio *et al*. provided a comprehensive review of this area, focusing on the use of ILs. They explored the potential of oxidative conversion by dissolving lignin model compounds, lignin and lignocellulosic composites in ILs. Alcell and soda lignin were dissolved in [C_2_C_1_im]­[(O)^2^C_2_)_2_PO_2_] and subsequently oxidized using various transition metal catalysts with molecular oxygen under mild conditions. Among these, CoCl_2_ in [C_2_C_1_im]­[(O)^2^C_2_)_2_PO_2_] proved highly effective for oxidation. This catalyst selectively oxidized benzyl and aliphatic hydroxyl groups in lignin, while leaving phenols, 5–5, β-O-4, and phenylcoumaran linkages intact. The γ-hydroxyl groups of cinnamyl alcohol were converted to cinnamaldehyde or cinnamic acid, and double bonds were oxidized to benzoic acids or epoxides. Phenolic hydroxyl groups in guaiacol, syringol and vanillyl alcohol remained unaffected, whereas the benzyl hydroxyl group of vanillyl alcohol was oxidized to form vanillin. This system holds promise for enhancing lignin's oxygen functionality prior to depolymerization or introducing additional functionalization post-depolymerization.

Wasserscheid *et al*. reported on oxidative depolymerization of lignin using ILs such as Fe­(III) (Fe_2_(SO_4_)_3_, FeCl_3_), Cu­(II) (CuSO_4_, CuCl_2_) and Mn­(II) (MnSO_4_, MnCl_2_, Mn­(NO_3_)_2_) as catalysts.^954^ The efficiency and selectivity of these conversions depended on both the ILs and the metal catalysts used. The highest lignin conversion rates were achieved in systems involving 1-ethyl-3-methylimidazolium triflate ([C_2_C_1_im]­[CF_3_SO_3_])/Mn­(NO_3_)_2_. With catalyst loadings of 2 wt% and 20 wt%, more than 63% of the lignin was converted, yielding 2,6-dimethoxy-1,4-benzoquinone as the primary product at higher catalyst loadings. The isolated yield of this compound was 11.5 wt% with a selectivity of 21.0%, based on the initial lignin input.

Han *et al*. introduced a novel approach using the IL (1-benzyl-3-methylimidazolium bis­(trifluoromethylsulfonyl)-imide, [BnC_1_im]­[(CF_3_SO_2_)_2_N]), to generate OOH free radicals during lignin transformation. They successfully produced benzoic acid and phenol from the lignin model compound 2-phenoxyacetophenone under metal-free conditions using O_2_ as the oxidant with catalytic H_3_PO_4_.^955^ This IL-based metal-free catalytic system effectively depolymerized various lignin model compounds and OrganoSolv lignin, facilitating lignin depolymerization via a redistribution mechanism with phenols in ILs. Two ILs ([C_2_C_1_im]­[C_1_C_1_bzSO_3_] and [C_4_C_1_im]­[C_1_SO_4_]) were identified as effective solvents for oxidative lignin depolymerization. These ILs enabled extensive depolymerization of both OrganoSolv and Klason lignins under oxidative conditions using a Cu/EDTA complex in the presence of a monomeric phenol, 4-*tert*-butyl-2,6-dimethylphenol, to prevent oxidative coupling at ortho and para positions, albeit resulting in depolymerized lignin with relatively high average molecular weights (1.2–2.0 × 103 Da).^956^ Further treatments were recommended to obtain lower molecular weight lignin-based chemicals.

Zhu *et al*. investigated the use of palladium nanoparticles dispersed in ILs for oxidizing lignin model compounds and lignin itself. They found that substituted benzyl alcohols were converted to aromatic aldehydes using O_2_ as the oxidant. Under optimized conditions, they achieved 72% lignin conversion, yielding mainly syringaldehyde, vanillin, *p*-hydroxybenzaldehyde, along with a minor amount of 2,6-dimethoxy-1,4-benzoquinone.^957^ A combined reaction separation process was developed for oxidative lignin degradation to aromatic aldehydes in ILs. CuSO_4_ catalyzed the process in [C_1_C_1_im]­[(C_1_O)_2_PO_2_], achieving complete lignin conversion with a total aromatic aldehyde yield of 29.7%.^958^ ILs containing aromatic rings in their cations demonstrated superior performance in oxidative lignin degradation, potentially due to improved solubility facilitating exposure of phenylpropane connecting points in lignin's heterogeneous three-dimensional matrix, thereby accelerating its degradation. This coupled process minimized over-oxidation of products, enhancing lignin conversion and aromatic aldehyde yield.

Reichert *et al*. exploited the electrochemical stability and solubility of specific PILs for alkaline lignin cleavage in triethylammonium methanesulfonate ([C_2_C_2_C_2_N]­[C_1_SO_3_]).^959^ Electrolysis at potentials ranging from 1.0 V to 1.5 V (vs an Ag pseudo-reference electrode) yielded a diverse array of aromatic fragment products identified by GC-MS and HPLC. This milder conversion process for lignin compared favorably to conventional chemical catalysis, although its efficiency requires further refinement. Prado *et al*. utilized ILs such as [C_4_im]­[HSO_4_] and triethylammonium hydrogen sulfate ([C_2_C_2_C_2_N]­[HSO_4_]) for *Miscanthus giganteus* delignification and subsequent lignin depolymerization using H_2_O_2_.^960^ They observed that lignin derived from [C_4_im]­[HSO_4_] was more susceptible to degradation, yielding aromatic acids such as benzoic acid, vanillic acid, and benzene dicarboxylic acid in their oils. Finally, vanadium-based polyoxometalate with non-toxic ILs like [C_4_im]­[HSO_4_] proved effective for lignin valorization under oxygen-rich conditions, yielding a spectrum of phenols and functionalized aromatics including vanillin and syringaldehyde.^474^ The yield and distribution of aldehyde products correlated with the original lignin species. Yang *et al*. demonstrated that the IL 1-octyl-3-methylimidazolium acetate ([C_8_C_1_im]­[C_1_CO_2_]) as a solvent promoted the aerobic oxidation of lignin model compound 2-phenoxyacetophenone under metal-free conditions, yielding phenol and benzoic acid with high yields of 96% and 86%, respectively.^961^ Control experiments highlighted the role of the basic acetate anion in inducing C–O bond cleavage of the aromatic ether, extending the applicability to typical lignin model compounds like 2-(2-methoxyphenoxy)-1-phenylethanone.

#### 2.11.3 Biocatalytic Depolymerization of Lignin in ILs and DESs

In the field of biocatalytic conversion of lignin, biocompatible ILs and DESs have been investigated for lignin-degrading enzymes such as laccase, alcohol oxidase, and lignin peroxidase.^962–965^ Minimal loss of biocatalytic activity of laccase was observed in a diethylamine-based IL diethylammonium hydrogensulfate ([C_2_C_2_N]­[HSO_4_]), whereas [Ch]­[Lys] and 1-ethyl-3-methylimidazolium acetate ([C_2_C_1_im]­[C_1_CO_2_]) had an inhibitory effect on the laccase enzyme. The oxidative cleavage of β-O-4 linkages resulted in aromatic aldehydes and ketones such as vanillin, syringaldehyde, acetosyringone and acetovanillone, which were extracted using ethyl acetate.

These observations allow the conclusion that the catalytic performance of enzymes in ILs is mainly influenced by the interactions of anions with the protein structure, resulting in enzyme activation when the protein structure is minimally changed and/or preserved and in protein denaturation and enzymatic deactivation when the structure is highly modified. However, the IL-enzymatic deactivation is supposed to be reversible and the recovery of enzymatic activity when the enzyme was dissolved in water was already observed.

The residual activity of laccase from *T. versicolor* in sodium acetate buffer and IL solutions was measured over 7 days, reporting that the enzyme half-life was more than a month in 15% (v/v) [C_2_C_1_im]­[C_2_SO_4_] solution, 9.8 days in 15% (v/v) [C_2_C_1_im]­[C_1_CO_2_] solution, and 2.4 days in a 0.1 M sodium acetate buffer with pH 4.5.^966^ The same study also evaluated the viscosity and the conductivity of the ILs solutions, concluding that these parameters were key properties to explain the faster decrease of laccase activity in acetate buffer. In low concentrations of ILs, the conductivity of the solution improved while the viscosity remained unchanged, which improved the enzyme stability by retaining water molecules on its structure.

Another study observed a reduction of 26% in the laccase activity in the presence of 10% (v/v) [C_2_C_1_im]­[C_2_SO_4_] over 7 days and a decrease of 35% in the activity in 0.05 mM citrate/0.1 mM phosphate buffer pH.^967^ According to the authors, the improvements in laccase stability were justified by higher water retention in the structure of the enzyme due to IL hydrophilic and water-miscible characteristics. In addition, the activity and stability of laccase in aqueous solutions of three different ILs were investigated, showing that [C_4_C_1_im]­[CF_3_SO_3_] and 1-butyl-1-methylpyrrolidinium triflate ([C_4_C_1_pyrr]­[CF_3_SO_3_]) destabilized the enzyme.^968^ After 5 days of incubation, these ILs at concentrations of 1 M, were responsible for maintaining only 20% of the enzyme initial activity, while without ILs the final activity corresponded to 38%. On the other hand, the IL tetramethylammonium triflate ([C_1_C_1_C_1_C_1_N]­[CF_3_SO_3_]) at 1 M greatly enhanced the stability of laccase, with residual activity of 95%. The effects of the ILs on laccase were supposed to be associated with the kosmotropicity and chaotropicity of the cations in Hofmeister series and were correlated with conformation changes in the enzyme structure, affecting its internal electron transfer rate, its substrate affinity, and its water association. These results demonstrate the capacity of ILs as solvents for improving the laccases stability and the importance of protein–water interactions as a key factor for the maintenance of laccases activity in ILs solutions. As for the enzymatic activity, changes in the enzymatic stability seem strictly related to the modification of protein conformation through ion interactions with protein-charged groups, resulting in less or more exposition of enzyme active sites. Particularly, when the ion–protein interaction is strong, there is a tendency towards destabilization, while interactions with the enzyme surrounding water tends to stabilize the enzyme.

Although the studies described above demonstrate substantial progress, there are still avenues for further research in lignin biocatalysis when using biocompatible ILs and DESs. Achieving efficient and selective lignin depolymerization in these solvents requires a delicate balance of factors such as lignin solubility, enzyme activity, and specificity and substrate accessibility. The complex composition and structure of lignin make it a challenging substrate for enzymatic catalysis. Furthermore, while ILs and DESs, particularly aqueous forms, show potential for enzymatic lignin depolymerization and fractionation, concerns remain about the compatibility and interactions between lignin-degrading enzymes and these solvents. The possibility of enzyme denaturation or destabilization by solvents is a significant issue in integrated processes. Additionally, lignin repolymerization or condensation post enzymatic depolymerization presents a primary challenge. Finally, beyond the solvent effects, byproducts generated during fractionation processes may also impact the catalytic activity of lignin-degrading enzymes.

Considering biocatalysis of lignin as a critical key to accelerating IL- and DES-based biorefinery deployment, we recommend future directions that may mitigate the challenges discussed above. For instance, generating water soluble lignin fractions suitable for enzymatic degradation using aqueous biocompatible ILs/DESs is critical. Previous studies have shown that such aqueous ILs/DESs can lower solvent costs and viscosity.^175,700,969^ To enable this, efficient pretreatment methods that not only enable maximum lignin removal but also minimize inhibitor formation are essential to promote integrated processing. Additionally, a major concern with enzymatic lignin depolymerization is the repolymerization of the degradation products. Consider combining the lignin-degrading enzymes and microorganisms, where the microorganisms can convert depolymerized lignin molecules into products preventing unwanted repolymerization.^78,970^ Furthermore, we recommend diversification of host organisms to reduce fermentation and medium costs, while boosting protein yield and productivity and elevating the activity and purity of lignin-degrading enzymes. This can be achieved by continued exploration and enhancing performances of enzymes through modification or better/newer design.^971^

#### 2.11.4 Pyrolysis Oil

Due to the recalcitrance of lignin, a thermochemical conversion method like pyrolysis, capable of breaking down lignin into smaller phenolic compounds, has been demonstrated as an effective approach for valorizing lignins obtained from different biomass pretreatment processes.^972–975^ Substantial research has focused on enhancing the pyrolysis oil yield by modifying the resulting lignins efficiently.^976–979^ Hence, it is intriguing to examine the potential contributions that ILs and DESs might offer in this context.

Lei *et al.* found that IL pretreatments at different temperatures (from 20 to 150 °C) significantly affect the distribution of subsequent lignin pyrolysis products. In their study, lignin recovered from pretreatment at 50 °C showed the highest phenolic yield.^980^ According to Li *et al.*, acidic DESs can also depolymerize lignin, increasing the thermal stability as more pyrolysis products came with less side chains on the aromatic rings.^981^ Further work by Li *et al.* studied the effect of the DES-regulated lignin (the DES including [Ch]­Cl/ethylene glycol, ZnCl_2_/ethylene glycol and [Ch]­Cl/acetic acid) on the subsequent pyrolysis product selectivity.^982^ Their findings not only confirmed that the DESs are conducive to reducing the molecular weight (MW) of lignin, but also more importantly demonstrated that such pretreatment is beneficial to increment of yield of pyrolysis oil and the selectivity of the monomer aromatic hydrocarbons.

Moreover, ILs have also been used to extract the various components from lignin pyrolysis oil.^983^ Three phosphonium ILs namely trihexyltetradecylphosphonium chloride ([C_6_C_6_C_6_C_14_P]­Cl), trihexyltetradecylphosphonium dicyanamide ([C_6_C_6_C_6_C_14_P]­[(CN)_2_N]), and trihexyltetradecylphosphonium bis-2,4,4-(trimethylpentyl) phosphinate ([C_6_C_6_C_6_C_14_P]­[(^4^C_1_^4^C_1_^2^C_1_)­C_5_)_2_PO_2_]), had shown high affinity for acetic acid and glycoladehyde during the liquid–liquid extraction.^984^ The reusability of the ILs was also estimated and the regenerated IL exhibited similar extraction performance as the fresh one. Additionally, the IL [C_4_C_1_im]­[(CF_3_SO_2_)_2_N] served as a great co-solvent to extract phenol from pyrolysis oil.^985^ Hou *et al.* also found similar extraction behavior with their imidazolium based ILs including [C_4_C_1_im]­[BF_4_], 1-butyl-3-methylimidazolium hexafluorophosphate ([C_4_C_1_im]­[PF_6_]), [C_4_C_1_im]­Cl, and [C_4_C_1_im]­Br.^986^ According to their systematic study, the anions of the imidazolium-based ILs had a significant impact on the phenol extraction efficiency, which follows the order: Cl^–^ > Br^‑^ > [BF_4_]^−^ > [PF_6_]^−^.

#### 2.11.5 Carbon Fibers

Carbon fibers exhibit high tensile strength and tensile modulus because of the unique fiber-oriented turbostratic or graphite-like carbonaceous crystal structure. Utilizing lignin as a precursor material for carbon fibers synthesis is not a new research topic. Thanks to the low cost, renewability and high carbon content of lignin, extensive research for its conversion to carbon fibers has been carried out since the end of the twentieth century.^987^ From an economic viewpoint, the higher carbon yield and the aromatic character make lignin a great candidate for carbon fiber synthesis as compared to many commercialized carbon fiber streamlines, such as polyacrylonitrile based fibers, with reported low carbon yield.^988^ However, the complex and disordered structure of lignin may lead to crosslinking and the formation of more pores in the resulting carbon fibers.^989^ The mechanical performance of the fibers is believed to be governed by the pore dimensions and their misorientation with the main axis of the fiber.^990,991^ In general, lignin-based carbon fibers show low mechanical performance, that is, low tensile strength <1GPa, low tensile modulus <150 GPa, and low elongation at break <1%.^992^ In addition, the high content of hydroxyl groups increases the agglomeration tendency of lignin fractions, leading to leaching phenomena in the coagulation bath in wet-spinning processes with other polymers.^993^ Another crucial drawback of lignin serving as the carbon fiber precursor derives from its low thermal stability, owing to the fact that the randomly constituted amorphous lignin oligomers inevitably lead to an undefined pore structure in the subsequent carbon fibers.^992,994^

Efforts have been made to focus on the impact of ILs on the improvement of the thermal properties of the lignin as carbon fiber precursors. Aiti *et al.* reported the use of the IL [C_2_C_1_im]Cl in mixing lignin and textile grade polyacrylonitrile for carbon fiber synthesis. They found that the IL acted as a “lubricating agent” in the fiber structure to orienting the fibers via the extensional forces being applied in the coagulation zone.^988^ This is similar to the finding of Brandt *et al.* of ILs acting as lubricants and orienting the fibers of pine wood during the grinding of IL-soaked wood chips.^995^ The TGA of the carbon fibers regenerated by Aiti *et al.* indicated that lignin-containing fibers exhibited better thermal stability than the neat polyacrylonitrile fibers. In terms of the improvements in tensile strength, there had not been too many works reported yet, especially regarding the role that ILs could play in the process. A noticeable strength enhancement was achieved by incorporating cellulose into the lignin as the co-precursor through the co-dissolution of both components with the IL during the mixing or fractionation stages.^771,996^ Identifying a robust and low-cost fabrication method is another challenge that needs adressing. Very recently, a mixture of the IL [C_4_C_1_C_1_N]­[HSO_4_] and water was shown to be an effective solvent for the continuous wet-spinning of fibers with high lignin content.^997^ As indicated in Figure 56, the precursor fibers had high lignin content (75–90%). After the carbonization at 1000 °C, the resulting carbon fibers had tensile strengths and moduli of up to 450 MPa and 40 GPa, respectively.

**Figure 56:**
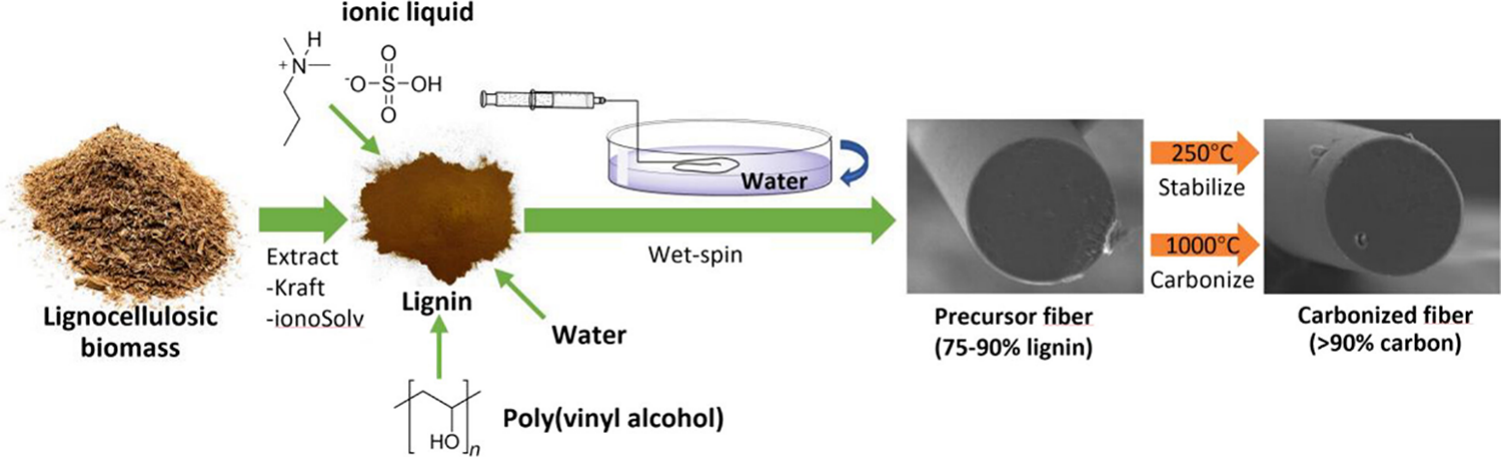
Workflow for spinning and conversion of high lignin content fibers using the IL [C_4_C_1_C_1_N]­[HSO_4_]. Adapted with permission from ref 997. Copyright 2023 American Chemical Society under CC BY 4.0 (https://creativecommons.org/licenses/by/4.0/).

#### 2.11.6 Bioplastics from Lignin

The increasing demand for sustainable alternatives to conventional plastics has driven the exploration of renewable feedstocks for bioplastic production. The development of bioplastics (plastics that are biodegradable and made of natural materials) could provide businesses with eco-friendly alternatives for products and packaging.^998,999^ Although the bioplastic industry is still in its early stages, a steady growth can be found in starch-based, cellulose-based, protein-based, bio-derived polyethylene and aliphatic polyesters.^1000^ Lignin is also a promising option due to its availability, low cost, and unique chemical structure.^1001,76^ However, inherent challenges such as its high molecular weight, insolubility and thermal instability must be addressed to achieve desirable material properties. Current lignin-based bioplastics need improvements in their mechanical, thermal and barrier properties.^1002,1003^ Also, properties such as tensile strength, breaking elongation, tear strength, flexibility, durability, printability, transparency, barrier, heat resistance and biodegradability of lignin-based materials should be well evaluated.^1004,1005^ Recent advances aim to overcome challenges in processing techniques, including blending, crosslinking and nanocomposite formation.^1006,1007^

##### 2.11.6.1 Lignin Extraction and Modification

Using ILs for biomass pretreatment enables easier access to the biomass polysaccharides. Extensive fundamental studies in the dissolution of cellulose or lignin have been conducted in the literature. Research on lignin dissolution in ILs initially focused on imidazolium-based ILs.^1008^ The Ragauskas group demonstrated that the [C_4_C_1_im]^+^ ILs can be used as aprotic solvents for lignin, and concluded that the solubility of lignin was influenced by the nature of anions in the ILs.^1008^ In 2014, Glas *et al.* studied a series of non-imidazolium ILs including ammonium, phosphonium, and pyrrolidinium based for lignin dissolution and concluded that the tributylmethylphosphonium methyl sulfate [C_4_C_4_C_4_C_1_P]­[C_1_SO_4_] displayed the highest Kraft lignin dissolution (460 g/kg) at 90 °C.^1009^ The work indicated that the IL could be recycled without loss in lignin dissolving ability. Similar recyclability for some imidazolium type, *i.e.*, [C_2_C_1_im] based, was also proven by Saha *et al.* and a maximum lignin yield up to 90.1% was achieved.^1010^ Another case study with food additive derived ILs also indicated the great efficiency in separating lignin from biomass.^1011^

As discussed earlier, the use of ILs to extract lignin from biomass results in structural alteration of lignin after its dissolution. This may potentially offer the opportunity to enhance its properties for bioplastic synthesis. As evidenced by work from An *et al.*, the β-O-4 linkage was broken during the Kraft lignin dissolution in [Ch] ILs including [Ch]­[C_1_CO_2_], [Ch]­[Glc], [Ch]­[Gly], [Ch]­[Lys], and [Ch] arginate ([Ch]­[Arg]), while the β–β′ and β-5′ linkages were formed.^516^ With the increased C–C bond interlinked in the lignin, a potential increase in strength properties of the resulting bio-composite is highly possible. Compared with the conventional Kraft process, the lignin from IL treatment tends to have larger molar mass and a more uniform molar mass distribution.^1011^ This could also potentially be a positive sign in generating promising bioplastics. A recent trend in lignin structure alteration during the isolation with ILs seeks to modify the hydroxyl group on the side chain of lignin units.^1012^ Potential benefits include the change of hydrophobicity of the isolated lignin and effect on the subsequent esterification capability in hybrid copolymer coupling steps.

##### 2.11.6.2 Lignin-Based Bioplastics Properties and Processing

Lignin possesses unique structural features that contribute to its potential as a bioplastic feedstock, including its aromatic nature, abundant hydroxyl groups, and polymeric structure.^75^ The hydroxyl groups, one of the most characteristic functional groups in lignin, constitute the reactive sites that can be exploited in polymer chemistry. It has been suggested that the basicity of the anion in ILs favors the weakening of the hydrogen bonding networks in lignin, potentially freeing hydroxyl groups in the lignin matrix.^1013^ Great interest had therefore been placed in ILs which may also enable the biopolymer to be obtained selectively. Renneckar group proposed a route to convert technical lignin into versatile lignin esters for tailored bioplastics.^1014^ In their work, around 90% of the aliphatic hydroxyl groups can be esterified with the conformation of the resulting polymers ranging from a rod-like structure to dense spheres that could potentially be suitable for multifunctional composite materials.

A possibility to improve plastic properties is the modification of lignin to enhance crosslinking with other co-agents or itself.^1015,1016^ One approach is to demethylate the methoxy groups on lignin to yield hydroxyl groups.^1017^ Many model compounds studies revealed that G-units can be effectively converted into C-units via demethylation.^1018,1019^ It has been proven that the catechol with two hydroxyl groups on the aromatic rings effectively enhances hardness and other properties of the resulting biopolymers.^1020,1021^ Various demethylation approaches for lignin are listed in Table 8 and the degree of demethylation rate is compared. As shown in the table, the demethylation rate increased as the approach transits from biological to chemical, and the highest rate was up to 87%. Among these chemical processes, IL has been established to play a positive role in demethylating lignin. Recently, Zhao *et al.* demonstrated an effective production of polyphenol (catechol-rich) from real lignin through a demethylation strategy under halogen-free conditions, enabled by low-cost bifunctional PILs including tri-2-hydroxyethylammonium acetate ([((HO)^2^C_2_)_3_N]­[C_1_CO_2_]), bis-2-hydroxyethylammonium acetate ([((HO)^2^C_2_)_2_N]­[C_1_CO_2_]), 2-hydroxyethylammonium iodide ([(HO)^2^C_2_N]­I), 2-hydroxyethylammonium chloride ([(HO)^2^C_2_N]­Cl), [(HO)^2^C_2_N]­[C_1_CO_2_], 2-hydroxyethylammonium lactate ([(HO)^2^C_2_N]­[(HO)^1^C_2_CO_2_]), 1,1,3,3-tetramethylguanidinium iodide ([(C_1_)_4_Gu]­I) and 1,1,3,3-tetramethylguanidinium lactate ([(C_1_)_4_Gu]­[(HO)^1^C_2_CO_2_]).^1022^ By their IL studies, the demethylation promoted by [(HO)^2^C_2_N]­[C_1_CO_2_] could reduce the methoxyl content up to 73%, increase Ph-OH group content and improve the reactivity of the lignin. Recycling of the ILs is an important aspect to examine. Thierry *et al.* had investigated the demethylation of lignin in various ILs and attempted to recover the ILs from the process, however only up to 75% of the starting IL could be recovered and reused.^1023^

**Table 8:**
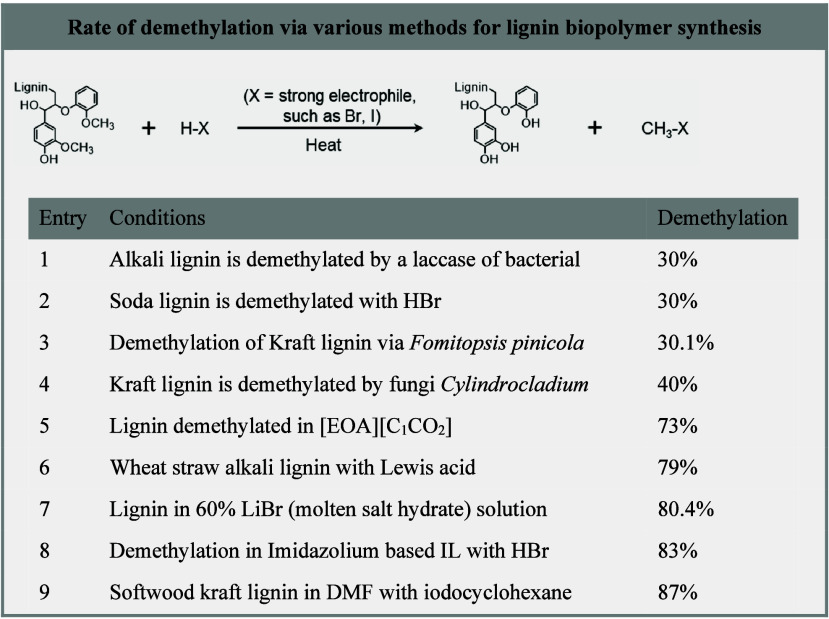
Various Demethylation Method for Lignin and the Resulting Rate^966–974^

##### 2.11.6.3 Applications and Future Prospects

Lignin-based bioplastics have demonstrated potential in a wide range of applications, including packaging materials, automotive components, and agricultural films.^1024^ Recent reviews have explored these applications and discuss the unique advantages offered by lignin, such as UV resistance, antioxidant properties, and flame retardancy.^1024–1026^ Here, we are reviewing some of the most relevant advances in the field.

#### 2.11.7 Lignin for Coating Applications

With the increased lignin fraction in many grafted lignin-based biopolymers, a decrease in the resulting biopolymer tensile strength and thermal stability is often found. However, according to Jang’s work, the surface characteristics of poly­(ε-caprolactone) grafted lignin-based polyols did not improve significantly when the lignin content increased.^1027^ This exemplifies that for biopolymers that serve for coating purposes, enhancing only the mechanical properties by the approaches aforementioned, such as demethylating lignin, is not enough to obtain the desired coating performance; and that the evaluation of the coating application for lignin-based copolymers requires performance tests more than enhancing its mechanical properties.

Esterification, oxypropylation and acetylation can also improve the coating performance of lignin. For example, esterified lignins and oxypropylated lignins normally show a great resistance to the loss of peel strength during the aging process of the resulting lignin-based coating films. Qi *et al.* synthesized an aqueous biopolymer dispersion coating system by esterifying softwood kraft lignin with long chain organic acid.^1003^ The forming lignin nanoparticles on different surfaces by either spray- or spin-coating, greatly enhance the surface hydrophobicity and roughness. Similar performance improvements have been found in oxypropylation and acetylation works.^1028,1029^ In addition, chemical derivatization that yields water-soluble lignin with anionic carboxylate groups gives lignin polyanionic behaviour and enables its utilization in the growth of UV-protective films.^1030^

ILs often function as an organocatalyst for specific lignin modification, such as esterification, oxypropylation and acetylation. Evidenced by the work from Kakuchi *et al.*, a rapid direct transesterification of cellulose with isopropenyl acetate in IL [C_2_C_1_im]­[C_1_CO_2_] was found under mild conditions.^1031^ The IL was proved to be effective in protecting the unsaturated C=C bonds during the reactions. The finding could certify the validation of using similar IL on modification of lignin to obtain the esterification selectively. Husson *et al.* described a chemical esterification of industrial lignins with maleic anhydride in an acidic IL, [C_4_C_1_im]­[HSO_4_], without an additional catalyst.^1032^ Their work also indicates the catalytic regioselectivity of the IL during the lignin esterification process.

Lignin potentially serves as an ideal material for antiviral surfaces due to its sustainability and biocompatibility. The antiviral mechanism is attributed to the local generation of reactive oxygen species upon exposure of the coating to light. Oxygen radicals induced on the surface can cause oxidative disruption of viruses.^1033^ Very recently, Boarino *et al.* reported a lignin-based film prepared by the spin-coating approach, and the resulting coating polymer showed durable antiviral activities toward HSV-2 similar to those of silver-based antiviral materials.^1034^ The key to enhancing the antiviral activity is to obtain a high concentration of phenols (playing a central role in reactive oxygen species generation) on the lignin surface. This leads to a greater chance for processing and modifying lignin in ILs. As suggested, aggregation of lignin structures is the result of the interaction of amphiphilic water-soluble lignin fraction and the surrounding solvents, leading to their specifically ordered mutual arrangement to form the lowest free surface energy.^1035^ A proper tuning of the desired IL or pretreatment conditions could impact the lignin aggregation and potentially form specific phenol-rich facets.

##### 2.11.7.1 Adhesives and Binders

Lignin contains many phenolic hydroxyl, aliphatic hydroxyl, aldehyde and carboxyl groups that can react with aldehydes or phenol, a process that is similar to the reaction between phenol and formaldehyde to form phenolic resin adhesives.^1036^ This hint at the huge potential of lignin to partially substitute phenol or formaldehyde in the industrial phenol-formaldehyde resin (PF) synthesis. One of the advantages to preparing lignin-based formaldehyde resin (LPF) is the tuneable adhesiveness made possible by modifying lignin itself. The commonly characterized adhesive properties include the lap shear strength (dried and wet), solid content, free formaldehyde emission, free phenol content, resin pH, water resistance, viscosity, wood failure percentage, gel time and curing temperature.^1037–1040^ The major adhesive characteristics of the lignin-based resins are listed in Figure 57.

**Figure 57:**
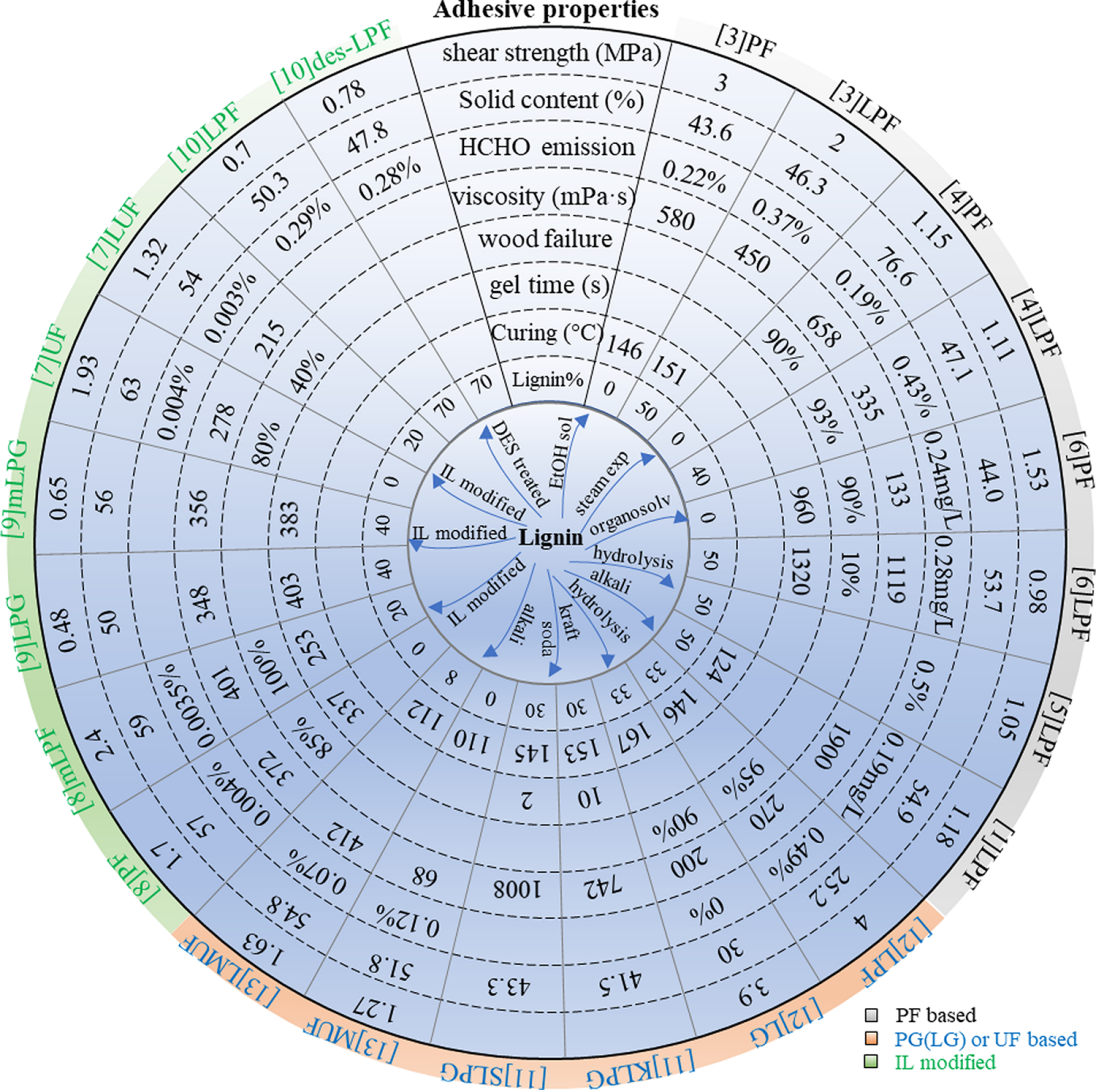
The most commonly characterized adhesive properties of lignin and IL modified lignin-based phenol-formaldehyde (PF), phenol-glyoxal (PG), lignin-glyoxal (LG), and urea-formaldehyde resins.^1037–1040,1043–1051^

ILs have shown good performance and application in the dissolution and modification of lignin as discussed in the biopolymer section. The dissolution in ILs can yield lignin with high phenolic hydroxyl content, small molecular weight and high activity due to the cleavage of aryl ether bonds.^177,1041^ IL-modified lignin has been used in preparing PF adhesive. Nevertheless, some of the challenges are to increase the lignin substitution percentage (lignin % in Figure 57) and to obtain satisfactory mechanical properties. Younesi-Kordkheili *et al.* attempted to substitute 50 wt% phenol in PF with the modified lignin and study the effect of modification methods on the properties of the resulting LPF.^1040^ They found that IL treatment had a prominent effect on lowering the formaldehyde emission and improving the mechanical strength as compared to the unmodified LPF. A parallel work done by the same group also revealed the low formaldehyde emission when they tried to replace urea with IL ([C_2_C_1_im]­[C_1_CO_2_]) modified lignin in neutral urea-formaldehyde (UF) resin.^1042^ In addition, an improvement on the water absorption by the IL modification was observed when compared to those made from unmodified lignin and commercial UF adhesives, respectively. Albeit, with respect to internal bonding performance, these results indicated even by the IL modification the resulting resins were still not comparable to commercial UF resins.

The utilization of lignin and glyoxal as the building blocks to produce phenolic resins is an emerging research field. Replacing up to 50 wt% phenol with real lignin and formaldehyde with glyoxal could result in a resin with higher tensile strength than a reference PF resin.^1052,1053^ This strategy paved a new route to potentially enhance the mechanical and thermal properties while increasing the portion of lignin in preparing the phenolic resin. More recently, a process that entirely substituted both the phenol and formaldehyde with corn stover lignin and glyoxal had been successfully developed.^1050^ The resulting lignin-glyoxal resin had a higher curing temperature and dry adhesion strength than the conventional LPF and PF resins. According to the chemical reactions revealed in the paper, it is critical to catalyze the reaction between the vacant ortho- positions to the phenolic hydroxyl group of lignin and the aldehyde. Under such context, any process that could lead to the exposure of more guaiacyl or coumaryl units from lignin will be appropriate to use to potentially enhance the resulting adhesive properties. This point should be considered when designing IL to modify lignin for the synthesis of lignin-based resins.

### 2.12 Products from Lipids and Extractives

In the past few decades, there has been an increasing demand for using naturals products instead of synthetic in industries such as cosmetics, food, nutraceuticals, animal feed and agriculture.^1054,1055^ This increment in demand has been triggered by several factors, the most relevant ones being consumers concern about potential detrimental effects of synthetic products and regulations which are promoting natural alternatives. Based on the increasing demand, academic and industrial research has focused on developing technologies to extract and purify valued-added biomolecules from natural sources.

Biomolecules are usually extracted from biomass using conventional methods based on organic solvents. Extraction processes using organic solvents exhibit several drawbacks such as low selectivity, high flammability, high toxicity and high volatility. Therefore, to overcome these drawbacks, the use of ILs as extracting solvents has been proposed because of their favorable properties, already discussed and that include low volatility, low flammability, notable solvating capacity, and high thermal and chemical stabilities.^1056,208,242^ Moreover, tunability of IL properties by using different cations and anions presents an advantage in extraction processes aiming to selectively extract some biomolecules.^1056^ To date, ILs have been used to extract alkaloids,^1057–1070^ phenolic compounds,^1065^ ^,^ ^1071–1075^ ^,^ ^847^ ^,^ ^1076–1090^ ^,^ ^373^ ^,^ ^1091^ ^,^ ^1092^ lactones,^1093–1095^ terpenoids,^1096^ ^,^ ^1097^ anthraquinones,^1098^ ^,^ ^1099^ flavonoids,^1100–1120^ saponins^847^ ^,^ ^1113^ ^,^ ^1121^ or essential oils^1073^ ^,^ ^1112^ ^,^ ^1114^ ^,^ ^1122^ ^,^ ^1123^ among other biomolecules from several bioresources. However, this section is only focused on the use of ILs to extract biomolecules from lignocellulosic biomass excluding fruits and flowers. Broader information about the use of ILs for extraction and purification of biomolecules from biomass, biomass related resources and analytical purposes can be found in reviews published by Venutra *et al.*,^1056^ Ullah *et al.* ^1124^ and Passos *et al.* ^95^

In general, most of the studies on extraction from lignocellulosic biomass focus on studying the effect of different extraction variables over the extraction yield of specific compounds or families of compounds in aqueous solutions of ILs, but the effect of using ILs as adjuvants in alcohols and alcohol/water extraction solutions has also been reported.^1064,1086,1110,1121,1125^ It is worth mentioning that only one study has reported the combined effect of ILs and enzymes in biomolecules extraction.^1078^ Extraction variables usually evaluated in literature are IL structure, IL concentration, temperature, time, solid/liquid ratio, and in some cases the particle size. It is worth noting that microwave-assisted and ultrasound-assisted extraction have been the preferred extraction methods in the literature, with the effect of power on extraction yield and other process variables frequently studied.

#### 2.12.1 Effect of the IL Structure

Most of the research performed to date has focused on evaluating the effect of the structure of aprotic ionic liquids on the recovery of specific biomolecules from the lignocellulosic biomass. In general, [C*_n_* C_1_im] cations have been studied to evaluate the effect of the cation in biomolecule extraction, with C_*n*_ usually being an alkyl chain from 2 to 10 carbons. The effect of the alkyl chain over the yield extraction depends on the biomolecule to be extracted and it is driven by the hydrophobicity of the molecule. ILs formed by the cation [C_8_C_1_im] has been shown to be the most efficient IL in the extraction of polyphenols and essential oils from *Rosmarius officinalis*,^1073^ polyphenols from *Psidium guajava Linn* leaves,^1071^ aesculin and aesculetin from *Cortex fraxini* wood,^1126^ and Tanshinones from *Salvia miltiorrhiza Bunge* root. On the other hand, the cation [C_4_C_1_im] has been the most effective to extract flavonoids from *Bauhinia championii (Benth.) Benth* leaves,^1102,1127^ proanthocyanidins from *Cinnamomum verum* J. Presl bark,^1075^ and alkaloids berberine, palmatine and jatrorrhizine from *Phellodendron amurense Rupr*.^1062^ [C_3_C_1_im] showed to be the most effective to extract ginsenosides from ginseng root.^1128^ Other functional groups instead of alkyl chain have been evaluated in imidazolium-based cations such as benzyl,^1076,1116^ aminopropyl^1064^ and ethoxyl,^1064^ showing a strong impact of the cation structure over the extraction of biomolecules.

To evaluate the effect of the anion in biomolecules extraction, anions with different structures have been evaluated using the same cation. Halogenated anions, carboxylates, [PF_6_]^−^, [(CF_3_SO_2_)_2_N]^−^, [HSO_4_]^−^ and [BF_4_]^−^ have been mainly evaluated. Halogenated anions trend to perform better than other anions when different anions are compared using the same imidazolium-based cation, with bromide exhibiting larger extraction yields than chloride.^1073,1075,1076,1083,1085,1102,1110,1128^ When halogenated anions were not evaluated, a clear trend is not observed regarding which anions display better performance. Yang *et al.* evaluated the extraction of chlorogenic acid from *Boehmeria nivea* L. leaves using aqueous solutions of [C_4_C_1_im]­[CF_3_SO_3_], [C_4_C_1_im]­[C_2_CO_2_], [C_4_C_1_im]­[C_1_CO_2_], 1-butyl-3-methylimidazolium phosphate ([C_4_C_1_im]­[H_2_PO_4_]) and [C_4_C_1_im]­[HSO_4_] in an ultrasound assisted extraction process.^1080^ Authors observed that higher extraction yields is obtained with [C_4_C_1_im]­[HSO_4_]. Ji *et al.* extracted the flavonoids glabridin, glycycoumarin, isoangustone, licoricidin and licoisoflavone from *Glycyrrhiza uralensis* roots using ultrasound-assisted extraction in aqueous solutions of 1-methyl-3-octylimidazolim hexafluorophosphate ([C_8_C_1_im]­[PF_6_]), 1-methyl-3-octylimidazolim tetrafluoroborate ([C_8_C_1_im]­[BF_4_]), 1-methyl-3-octylimidazolim hexafluoroantimonate ([C_8_C_1_im]­[SbF_6_]), 1-methyl-3-octylimidazolim diacyanamide ([C_8_C_1_im]­[N­(CN)_2_]), 1-methyl-3-octylimidazolim bis­(trifluoromethylsulfonyl)­imide ([C_8_C_1_im]­[(CF_3_SO_2_)_2_N]) and 1-methyl-3-octylimidazolim triflate ([C_8_C_1_im]­[CF_3_SO_3_]).^1115^ [C_8_C_1_im]­[BF_4_] exhibited the largest extraction yield among the studied ILs. Molecular dynamic simulations showed a strong interaction between [C_8_C_1_im]­[BF_4_] and glabridin, stronger than that of glabridin and methanol, in particular between glabridin and the [BF_4_]^–^ anion. On the other hand, bromide is not always the best alternative when imidazolium-based cations are used. For instance, Lei *et al.* studied the extraction of flavonoids from *Selaginella involven* using ILs based in [C_2_Py]^+^ dissolved in ethanol in an ultrasound-assisted system.^1115^ After screening several ILs, authors observed that *N*-ethylpyridinium tetrafluoroborate [C_2_Py]­[BF_4_] led to the highest extraction yield, even better than when using Br^−^ as the anion.

While AILs have dominated the literature, some studies have been published using PILs for biomolecules extraction. Yansheng *et al.* extracted the lactones senkyunolide I, senkyunolide H, and *Z*-ligustilide from *Ligusticum chuanxiong* *Hort* using microwave-assisted extraction with the PIL *N*,*N*-dimethyl-*N*-(2- hydroxyethoxyethyl)­ammonium propionate ([(HO)^2^C_2_OC_2_)­(C_1_)_2_N]­[C_3_CO_2_]), demonstrating that it is possible to reach high extraction yield using this PIL.^1093^ Chowdhury *et al.* extracted tannins (polyphenols) from *Acacia Catechu* using the PIL *N*,*N*-dimethylammonium *N*′,*N*′-dimethylcarbamate.^1072^ This IL is formed by mixing CO_2_ and dimethylamine in a 1:2 ratio, being distillable at 45 °C by reforming CO_2_ and dimethylamine. The authors revealed that this IL exhibits higher tannin recovery than the traditional extraction method using water. The effect of the anion over extraction yield in PILs synthesized from 1-amino-2-propanol was evaluated by Yu *et al.* ^1087^ Researchers extracted total polyphenols, flavones (class of polyphenols) and polysaccharides from *Artemisia argyi* *Lévl. et Vant* using an ultrasound-assisted extraction process. Several anions with carboxylate groups were evaluated to compare the extraction yield of the 3 biomolecules. Results show that anion structure strongly affects the extraction of the biomolecules, with the highest recoveries obtained with different anions for the 3 biomolecules. Considering that flavones were the target of this research; the highest recoveries were achieved using malate and salicylate. Interestingly, authors also show that 1-amino-2-propanol based ILs exhibit lower toxicity than 1-amino-2-propanol. Previous studies used pure IL as extraction solvent, but also aqueous solutions of PILs have been studied. Roman *et al.* evaluated the extraction of polyphenols from *Lycopodium clavatum*, *Cetraria islandica*, and *Dipsacus fullonum* using aqueous solutions of PILs based on triethanolamine and amino acids in ultrasound-assisted extraction.^1088^ ILs improve polyphenol extraction in comparison to water, especially in the case of *Cetraria islandica*, where polyphenols extraction increased more than 3 times when [C_2_C_2_C_2_N]­[Thr] or [C_2_C_2_C_2_N]­[Met] were used at 2.5% concentration. Toledo *et al.* studied the extraction of polyphenols from *Ilex Paraguarienses* leaves using aqueous and ethanol solutions of the DES [Ch]­Cl:C_1_CO_2_, the PIL [MEA]­[Ace], and the salt [Ch]­Cl, observing that the highest extraction reach was achieved using a 75% aqueous solution of [(HO)­C_1_N]­[C_1_CO_2_], reaching more than double of the extraction yield using pure ethanol–water solutions.^373^ Another interesting study was performed by Claudio *et al.*, who used surface active ILs.^1094^ They evaluated the recovery of oleanolic acid from olive tree leaves using aqueous solution of ILs constituted by [C_x_C_1_im] cations (*x*: 8–18) and several anions including halides, sulfates, phosphonates, and carboxylates. Additionally, the biodegradable IL dodecylbetainium chloride ([C_12_bet]­Cl) was studied. Results show that 1-methyl-3-tetradecylimidazolium chloride ([C_14_C_1_im]­Cl) displayed the highest extraction yield, even better than the benchmark system of methanol and ethyl acetate. [C_12_bet]Cl also outperformed methanol and ethyl acetate as extraction solvents.

#### 2.12.2 Effect of the Extraction Parameters

In order to evaluate the effect of variables on extraction yield independently of the IL structure, researchers have evaluated the effect of changing one variable at time.^1073,1101,1102,1126^ Studies varying only one variable do not enable exploring potential interaction between the parameters. Therefore, some authors have performed response surface experiment designs to explore interactions between variable, being the Box–Behnken the preferred design (refs 1062–1064 ,^1069^,^1129^,^1076^,^1077^,^1079^,^1080^,^1082^,^1083^,^1098^,^1103^,^1104^,^1106–1108,1111^ ,^1112^,^1114^,^1117^,^1119^,^1120^,^1122^,^1125^). In general, studies using response surface designs show that there is strong interaction between the extraction variables independent of the IL and raw material studied, meaning that optimum values of a variable depend on the value of the other variables. For example, optimum values for ultrasound and microwave assisted extraction has been reported for the variables power, time, solid/liquid ratio and IL concentration for extraction of alkaloids,^1062,1063^ flavonoids (mixture of polyphenols),^1077,1107,1112,1114,1119^ phenols^1077,1080^ and essential oils (mixture terpenoids).^1112,1122^ In general, studies varying one variable and response design experiments show that temperature and time exhibit positive effects over the extraction yield. On the other hand, increasing the solid-liquid ration has a negative effect, while optimum values are often reported for ionic liquid concentration.

#### 2.12.3 Purification of Biomolecules and IL Recovery

Although several studies have been published related to extraction of biomolecules using ILs from lignocellulosic biomass, there are only few studies which evaluates the selectivity of ILs during the extraction process.^1096,1105,1107,1110,1113,1121^ Selectivity is an important parameter to consider when the extraction performance is evaluated because certain degrees of purity are usually required to use the extracted biomolecules. Although extracts normally need to be further processed to increase the purity of specific molecules, it is valuable to start with extracts exhibiting as high purity as possible in order to facilitate downstream processing.

Ribeiro *et al.* evaluated the selectivity in extracting polyphenols and saponins from *Agave sisalana* and *Ziziphus joazeiro* using aqueous and ethanol–water solutions with 50% ILs.^1121^ [Ch] based ILs were evaluated using chloride and several carboxylates as anions. The authors reported higher saponin recovery when using an ethanol–water solution than water, while the selectivity of saponins over polyphenols tends to be higher for ethanol–water solutions. They also observed that the anion structure strongly affects the extraction selectivity of saponins when compared with polyphenols, and this effect depends on the raw material. Consequently, higher selectivities are observed using [Ch]­[C_1_CO_2_] for *Ziziphus joazeiro* and [Ch]Cl for *Agave sisalana*. Ma and Row extracted scoparone and the polyphenols rutin and quercetin from *Herba Artemisiae Scopariae* using 0.5 g/L of IL as adjuvant observing reducing the alkyl chain in from [C_4_C_1_im]Br to [C_2_C_1_im]Br strongly decreased the scoparone extraction without affecting rutin and quercetin extraction.^1110^ Meanwhile, increasing the alkyl chain from 1-hexyl-3-methylimidazolium bromide ([C_6_C_1_im]­Br) to 1-decyl-3-methylimidazolium bromide ([C_10_C_1_im]­Br) strongly reduced the extraction of scaparone and quercitin without affecting rutin. Ji *et al.* studied the extraction of the flavonoids liquiritin, isoliquiritin, liquiritin apioside, and isoliquiritin apioside from *Glycyrrhiza uralensis* using ultrasound-assisted extraction.^1113^ Results show that anion and cation structure display different effects toward different molecules. For instance, by comparing different alkyl chains in [C*_n_* C_1_im]Br ILs, almost 100% efficient recovery is reached for the 4 flavonoids using [C_4_C_1_im]­Br; on the other hand, when [C_6_C_1_im]Br and [C_8_C_1_im]Br were used, almost 100% recovery was reached for liquiritin apioside and about 80% for liquiritin. Some selectivity was also observed when the flavonoids hesperidin, hyperoside, and rutin were extracted from *S. tianschanica Rupr.* leaves.^1107^ Authors report that an increment in hyperoside extraction yield is observed when the IL was [C_6_C_1_im]­[BF_4_] instead of [C_4_C_1_im]­[BF_4_], while the opposite effect is observed for rutin. Zhang *et al.* extracted flavonoids baicalin, wogonoside, baicalein and wogonin from *Scutellaria baicalensis* using microwave-assisted extraction.^1105^ After screening several anions and imidazolium-based cation, the authors concluded that the overall higher extraction is reached using aqueous solutions of [C_8_C_1_im]­Br. However, results show that some selectivity can be achieved by changing the cation. For instance, 100% recovery of baicalin, wogonoside, and wogonin using [C_8_C_1_im]­Br, while about 60% recovery of baicalin and wogonoside and 100% recovery of baicalein and wogonin was reached using [C_6_C_1_im]­Br. Additionally, researchers also observed that the power, time, and [C_8_C_1_im]Br concentration exhibit different effects on extraction yield depending on the biomolecule, being usually positive for baicalin, wogonoside and negative for baicalein and wogonin, probably because the former are more stable molecules due to the sugar motif in the molecule.

Downstream process design post-extraction is essential for the application of IL extraction at industrial scale. These downstream processes are necessary to increase the purity of the biomolecules to obtain commercially viable products, and they should also lead to the recovery of the IL to be reused in the extraction process to reduce the environmental impact to the process and costs related to IL make-up. Some researchers have evaluated different approaches in order to purify biomolecules of interest and/or recycle the IL. In the work performed by Yangnsheng *et al.* to extract lactones from *Ligusticum chuanxiong* *Hort* using [((HO)^2^C_2_OC_2_)­C_1_C_1_N]­[C_2_CO_2_], the IL is recycled by precipitating lactones using methanol and removing the methanol through evaporation.^1093^ Unfortunately, the recovery significantly decreased after the second recycle. Extraction from the [((HO)^2^C_2_OC_2_)­C_1_C_1_N]­[C_2_CO_2_] using *n*-hexane after methanol precipitation improved the extraction capacity of [((HO)^2^C_2_OC_2_)­C_1_C_1_N]­[C_2_CO_2_], suggesting that some molecules remain soluble in [((HO)^2^C_2_OC_2_)­C_1_C_1_N]­[C_2_CO_2_] after methanol precipitation impairing the recovery of lactones. The formation of an aqueous biphasic system was evaluated by Yang *et al.* to recover chlorogenic acid from *Boehmeria nivea L.* after extraction using an aqueous solution of [C_4_C_1_im]­[HSO_4_].^1080^ Researchers evaluated the formation of aqueous biphasic systems for purifying chlorogenic acid by adding KH_2_PO_4_, NH_4_Ac, Na_2_SO_4_, or (NH_4_)_2_SO_4_ to the IL solution. They reported that Na_2_SO_4_ and (NH_4_)_2_SO_4_ led to the formation of a second aqueous phase, with the former more efficient in recovering chlorogenic acid with an extraction efficiency of 96.2% at optimum conditions. After separating both aqueous phases, chlorogenic acid was recovered from the IL rich phase through liquid–liquid extraction using *n*-butanol to reach a final product containing 81.3% chlorogenic acid. Additionally, the IL was recycled once to perform a second extraction, reaching an extraction efficiency of 91.6%. Unfortunately, no comments about the purity of the second recycling and the amount of IL recovered is mentioned in the study. Using a similar approach, Tan *et al.* recovered flavonoids from *Apocynum venetum* leaves.^1106^ In this case, 1-butyl-3-methylimidazolium diacyanamide ([C_4_C_1_im]­[N­(CN)_2_]) was used as IL and (NH_4_)_2_SO_4_ was used to form the aqueous biphasic system. *N*-Butanol was also utilized for recovering flavonoids from the IL rich phase (Figure 58). Although the authors report that similar results are obtained in the partition of flavonoids in the aqueous biphasic system when the recycled IL was used, there are no comments about the effect of the recycling over the extraction yield of flavonoids.

**Figure 58:**
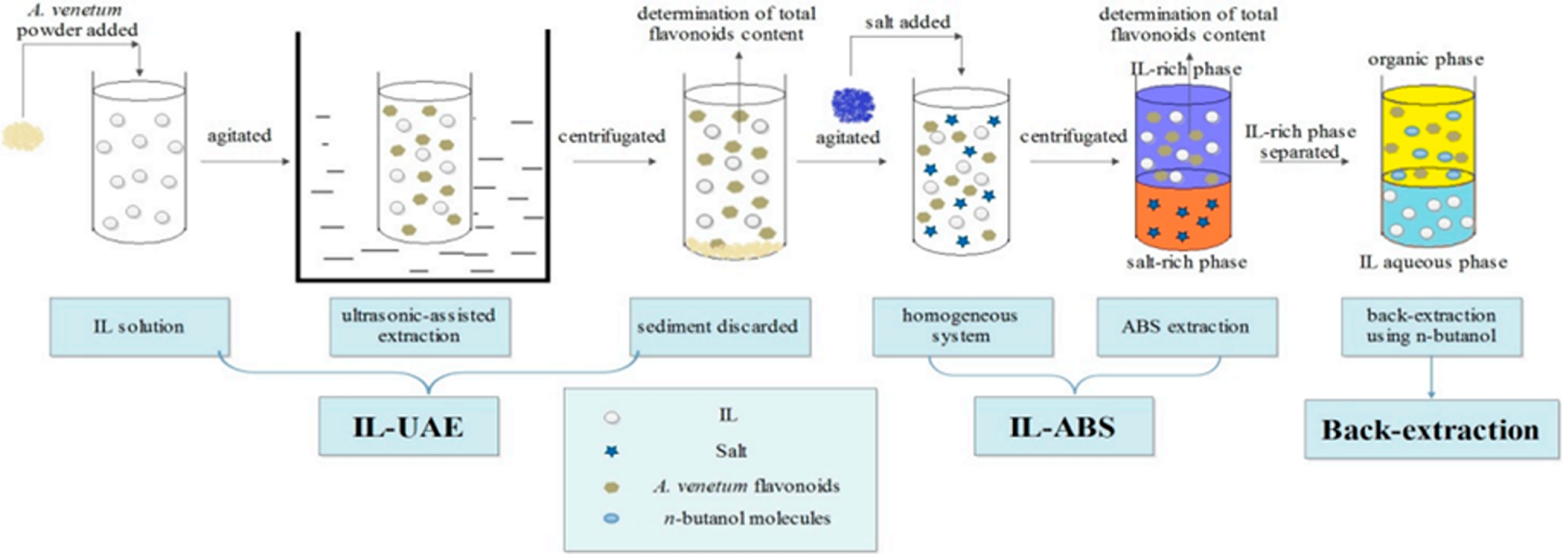
Flavonoids purification process from *Apocynum venetum* leaves. Adapted with permission from ref 1106. Copyright 2016 MDPI under CC BY 4.0 (https://creativecommons.org/licenses/by/4.0/).

Wang *et al.* also used aqueous biphasic system after biomolecule extraction. Researchers extracted flavonoids and iridoids from *Eucommia ulmoides* using [Ch] tryptophanate ([Ch]­[Try]).^1120^ After extraction, aqueous biphasic system was formed using K_3_PO_4_ to obtain an IL rich phase containing flavonoid, a salt-rich phase and an intermediate phase containing irioids. Also focused on polyphenols recovery, Chen *et al.* evaluated a process to purify salicin, hyperin and rutin from *Populus alba* × *P. berolinensis* after extracting them using a vacuum microwave-assisted extraction method with aqueous solutions of [C_4_C_1_im]­[BF_4_].^1077^ Authors also evaluated the recyclability of [C_4_C_1_im]­[BF_4_]. Polyphenols were absorbed in a column filled with D101 resin, while [C_4_C_1_im]­[BF_4_] was not adsorbed. Then, [C_4_C_1_im]­[BF_4_] was washed from the column using water and a fraction rich in polyphenols was obtained by desorbing them using a 60% ethanol solution. It was demonstrated that extraction yield is not impaired after 5 cycles of [C_4_C_1_im]­[BF_4_]. Unfortunately, the recycling process requires large amounts of water (about 4.5 equiv) and ethanol solution (about 4.5 equiv). The same group extracted flavonoids hesperidin, hyperoside and rutin from *S. tianschanica Rupr.* leaves using vacuum microwave-assisted extraction followed by resin purification.^1107^ In this case, the IL [C_6_C_1_im]­[BF_4_] delivered the best extraction performance and an AB-8 resin was used to recover the polyphenols. Unfortunately, the IL recyclability was not evaluated. Similarly, Ji *et al.* evaluated the resins *N*-vinylpyrrolidone and divinylbenzene to recover the flavonoids glabridin, glycycoumarin, isoangustone, licoricidin, and licoisoflavone from an aqueous solution of [C_8_C_1_im]­[BF_4_] after extracting *Glycyrrhiza uralensis* roots.^1115^ [C_8_C_1_im]­[BF_4_] and flavonoids were adsorbed by the resin. [C_8_C_1_im]­[BF_4_] was recovered from the adsorbent using a 60% methanol solution, while flavonoids were recovered using pure methanol (78.9% recovery). In this case, recyclability of [C_8_C_1_im]­[BF_4_] was evaluated after methanol evaporation showing that after 3-cycles the recovery of flavonoids was not significantly impaired.

Jiao *et al.*, after optimizing the extraction of essential oils from *Dryopteris fragrans* using aqueous solutions of [C_2_C_1_im]­[C_1_CO_2_], evaluated a process to recover essential oils by coupling a microwave-assisted extraction followed by a microwave-assisted hydrodistillation (Figure 59).^1122^ After recovering the essential oils, absolute ethanol was added to the aqueous solution to recover the IL through an azeotropic distillation of ethanol and water. Authors showed that [C_2_C_1_im]­[C_1_CO_2_] loses about 50% of its extraction capacity after 3-recycling steps, but extraction capacity remains stable during cycles 4 and 5.

**Figure 59:**
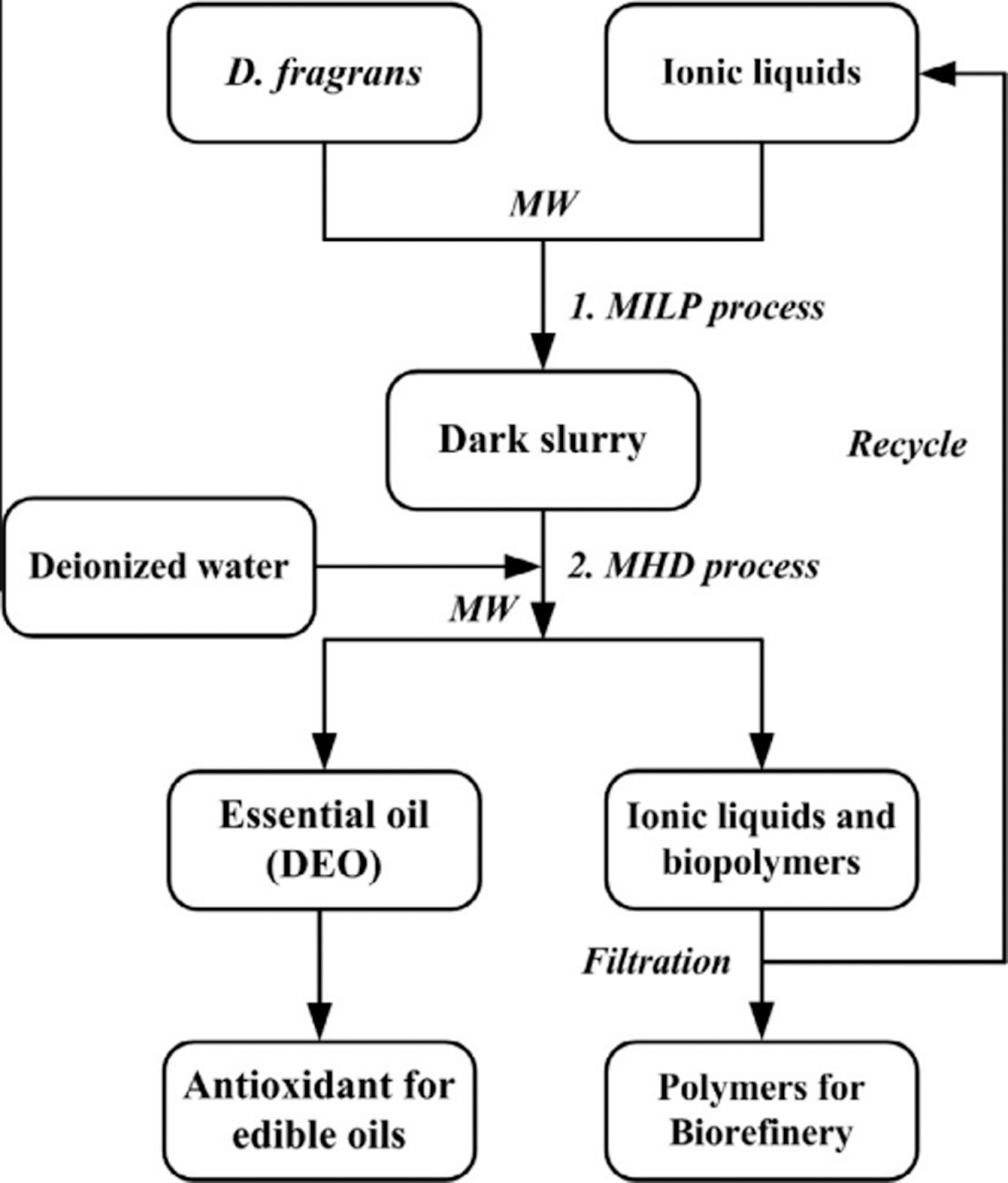
Essential oils recovery from *Dryopteris fragrans* using [C_2_C_1_im]­[C_1_CO_2_]. Adapted with permission from ref 1122. Copyright 2013 Elsevier Ltd.

Chen *et al.* developed a process to recover paeonol and paeoniflorin using an aqueous solution of IL.^1084^ The process consists in a microwave-assisted extraction coupled to a distillation system to distillate paeonol while paeoniflorin is extracted. After extraction using [C_4_C_1_im]­Br, distilled paeonol was purified by dissolving it in hot water and crystallizing paeonol by cooling the solution. After 2 crystallization steps, 87.6% yield and 94.8% purity were reached. Additionally, the aqueous solution containing [C_4_C_1_im]Br and paeoniflorin was treated using the microporous resin HPD100B in order to retain [C_4_C_1_im]Br and paeoniflorin in the resin; then, water was used to desorb [C_4_C_1_im]Br and an ethanol–water solution (40% v/v) was used to recover paeoniflorin reaching 23.6% of purity. Recycling of [C_4_C_1_im]Br was evaluated over 5 cycles, showing that paeonol recovery is not affected due to the recycling, while [C_4_C_1_im]Br and paeoniflorin recoveries are constantly reduced after each recycling run.

Ressman *et al.* developed a process to recover betulin from Birch bark using [C_2_C_1_im]­[C_1_CO_2_], which is depicted in Figure 60.^1096^ The process begins by extracting the biomass using [C_2_C_1_im]­[C_1_CO_2_] in a microwave heated system at 100 °C during 15 min followed by adding ethanol to precipitate biopolymers. The betulin is recovered from the IL using 20% water as antisolvent, reaching up to 30% recovery and 87% purity. Finally, the filtrate is evaporated to remove water and ethanol to recycle the [C_2_C_1_im]­[C_1_CO_2_]. The authors demonstrated that after 3 recycling steps the IL loses performance and betulin recovery is reduced by about 30%, and further losses in recovery are observed in additional recycling steps.

**Figure 60:**
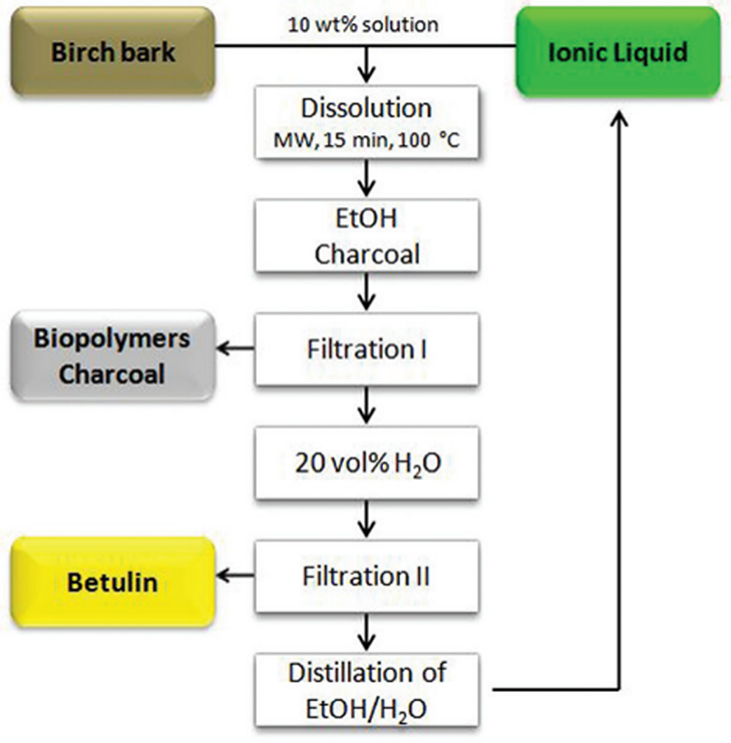
Process to recover betulin from Birch bark using [C_2_C_1_im]­[C_1_CO_2_]. Adapted with permission from ref 1096. Copyright 2012 Royal Society of Chemistry.

An interesting study was performed by da Costa Lopes *et al.* to recover biopolymers and biomolecules from wheat straw.^1081^ The authors recovered lignin, cellulose and hemicellulose by dissolving the biomass in [C_2_C_1_im]­[C_1_CO_2_] followed by cellulose precipitation adding a 3% (w/v) NaOH solution; then, the filtrate was concentrated, pH was adjusted, and ethanol was added to precipitate hemicellulose. The solid fraction was washed several times with water. Finally, ethanol was evaporated and the aqueous solution acidified using HCl to precipitate a lignin-rich fraction. After biopolymer recovery, the obtained filtrate was neutralized using NaOH and the water was evaporated to obtain a solid residue which was extracted using acetonitrile to recover the IL. The IL was dried under vacuum to remove acetonitrile and the remaining water before polyphenols extraction. In order to extract the polyphenols from the recovered IL, the researchers evaluated adsorption resins such as silica C-18, XAD-2, XAD-7 and PVPP. The IL was contacted with the adsorption resins to adsorb the polyphenols; then, the resin was recovered, washed with water, and polyphenols were desorbed using 96% methanol. Results show that XAD-7 resin is the best option to recover polyphenolic compounds from the recovered [C_2_C_1_im]­[C_1_CO_2_]. Further purification of polyphenols was achieved using supercritical CO_2_ extraction. Unfortunately, the authors did not show results related to the recyclability of [C_2_C_1_im]­[C_1_CO_2_] after the polyphenols recovery, but it is an intriguing biorefinery concept utilizing recovery of biopolymers and biomolecules from a raw material. Another work which aims to seize the whole potential of the biomass was performed by Papa *et al.* ^1123^ They pretreated loblolly pine using [C_2_C_1_im]­[C_1_CO_2_] to obtain fermentable sugars and recover the terpene α-pinene. Recover of α-pinene was evaluated using 2 different methods: (1) hexane was used to extract α-pinene from the IL solution after the pretreatment, and (2) dodecane was added in the pretreatment to extract α-pinene in a hydrophobic phase during the process (one-pot process). Both methods reach over 80% α-pinene recovery, while glucose released from the pretreated biomass seems unaffected by α-pinene recovery. A techno-economic analysis performed by the authors shows that the minimum selling price of ethanol produced from the sugars recovered from the biomass decreased from $5.8 per gallon to <$1 per gallon considering a selling price of α-pinene of $2.3–3.0 per kg. However, as in the study of da Costa Lopes *et al.*, [C_2_C_1_im]­[C_1_CO_2_] recyclability is not evaluated, which is essential for the scalability of the process.

Feng *et al.* developed a process to recover stilbene glycoside and anthraquinones from *Polygonum multiflorum* roots using ILs (Figure 61).^1098^ First, stilbene glycoside is extracted using an aqueous solution of *N*-butylbenzothyazolium tetrafluroborate ([C_4_Bth]­[BF_4_]) at rt and recovered from the solution using *n*-butanol, which leads to a powder rich in stilbene glucoside upon evaporation of *n*-butanol. Anthraquinones are extracted from the residual solids recovered after the first extraction using an aqueous solution of *N*-butylbenzothyazolium *para*-toluenesulfonate ([C_4_Bth]­[*^p^* C_1_PhSO_3_]) in an ultrasound-assisted extraction system. After extraction, the [C_4_Bth]­[*^p^* C_1_PhSO_3_] is removed from the solution using strongly acidic cation-exchange resins and a powder rich in anthraquinones is recovered upon evaporation. [C_4_Bth]­[*^p^* C_1_PhSO_3_] was recovered from the resin using an aqueous solution of HCl. Although [C_4_Bth]­[BF_4_] and [C_4_Bth]­[*^p^* C_1_PhSO_3_] recovery is mentioned by the authors, unfortunately, there is no reported evidence to show the recyclability of the ILs in the process. Also, there is no mention regarding the final purity of the produced powders. Wang *et al.* also used *n*-butanol to recover the biomolecules from an aqueous solution of IL.^1111^ They extracted flavonoids from *Phyllostachys heterocycle* leaves using an aqueous solution of [C_4_C_1_im]Br and recovered the flavonoids using butanol to reach a recovery of 97.9% of flavonoids, but recyclability of the ILs was not evaluated.

**Figure 61:**
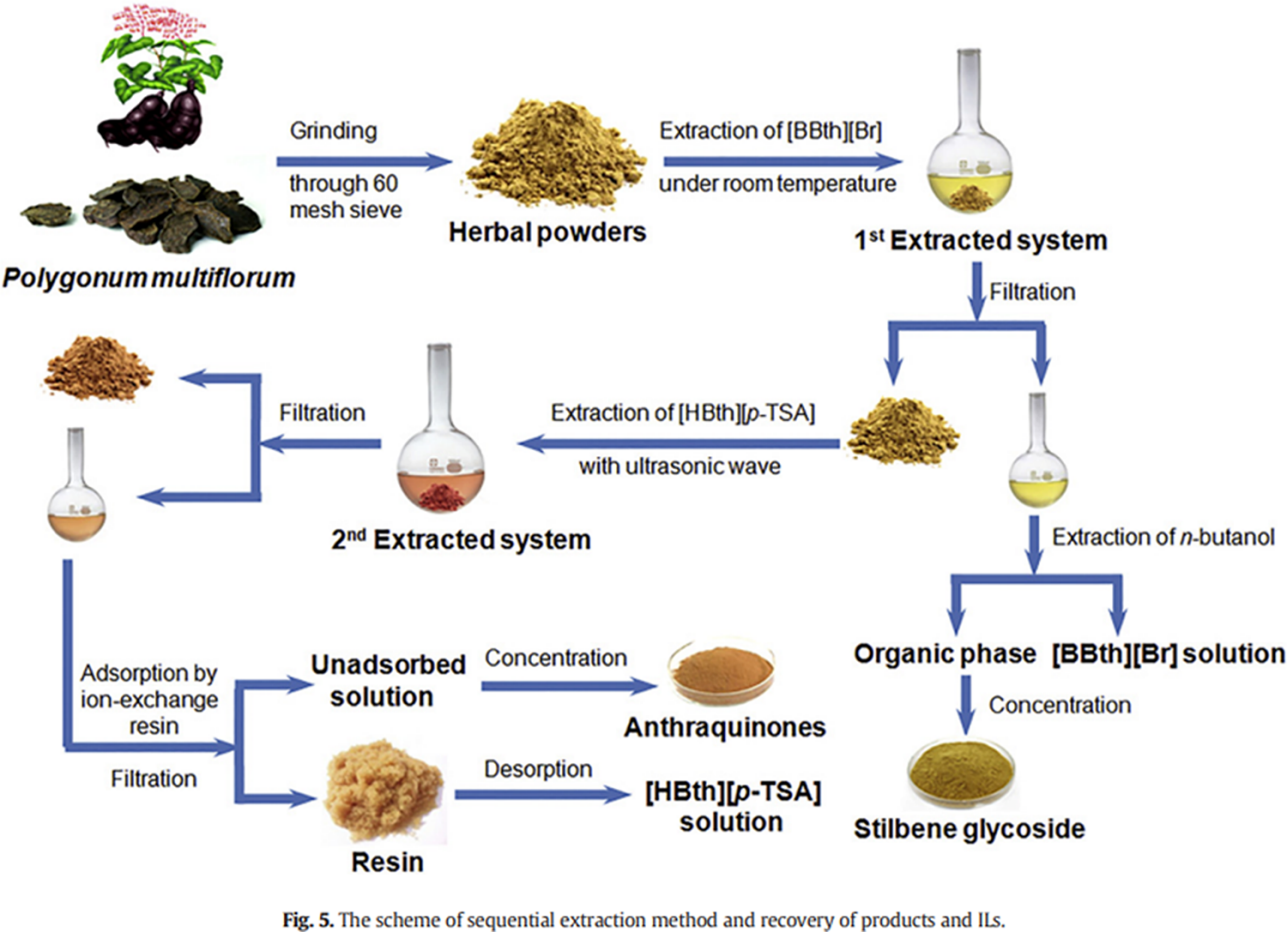
Recovery of stilbene glycoside and anthraquinones from *Polygonum multiflorum* roots using ILs. Adapted with permission from ref 1098. Copyright 2017 Elsevier Ltd.

Resins were also used by Yang *et al.* to recover alkaloids from aqueous solution of [(C_1_=C_2_)­C_1_im]­Br.^1070^ Several resins were evaluated and the best performance was obtained using the macroporous resin HPD750. The process consisted in diluting the extract with water and then adsorbing the alkaloids and [(C_1_=C_2_)­C_1_im]Br in the resin. [(C_1_=C_2_)­C_1_im]Br was recovered using water and alkaloids were recovered using ethanol–water mixtures. In order to recycle [(C_1_=C_2_)­C_1_im]­Br, the aqueous solution of [(C_1_=C_2_)­C_1_im]­Br obtained from the resin desorption was treated with activated carbon to remove impurities. The authors found that after 5 cycles the extraction efficiency of [(C_1_=C_2_)­C_1_im]­Br was not affected.

De Faria *et al.* evaluated the extraction of cynaropicrin from *Cynara cardunculus L.* leaves using aqueous solutions of the surface-active IL [C_14_C_1_im]­Cl.^1094^ After optimizing extraction conditions, the researchers propose a process to extract and recover cynaropicrin from the aqueous solution of [C_14_C_1_im]Cl by diluting the aqueous solution with water, which leads to cynaropicrin precipitation (65% precipitated); then, water was evaporated and the aqueous solution of IL recycled to extract another batch of raw material (Figure 62). Unfortunately, data related to the recycling of the IL solution was not reported in this work.

**Figure 62:**
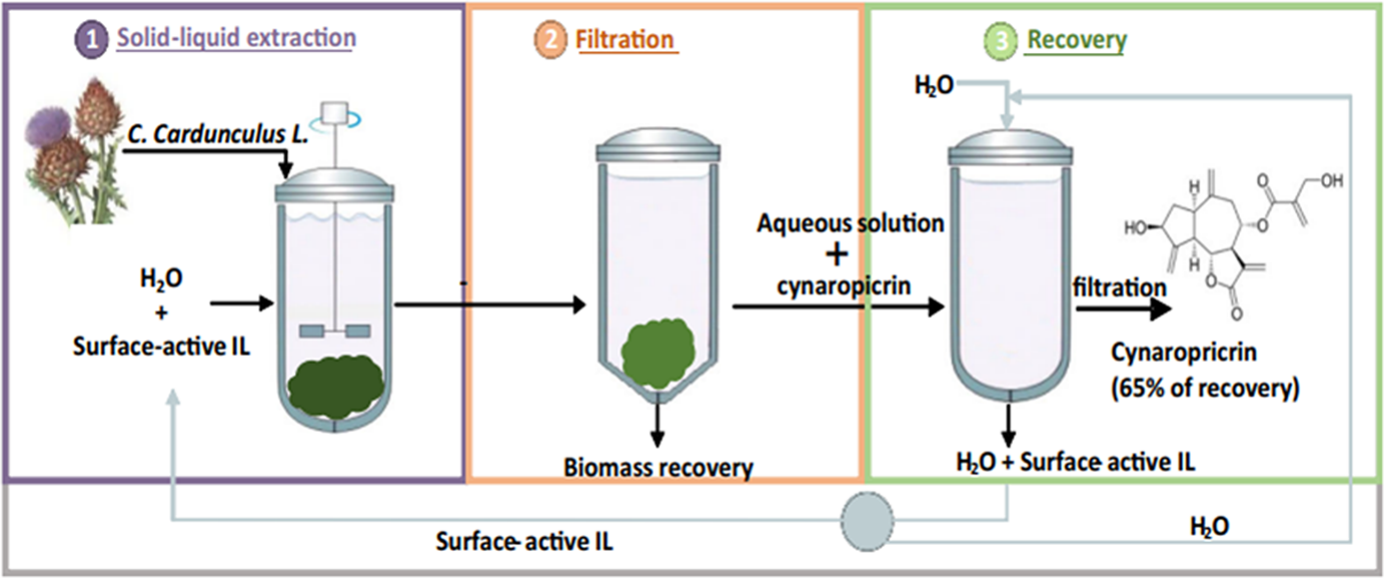
Cynaropicrin purification from *Cynara cardunculus L.* leaves using [C_14_C_1_im]­Cl. Adapted with permission from ref 1094. Copyright 2018 Springer Nature.

Aqueous biphasic systems to purify biomolecules from extract previously obtained using conventional techniques has been proposed for some authors.^1130–1132^ For instance, Xu *et al.* studied the extraction and purification of acteoside from *Cistanche tubulosa* stems.^1131^ First, an extract was prepared using a 50% ethanol solution and a powder was obtained after removing the solvent. Then, the extract was treated using an aqueous two-phase system constituted by [C_4_C_1_im]­[BF_4_] and (NH_4_)_2_SO_4_ in water in order to separate acteoside from its glucoside echinacoside. The authors demonstrated that by using this system it is possible to recover 98.2% of acteoside with a selectivity of 216.5 expressed as the ration between the partition coefficients between the two phases. Solvent extraction and adsorption methods were evaluated to recover acteoside from the IL-rich phase, concluding that using single wall carbon nanotube over 95% of acteoside is removed from the IL-rich phase which can later be efficiently recovered with a solution of methanol/acetic acid/water (1:1:8). Unfortunately, the recyclability of the IL was not evaluated.

An interesting approach to purify plant sterols from tall oil was proposed by Aravena and Hallet.^1133^ Tall oil is an oily residue obtained from the Kraft process to produce cellulose paste, which is rich in fatty acids and resin acids. To recover plant sterols, the authors synthesized an in situ PIL using the acids presented in the tall oil by adding triethylamine; then, plant sterols were precipitated by adding water and methanol to obtain products with over 95% purity. The same authors also showed that triethylamine could be recovered from mixtures with fatty acids to be re-used in a new precipitation cycle.^1086^

## 3 Implementation, Perspectives, and Challenges of Biorefineries Based on ILs and DESs

Technologies based on the use of ILs and DESs are very promising candidates for the development of successful biorefineries that will unlock the full potential of lignocellulosic biomass to produce energy and manufacture chemical products, including biofuels, platform chemicals, and materials, matching the diversity and flexibility of petroleum refineries. Nevertheless, the industrial implementation of biorefineries, in particular those based on ILs and DESs, still faces many challenges, including feedstock supply (including transportation) and heterogeneity (as it varies from different species, growing conditions, etc.), compatibility of ILs and DESs with current equipment (*e.g.,* corrosion issues associated with acidic ILs), handling issues (*e.g.,* the high viscosity of some ILs might hinder their application in certain processes), thermal and chemical stability and recyclability of the solvents, health, safety and environmental concerns, energy usage, etc.^9^

It is possible to make high level assessments about solvent costs and impacts of the implementation of new solvents, including ILs and DESs, at the development stage of any process, considering the feedstocks and synthetic procedures that will be employed. These considerations should be used to make informed decisions about the solvents of choice for each process. Moreover, tools such techno-economic analysis (TEA) allow for more in-depth estimations. These assessments should be made following a cradle-to-cradle approach, a holistic framework for the development of waste-free production systems that frames the circular economy, to comply with increasing policy requirements and public demands.^9^

### 3.1 Sustainability Assessment of Biorefineries Based on ILs and DESs

TEA is a powerful tool for pointing out the feasibility of a process, helping to identify at an early stage the main challenges and bottlenecks that compromise the economic and technical viability. It normally comprises the calculation of capital investment, operating costs, and minimum selling price of a selected product (normally ethanol, MESP) by carrying out energy and mass balances from simulations based on experimental data.^1134^

In order to make biomass treatment with ILs a profitable technology at a large scale, there are some challenges that need to be overcome, namely, high solvent cost and its recovery and reuse, solid loading during pretreatment and particle size. The first study that analyzed the effects of these main factors on the process economics was that carried out by Klein-Marcuschamer *et al.* (2011), where they studied the effect of IL cost between $2.5/kg and $50/kg, IL/biomass ratio between 1 and 10 and IL recycling rate between 94% and 99.6% on an ethanol-based biorefinery.^43^ The pretreatment conditions were fixed at 120 °C and 30 min and the saccharification ran for 10 h. They found out that lowering IL cost was the most important factor to make the process profitable, since other aspects had limited impact without lowering IL price. Also, reducing IL loading was more effective than increasing IL recycling rate, as it lowers capital cost, electricity usage and operating costs. Finally, they posited that looking for high value-added lignin products can very effectively lower the MESP, even to a point that lignin becomes the first revenue of the biomass treatment process.^43^

Based on these results, Konda *et al.* (2014) studied the main cost drivers of two post-pretreatment process configurations, water washing and one-pot, a configuration that aims to performing the IL pretreatment and subsequent steps in the same vessel, under the most cost-beneficial conditions from the previous study.^45^ They also analyzed the impact of biomass loading, since its effect was different for both scenarios. Their results suggested that the water consumption is the main cost in the water washing scenario, whereas the acid/base cost (needed during the sugar extraction and recovery) is the most impactful in the one-pot approach. In both scenarios, increasing biomass loading was found to be essential for the economics of the process, being more important in the one-pot approach. At a 50% biomass loading, the MESP was always above $4/gal, even after optimizing water or acid/base usage, and was only lowered by the inclusion of lignin as a selling product in the biorefinery, pointing out once again the key role of lignin valorization.^45^ Thus, the one-pot approach, although very promising, needs some improvements to become economically feasible, so some research focused on carrying out TEA on variations of the one-pot process. Sun *et al.* (2017) studied the one-pot treatment of switchgrass with the PIL [(HO)^2^C_2_N]­[C_1_CO_2_] removing the washing and the pH adjustment steps, as it was previously proven that were two main cost-drivers of the biomass pretreatment with ILs.^45,141^ Under optimized conditions, an ethanol yield of 70% was achieved, and operating costs were calculated with a 40% reduction of the MESP compared to the counterpart that needed pH adjustment.^141^ In addition, they identified possible improvements during the SSF stage of the process. Another possible improvement of the one-pot process is to aim for a multiproduct biorefinery, as it was shown promising with ethanol and lignin co-production before.^237^ In this sense, Zhang *et al.* (2020) evaluated the potential of including a simultaneous furfural production by incorporating a MIBK phase that extracted the furan compound during the pretreatment, reaching a 49% carbon conversion into furfural, lignin, and ethanol and thus reducing the furfural selling price by a 37.5%.^1135^ A similar approach to the one-pot process was carried out by Achinivu *et al.* (2022), where the one-pot was carried out between PIL synthesis and pretreatment, and not between pretreatment and hydrolysis and fermentation. They reported a decrease in the operating costs of around 50% compared to the normal PIL synthesis followed by the pretreatment in separate stages.^265^

Ethanol is normally considered the first product of the IL-based biorefinery, since its production is well established via fermentation. However, there are still some challenges to make this technology competitive. Oleskowicz-Popiel *et al.* (2014) studied the production of lignocellulosic ethanol without the use of enzymes after the IL pretreatment. Their model was not intended to be totally accurate, but as a tool to guide scientists and identify the main challenges. It was discovered that the MESP on the IL-based acidolysis was expensive compared to established technologies, but it could be lowered by $4/gal by optimizing aspects not directly related to the IL pretreatment step, such as the hydrolysis yield, the sugar recovery, and extraction efficiency.^44^ More recently, Leal Silva *et al.* (2022) performed a TEA of the production of 2G ethanol via PIL pretreatment and washed the pretreated pulp with four different washing methods. In the best scenario, the ethanol yield was increased up to 33% due to an increase in the saccharification yield and the more efficient counter-current washing method. The production of technical lignin by this process was also considered, leading to an increment on the internal rate of return of 2 percentage points.^191^

Apart from the aforementioned, there are other cost drivers of the IL pretreatment process. Baral and Shah (2016) focused on understanding them at a larger scale of 113 million L/year cellulosic biorefinery. They found out that, apart from IL cost and recovery, heat recovery, and integration and pretreatment severity factors played a major role in the economics of the process, as they highly impacted the operating costs.^190^ It was calculated that 90% heat recovery was necessary to make a $1/kg IL suitable for biomass processing with a 97% recovery rate. In addition, they considered three different feedstocks (corn stover, switchgrass, and poplar) and showed that they were all economically competitive, especially corn stover due to a higher availability. The environmental sustainability of the IL pretreatment process was assessed with satisfactory results.^190^

One of the main challenges to make IL-based pretreatments economically viable is the IL price. In that sense, the search for cheaper ILs was crucial for developing an IL-based pretreatment technology. Brandt-Talbot *et al.* (2017) developed a TEA of the ionoSolv process, that is, using cheaper protic ILs, with a 99% IL recovering rate and 4 times reused from experimental results. They found that the ionoSolv process with PILs presented lower operating costs than those for benchmark processes, such as dilute acid pretreatment, and highlighted some critical points that needed to be further addressed, such as solid loading, heat integration, process scale-up and pretreatment conditions.^174^

The effect of the operating conditions during pretreatment had been previously identified as a knowledge gap that needed to be addressed to improve the economic performance of IL-based biorefineries.^174^ In this sense, and for the water washing approach explained before, Ovejero-Pérez *et al.* (2021) pretreated *Eucalyptus* with [C_2_C_1_im]­[C_1_CO_2_] and [Ch]­[C_1_CO_2_] and washed the recovered pulp with different amounts of water to test the effect of the employed water on sugar yields and the economics of the process. They calculated higher operating costs in the IL recovery step with incremental amounts of water in the washing fraction. However, it was necessary to wash with 5.5 g water/g IL to ensure that all the IL was recovered without excessive washing, minimizing the IL make-up costs and thus the total reutilization costs.^1136^ Additionally, Ferrari *et al.* (2021) examined other pretreatment operating conditions (temperature 80–130 °C, water content in the IL 20–60%, and solid loading 10–30%) impact on an ethanol-selling biorefinery. Solid loading and water content in the IL were found to be the most impactful parameters on energy consumption rather than pretreatment temperature. Surprisingly, the IL price showed very little effect, as the proposed IL recovery rate was established at 99%, making IL price unimportant.^192^

Overall, IL-based pretreatment of biomass for chemicals or fuel production is a promising alternative to other types of pretreatments, but economic evaluation is still needed to successfully implement the technology. The main bottlenecks in the process are IL price, IL recyclability, water consumption, process conditions and biomass loading. IL price has been addressed by the inclusion of PILs, much cheaper than conventional aprotic ones, whereas recycling rate is generally established to be 96–99%. Water washing has been optimized by different approaches, either minimizing water consumption or by using different washing alternatives. One of those is the one-pot process, although presenting some challenges is a promising alternative to reduce water consumption post-pretreatment. Another alternative that has been proposed to reduce the hydric footprint is the replacement of fresh water by seawater.^1137^ Although this concept has not been exploited yet for 2G biorefineries that fractionate lignocellulose, the use of seawater has been demonstrated in 1G biorefineries that produce bioethanol, where life cycle analysis found reductions in water depletion of around 31% when using seawater instead of fresh water.^1137^ For hydrothermal reactions, the seawater salts helped improve the efficiency of the hemicellulose removal. The presence of ions that can act as catalysts in biomass processing and as a source of nutrients for bioconversion processes is another potential advantage of the use of seawater for biorefineries. On the other hand, excessive release of acids, that might lead to undesired degrees of depolymerization and the formation of degradation products, are potential issues.

The valorization of by-products has been proven to be beneficial, even a necessity, to improve economic feasibility. Biomass solid loading remains one of the most challenging issues to face regardless of the process configuration employed. In that regard, process intensification should be carried out to try increasing the solid loading and minimizing biomass conditioning, which can be done by optimizing stirring and stirrer design.^182^ Not only that, process intensification should be performed in order to reduce wastewater after the pretreatment process, minimizing or reusing water use,^714,1138^ as well as to overcome the challenges identified by TEA and research focus in that direction is necessary.

Apart from economics, the environmental impact of a process is an important aspect to consider. Life cycle assessment (LCA) is a powerful tool commonly used to evaluate environmental impacts of a process throughout the entire life cycle. However, there is an important knowledge gap in this sense regarding biomass pretreatment with ILs. The first study on ILs with biomass (or biomass-derived compounds) performed a cradle to gate LCA comparing the dissolution process of cellulose with [C_4_C_1_im]Cl and with NMMO/H_2_O, the most ecofriendly dissolution process known to date. It was shown that the IL was in general more environmentally friendly, giving rise to interest in the employment of ILs for biomass processing. However, there were still some impacts, especially from precursor synthesis, pointing out the importance of considering the whole chain to assess environmental impacts.^1139^ LCA and TEA often go hand in hand, so some of the works already explained in the TEA section also covered some environmental study. For example, Ferrari *et al.* (2021) conducted an LCA under different process condition scenarios and found out that the IL production had the largest impact. Thus, scenarios that minimized IL make-up were more environmentally friendly.^192^ Achinivu *et al.* (2022) also performed a LCA of their new process configuration of PIL synthesis + pretreatment. Their findings suggested that the new in situ PIL synthesis was environmentally beneficial compared to traditional PIL synthesis approaches due to the elimination of solvents and reduction of the energy required for product separation, drying and cooling processes.^265^ In an in-depth study on the environmental impacts of biomass pretreatment with ILs, Baaqel *et al.* (2020), used the concept of monetization of endpoint impact indicators to calculate the total cost (also considering solvent production costs) of employing acetone, glycerol, [C_1_im]­[HSO_4_], and [C_2_C_2_C_2_N]­[HSO_4_] for biomass processing. They showed that [C_2_C_2_C_2_N]­[HSO_4_] presented the lowest total costs of all solvents, mainly attributed to a higher recyclability, which ends up being beneficial for both the economics of the process and the environment.^259^ Murali and Shastri (2022) conducted LCA for ethanol production with 32 process combinations (8 pretreatment processes and 4 different hydrolysis–fermentation scenarios) and concluded that the dilute acid pretreatment with SSF presented the lowest environmental impacts. Interestingly, the estimated IL pretreatment impacts were in the middle–upper positions, pointing out the necessity of process intensification and improvement, mostly by reducing waste and increasing IL reuse.^1140^

TEA and LCA are powerful tools to analyze and understand a case study process. On their own, each can help us assess which actions could be the most important and beneficial for a given process. Combined with each other (or with other similar tools) their potential to help the decision-making process is maximized from both economic and environmental perspectives.

### 3.2 Technology Scale-up

#### 3.2.1 Supply Chain and Logistics of ILs and DESs

Solvent availability at reasonable cost is a major concern for the development of IL and DES-based biorefineries. Ensuring a reliable supply source at industrial scale for ILs and DESs and their precursors is key for the viability of these technologies. Although they are relatively new types of solvents, several examples of their implementation at industrial scale and capability to produce ILs at the ton scale already exist, as discussed in section 2.5.1. However, there are still concerns related to their manufacturing at the required scale.

The synthesis of many ILs from its fundamental precursors requires many different synthetic stages, involving nonrenewable feedstocks and hazardous intermediates. This poses a challenge to the sustainability claims of their implementation by simply moving the sustainability, environmental and health concerns back along the production process. This is particularly concerning for some of the most widely used AILs.^1134^ It should be highlighted that the synthesis of ILs and DESs using less polluting and energy intensive synthetic procedures that rely on enterally renewable feedstocks is theoretically feasible, *e.g.*, amines to be used as cation source could be prepared from atmospheric nitrogen and hydrogen and bio-alcohols produced renewably; [Ch] salts could be sourced from fermentation rather than petrochemical synthesis, etc. To ensure the future viability of industrial processes based on IL and DES, these sustainable synthetic procedures need to be fully developed and implemented. However, sourcing from renewable feedstocks is often pricier than their nonrenewable counterparts (significantly so for [Ch] or bio-based amines), which still hinders their potential. On the other hand, preparation of ILs from biomass building blocks, including sugars and lignin monomers, and their use in biomass processing has been reported.^228,260^

#### 3.2.2 Technological Challenges

##### 3.2.2.1 Synthetic and Utilization Costs of the ILs and DESs

The industrial implementation of ILs and DESs in biomass pretreatment still faces many challenges. A significant hurdle is their steep cost due to intricate synthesis and purification processes, often costing tens to hundreds of dollars per kilogram. This cost impact on biorefinery economics is substantial.^1141^

AILs proficiently disintegrate biomass structure, aiding cellulose accessibility and lignin removal, but their cost poses challenges for extensive commercialization, potentially hindering biorefinery viability reliant on IL pretreatment.^1142^ To mitigate this, researchers and industry experts strive to cut synthesis and purification expenses, enhance recycling and uncover lower-cost alternative pretreatment methods. Achieving a balance between technical merits and economic factors is pivotal for successful large-scale biorefinery integration.^163^ Addressing this, low-cost protic ILs were introduced for biomass pretreatment in biofuel production. These are synthesized by combining Brønsted bases and acids, costing less than $1 per kilogram.^470^ AILs’ cations maintain charge without equilibrium between neutral and ionized forms. However, PILs, after synthesis, establish enduring hydrogen bonding networks due to proton transport from acid to base, allowing coexistence of neutral and charged groups.^140^ In comparison to AILs, the potential of PILs for biomass pretreatment lies in their cost-effectiveness and reasonable conversion efficiency.^477^ Nevertheless, it is vital to acknowledge that the cost of PILs significantly outpaces that of conventional options like other acidic and basic reaction media. Despite their uncomplicated synthesis through Brønsted acid–base pairing, their cost raises pertinent practicality concerns.^477,1143,1144^ Additionally, there are not many companies that can supply ILs at a higher scale such as ton. For instance, Proionic (Raaba-Grambach, Austria) and iolitec (Heilbronn, Germany) seem to be the only manufacturers to produce custom IL batches up to the ton scale. Therefore, limiting the potential scale-up of IL process quantitatively and geographically (as it needs to be next to Europe to reduce transportation costs). Consequently, the demand for cost-effective methods to recover and reintegrate ILs is burgeoning.

Despite being recyclable, ILs’ effectiveness in biomass breakdown might wane over time, reducing efficiency in successive cycles, which may be caused to thermal degradation or the presence of impurities that remain between subsequent cycles of biomass processing.^1145^ Loss of ILs during pretreatment further complicates recycling and amplifies costs. However, the pilot plant of the EU-funded start-up Lixea, pioneering in the industrial application of ILs for biomass pretreatment, has worked on a continuous-flow with the same IL batch since it opened in May 2022 with a feedstock scale of 20 kg/batch, which highlights the recyclability of some ILs and the potential of the technology to be affordable and scalable.^245,1146^ In addition to the Lixea technology , the Advanced Biofuels and Bioproducts Process Development Unit, part of Lawrence Berkeley National Laboratory (California, USA) has also demonstrated the successful scale-up of the one-pot processing of mixed species of woody biomass using [Ch]­[Lys] to produce 2G ethanol.^183^

##### 3.2.2.2 Recovery and Recycling of ILs and DESs

Recovering ILs and DESs from the pretreated slurry is vital for cost reduction, as recycling them for biomass pretreatment aligns with the economic viability of refineries.^192^ Most of the efforts in literature have been towards the recovery of ILs as discussed here. In most reported washing procedures, the treated mixture is exposed to an extensive antisolvent wash to isolate solid remnants, often utilizing antisolvents such as acetone/water (1:1, v/v). Following this, the mixture is separated into solid and liquid components via filtration or centrifugation. The solid part is subjected to an additional water rinse to minimize IL content, while the liquid fraction (comprising water, acetone, IL and lignin) is distilled to eliminate acetone, inducing lignin precipitation. Ultimately, post segregating lignin from ILs, the water component is evaporated. Despite the introduction of techniques such as ultrafiltration and electrodialysis for IL recovery, these multistep procedures require amplified energy consumption and chemical degradation, resulting in escalated operational costs and heightened equipment investments for biofuel refineries.^1142,1147^

In terms of IL recovery rate and reuse performance, Nakasu *et al.* (2020) were able to recover 96.1–98.0% of the solvent by rinsing [C_2_C_1_N]­[C_1_CO_2_] pretreated sugar cane bagasse with sufficient water. Unfortunately, more than 86% of the IL degraded into *N*-(2-hydroxyethyl)­acetamide after the sixth reuse. Furthermore, as the IL recycling times increased, the lignin removal efficiency and molecular weight of the recovered lignin decreased.^178^

Outeiriño *et al.* employed a solution of acetone/water (1:1 v/v) for washing IL-pretreated biomass slurry, resulting in [Ch] glycinate recovery rates ranging from 81.8% to 96.7%, with no significant trends observed with increasing cycles.^1148^ The identification of the optimal antisolvent concentration, as advocated by Ovejero-Pérez *et al.*, who recommended a ratio of 5.5 g water/g IL to enhance refinery efficiency, becomes a critical consideration.^1136^ The emergence of alternative solvents with similar capabilities could markedly reduce the cost of recovering ILs from the treated residue.^1149,1150^ Besides conventional antisolvents like water, acetone and methanol, research indicates that ethanol can serve as an effective antisolvent for maximizing sucrose yield. Ethanol has been demonstrated to efficiently recycle PILs containing hydrogen sulfate anions.^1151^

Conversely, the use of enzymes and microbes tolerant to ILs could expedite the conversion process.^1152^ However, it is noteworthy that the common practice involves substantial post-pretreatment addition of water or buffer solution to dilute the IL concentration and enhance enzyme and microorganism efficiency.^1152,1153^ Nonetheless, this approach hampers achieving high biofuel concentrations during ensuing distillation or separation. In response, certain studies opt for minimal concentrations of ILs during pretreatment, succeeded by simultaneous saccharification and fermentation, resulting in higher biofuel concentrations.^334^ Yet, uncertainties persist concerning the recuperation of ILs and lignin from fermented and distilled slurries, as well as the quality of recuperated ILs for subsequent use. The use of intricate ILs that are toxic to enzymes and microorganisms for biomass pretreatment could impede biofuel production. Recent strides in synthesizing biocompatible ILs compatible with enzymes and microorganisms offer a potential solution.^259^

It should be noted that the company Proionic GmbH has developed a patent-pending “High Performance Recovery” technology for the recycling of IL during a biomass pretreatment process, based around the distillability of PILs with relatively low Δp*K*_a_ between the forming acid–base pair, such as [(HO)^2^C_2_N]­[C_1_CO_2_]. It applies a thin-film high viscosity evaporation process, allowing the recovery and recycling of the IL in nearly quantitative rates, while saving for purification stages and obtaining a pretreated biomass that is ready for further processing.^1154^

##### 3.2.2.3 Corrosion Issues Related with the Use of ILs

Material compatibility is a major challenge for the industrial applications of ILs. In particular, metal corrosion is a main concern. It is a natural process in which metals are converted to more chemically stable forms, like their respective oxides, hydroxides, or sulfides, by reactions with their environment. It results in the gradual degradation of materials and their properties (including strength). It can lead to structural and equipment failures of potentially catastrophic consequences. In 2002, it was estimated that the total direct cost of corrosion in the USA was equivalent to 3.1% of the country's G.D.P.^1155^ Metal corrosion has an electrochemical nature. Therefore, it is expected that the use of ILs will result in higher corrosion-related issues than low-conducting molecular solvents. Also, corrosion is enhanced by temperature.^1156^ Hence, it is a critical parameter for biomass processing applications, which are usually performed above 100 °C. For example, the high corrosivity of [C_2_C_1_im]Cl forced BASF to replace it by [C_2_C_1_im]­[C_1_CO_2_] for their commercial CELLIONIC formulation (IL solutions of cellulose).

Unfortunately, there is very little data about the corrosion behavior of ILs towards metals and the data available is mainly focused on fluorinated ILs. Only F. Malaret, in his Ph.D. thesis, has reported information about the corrosivity of acidic PILs, as the ones employed in the ionoSolv process for biomass pretreatment. His conclusions suggest that protic ILs are more corrosive than aprotic ILs due to the presence of protons that can be reduced to H_2_ and that both temperature and the presence of water promote corrosion. Also, that for PILs based on [HSO_4_]^−^ and Cl^–^, corrosion is metal dependent, with Fe, Al, Zn and Ti being more affected by the former, and Cu, Br, Ni, and some alloys of stainless steel being more affected by the latter.^1157^ On the other hand, some studies of ILs as corrosion inhibitors have also been published.^1158^ In brief, the cations of ILs can interact with the metal surface, forming a protective multilayer that protects metal surfaces by isolating them from the corrosive environment.^1158,1159^

## 4 Conclusions and Outlook

The high cost associated with many ILs and the potential toxicity of certain ILs to aquatic life demands nearly quantitative recoveries for industrial processes based on ILs. Careful assessment must be done on the cost of these solvents, their disposal and toxicity, and whether the benefits of their implementation can successfully counterbalance their potential shortcomings. The commercial potential of ionic liquids in biomass conversion is significant, offering significant advantages in terms of efficiency, sustainability and product diversity. While challenges exist, ongoing research and industrial interest are likely to drive key advancements that will make IL- and DES-based processes more economically viable and scalable. As such, ILs and DESs hold great promise for transforming the biofuels and bioproducts industries, contributing to a more sustainable and circular bioeconomy at the enterprise level.

One of the best indicators of the significant potential of ILs and DESs as key technologies used in future biorefineries are the successful deployment of several pilot plants for lignocellulose conversion, namely the Ioncell process for the production of cellulose fibers by dry-jet wet spinning from ILs, the Metsä Spring process, also producing cellulose fibers using ILs, the Dendronic process for biomass fractionation with PILs, implemented by Lixea, the use of one-pot biomass conversion and using distillable ILs for efficient biomass pretreatment developed at the Joint BioEnergy Institute licensed by Erg Bio and the high performance recovery process developed by Proionic to recycle distillable ILs employed in biomass pretreatment.
